# Visible Light Induced Mukaiyama Reagent Promoted Desulfurative Modification of Peptides and Proteins with Nucleotides

**DOI:** 10.1021/acscentsci.5c01241

**Published:** 2025-09-29

**Authors:** Mengran Wang, Yongjia Lei, Xinyu Song, Chunlin Wang, Quanping Guo, Xiuren Zhou, Wenbo Mao, Kuan Chen, Zhaoqing Xu

**Affiliations:** † School of Basic Medical Sciences, 12426 Lanzhou University, Lanzhou 730000, China; ‡ School of Pharmacy, 12426 Lanzhou University, Lanzhou 730000, China; § Key Laboratory of Preclinical Study for New Drugs of Gansu Province, School of Basic Medical Sciences, 12426 Lanzhou University, Lanzhou 730000, China; ∥ Research Unit of Peptide Science, Chinese Academy of Medical Sciences, 2019RU066, Lanzhou 730000, China

## Abstract

Site-selective modification of peptides and proteins serves as a powerful tool for biological research and therapeutic development. We present a visible-light-driven stereoretentive peptide/protein–nucleotide conjugation via Cys desulfurization, enabling C5-selective coupling with 6-azauracil nucleosides through stable C–C bond formation. Using Mukaiyama reagent (*N*-alkyl-2-halopyridinium) activation under visible light (400 or 420–430 nm), this method generates configurationally stable Ala radicals while avoiding the detrimental side effects associated with UVA irradiation. The disulfide-compatible system preserves native stereochemistry and accommodates diverse substrates including oligonucleotides, functionalized nucleosides, and drug conjugates in good yields. Biocompatible reductants (NADH/Hantzsch ester) further facilitate conjugation with various radical acceptors under mild conditions. This approach established a versatile platform that enables both precision modification of peptides/proteins and investigation of structure–function relationships in peptides, proteins, and nucleic acids under physiologically relevant conditions.

## Graphical abstract

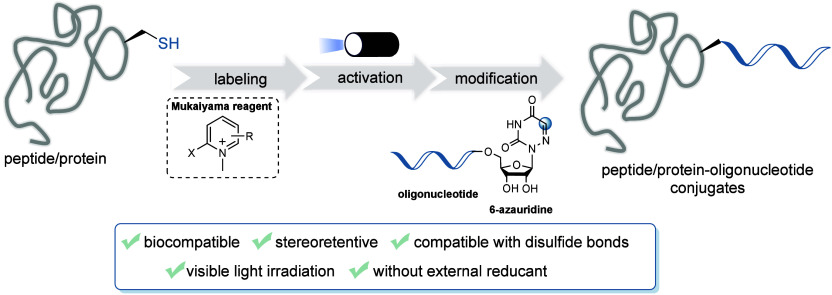

## Introduction

Site-selectively modifying peptides or proteins with fluorophores, functional groups, drug molecules, or bioactive substances provides invaluable tools for studying and manipulating biological systems, as well as for developing therapeutic and diagnostic agents.^1^ However, targeting specific amino acid residue in peptides or proteins to achieve site-selective installation of useful groups under biocompatible conditions, i.e., in an aqueous environment, at near neutral pH, and <37 °C remains significant challenges.^2,3^ Although noncanonical amino acids can be incorporated into proteins as unique reactive handles via the technique of amber codon suppression, the methods can be limited due to the complexities of the technique and the chemistry available for reaction.^4^ The total synthesis of modified peptides and proteins based on native chemical ligation (NCL) is another powerful technology in the study of post-translational modifications (PTMs).^5^

Compared with the above strategies, the direct chemical modification of specific sites on canonical amino acid residues in peptides and proteins provides a more accessible approach for PTMs.^6^ Owing to the superior nucleophilicity and the relatively low abundance (<2%) in native proteins, cysteine (Cys) residue offers a unique reactive handle for facile modification of peptides and proteins at a single site.^7^ Over the past decades, significant progress has been achieved on selective functionalization of Cys residues to generate new S–C and S–X bonds through the two-electron (2e) transformations of −SH groups.^7,8^ By contrast, the site/chemical selective conversions of −SH to other C-based groups for peptides and proteins modification lagged behind due to the difficulties in forming C–C bonds through traditional 2e chemistry using the C_β_ of Cys under biocompatible conditions.^6^ Indeed, C–C bonds are highly abundant in most biological molecules and are present in the side chains of all amino acids.^9^ In order to bridge this gap between synthetic chemistry and chemical biology, the conversion of Cys residues to dehydroalanine (Dha) has been explored, thereby using Michael addition and Giese reaction to construct the C–C bonds required for PTMs.^10–14^ Although this strategy has wide applicability and strong efficacy, it results in the loss of natural stereochemistry at the modified site, yielding a mixture of diastereomers (Figure 1a, left).

**Figure 1:**
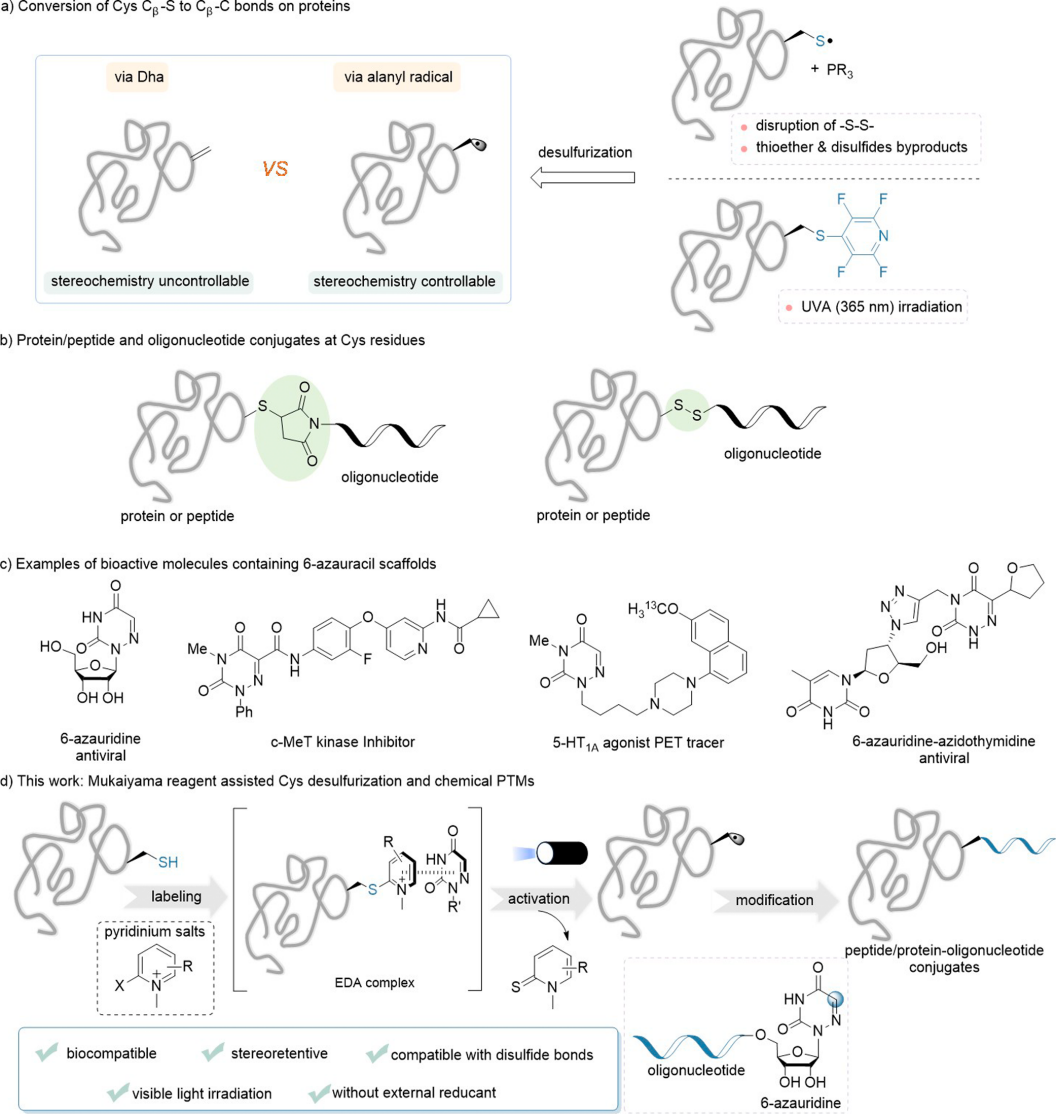
Site-specific modification of the peptide and protein based on cysteine. (a) Conversion of Cys C_β_-S to C_β_-C bonds on proteins; (b) protein/peptide and oligonucleotide conjugates at Cys residues; (c) examples of bioactive molecules containing 6-azauracil scaffolds; (d) this work: Mukaiyama reagent assisted Cys desulfurization and chemical PTMs.

To address stereochemical erosion in Dha-based protein modifications, phosphine-mediated photodesulfurization was developed to generate configurationally stable alanyl (Ala) radicals from Cys residues, enabling stereoretentive C–C/C–X bond formation in peptides/proteins (Figure 1a, right).^15–18^ However, this approach requires stoichiometric phosphine reductants that could disrupt disulfide bonds or generate thiyl radical intermediates leading to thioether/disulfide byproducts, limiting its widespread utility in PTMs. Recently, Davis reported a UVA (365 nm)-mediated desulfurization to convert Cys residues into Ala radicals using tetrafluoropyridyl as an activating group.^19^ While UVA light (365 nm) possesses higher energy than visible light and may excite endogenous chromophores (e.g., nucleobases) in biological systems, leading to detrimental side reactions,^20^ visible-light-driven (∼400–700 nm) chemical PTMs are inherently more biocompatible and preferable. However, the authors noted that metal-based photocatalysts (e.g., Ir and Ru complexes), which exhibit catalytic activity in the visible-light range, could induce substantial oxidative damage to proteins, thereby constraining their use in PTMs.^14,19^ Thus, a visible-light-driven platform enabling disulfide-compatible Cys C_β_–S cleavage without deleterious side reactions remains an unresolved challenge.

Nucleic acids exhibit substantial therapeutic promise, as demonstrated by the recent approval of several oligonucleotide-based therapeutics. However, their inherent physicochemical characteristics pose challenges in achieving an efficient delivery to target sites. To address this, conjugation approaches employing peptides or proteins have emerged as effective strategies to improve the delivery efficiency. In particular, antibody–oligonucleotide conjugates (AOCs) have attracted significant interest due to their ability to combine the tissue-specific delivery capacity of antibodies with the high specificity of oligonucleotides. Compared to noncovalent conjugation strategies (e.g., ionic interactions or affinity binders), direct covalent conjugation method of AOCs offers advantages including smaller linker sizes and minimal perturbation of the biological function of the conjugates.^21^ A widely adopted approach involves site-selective modification of cysteine residues, which are ideal handles due to their strong nucleophilicity and low natural abundance. Nevertheless, current chemical modification approaches exhibit critical limitations: maleimide-derived thioether linkages are prone to retro-Michael elimination and conjugate degradation, while disulfide bond linkages demonstrate reversible behavior in biological redox environments (Figure 1b).^22^ Thus, stable and biocompatible strategies for chemical PTMs with nucleic acids, especially site-selective target Cys residues, are desired.

Modifying nucleobases is another strategy to enhance the drug-like properties of nucleic acids. For example, the ribonucleosides of 6-azauracil, a uracil analogue, have demonstrated diverse biological activities, including antiviral, antitumor, and antifungal effects (Figure 1c).^23,24^ Moreover, C5-modified pyrimidines have been found to significantly increase their binding affinity for complementary RNA sequences, thereby enhancing therapeutic efficacy.^25^ We hypothesized that conjugating peptides and proteins to nucleic acids at the C5 position of 6-azauracil could be valuable for biological studies and potentially beneficial for optimizing complementary base pairing with target mRNA sequences by fine-tuning the peptide sequence and protein structure. Continuing with our interest in photoinduced peptide late-stage modifications,^26–28^ we here report a novel site-specific conjugation of peptide and protein with nucleic acids at C5 position of 6-azauracil through a photopromoted stereoretentive desulfuration of Cys residues. Importantly, these reactions can proceed under visible light (400 nm) irradiation without the need for external reductants, thus avoiding potential side reactions of nucleobases induced by UVA.^20^ It is noteworthy that using our strategy precise chemical modification of peptides with various radical acceptors can also be carried out under visible light (420–430 nm) with biocompatible NADH serving as the reductant.

## Results and Discussion

### 6-Azauracil Modification of Peptide via Cys Desulfurization

*N*-Alkyl-2-halopyridinium salts, particularly *N*-methyl-2-chloropyridinium iodide (Mukaiyama reagent), have been widely utilized as highly reactive condensation reagents since their discovery.^29^ Due to their excellent electrophilic properties, these salts enable highly chemoselective heteroarylation of Cys residues in peptides and proteins under biologically compatible conditions, as demonstrated by Wang^30^ and Li.^31^ In our studies, the combination of the Cys-Mukaiyama reagent adduct and 6-azauridine produced a distinct yellow color, accompanied by a significant bathochromic shift in the UV–vis spectrum. These observations suggest the formation of an electron donor–acceptor (EDA) complex. Upon irradiation with 400 nm visible light, cleavage of the C–S bond in the adduct occurred, generating an Ala radical that retained the *L*-configuration and releasing 1-methylpyridine-2­(1H)-thione (see Figures S48–S53 for details). This finding allows us to furnish our desired visible light promoted chemical protein PTMs with high diastereoselectivity. We envision that the Ala radical could undergo a radical addition reaction with 6-azauridine to form a stable C–C bond and facilitate a biocompatible conjugation of peptides and proteins with oligonucleotides (Figure 1d).

Initially, we used the model substrates dipeptide **1a** (BzNH-Ala-Cys-COOH) and 6-azauridine **2a** with a pyridinium salt as the activator. Since the S_N_Ar reaction between Cys residues and the Mukaiyama reagent is highly selective in proteins and completes very quickly,^30,31^ a slight excess of **VII** (1.1 equiv) was employed. Right after mixing **1a**, **2a**, and **VII**, the reaction mixture was exposed to light irradiation (Table 1, see Tables S1–S4 for the details). To our delight, under 400 nm visible light irradiation, and using PBS buffer (pH 8.0) with 9% CH_3_CN as the cosolvent, the reaction successfully produced the desired peptide and 6-azauridine C-5 position conjugate product **3aa** in 90% HPLC yield and 75% isolated yield after 10 h (entry 1). In 95% Tris-HCl buffer (pH 8.0), **3aa** was obtained in a 78% HPLC yield (entry 2). Other feasible pyridinium salts resulted in substantially lower yields compared with **VII** (entry 3). Shortening the reaction time to 5 h lowered the yield to 77% (entry 4). Control experiments demonstrated that both light irradiation and activation by pyridinium salts were crucial to the reaction (entries 5–6). Carrying out the reaction in air led to diminished yields (entry 7). Addition of 2,6-lutidine can slightly increase the reaction yield (entry 8). The reaction was inhibited when TEMPO (2,2,6,6 - tetramethylpiperidine-1-oxyl, 4 equiv) was present, suggesting a radical pathway might be involved (entry 9). The remarkable chemoselectivity of the reaction was validated through competitive experiments that involved other amino acids (Lys, Tyr, His, Trp, Ser, Arg, Glu) with nucleophilic side chains (entry 10). Notably, we observed S_N_Ar of pyridinium salt with Tyr or His, forming Tyr-pyridinium or His-pyridinium adducts, respectively. These adducts showed no response to photoirradiation but could be regenerated to native Tyr and His through GSH exchange. This selective photosensitive property ensures exclusive Cys activation for subsequent PTMs chemistry. Under standard reaction conditions, the reactivity of **1a** with canonical nucleosides (T, U, C, G, and A) was also investigated (entry 11). While T, U, and C remained completely inert, MS analysis detected trace amounts of Cys desulfurative coupling products with G and A. In summary, our strategy demonstrates exceptional site- and chemoselectivity toward Cys and 6-azauridine compared to other amino acids and nucleosides, which enables a precise conjugation of proteins and oligonucleotides.

**Table 1:**
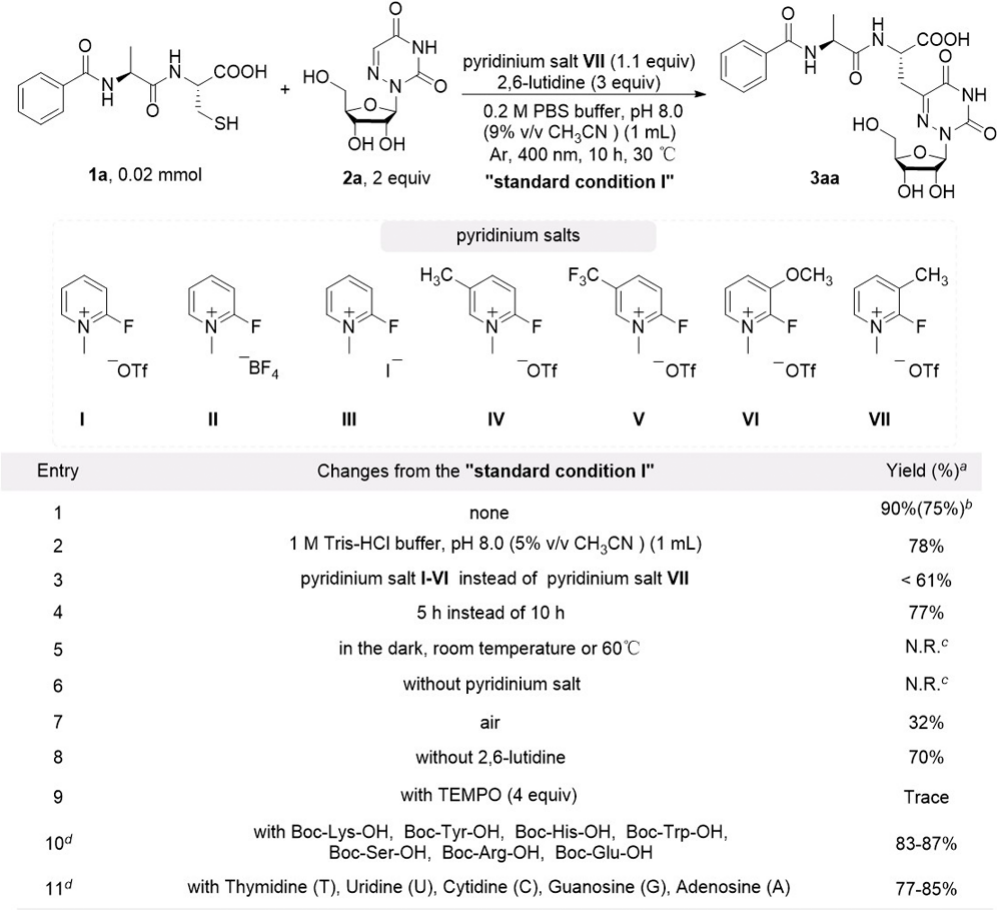
Initial Investigation of the Model Reaction

a Yields were determined by integrated areas of HPLC peaks (at 220 nm) with coumarin as an internal standard.

b Isolated yield in parentheses.

c N.R. = No Reaction.

d 2 equiv of amino acids or nucleosides used.

With optimized conditions established, we examined the reaction’s generality using peptides containing multiple reactive side chains (Figure 2). Initially, the desulfurization of GSH proceeded smoothly, resulting in its coupling with 6-azauridine at the C-5 position (**3ba**). As previously mentioned, although Tyr and His residues reacted with pyridinium salt **VII**, they could be quantitatively regenerated by GSH upon completion of the photoreaction. This ensured the formation of exclusive Cys desulfurization products in good yields (**3ca**, **3da**, **3ea**, **3ia**, and **3ja**). Notably, for peptide **1e**, which contains various nucleophilic residues, the 6-azauridine-modified product resulting from Cys desulfurization was solely monitored by LC-MS (**3ea**). The peptide containing two Cys residues underwent double desulfurative nucleotide modification to yield the dimodified product **3fa-I** when **2a** (4 equiv), **VII** (2.2 equiv), and 2,6-lutidine (6 equiv) were used. However, under standard condition I [**2a** (2 equiv), **VII** (1.1 equiv), and 2,6-lutidine (3 equiv)], the reaction yielded both mono- and dimodified products: the dimodified product **3fa-I** was obtained in 10% yield, monomodified products included **3fa-II** and **3fa-III** gave 34% combined yield, and the S_N_Ar products (**3fa-IV** and **3fa-V**) formed in 11% combined yield (see Figure S11b for the details). Several biologically active peptides (9–15 residues) were successfully modified, with HPLC analysis confirming consistently high yields (**3ga**–**3ka**, 63–94%). MS/MS analysis of purified product **3ga** confirmed exclusive modification at the Cys residue, demonstrating the site-specific activation capability of *N*-alkyl-2-halopyridinium salts for 6-azauridine conjugation (see Figure S13 for the details). As anticipated, the antibacterial peptide **1l**, comprising 33 amino acid residues, underwent successful conjugation with the nucleoside, yielding the product **3la** with a good conversion rate. In addition to linear peptides, this method is also applicable to Cys residues in cyclic peptides (**3ma** and **3na**). Notably, the Cys residue in cyclic peptide **1o**, which bears a ^2^Cys-^7^Cys disulfide bond, was successfully coupled with 6-azauridine without disrupting the disulfide bond (**3oa**). This result demonstrates the potential of our method for modifying disulfide-containing peptides and proteins, which cannot be achieved using the P­(III)-mediated desulfuration of Cys residues.^15–18^

**Figure 2:**
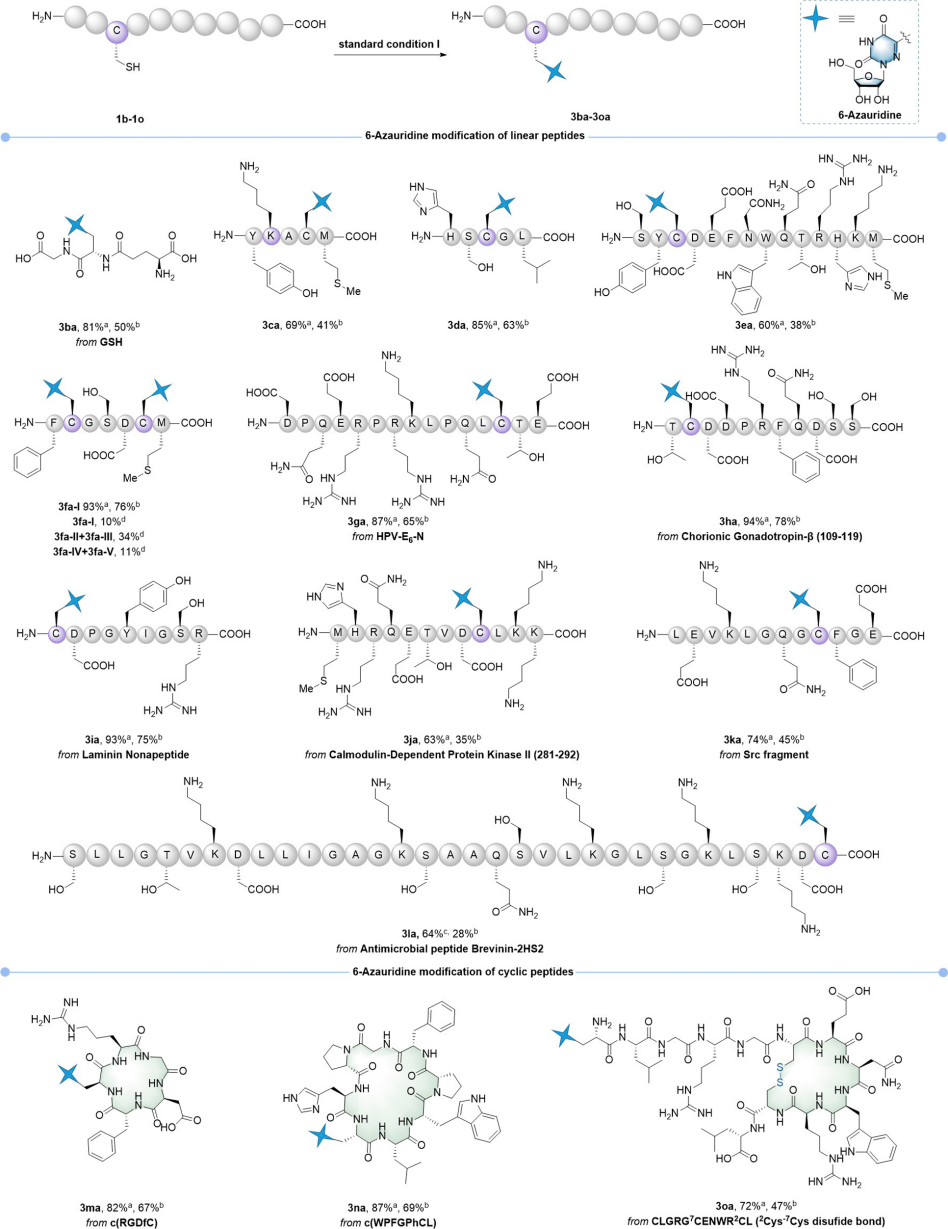
Desulfurizative coupling of the Cys residue with 6-azauridine in peptides. Reaction conditions: peptide (5 μmol), **2a** (2 equiv), **VII** (1.1 equiv), and 2,6-lutidine (3 equiv) in 250 μL PBS buffer (0.2 M, pH 8.0, 9% v/v CH_3_CN), visible light (100 W, 400 nm), Ar atmosphere, 10 h, 30 °C. For **3ba** and **3ma**, isolated yields on 60 μmol scale. For **3fa**, **2a** (4 equiv), **VII** (2.2 equiv), and 2,6-lutidine (6 equiv) were used. After the peptides containing Tyr and His (**3ca**–**3ea**, **3ia**, **3ja**, and **3na**) were prepared, GSH (4 equiv) was added and stirred for 1 h to regenerate Tyr and His. ^*a*^HPLC yields were based on analysis of the reaction mixture by integrating UV absorptions of peptide-related peaks at 220 nm. ^*b*^Isolated yield by semipreparative HPLC. ^*c*^Conversion rate was estimated using total ion count (TIC), and **1l** (2 μmol) was used. ^*d*^ **2a** (2 equiv), **VII** (1.2 equiv), and 2,6-lutidine (3 equiv) were used.

The integration of 6-azauridine into oligoribonucleotides has proven to be an invaluable tool for studying nucleic acid folding and ribozyme activity.^32^ Having established the protocol for coupling peptides with 6-azauridine, we extended our investigation to explore peptide conjugation with 6-azauridine-containing nucleotides (Figure 3). Dinucleotides comprising 6-azauridine smoothly underwent reactions with the RGD peptide, yielding the corresponding products **3mb**–**3mf** with consistently high efficiencies (>91% yield) without affecting other nucleobases (U, T, C, A, G). Encouragingly, peptide–oligonucleotide conjugate **3mg** was also obtained with a satisfactory yield. Furthermore, we successfully extended this methodology to various nucleoside derivatives, including *N*-methyl-6-azauracil riboside (**2h**), 6-azauracil deoxyriboside (**2i**), and 6-azauracil glucoside (**2j**). All of these substrates reacted cleanly with RGD peptide to afford the desired products (**3mh**–**3mj**) in moderate to good yields (58–78%). Significantly, 6-azauracil derivatives bearing various functional groups, including alkenyl (**3mk**), alkynyl (**3 mL**), and PEG2-linked azido (**3 mm**) moieties, all demonstrated excellent compatibility. Moreover, drug compounds, such as biotin (**3mn**), ibuprofen (**3mo**), and diclazuril (**3mp**), contained 6-azauracil derivatives also effectively conjugated to the RGD peptide (55–86% yields).

**Figure 3:**
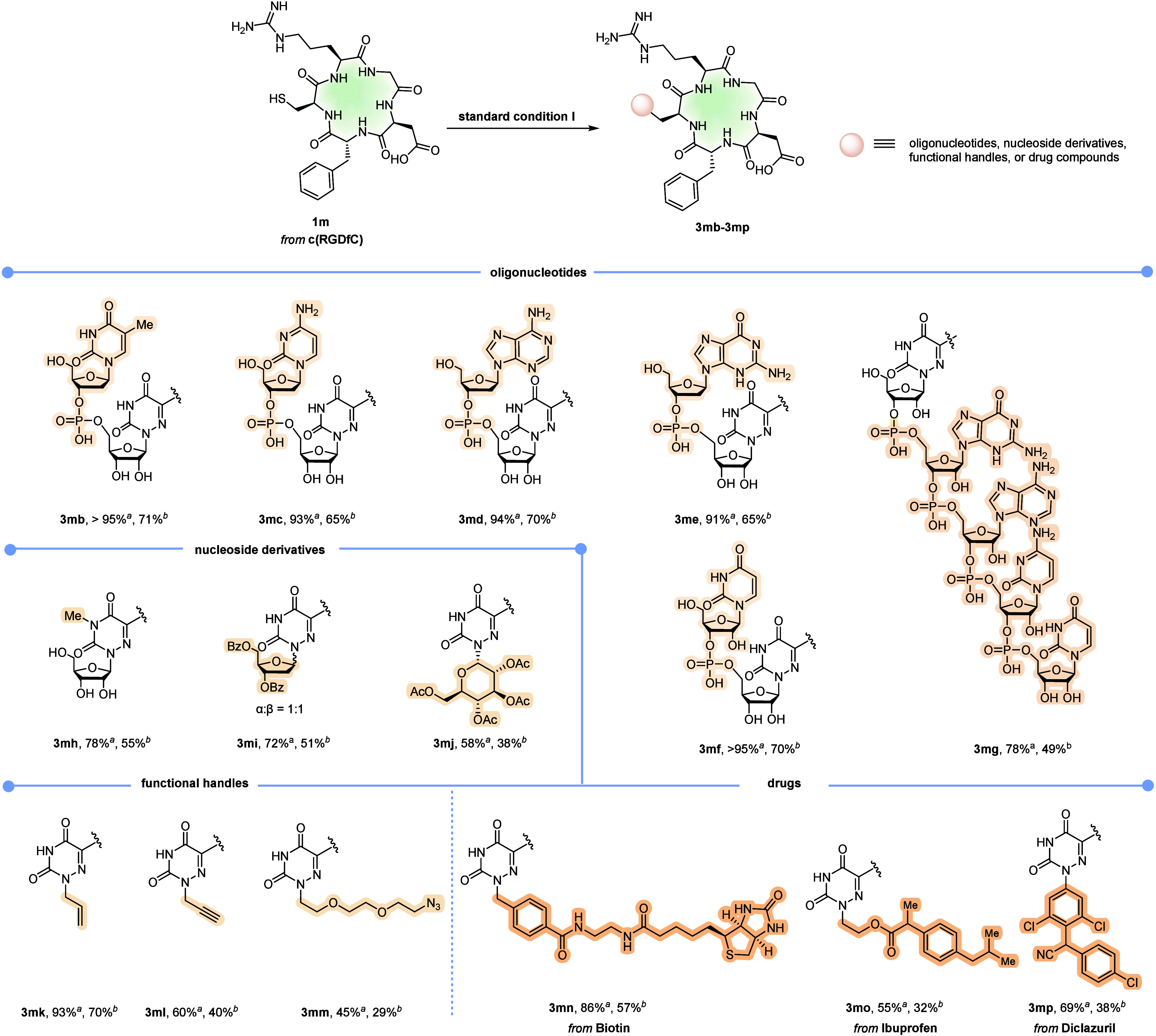
Modifications of peptides with 6-azauracil-containing nucleotides and derivatives. Reaction conditions: **1m** (5 μmol), **2b**–**2p** (2 equiv), **VII** (1.1 equiv), and 2,6-lutidine (3 equiv) in 250 μL PBS buffer (0.2 M, pH 8.0, 9% v/v CH_3_CN), visible light (100 W, 400 nm), Ar atmosphere, 10 h, 30 °C. For **3mg**, **2g** (1 equiv) was used. For **3mi**, **3mj**, **3mm**, **3mo**, and **3mp**, 10% DMF was added to help dissolve. ^*a*^HPLC yields based on analysis of the reaction mixture by integrating UV absorptions of peptide-related peaks at 220 nm. ^*b*^Isolated yield by semipreparative HPLC.

### Desulfurizative Modification of Peptide Cys Residues with Various Radical Acceptors

The formation of Ala radicals through Cys desulfurization has been utilized for site-specific chemical modifications in peptides and proteins.^15–19^ To further expand the applicability of our strategy, we investigated its reactivity with a series of radical acceptors. Initially, we selected GSH peptide **1b** and phenyl vinyl sulfone **4a** as model substrates (Table 2, see Tables S5–S8 for the details). Under visible light irradiation (400 nm) for 3 h using Hantzsch ester as the reductant, the reaction achieved a 90% HPLC yield of the target product **5ba** (entry 1). Notably, when NADH replaced Hantzsch ester as the endogenous reductant, the reaction proceeded effectively under 420–430 nm light, delivering the product in 86% yield after 5 h (entry 2). Time-course analysis revealed a rapid reaction with yields reaching 82% and 74% within 1.5 h, respectively (entries 3 and 4). Control experiments confirmed the essential roles of light, pyridinium salt, and reductant in the reaction (entries 5–7). Air exposure diminished yields (entry 8). The addition of 2,6-lutidine had a positive impact on promoting the reaction (entry 9). Notably, TEMPO introduction suppressed **5ba** formation, with only trace amounts detected while the GSH-TEMPO adduct was observed (entry 10), strongly suggesting the involvement of a radical-mediated mechanism.

**Table 2:**
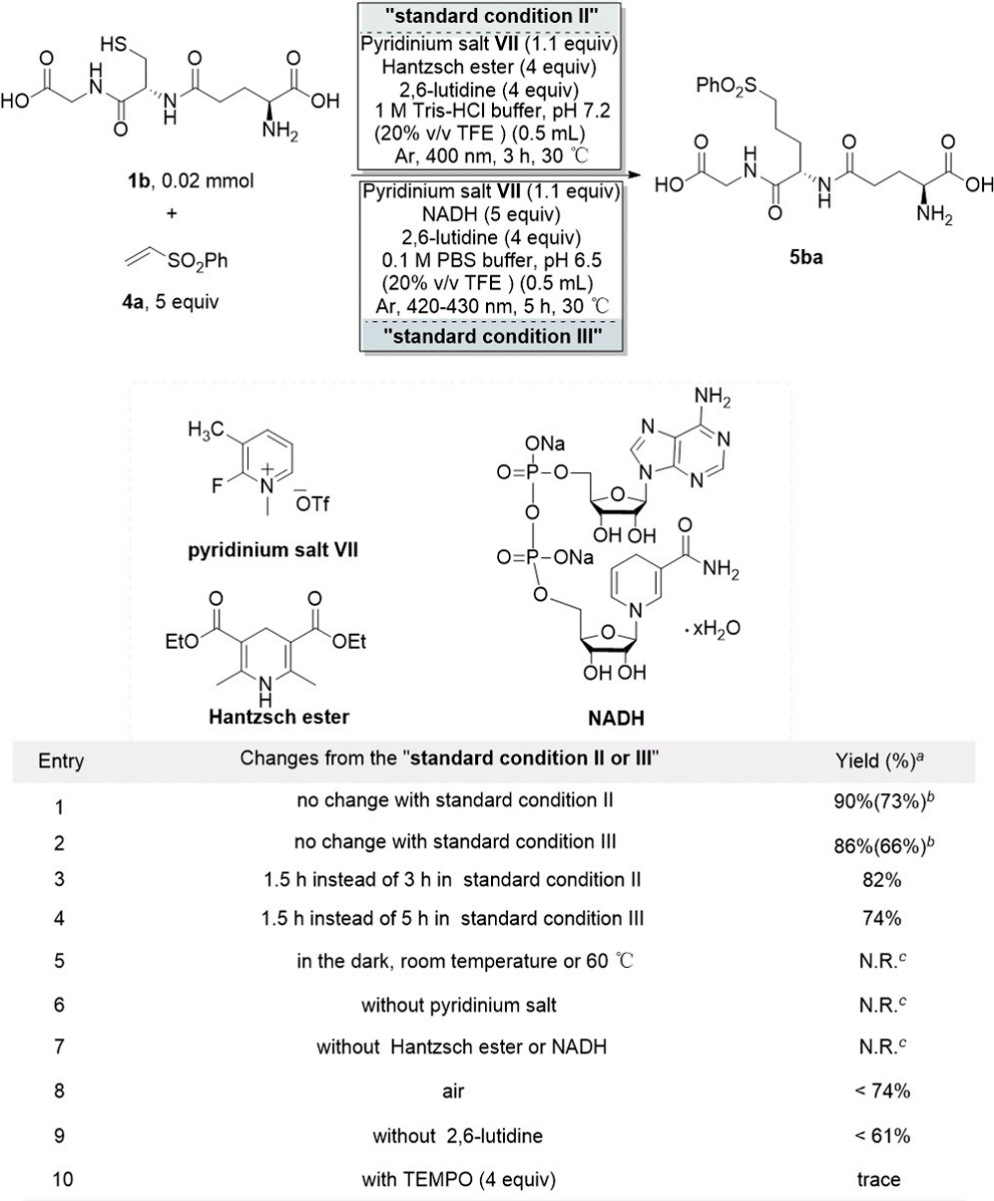
Optimized Reaction Conditions of Ala Radical Trapping with Radical Acceptors

a Yields were determined by integrated areas of HPLC peaks (at 220 nm) with coumarin as an internal standard.

b Isolated yield in parentheses by semipreparative HPLC.

c N.R. = No Reaction.

To validate the versatility of our method, we extended its application to construct novel C–C bonds using other Michael acceptors (vinyl phosphonate **4b** and 4-vinylpyridine **4c**). As anticipated, the desulfurizative alkylation products were obtained in high yields (Scheme 1, **5bb** and **5bc**). Significantly, the reaction with phenyl allylsulfone efficiently converted Cys residues to homoallylglycine (Hag) residues (**5bd**), which is a powerful handle for thiol–ene click reactions and metathesis-based peptide stapling. Moreover, diselenide could also participate in the reaction, leading to the in situ formation of a phenyl selenocysteine residue (**5be**). The TEMPO trapping reaction constructed a new C–O bond (**5bf**), which could facilitate the incorporation of diverse TEMPO-functionalized groups into peptides.^15^ Remarkably, our strategy enabled the site-selective conversion of Cys to the Lys residue, achieving precise late-stage editing of the peptide backbone (**5bg**). The protocol was successfully applied to the bioactive peptide **1h**, delivering modified products **5ha**, **5hb**, and **5he** in good yields. In the absence of radical traps, the Ala radical intermediate could be reduced by the Hantzsch ester or NADH to yield Ala residues (**5hh**). The combination of visible light irradiation (400 or 420–430 nm) with biocompatible reductants (Hantzsch ester or NADH) under mild conditions enables this method as a powerful tool for precision peptide modification.

**Scheme 1:**
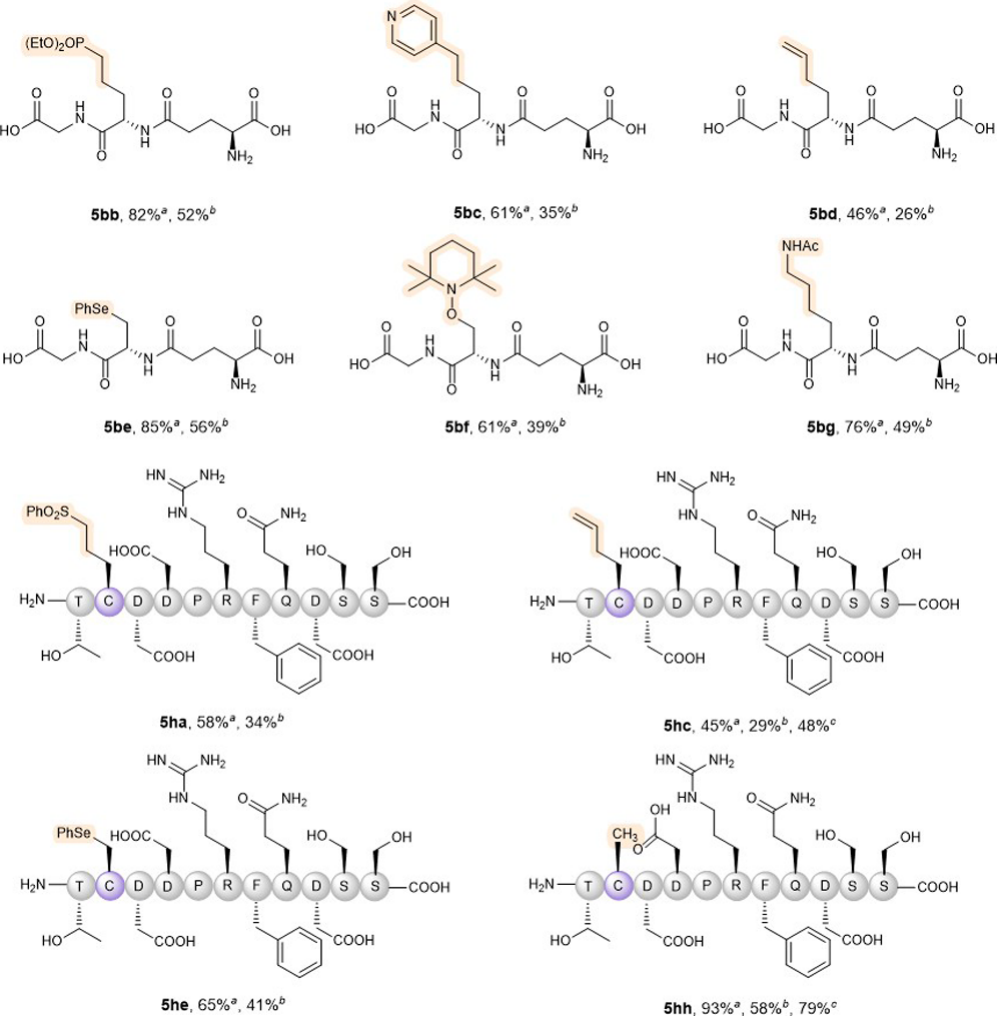
Scope of *L*-Alanyl Radical-Trapping from Peptide

a HPLC yields under condition II based on HPLC analysis of the reaction mixture by integrating UV absorptions of peptide-related peaks at 220 nm.
b Isolated yield by semipreparative HPLC.
c HPLC yields under condition III based on HPLC analysis of the reaction mixture by integrating UV absorptions of peptide-related peaks at 220 nm.

### Modification of Cys-Contained Protein

Building on our success with precise peptide modification, we extended this strategy to complex protein functionalization. Utilizing bovine serum albumin (BSA), the most abundant serum carrier protein featuring 17 disulfide bonds and a single-solvent-exposed cysteine residue (Cys34), we activated Cys34 using pyridinium salt **VII**. Subsequent 400 nm irradiation triggered desulfurization, followed by the site-selective conjugation of nucleoside **2a** and dinucleotide **2b** specifically at Cys34 (Figure 4a,b), achieving 80% and 93% conversion, respectively, while maintaining all native disulfide bonds, as confirmed by UPLC-HRMS analysis. Remarkably, we installed a bioorthogonal alkyne handle onto BSA through treatment with **VII** (1.25 equiv) and **2l** (100 equiv) in Tris-HCl buffer (0.1 M, pH 8.0) under ambient 400 nm irradiation. This allowed efficient fluorescent labeling via CuAAC click chemistry with FAM dye, as confirmed by SDS-PAGE (Figure 4d). Collectively, this strategic approach not only facilitates the conjugation of proteins with nucleotides but also enables the installation of functional handles and the labeling of proteins with fluorescent tags, thereby offering a practical platform for protein bioconjugation under biologically compatible conditions.

**Figure 4:**
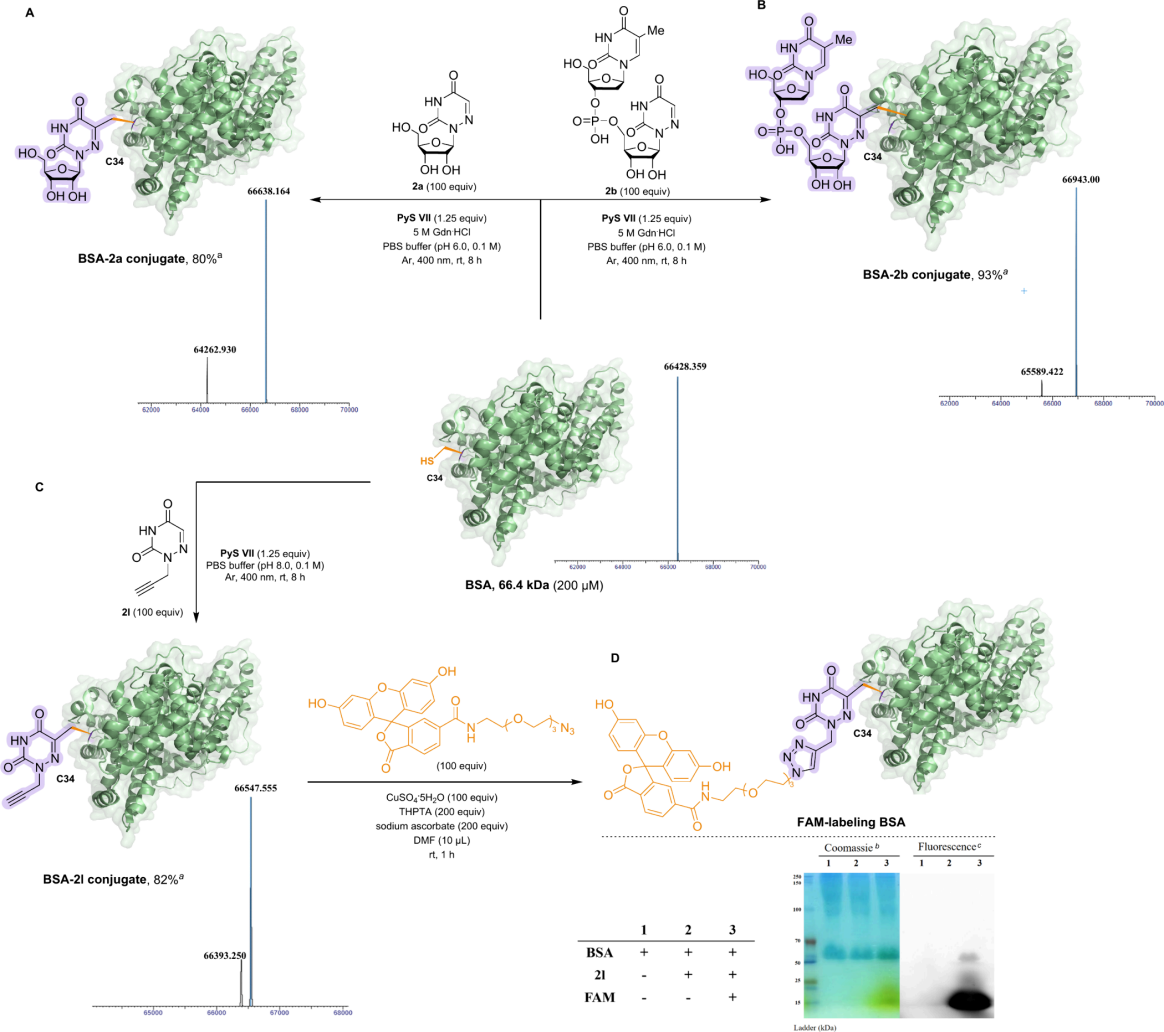
Modifications of Cys-contained protein. (A) Conjugation of nucleoside **2a** and BSA. (B) Conjugation of dinucleotide **2b** and BSA. (C) Installation of the alkyne handle onto BSA. (D) Labeling of BSA with fluorescent FAM. ^*a*^Conversion rate was estimated using mass intensity of the protein-related peak based on the deconvoluted spectrum. ^*b*^Coomassie blue staining. ^*c*^Irradiation was carried out with a 460 nm wavelength laser.

In summary, we developed a novel strategy for site-selective, stereoretentive modification of peptides and proteins via pyridinium salt-directed Cys residue desulfurization under visible light activation. Critically, the generated Ala radicals retain the native *L*-configuration at the stereogenic C_α_ center during post-translational editing. This strategy enables conjugation of diverse payloads, including nucleotides, functional groups, and therapeutic agents, to peptides by constructing new C–C bonds at position C5 of 6-azauracil. Notably, common radical scavengers could also be incorporated into Cys-containing peptides under mild reductive conditions using either Hantzsch ester or NADH. Although the complete mechanism remains under investigation, and alternative pathways cannot be ruled out, our preliminary data are consistent with a pathway initiated by an EDA complex that mediates a photoinduced SET process. This approach not only expands the toolbox for studying PTMs, but provides a versatile platform for investigating structure–function relationships across peptides, proteins, and nucleic acids under physiologically compatible conditions.

## Supplementary Material



## Notes

Experimental details, analytical data for all new compounds, and NMR spectra (PDF)
