# Supplementary material for: Visible Light Induced Mukaiyama Reagent Promoted Desulfurative Modification of Peptides and Proteins with Nucleotides
Source: ACS Cent Sci. 2025 Sep 29;11(11):2206–14. doi: 10.1021/acscentsci.5c01241 (PMC12670305; doi:10.1021/acscentsci.5c01241)
Supplement: Supplementary file 1 [file oc5c01241_si_001.pdf]

Supporting Information for Publication

Visible Light Induced Mukaiyama Reagent Promoted Desulfurative Modification of Peptides and Proteins with Nucleotides

Mengran Wang[1, *], Yongjia Lei[1], Xinyu Song[1], Chunlin Wang[2], Quanping Guo[1], Xiuren Zhou[1], Wenbo Mao[1], Kuan Chen[1], Zhaoqing Xu[1,3,4, *]

[1]*School of Basic Medical Sciences, Lanzhou University, Lanzhou 730000, China;* [2]*School of Pharmacy, Lanzhou University, Lanzhou 730000, China;* [3]*Key Laboratory of Preclinical Study for New Drugs of Gansu Province, School of Basic Medical Sciences, Lanzhou University, Lanzhou 730000, China;* [4]*Research Unit of Peptide Science, Chinese Academy of Medical Sciences*

* Correspondence to: zqxu@lzu.edu.cn
wangmr@lzu.edu.cn

Table of Contents

1. General information 1
2. Synthesis of substrates 1 and 2 1
    2.1 Synthesis of peptides 1
    2.2 Synthesis of 2b-2f 3
    2.3 Synthesis of 6-azauridine nucleoside phosphoramidite (DMT-2'O-TBS-6-Aza-rU phosphoramidite) 13
    2.4 Synthesis of 2h-2p 15
    2.5 Synthesis of FAM-PEG$_3$-N$_3$ 21
3. Optimization of reaction conditions 22
    3.1 Screen the reaction conditions for 6-azauridine modification of peptide 22
    3.2 Screen the reaction conditions of *L*-alanyl radical with radical acceptor 25
    3.3 General Procedure 4 (GP4) of condition I 29
    3.4 General Procedure 5 (GP5) of condition II 29
    3.5 General Procedure 6 (GP6) of condition III 29
    3.6 Picture of the reaction photo set-up (400 nm) 29
4. Characterization of products 31
5. Analytical HPLC data of peptides 35
6. Mechanistic studies 78
    6.1 Radical trapping experiment 78
    6.2 Chirality verification of alanyl radical 78
    6.3 Analysis of UV-vis absorption spectra 79
    6.4 Light/dark experiment 80
    6.5 Proposed mechanism 80
7. Modifications of bovine serum albumin (BSA) 82
8. References 91
9. NMR spectra of products 92

1. General information

All commercially available reagents were used without further purification unless otherwise stated. ^{1}H NMR, ^{19}F NMR, ^{31}P NMR and ^{13}C NMR spectra were recorded on a Bruker AVANCE NEO 400 or Bruker AVANCE NEO 600 instrument spectrometer. Data for ^{1}H NMR are recorded as follows: chemical shift (δ, ppm), multiplicity (s = singlet, d = doublet, t = triplet, m = multiplet, q = quartet, dd = doublet of doublets, dt = doublet of triplets, td = triplet of doublets, and brs = broad signal, and coupling constant(s) in Hz integration). Data for ^{13}C NMR, ^{19}F NMR and ^{31}P NMR are reported in terms of chemical shift (δ, ppm). All new compounds were further characterized by high resolution mass spectra (HRMS, ESI source, Bruker MaXis 4G). The photochemical reactions were conducted using Heyseekt LED. Reactions purification were carried out using Semi preparative HPLC (Hanbon Sci.& Tech). Purity analysis were performed on HPLC (UNIMICRO EasySep®-3030) using a Waters Symmetry® C18 Column (100 Å, 5 µm, 4.6 mm × 250 mm), or Globalsil® C18-AP column (120 Å, 5 µm, 4.6 mm × 250 mm). Linear gradients using A: MeCN (0.1% CF_3COOH) and B: H_2O (0.1% CF_3COOH). LC-MS was performed on a Thermo Scientific Orbitrap IQ-X Tribrid or Bruker Sciex X500R.

2. Synthesis of substrates 1 and 2

2.1 Synthesis of peptides

General procedure 1 (GP1) for solid phase peptide synthesis

All peptides were synthesized using standard Fmoc-based Solid Phase Peptide Synthesis (SPPS) chemistry with the Rink Amide MBHA resin or 2-Chlorotrityl Chloride resin as the solid support. All coupling reactions were carried out on 0.2 mmol scale unless otherwise noted and used commercially available Fmoc-protected amino acids building blocks. For peptide elongation, the resin was shaken with Fmoc-protected amino acids (3 equiv), HBTU (3 equiv), HOBt (3 equiv), DIPEA (6 equiv) in DMF. After each coupling reaction, the Fmoc group was cleaved using 1% piperidine and 1% DBU in DMF. The cleavage of peptides from resin and the globally deprotection were carried out with Reagent A (a solution of TFA/TIS/H_2O (95:2.5:2.5)) at room temperature for 2 hours. The reaction mixture was filtered, concentrated and precipitated out by the addition of cold ethyl ether, and the obtained crude product was purified by semi-HPLC (Hanbon Sci.& Tech (Nucifera C18U, 10µ, 120Å, 20 mm × 250 mm), linear gradients using A: MeCN (0.1% CF_3COOH) and B: H_2O (0.1% CF_3COOH)). The purity of all peptides was determined by HPLC analysis (UNIMICRO EasySep®-3030 using a Waters Symmetry® C18 Column (100 Å, 5 µm, 4.6 mm × 250 mm) or Globalsil® C18-AP column (120 Å, 5 µm, 4.6 mm × 250 mm)). The pure peptides were obtained as solid compounds after lyophilization. Linear peptide **1l** and cyclic peptide **1o** were custom synthesized by GL Biochem (Shanghai) Ltd.

General procedure 2 (GP2) for cyclic peptide 1m synthesis [1]

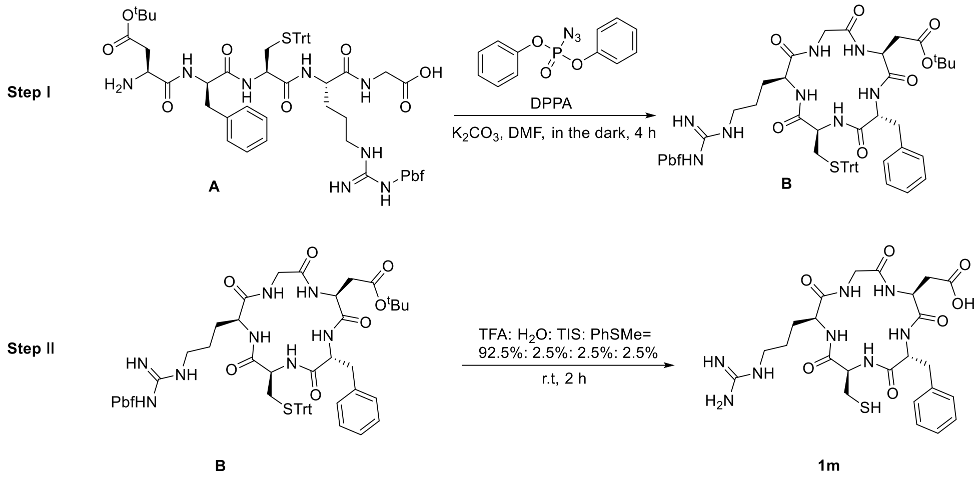

Step I: The linear peptide **A** H-Asp(O^{t}Bu)-Phe(D)-Cys(Trt)-Arg(Pbf)-Gly-OH (1 mmol) was prepared according to GP1. Linear peptide **A** was dissolved in DMF (400 mL, c = 2.5 × 10^{-3} M for very high dilution conditions), DPPA (3 equiv) and K_2CO_3 (5 equiv) were added and the mixture was stirred for 4 hours at room temperature in the dark. The DMF was evaporated under high vacuum.

Step II: The protected cyclic peptide was dissolved in a solution of TFA:H_2O:TIS:PhSMe (92.5%:2.5%:2.5%:2.5%). The reaction mixture was allowed to stir for 2 hours at room temperature. The solvent was evaporated under reduced pressure. Then the crude material was purified by the Hanbon machine (Hanbon Sci.& Tech.) equipped with the Nucifera C18U column and lyophilization to afford the desired cyclic peptide **1m**.

The spectra of the isolated products matched that of previously reported materials.[1]

2.2 Synthesis of 2b-2f [2]

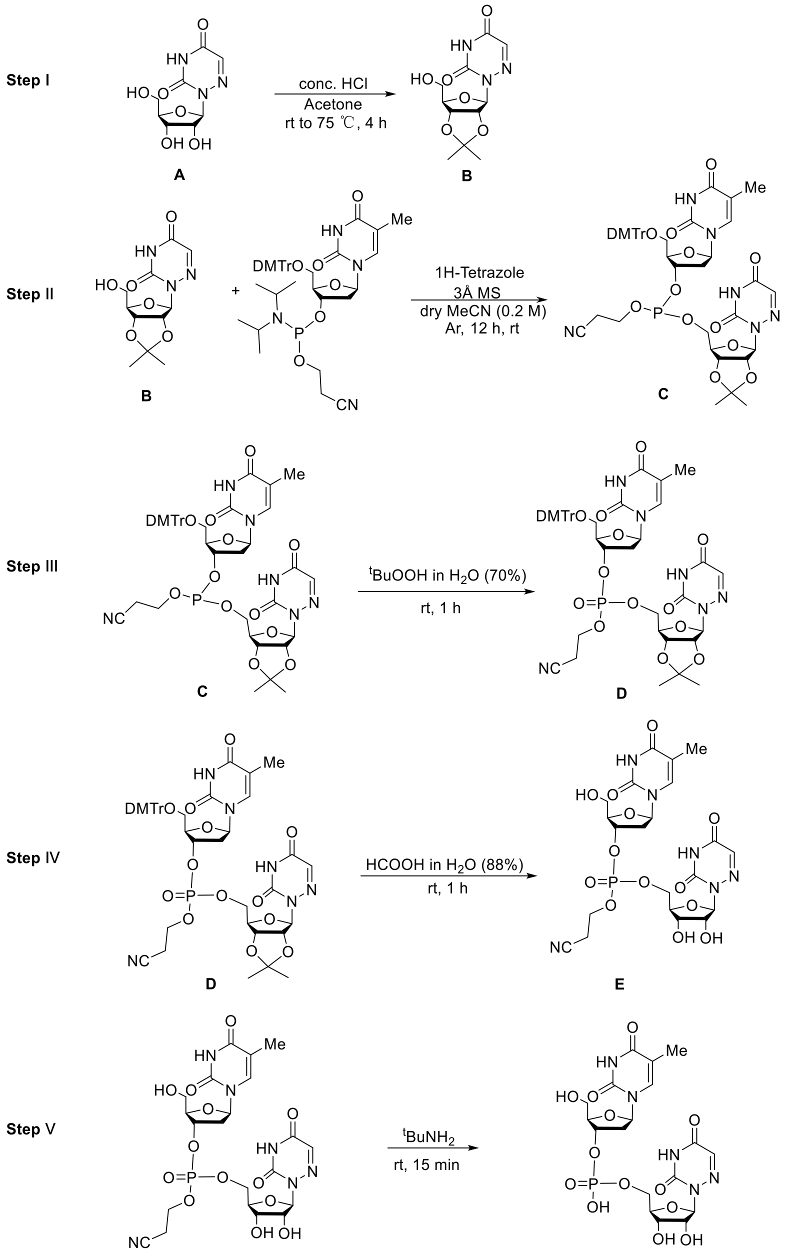

Step I: 6-azauridine **A** (2 mmol, 1.0 equiv) and acetone (5 mL) were added to the pressure-resistant tube, followed by dropwise addition of conc. HCl (40 μL). Then the mixture was stirred at 75 °C for 4 hours. After the reaction was completed, the mixture was cooled to room temperature, Et_3N (0.5 mL) was added dropwise to quench the reaction. The resulting mixture was filtered and the solids are washed with DCM (× 3). The organic layers were combined and concentrated under reduced pressure. The resulting mixture was purified through rapid silica gel column chromatography using petroleum ether/ethyl acetate as eluent.

2-((3*aR*,4*R*,6*R*,6*aR*)-6-(hydroxymethyl)-2,2-dimethyltetrahydrofuro[3,4-d][1,3]dioxol-4-yl)-1,2,4-triazine-3,5(2H,4H)-dione (product **B)** Yield: 60%. **^{1}H NMR** (600 MHz, $CDCl_3$) δ 9.17 (s, 1H), 7.49 (s, 1H), 6.26 (s, 1H), 5.05 (q, J = 3.0 Hz, 1H), 4.88 (q, J = 3.0 Hz, 1H), 4.37 (dd, J = 3.6 Hz, 2.0Hz, 1H), 3.87-3.85 (m, 1H), 3.75-3.71 (m, 1H), 2.69-2.67 (m, 1H), 1.62 (s, 3H), 1.39 (s, 3H). **^{13}C NMR** (150 MHz, $CDCl_3$) δ 156.03, 147.91, 136.42, 114.04, 92.65, 87.41, 83.24, 81.11, 63.19,

27.11, 25.34. **HRMS (ESI)** $C_{11}H_{15}N_3NaO_6$ $[M+Na]^+$ calcd: 308.0865, found: 308.0853.

Step II: A flame-dried 25 mL Schlenk tube equipped with magnetic stir bar was charged with DMT-dT phosphoramidite (0.4 mmol, 1.0 equiv), product **B** (0.4 mmol, 1.0 equiv), 1H-tetrazole (0.48 mmol, 1.2 equiv), and flame-dried 3 Å MS (powder, about 100 mg). Ar was substituted for three times. The solids were suspended in anhydrous acetonitrile (2 mL, 0.2 M). TLC could be utilized to monitor the process of the reaction. After vigorous stirring at room temperature for about 12 hours.

Step III: Then the solution of tert-butyl hydroperoxide (70% in H_2O, 5.0 equiv) was added and the reaction mixture was stirred for about an additional 1 hour. The reaction mixture was filtered through a plug of Celite and the filter cake washed with DCM. The filtrate was concentrated under reduced pressure to obtain compound **D**. Due to the presence of multiple protecting groups, the reaction intermediates could be directly used in the next step without separation and purification.

Step IV: To a 100 mL round bottom flask equipped with magnetic stir bar, charged with compound **D**, and closed tightly with rubber septum and a balloon, the formic acid (88 % in H_2O, 0.05 M) was added at room temperature under continuous stirring. TLC could be utilized to monitor the process of the reaction. The mixture was concentrated under reduced pressure to obtain compound **E**. The reaction intermediates could be directly used in the next step without separation and purification.

Step V: Then the compound **E** was dissolved in tert-butyl amine (0.05 M). After 15 minutes, the mixture was concentrated again. Then the crude material was purified by the semi-HPLC (Hanbon Sci.& Tech.) equipped with the Nucifera C18U column to afford the desired product **2b**. Linear gradients using A: MeCN and B: H_2O (0.5% NH_4OAc).

((2*R*,3*S*,4*R*,5*R*)-5-(3,5-dioxo-4,5-dihydro-1,2,4-triazin-2(3H)-yl)-3,4-dihydroxytetrahydrofuran-2-yl)methyl((2*R*,3*S*,5*R*)-2-(hydroxymethyl)-5-(5-methyl-2,4-dioxo-3,4-dihydropyrimidin-1(2H)-yl)tetrahydrofuran-3-yl) hydrogen phosphate (2b) Yield: 66%. **^{1}H NMR** (400 MHz, D_2O) δ 7.47 (s, 1H), 7.32 (s, 1H), 6.09 (dd, J = 6.0 Hz, 1.8 Hz, 1H), 5.96 (d, J = 4.0 Hz, 1H), 4.59-4.56 (m, 1H), 4.43-4.41 (m, 1H), 4.30 (t, J = 5.6 Hz, 1H), 4.05-4.00 (m, 2H), 3.96-3.91 (m, 1H), 3.85-3.80 (m, 1H), 3.62-3.54 (m, 2H), 2.38-2.32 (m, 1H), 2.16-2.09 (m, 1H), 1.73 (s, 3H). **^{13}C NMR** (101 MHz, D_2O) δ 181.38, 170.97, 166.42, 164.13, 151.56, 137.35, 136.55, 89.99, 85.54, 85.02, 82.20 (d, J = 9.0 Hz), 75.51 (d, J = 5.0 Hz), 72.58, 70.04, 65.48 (d, J = 5.3 Hz), 61.19, 51.87, 38.68. **^{31}P NMR** (162 MHz, D_2O) δ -0.92. **HRMS (ESI)** $C_{18}H_{24}N_5NaO_{13}P$ $[M+Na]^+$ calcd: 572.1003, found: 572.1000.

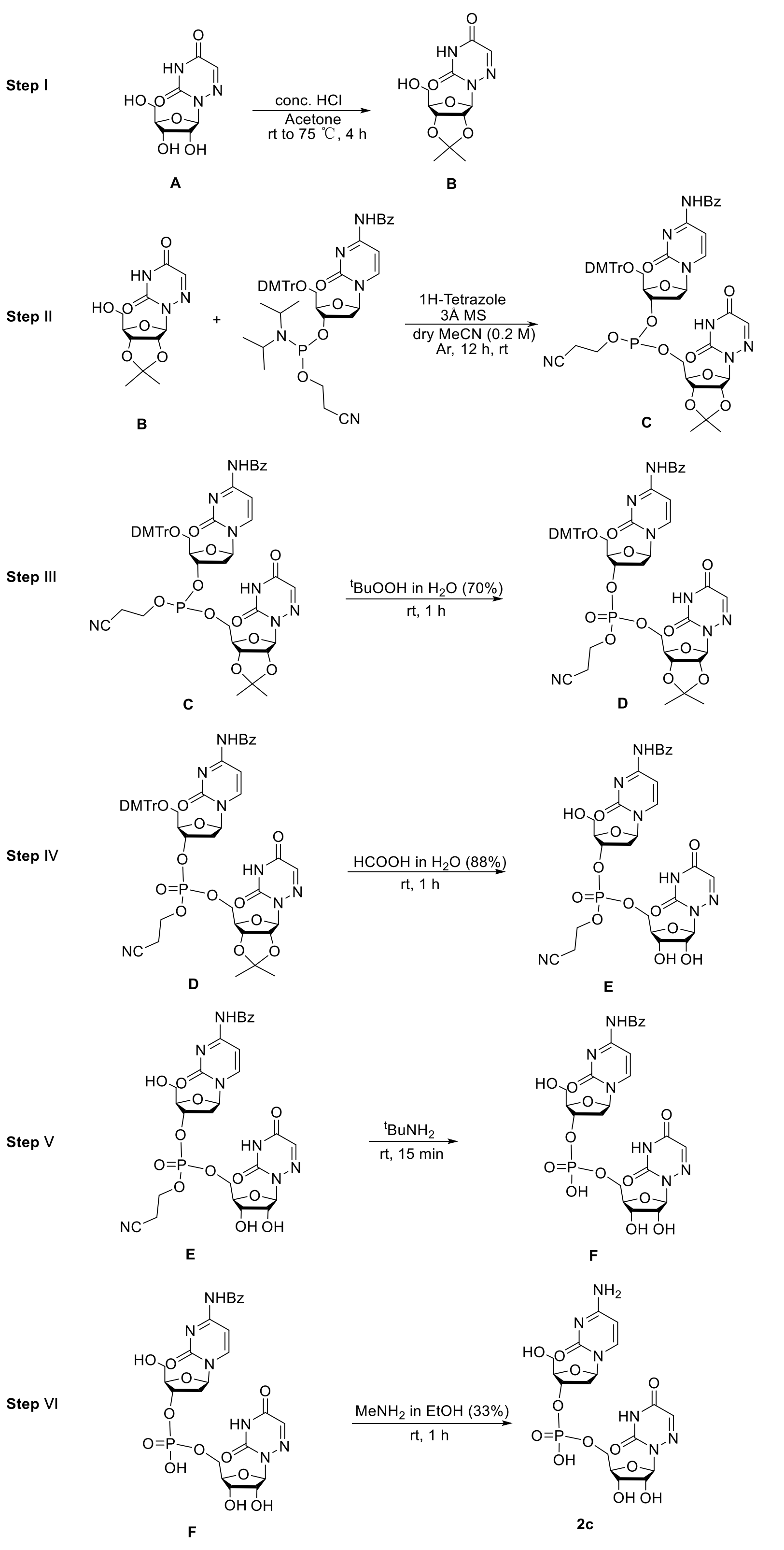

Step I: 6-azauridine **A** (2 mmol, 1.0 equiv) and acetone (5 mL) were added to the pressure-resistant tube, followed by dropwise addition of conc. HCl (40 μL). Then the mixture was stirred at 75 ℃ for 4 hours. After the reaction was completed, the mixture was cooled to room temperature, Et_3N (0.5 mL) was added dropwise to quench the reaction. The resulting mixture was filtered and the solids are washed with DCM (× 3). The organic layers were combined and concentrated under

reduced pressure. The resulting mixture was purified through silica gel column chromatography using petroleum ether/ethyl acetate as eluent.

Step II: A flame-dried 25 mL Schlenk tube equipped with magnetic stir bar was charged with DMT-dC(bz) phosphoramidite (0.4 mmol, 1.0 equiv), product **B** (0.4 mmol, 1.0 equiv), 1H-tetrazole (0.48 mmol, 1.2 equiv), and flame-dried 3 Å MS (powder, about 100 mg). Ar was substituted for three times. The solids were suspended in anhydrous acetonitrile (2 mL, 0.2 M). TLC could be utilized to monitor the process of the reaction. After vigorous stirring at room temperature for about 12 hours.

Step III: Then the solution of tert-butyl hydroperoxide (70% in H_2O, 5.0 equiv) was added and the reaction mixture was stirred for about an additional 1 hour. The reaction mixture was filtered through a plug of Celite and the filter cake washed with DCM. The filtrate was concentrated under reduced pressure to obtain compound **D**. Due to the presence of multiple protecting groups, the reaction intermediates could be directly used in the next step without separation and purification.

Step IV: To a 100 mL round bottom flask equipped with magnetic stir bar, charged with compound **D**, and closed tightly with rubber septum and a balloon, the formic acid (88% in H_2O, 0.05 M) was added at room temperature under continuous stirring. TLC could be utilized to monitor the process of the reaction. The mixture was concentrated under reduced pressure to obtain compound **E**. The reaction intermediates could be directly used in the next step without separation and purification.

Step V: Then the compound **E** was dissolved in a tert-butyl amine (0.05 M). After 15 minutes, the mixture was concentrated again.

Step VI: The 12 mL the methylamine (solution in methanol, 0.1 M) was added and stirred for about 2 hours. Then the crude material was concentrated and purified by the semi-HPLC (Hanbon Sci.& Tech.) equipped with the Nucifera C18U column to afford the desired product **2c**. Linear gradients using A: MeCN and B: H_2O (0.5% NH_4OAc).

(2*R*,3*S*,5*R*)-5-(4-amino-2-oxopyrimidin-1(2H)-yl)-2-(hydroxymethyl)tetrahydrofuran-3-yl (((2*R*,3*S*,4*R*,5*R*)-5-(3,5-dioxo-4,5-dihydro-1,2,4-triazin-2(3H)-yl)-3,4-dihydroxytetrahydrofuran-2-yl)methyl) hydrogen phosphate (2c) Yield: 67%. **^{1}H NMR** (400 MHz, D_2O) δ 7.62 (d, *J* = 8.0 Hz, 1H), 7.31 (s, 1H), 6.08 (dd, *J* = 6.4 Hz, 1.3 Hz, 1H), 5.96 (d, *J* = 3.5 Hz, 1H), 5.90-5.85 (m, 1H), 4.56-4.53 (m, 1H), 4.42 (t, *J* = 4.0 Hz, 1H), 4.29 (t, *J* = 4.0 Hz, 1H), 4.06-4.02 (m, 1H), 3.96-3.91 (m, 1H), 3.85-3.80 (m, 1H), 3.72-3.46 (m, 3H), 2.41-2.36 (m, 1H), 2.10-2.03 (m, 1H). **^{13}C NMR** (101 MHz, D_2O) δ 181.38, 166.07, 157.41, 149.91, 141.58, 141.49, 136.56, 96.38, 90.03, 86.12, 85.64 (d, *J* = 5.0 Hz), 82.24 (d, *J* = 9.0 Hz), 75.50 (d, *J* = 6.0 Hz), 72.57, 70.09, 61.24, 38.27 (d, *J* = 4.0 Hz). **^{31}P NMR** (162 MHz, D_2O) δ -0.90. **HRMS (ESI)** $C_{17}H_{22}N_6O_{12}P$ $[M-H]^-$ calcd: 533.1032, found: 533.1039.

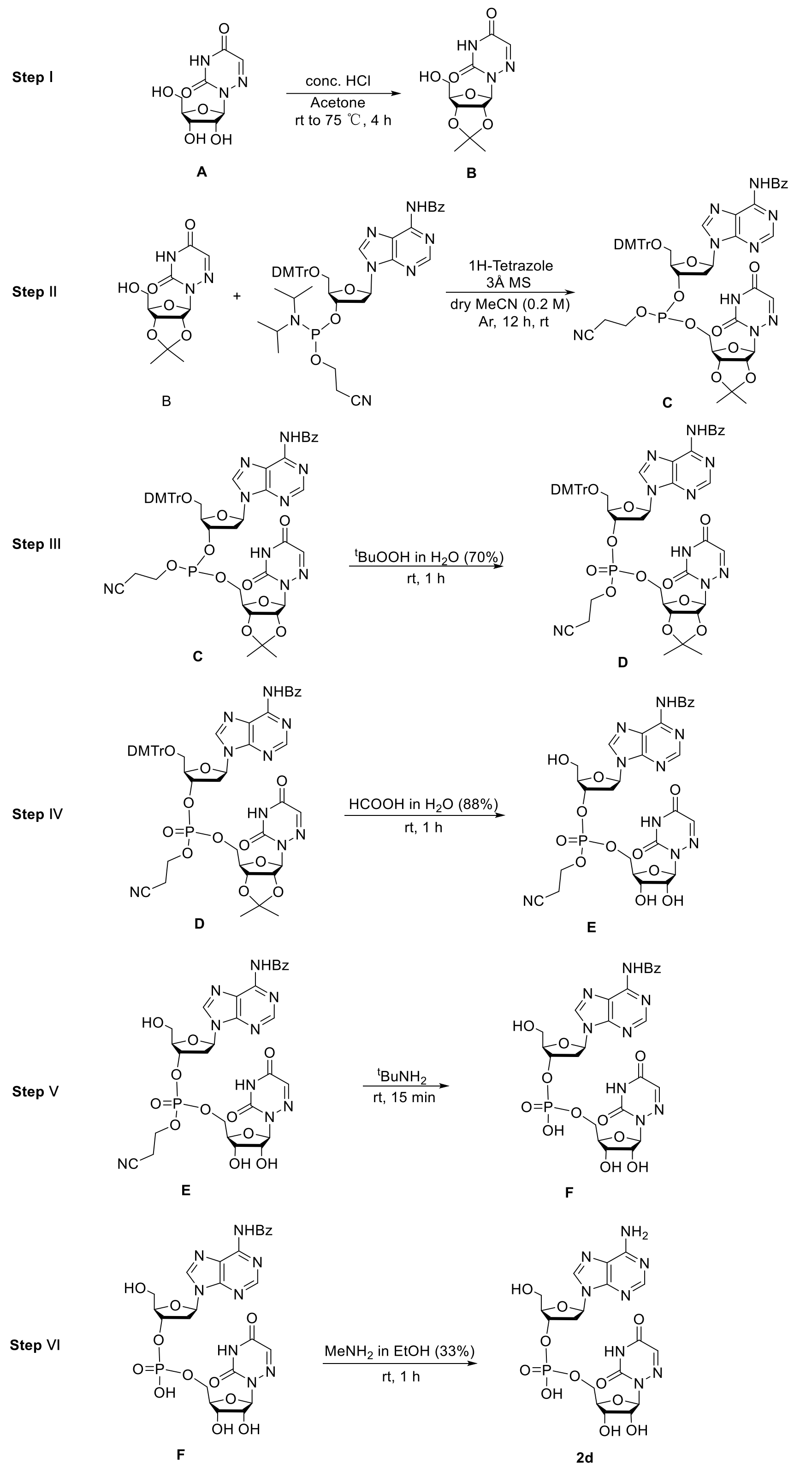

Step I: 6-azauridine **A** (2 mmol, 1.0 equiv) and acetone (5 mL) were added to the pressure-resistant tube, followed by dropwise addition of conc. HCl (40 μL). Then the mixture was stirred at 75 °C for 4 hours. After the reaction was completed, the mixture was cooled to room temperature, Et_3N (0.5 mL) was added dropwise to quench the reaction. The resulting mixture was filtered and the solids are washed with DCM (× 3). The organic layers were combined and concentrated under

reduced pressure. The resulting mixture was purified through silica gel column chromatography using petroleum ether/ethyl acetate as eluent.

Step II: A flame-dried 25 mL Schlenk tube equipped with magnetic stir bar was charged with DMT-dA(bz) phosphoramidite (0.4 mmol, 1.0 equiv), product **B** (0.4 mmol, 1.0 equiv), 1H-tetrazole (0.48 mmol, 1.2 equiv), and flame-dried 3 Å MS (powder, about 100 mg). Ar was substituted for three times. The solids were suspended in anhydrous acetonitrile (2 mL, 0.2 M). TLC could be utilized to monitor the process of the reaction. After vigorous stirring at room temperature for about 12 hours.

Step III: Then the solution of tert-butyl hydroperoxide (70% in H_2O, 5.0 equiv) was added and the reaction mixture was stirred for about an additional 1 hour. The reaction mixture was filtered through a plug of Celite and the filter cake washed with DCM. The filtrate was concentrated under reduced pressure to obtain compound **D**. Due to the presence of multiple protecting groups, the reaction intermediates could be directly used in the next step without separation and purification.

Step IV: To a 100 mL round bottom flask equipped with magnetic stir bar, charged with compound **D**, and closed tightly with rubber septum and a balloon, the formic acid (88% in H_2O, 0.05 M) was added at room temperature under continuous stirring. TLC could be utilized to monitor the process of the reaction. The mixture was concentrated under reduced pressure to obtain compound **E**. The reaction intermediates could be directly used in the next step without separation and purification.

Step V: Then the compound **E** was dissolved in a tert-butyl amine (0.05 M). After 15 minutes, the mixture was concentrated again. The reaction intermediates could be directly used in the next step without separation and purification.

Step VI: The 12 mL the methylamine (solution in methanol, 0.1 M) was added and stirred for about 2 hours. Then the crude material was concentrated and purified by the semi-HPLC (Hanbon Sci.& Tech.) equipped with the Nucifera C18U column to afford the desired product **2d**. Linear gradients using A: MeCN and B: H_2O (0.5% NH_4OAc).

(2*R*,3*S*,5*R*)-5-(6-amino-9H-purin-9-yl)-2-(hydroxymethyl)tetrahydrofuran-3-yl(((2*R*,3*S*,4*R*,5*R*)-5-(3,5-dioxo-4,5-dihydro-1,2,4-triazin-2(3H)-yl)-3,4-dihydroxytetrahydrofuran-2-yl)methyl) hydrogen phosphate (2d) Yield: 55%. **^{1}H NMR** (400 MHz, D_2O) δ 8.09 (s, 1H), 8.02 (s, 1H), 7.25 (s, 1H), 6.24 (t, *J* = 8.0 Hz, 1H), 5.88 (d, *J* = 7.6 Hz, 1H), 4.74 (brs, 1H), 4.39 (t, *J* = 4.0 Hz, 1H), 4.30 (t, *J* = 5.6 Hz, 1H), 4.20-4.19 (m, 1H), 4.06-4.05 (m, 1H), 4.02-3.98 (m, 1H), 3.90-3.85 (m, 1H), 3.67-3.60 (m, 2H), 2.63-2.57 (m, 2H). **^{13}C NMR** (101 MHz, D_2O) δ 181.33, 159.65, 155.57, 152.37, 150.83, 148.19, 140.54, 136.49, 119.07, 89.60, 86.59 (d, *J* = 7.0 Hz), 85.16, 82.35 (d, *J* = 9.0 Hz), 76.64 (d, *J* = 5.4 Hz), 72.37, 69.73, 65.24, 61.81, 37.96. **^{31}P NMR** (162 MHz, D_2O) δ -1.01. **HRMS (ESI)** $C_{18}H_{22}N_8O_{11}P$ $[M-H]^-$ calcd: 557.1138, found: 557.1151.

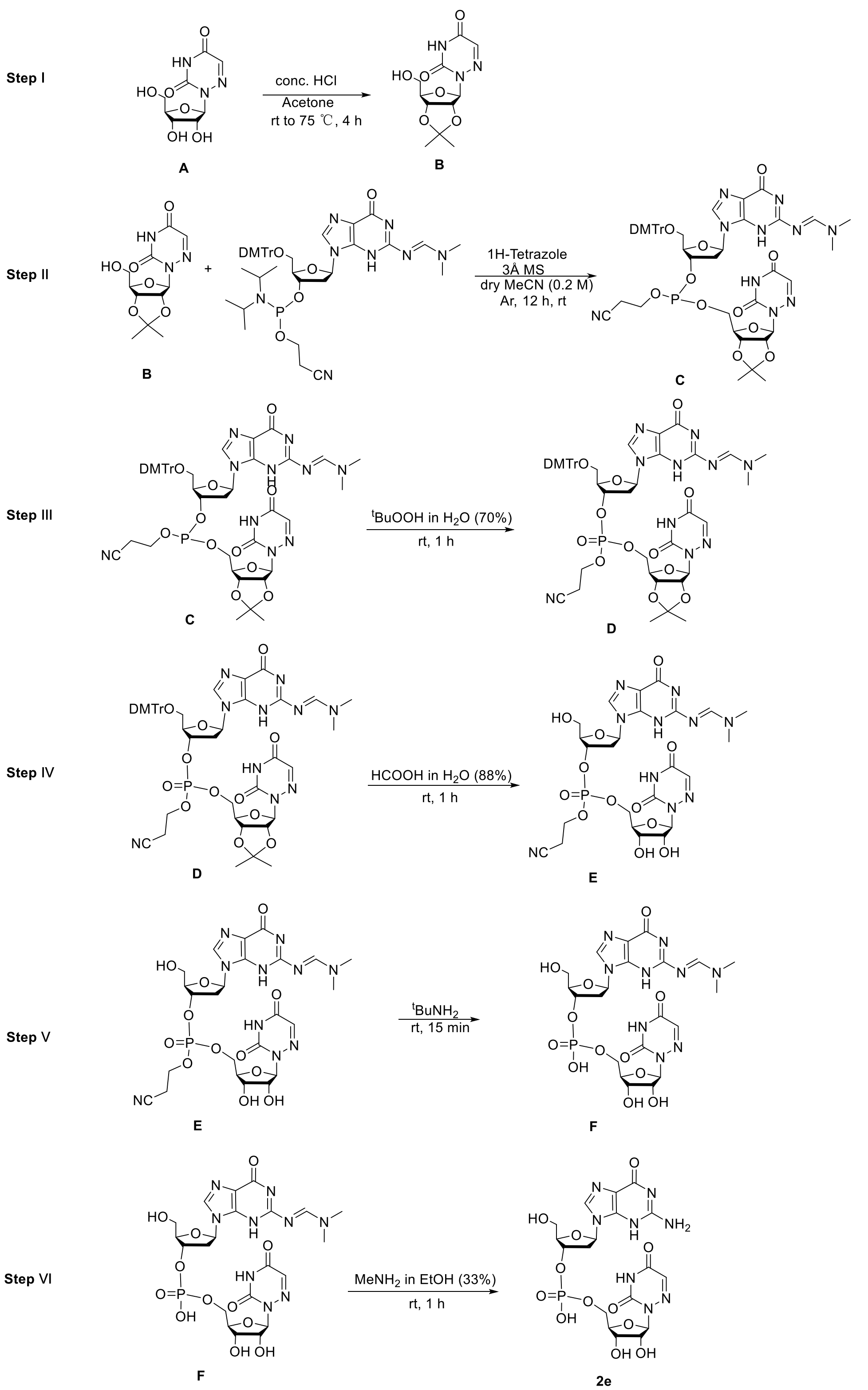

Step I: 6-azauridine **A** (2 mmol, 1.0 equiv) and acetone (5 mL) were added to the pressure-resistant tube, followed by dropwise addition of conc. HCl (40 μL). Then the mixture was stirred at 75 °C for 4 hours. After the reaction was completed, the mixture was cooled to room temperature, Et_3N (0.5 mL) was added dropwise to quench the reaction. The resulting mixture was filtered and the solids are washed with DCM (× 3). The organic layers were combined and concentrated under reduced pressure. The resulting mixture was purified through silica gel column chromatography

using petroleum ether/ethyl acetate as eluent.

Step II: A flame-dried 25 mL Schlenk tube equipped with magnetic stir bar was charged with DMT-dG(dmf) amidite (0.4 mmol, 1.0 equiv), product **B** (0.4 mmol, 1.0 equiv), 1H-tetrazole (0.48 mmol, 1.2 equiv), and flame-dried 3 Å MS (powder, about 100 mg). Ar was substituted for three times. The solids were suspended in anhydrous acetonitrile (2 mL, 0.2 M). TLC could be utilized to monitor the process of the reaction. After vigorous stirring at room temperature for about 12 hours.

Step III: Then the solution of tert-butyl hydroperoxide (70% in H_2O, 5.0 equiv) was added and the reaction mixture was stirred for about an additional 1 hour. The reaction mixture was filtered through a plug of Celite and the filter cake washed with DCM. The filtrate was concentrated under reduced pressure to obtain compound **D**. Due to the presence of multiple protecting groups, the reaction intermediates could be directly used in the next step without separation and purification.

Step IV: To a 100 mL round bottom flask equipped with magnetic stir bar, charged with compound **D**, and closed tightly with rubber septum and a balloon, the formic acid (88% in H_2O, 0.05 M) was added at room temperature under continuous stirring. TLC could be utilized to monitor the process of the reaction. The mixture was concentrated under reduced pressure to obtain compound **E**. The reaction intermediates could be directly used in the next step without separation and purification.

Step V: Then the compound **E** was dissolved in a tert-butyl amine (0.05 M). After 15 minutes, the mixture was concentrated again. The reaction intermediates could be directly used in the next step without separation and purification.

Step VI: The 12 mL the methylamine (solution in methanol, 0.1 M) was added and stirred for about 2 hours. Then the crude material was concentrated and purified by the semi-HPLC (Hanbon Sci.& Tech.) equipped with the Nucifera C18U column to afford the desired product **2e**. Linear gradients using A: MeCN and B: H_2O (0.5% NH_4OAc).

(2*R*,3*S*,5*R*)-5-(2-amino-6-oxo-3,6-dihydro-9H-purin-9-yl)-2-(hydroxymethyl)tetrahydrofuran-3-yl(((2*R*,3*S*,4*R*,5*R*)-5-(3,5-dioxo-4,5-dihydro-1,2,4-triazin-2(3H)-yl)-3,4-dihydroxytetrahydrofuran-2-yl)methyl) hydrogen phosphate (2e) Yield: 60%. **^{1}H NMR** (400 MHz, D_2O) δ 7.77 (s, 1H), 7.28 (s, 1H), 6.09-6.06 (m, 1H), 5.91 (d, J = 3.6 Hz, 1H), 4.72-4.70 (m, 1H), 4.42-4.40 (m, 1H), 4.30 (t, J = 5.2 Hz, 1H), 4.12-4.11 (m, 1H), 4.05 (brs, 1H), 4.05-3.96 (m, 1H), 3.90-3.84 (m, 1H), 3.64-3.57 (m, 2H), 2.60-2.51 (m, 2H). **^{13}C NMR** (101 MHz, D_2O) δ 181.38, 161.36, 158.81, 153.64, 150.99, 138.01, 136.53, 116.65, 89.70, 86.30 (d, J = 6.0 Hz), 84.48, 82.28 (d, J = 9.0 Hz), 76.35 (d, J = 6.0 Hz), 72.39, 69.76, 65.19, 61.69, 37.50. **^{31}P NMR** (162 MHz, D_2O) δ -0.91. **HRMS (ESI)** $C_{18}H_{22}N_8O_{12}P$ $[M-H]^-$ calcd: 573.1073, found: 573.1100.

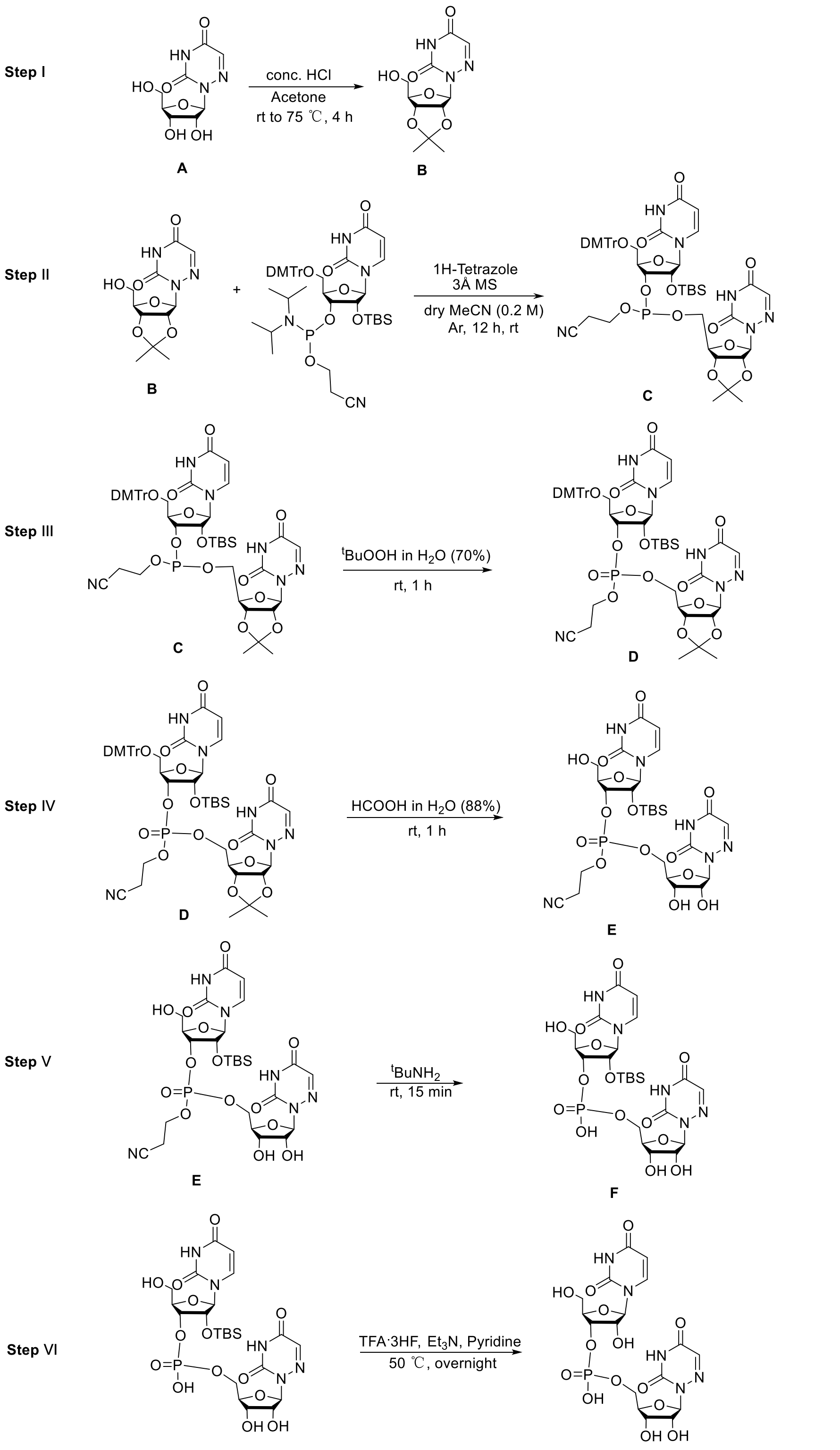

Step I: 6-azauridine A (2 mmol, 1.0 equiv) and acetone (5 mL) were added to the pressure-resistant tube, followed by dropwise addition of conc. HCl (40 μL). Then the mixture was stirred at 75 °C for 4 hours. After the reaction was completed, the mixture was cooled to room temperature, Et_3N (0.5 mL) was added dropwise to quench the reaction. The resulting mixture was filtered and the solids are washed with DCM (× 3). The organic layers were combined and concentrated under reduced pressure. The resulting mixture was purified through silica gel column chromatography

using petroleum ether/ethyl acetate as eluent.

Step II: A flame-dried 25 mL Schlenk tube equipped with magnetic stir bar was charged with DMT-2′O-TBS-rU phosphoramidite (0.4 mmol, 1.0 equiv), product **B** (0.4 mmol, 1.0 equiv), 1H-tetrazole (0.48 mmol, 1.2 equiv), and flame-dried 3 Å MS (powder, about 100 mg). Ar was substituted for three times. The solids were suspended in anhydrous acetonitrile (2 mL, 0.2 M). TLC could be utilized to monitor the process of the reaction. After vigorous stirring at room temperature for about 12 hours.

Step III: Then the solution of tert-butyl hydroperoxide (70% in H_2O, 5.0 equiv) was added and the reaction mixture was stirred for about an additional 1 hour. The reaction mixture was filtered through a plug of Celite and the filter cake washed with DCM. The filtrate was concentrated under reduced pressure to obtain compound **D**. Due to the presence of multiple protecting groups, the reaction intermediates could be directly used in the next step without separation and purification.

Step IV: To a 100 mL round bottom flask equipped with magnetic stir bar, charged with product **D**, and closed tightly with rubber septum and a balloon, the formic acid (88% in H_2O, 0.05 M) was added at room temperature under continuous stirring. TLC could be utilized to monitor the process of the reaction. The mixture was concentrated under reduced pressure to obtain compound **E**. The reaction intermediates could be directly used in the next step without separation and purification.

Step V: Then the compound **E** was dissolved in tert-butyl amine (0.05 M). After 15 minutes, the mixture was concentrated again. The reaction intermediates could be directly used in the next step without separation and purification.

Step VI: The solvent of this mixture was removed and the triethylamine trihydrofluoride (0.17 M), triethylamine (0.14 M), and pyridine (0.26 M) were added. Then the round bottom flask was placed in oil bath and heated at 50 °C overnight. The mixture was concentrated to a small volume and purified by the semi-HPLC (Hanbon Sci.& Tech.) equipped with the Nucifera C18U column to afford the desired product **2f**. Linear gradients using A: MeCN and B: H_2O (0.5% NH_4OAc).

(2*R*,3*S*,4*R*,5*R*)-5-(2,4-dioxo-3,4-dihydropyrimidin-1(2H)-yl)-4-hydroxy-2-(hydroxymethyl)tetrahydrofuran-3-yl(((2*R*,3*S*,4*R*,5*R*)-5-(3,5-dioxo-4,5-dihydro-1,2,4-triazin-2(3H)-yl)-3,4-dihydroxytetrahydrofuran-2-yl)methyl) hydrogen phosphate (2f) Yield: 59%. **^{1}H NMR** (400 MHz, D_2O) δ 7.74 (d, *J* = 8.1 Hz, 1H), 7.47 (s, 1H), 6.01 (d, *J* = 2.4 Hz, 1H), 5.81-5.79 (m, 2H), 4.46-4.43 (m, 2H), 4.35 (t, *J* = 6.0 Hz, 1H), 4.28 (t, *J* = 5.6 Hz, 1H), 4.24-4.23 (m, 1H), 4.13-4.09 (m, 1H), 4.05-4.01 (m, 1H), 3.88-3.83 (m, 1H), 3.65 (d, *J* = 3.2 Hz, 2H). **^{13}C NMR** (101 MHz, D_2O) δ 181.33, 166.10, 157.81, 151.75, 141.48, 136.81, 102.68, 90.08, 87.90, 83.41, 82.36, 72.98, 70.38, 66.19, 60.84, 51.87, 38.67. **^{31}P NMR** (162 MHz, D_2O) δ -0.95. **HRMS (ESI)** $C_{17}H_{21}N_5O_{14}P$ $[M-H]^-$ calcd: 550.0807, found: 550.0828.

2.3 Synthesis of 6-azauridine nucleoside phosphoramidite (DMT-2'O-TBS-6-Aza-rU phosphoramidite) [3,4]

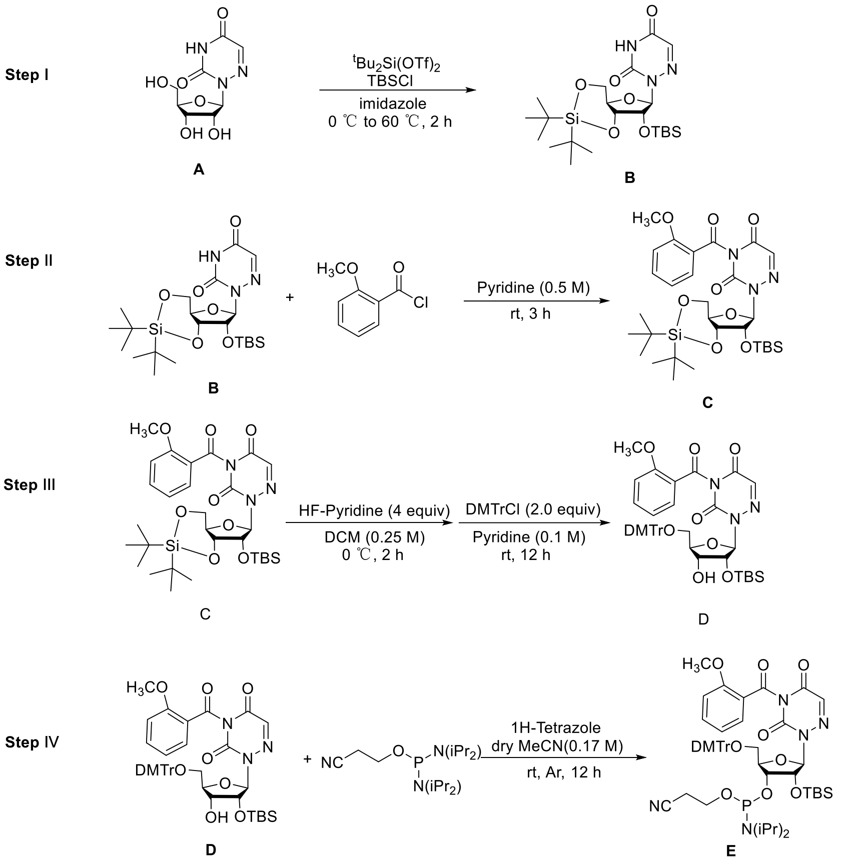

Step I: To a stirred suspension of the 6-azauridine **A** (15 mmol) in DMF (30 mL) at 0 °C was added di-tert-butylsilandiyl ditriflate (6.6 mL, 18 mmol) dropwise over 15 min. After stirring at the same temperature for 30 min, imidazole (5.1 g, 75 mmol) was added, the resulting mixture was stirred at 0 °C for another 5 min and then at room temperature for 45 min. Thereafter tertbutyldimethylchlorosilane (3.4 g, 22.5 mmol) was added and the reaction mixture was stirred at 60 °C for 2 hours. Then the DMF was partially removed in vacuo and the remaining reaction mixture was partially partitioned between water (30 mL) and ether (30 mL). The layers were separated and the aqueous layer was extracted with ether (3 × 20 mL). The combined organic extract was washed with brine (50 mL), dried over anhydrous Na_2SO_4, concentrated in vacuo and purified by column chromatography (8:1 petroleum ether/ethyl acetate to 6:1 petroleum ether/ethyl acetate) to obtain product **B**.

2-((4*aR*,6*R*,7*R*,7*aR*)-2,2-di-tert-butyl-7-((tert-butyldimethylsilyl)oxy)tetrahydro-4H-furo[3,2-d][1,3,2]dioxasilin-6-yl)-1,2,4-triazine-3,5(2H,4H)-dione (product **B)** Yield: 80%. **^{1}H NMR** (600 MHz, $CDCl_3$) δ 9.15 (brs, 1H), 7.39 (s, 1H), 5.99 (s, 1H), 4.34-4.30 (m, 2H), 4.09-4.05 (m, 1H), 4.03-3.99 (m, 1H), 3.84-3.81 (m, 1H), 1.00 (s, 9H), 0.96 (s, 9H), 0.84 (s, 9H), 0.05 (s, 6H).

Step II: product **B** (12 mmol, 1.0 equiv) in pyridine (0.5 M) at room temperature was added *o*-anisoyl chloride (1.5 equiv) dropwise over 5 min. After being stirred for 3 hours, the mixture was co-evaporated with toluene to remove pyridine completely. The crude compound **C** was purified through silica gel column chromatography using petroleum ether/ethyl acetate (8:1) as eluent.

2-((4*aR*,6*R*,7*R*,7*aR*)-2,2-di-tert-butyl-7-((tert-butyldimethylsilyl)oxy)tetrahydro-4H-furo[3,2-d][1,3,2]dioxasilin-6-yl)-4-(2-methoxybenzoyl)-1,2,4-triazine-3,5(2H,4H)-dione (product **C)**

Yield: 70%. **^{1}H NMR** (600 MHz, $CDCl_3$) δ 8.09 (d, *J* = 7.8 Hz, 1H), 7.61 (t, *J* = 6.0 Hz, 1H), 7.59 (s, 1H), 7.09 (t, *J* = 7.8 Hz, 1H), 6.93 (d, *J* = 8.4 Hz, 1H), 6.07 (s, 1H), 4.44-4.39 (m, 2H), 4.21-4.19 (m, 1H), 4.10-4.07 (m, 1H), 3.91 (t, *J* = 9.6 Hz, 1H), 3.74 (s, 3H), 1.08 (s, 9H), 1.03 (s, 9H), 0.91 (s, 9H), 0.12 (s, 6H).

Step III: To a solution of product **C** (8.4 mmol, 1.0 equiv) in DCM (0.25 M) at 0 °C was added dropwise over 15 min a chilled solution of hydrogen fluoride–pyridine complex (4 equiv) in pyridine (6 mL). The reaction mixture was stirred at 0 °C for 2 hours. Thereafter, the reaction mixture was washed with saturated $NaHCO_3$ solution (× 3) and extracted with DCM (× 3). The combined organic extract was washed with saturated NaCl (× 3), dried over anhydrous Na_2SO_4, concentrated and dried in vacuo. For desilylated compound was dissolved in anhydrous pyridine (0.1 M). DMTrCl (2.0 equiv) was added and the reaction mixture stirred at room temperature for 12 hours. The reaction was quenched by addition of MeOH (3 mL). Concentration of the reaction mixture gave a viscous liquid which was washed with saturated $NaHCO_3$ and the aqueous layer was then extracted with DCM (× 3). The combined organic extract was washed with saturated NaCl (× 3), dried over anhydrous Na_2SO_4 and concentrated in vacuo. The resulting residue was purified by flash column chromatography using elution gradient from 8:1 petroleum ether/ethyl acetate to 4:1 petroleum ether/ethyl acetate to afford pure product **D**.

2-((2*R*,3*R*,4*R*,5*R*)-5-((bis(4-methoxyphenyl)(phenyl)methoxy)methyl)-3-((tert-butyldimethylsilyl)oxy)-4-hydroxytetrahydrofuran-2-yl)-4-(2-methoxybenzoyl)-1,2,4-triazine-3,5(2H,4H)-dione (product **D)** Yield: 40%. **^{1}H NMR** (600 MHz, $CDCl_3$) δ 8.10 (d, *J* = 6.6 Hz, 1H), 7.60 (t, *J* = 8.4 Hz, 1H), 7.47-7.46 (m, 3H), 7.34 (d, *J* = 7.2 Hz, 4H), 7.28-7.25 (m, 2H), 7.21 (t, *J* = 7.2 Hz, 1H), 7.09 (t, *J* = 7.8 Hz, 1H), 6.91 (d, *J* = 8.4 Hz, 1H), 6.81 (d, *J* = 9.0 Hz, 4H), 6.15 (d, *J* = 3.6 Hz, 1H), 4.73-4.72 (m, 1H), 4.26-4.25 (m, 1H), 3.79 (s, 6H), 3.70 (s, 3H), 3.42-3.40 (m, 1H), 3.21-3.18 (m, 1H), 2.60 (d, *J* = 6.6 Hz, 1H), 0.92 (s, 9H), 0.12 (d, *J* = 6.0 Hz, 6H).

Step IV: To a solution of the product **D** (3.4 mmol) in anhydrous MeCN (9 mL) was added 1H-tetrazole (1.0 equiv) under Ar atomosphere. Thereafter, $NCCH_2CH_2OP(N(i\text{-}Pr)_2)_2$ (2.0 equiv) was added dropwise at room temperature and the reaction mixture was stirred for 12 hours. The mixture was concentrated under reduced pressure to obtain crude compound **E**, which was purified through recycling preprative HPLC system (Japan Analytical Industry Co.,Ltd LaboACE LC-5060 Plus II, ethyl acetate).

(2*R*,3*R*,4*R*,5*R*)-2-((bis(4-methoxyphenyl)(phenyl)methoxy)methyl)-4-((tert-butyldimethylsilyl)oxy)-5-(4-(2-methoxybenzoyl)-3,5-dioxo-4,5-dihydro-1,2,4-triazin-2(3H)-yl)tetrahydrofuran-3-yl (2-cyanoethyl) diisopropylphosphoramidite (product **E)** Yield: 70%. **^{1}H NMR** (600 MHz, $CDCl_3$) δ 8.07 (t, *J* = 6.0 Hz, 1H), 7.59 (t, *J* = 6.0 Hz, 1H), 7.51-7.46 (m, 3H), 7.36 (q, *J* = 6.0 Hz, 4H), 7.29-7.25 (m, 2H), 7.23-7.19 (m, 1H), 7.07 (t, *J* = 6.0 Hz, 1H), 6.91 (d, *J* = 6.0 Hz, 1H), 6.81 (t, *J* = 9.0 Hz, 4H), 6.18 (dd, *J* = 4.8 Hz, 6.0, 1H), 4.80 (dt, *J* = 4.8 Hz, 6.0, 1H), 4.33-4.30 (m, 1.4H), 4.25-4.24 (m, 0.6H), 3.91-3.86 (m, 0.6H), 3.82-3.81 (m, 0.4H), 3.78 (d, *J* = 4.8 Hz, 6H), 3.71 (d, *J* = 10.2 Hz, 3H), 3.66-3.61 (m, 0.6H), 3.58-3.53 (m, 1.4H), 3.49-3.37 (m, 1H), 3.11-3.06 (m, 1H), 2.64-2.54 (m, 1H), 1.27-1.24 (m, 1H), 1.20-1.18 (m, 1H), 1.15-1.12 (m, 9H), 0.99 (d, *J* = 6.6 Hz, 3H), 0.89 (d, *J* = 7.2 Hz, 9H), 0.11-0.08 (m, 6H). **^{31}P NMR** (162 MHz, $CDCl_3$) δ 148.67.

The spectra of the isolated product **B\C\D\E** matched that of previously reported material. [3,4]

Oligonucleotide **2g** was custom synthesized by Accurate Biology.

2.4 Synthesis of 2h-2p

Synthesis of 2h

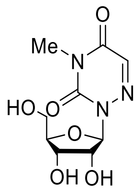

2h

Iodomethane (5.0 mmol, 1.0 equiv) was added dropwise to a stirring solution of 6-azauridine (5.0 mmol, 1.0 equiv), K_2CO_3 (2.5 mmol, 0.5 equiv) in DMF (20 mL). The reaction mixture was allowed to stir at room temperature for 16 hours. Then, the mixture was quenched with saturated Na_2CO_3 solution and extracted with DCM (× 3). The organic layers were combined, dried over anhydrous Na_2SO_4 and concentrated under reduced pressure. Purification by column chromatography to give **2h**.

2-((2*R*,3*R*,4*S*,5*R*)-3,4-dihydroxy-5-(hydroxymethyl)tetrahydrofuran-2-yl)-4-methyl-1,2,4-triazine-3,5(2H,4H)-dione (2h) Yield: 92%. **^{1}H NMR** (600 MHz, D_2O) δ 7.41 (s, 1H), 5.93 (s, 1H), 4.37-4.36 (m, 1H), 4.17-4.15 (m, 1H), 3.89-3.87 (m, 1H), 3.62-3.59 (m, 1H), 3.49-3.46 (m, 1H), 3.07 (s, 3H). **^{13}C NMR** (150 MHz, D_2O) δ 157.33, 149.95, 135.55, 90.76, 84.04, 72.85, 70.11, 61.41, 26.86. **HRMS (ESI)** $C_9H_{13}N_3NaO_6$ $[M+Na]^+$ calcd: 282.0703, found: 282.0697.

Synthesis of 2i and 2j [5, 6]

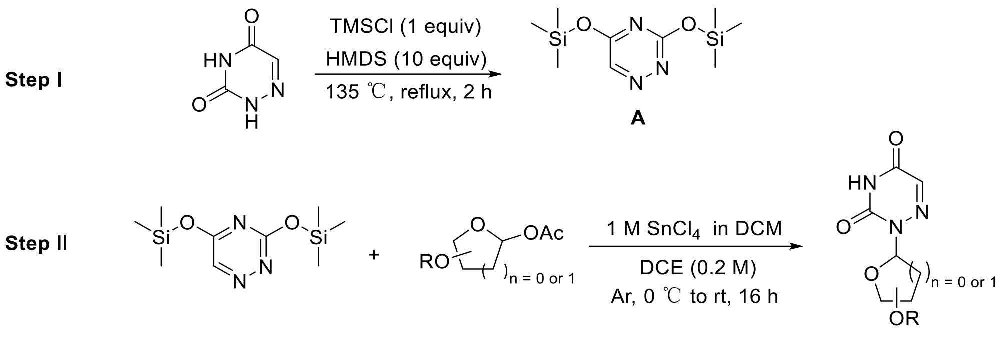

Step I: 6-Azauracil (1.0 equiv) and HMDS (10 equiv) were added to the pressure-resistant tube, followed by dropwise addition of TMSCl (1 equiv), and the mixture was refluxed and stirred at 135 °C for 2 hours. After the reaction completed, the mixture was cooled to room temperature and concentrated under reduced pressure to obtain compound **A**. The compound **A** could be directly used in the next step without separation and purification.

Step II: To a round-bottom flask with a stirring bar was added compound **A** (1.2 equiv) and α/β 1-*O*-acetyl-2-deoxyl-3,4-*O*-benzoy-*D*-ribose or β-*D*-Glucose pentaacetate (1.0 equiv). Then, air was withdrawn and backfilled with Ar (× 3). DCE (0.2 M) was added, and 1 M $SnCl_4$ (0.75 equiv) in DCM was dropwise into the mixture at 0 °C. The reaction mixture was warmed to room temperature and stirred for 16 hours. Then, the mixture was diluted with DCM, and washed by saturated $NaHCO_3$ solution (× 3), brine (× 3). The organic layers were combined, dried over anhydrous Na_2SO_4 and concentrated under reduced pressure. Purification by column chromatography to give product **2i** and **2j**.

((2*R*,3*S*)-3-(benzoyloxy)-5-(3,5-dioxo-4,5-dihydro-1,2,4-triazin-2(3H)-yl)tetrahydrofuran-2-yl)methyl benzoate (2i) Yield: 65%. **^{1}H NMR** (400 MHz, $CDCl_3$) δ 9.02 (s, 0.5H), 8.98 (s, 0.5H), 8.07-8.02 (m, 4H), 7.61-7.55 (m, 2H), 7.49-7.41 (m, 4.5H), 7.29 (s, 0.5H), 6.69-6.68 (m, 0.5H), 6.64-6.61 (m, 0.5H), 5.76-5.74 (m, 0.5H), 5.53-5.51 (m, 0.5H), 4.82-4.81 (m, 0.5H), 4.70-4.60 (m, 1H), 4.56-4.49 (m, 1.5H), 3.04-2.98 (m, 1H), 2.68 (dt, *J* = 4.0 Hz, 6.4 Hz, 0.5H), 2.54-2.48 (m, 0.5H). **^{13}C NMR** (101 MHz, $CDCl_3$) δ 166.17, 166.12, 165.97, 155.46, 155.26, 147.72, 147.54, 136.03, 135.49, 133.60, 133.56, 133.29, 133.25, 129.78, 129.74, 129.70, 128.55, 128.48, 128.43, 86.51, 85.93, 82.49, 82.35, 75.06, 74.41, 64.23, 64.11, 35.99, 35.04. **HRMS (ESI)** $C_{22}H_{19}N_3NaO_7$ $[M+Na]^+$ calcd: 460.1122, found: 460.1115.

(2*R*,3*R*,4*S*,5*R*,6*S*)-2-(acetoxymethyl)-6-(3,5-dioxo-4,5-dihydro-1,2,4-triazin-2(3H)-yl)tetrahydro-2H-pyran-3,4,5-triyl triacetate (2j) Yield: 82%. **^{1}H NMR** (400 MHz, $CDCl_3$) δ 9.13 (s, 1H), 7.43 (s, 1H), 7.20 (s, 1H), 5.82 (d, *J* = 9.2 Hz, 1H), 5.60 (t, *J* = 10.0 Hz, 1H), 5.30 (t, *J* = 9.6 Hz, 1H), 5.12 (t, *J* = 9.6 Hz, 1H), 4.22-4.18 (m, 1H), 3.88-3.84 (m, 1H), 2.02 (s, 3H), 1.99 (s, 3H), 1.96 (s, 3H), 1.91 (s, 3H). **^{13}C NMR** (101 MHz, $CDCl_3$) δ 169.64, 169.22, 168.36, 167.91, 153.95, 146.67, 135.27, 73.34, 72.52, 67.01, 66.48, 60.53, 59.42, 20.07, 19.73, 19.57, 19.55. **HRMS (ESI)** $C_{17}H_{21}N_3NaO_{11}$ $[M+Na]^+$ calcd: 466.1079, found: 466.1068.

Synthesis of α/β 1-O-acetyl-2-deoxyl-3,4-O-benzoy-D- ribose [7]

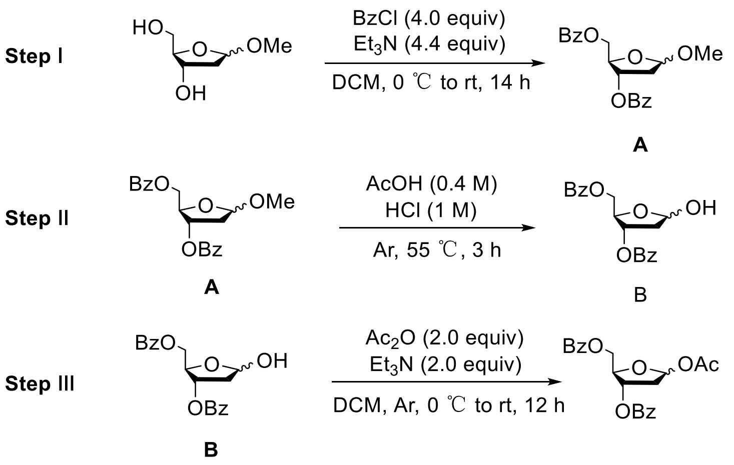

Step I: In a 25 mL round-bottom flask, 1-*O*-methyl-2-deoxy-*D*-ribose (3.0 mmol, 1.0 equiv) was dissolved in 10 mL DCM. Benzoyl chloride (12.0 mmol, 4.0 equiv) and Et_3N (13.2 mmol, 4.4 equiv) were dropwise in the mixture at 0 °C. The reaction mixture was warmed to room temperature and stirred for 14 hours. Then, the mixture was quenched with H_2O and extracted with DCM (× 3). The resulting mixture was washed by 1 M HCl (× 3) and brine (× 3). The organic layers were combined, dried over anhydrous Na_2SO_4 and concentrated under reduced pressure. Purification by column chromatography to give product **A**.

Step II: To a round-bottom flask with a stirring bar was added product **A** (1.0 equiv). Then, air was withdrawn and backfilled with Ar (× 3). AcOH (2.5 mL/mmol) was added, and 1 M HCl aqueous solution (2.5 mL/mmol) was dropwise in the mixture at room temperature and stirred at 55 °C for 3 hours. After the reaction was completed, the mixture was cooled to room temperature, water and toluene were added to the reaction mixture, followed by extraction with toluene. The organic layer was washed with saturated $NaHCO_3$ solution (× 3) and brine (× 3), then combined, dried over anhydrous Na_2SO_4, and concentrated under reduced pressure. Purification by column chromatography to give product **B**.

Step III: To a round-bottom flask with a stirring bar was added product **B** (1.0 equiv). Then, air was withdrawn and backfilled with Ar (× 3). DCM was added, Ac_2O (2.0 equiv) and Et_3N (2.0 equiv) were dropwise in the mixture at 0 °C. The reaction mixture was warmed to room temperature and stirred for 12 hours. Then, the mixture was quenched with H_2O and extracted with DCM for three times. The organic layer was washed by 1 M HCl (× 3), saturated $NaHCO_3$ solution (× 3), and brine (× 3), then combined, dried over anhydrous Na_2SO_4 and concentrated under reduced pressure. Purification by column chromatography to give the desired product α/β 1-*O*-acetyl-2-deoxyl-3,4-*O*-benzoy-*D*-ribose.

The spectra of the isolated product matched that of previously reported materials. [7]

General Procedure 3 (GP3) for the Synthesis of 2k-2p [8]

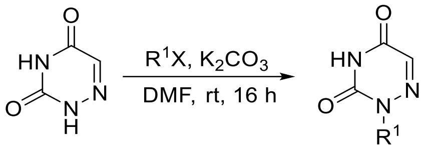

Alkyl halides (5.0 mmol, 1.0 equiv) was added dropwise to a stirring solution of 6-azauracil (5.0 mmol, 1.0 equiv), K_2CO_3 (2.5 mmol, 0.5 equiv) in DMF (20 mL). The reaction mixture was allowed to stir at room temperature for 16 hours. Then, the mixture was quenched with saturated Na_2CO_3 solution and extracted with DCM for three times. The organic layers were combined, dried over anhydrous Na_2SO_4 and concentrated under reduced pressure. The crude products were purified through silica gel column chromatography using petroleum ether/ethyl acetate as eluent to give to afford the corresponding products.

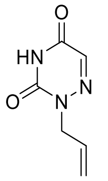

2k

The compound was prepared according to GP3 using allyl bromide (1.0 equiv), and 6-azauracil (1.0 equiv), K_2CO_3 (0.5 equiv) for the monoalkylation. Purification by column chromatography gave **2k** as white solid.

2-allyl-1,2,4-triazine-3,5(2H,4H)-dione (2k) Yield: 80%. **^{1}H NMR** (600 MHz, $CDCl_3$) δ 9.60 (s, 1H), 7.42 (s, 1H), 5.91-5.84 (m, 1H), 5.33 (d, J = 16.8 Hz, 1H), 5.27 (d, J = 10.2 Hz, 1H), 4.52 (d, J = 6.0 Hz, 2H). **^{13}C NMR** (150 MHz, $CDCl_3$) δ 155.67, 149.55, 135.54, 129.85, 119.54, 42.09. **HRMS (ESI)** $C_6H_7N_3NaO_2$ $[M+Na]^+$ calcd: 176.0437, found: 176.0430.

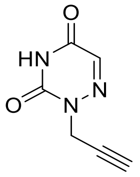

2l

The compound was prepared according to GP3 using propargyl bromide (1.0 equiv), and 6-azauracil (1.0 equiv), K_2CO_3 (0.5 equiv) for the monoalkylation. Purification by column chromatography gave **2l** as white solid.

2-(prop-2-yn-1-yl)-1,2,4-triazine-3,5(2H,4H)-dione (2l) Yield: 82%. **^{1}H NMR** (400 MHz, $CDCl_3$) δ 11.80 (s, 1H), 7.45 (s, 1H), 4.68 (d, J = 4.0 Hz, 2H), 2.26 (s, 1H). **^{13}C NMR** (101 MHz, $CDCl_3$) δ 155.13, 148.67, 135.12, 76.31, 71.84, 36.66. **HRMS (ESI)** $C_6H_5N_3NaO_2$ $[M+Na]^+$ calcd: 174.0278, found: 174.0274.

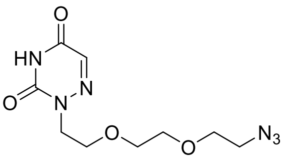

2m

The compound was prepared according to GP3 using bromo-PEG$_2$-azide (1.0 equiv), and 6-azauracil (1.0 equiv), K_2CO_3 (0.5 equiv) for the monoalkylation. Purification by column

chromatography gave **2m** as colorless oil.

2-(2-(2-(2-azidoethoxy)ethoxy)ethyl)-1,2,4-triazine-3,5(2H,4H)-dione (2m) Yield: 75%. **^{1}H NMR** (400 MHz, $CDCl_3$) δ 10.51 (s, 1H), 7.33 (s, 1H), 3.77 (t, *J* = 6.0 Hz, 1H), 3.71 (t, *J* = 5.6 Hz, 2H), 3.64-3.56 (m, 7H), 3.31 (t, *J* = 4.0 Hz, 2H). **^{13}C NMR** (101 MHz, $CDCl_3$) δ 156.18, 149.53, 135.29, 70.65, 70.11, 70.03, 67.05, 50.66, 39.03. **HRMS (ESI)** $C_9H_{14}N_6NaO_4$ $[M+Na]^+$ calcd: 293.0980, found: 293.0969.

Synthesis of 2n

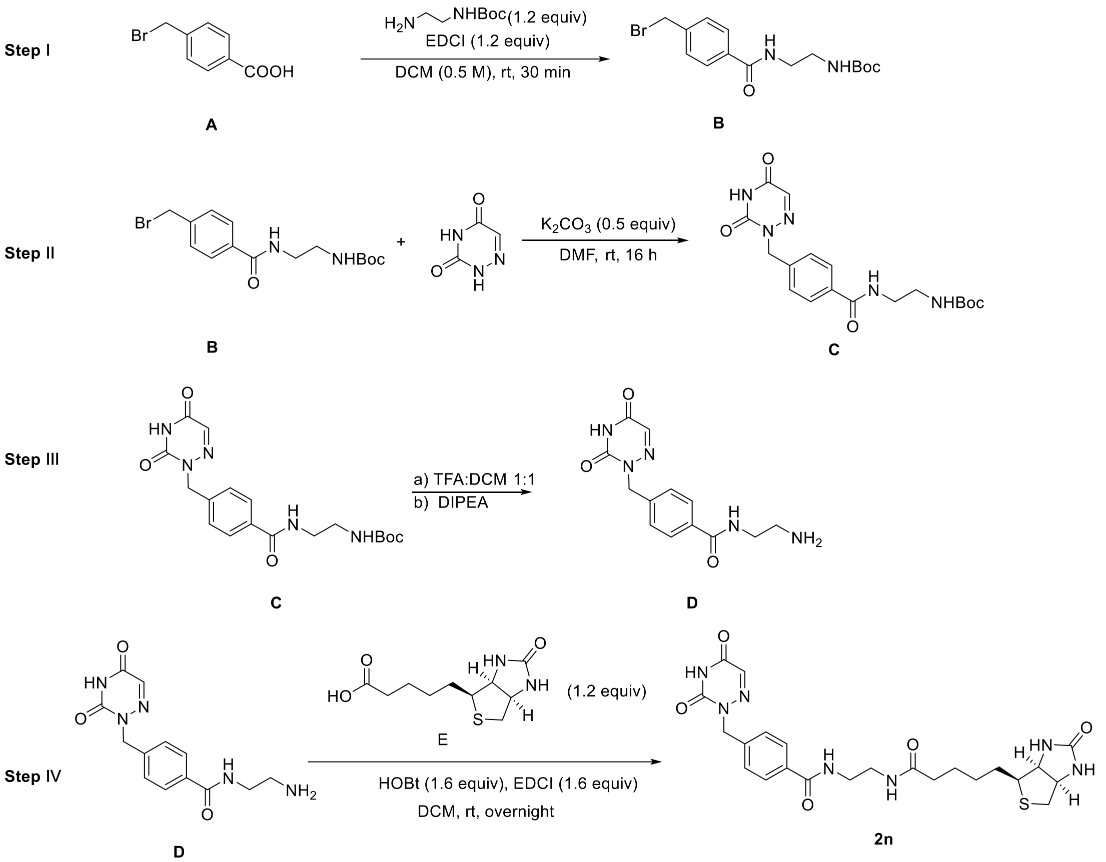

Step I: To a 100 mL round-bottom flask, 4-bromomethylbenzoic acid (10.0 mmol, 1.0 equiv), *N*-Boc-ethylenediamine (1.2 equiv), and EDCI (1.2 equiv) were dissolved in 20 mL DCM. The reaction mixture was allowed to stir at room temperature for 30 min. After completion of the reaction monitored by TLC, the reaction mixture was concentrated under reduced pressure. The compound **B** were purified through silica gel column chromatography using petroleum ether/ethyl acetate as eluent.

tert-butyl (2-(4-(bromomethyl)benzamido)ethyl)carbamate Yield: 80%. **^{1}H NMR** (400 MHz, $CDCl_3$) δ 7.82 (d, *J* = 8.0Hz, 2H), 7.45 (d, *J* = 8.0Hz, 2H), 4.97 (brs, 1H), 4.61 (s, 2H), 3.58-3.54 (m, 2H), 3.44-3.41 (m, 2H), 1.43 (s, 9H). **^{13}C NMR** (101 MHz, $CDCl_3$) δ 167.13, 155.35, 140.71, 134.15, 128.63, 127.47, 80.15, 45.44, 42.37, 39.90, 28.34.

Step II: The compound **C** was prepared according to GP3 using compound **B** (8.0 mmol, 1.0 equiv), and 6-azauracil (1.0 equiv), K_2CO_3 (0.5 equiv) for the monoalkylation. Purification by column chromatography to give compound **C**.

tert-butyl (2-(4-((3,5-dioxo-4,5-dihydro-1,2,4-triazin-2(3H)-yl) methyl) benzamido) ethyl) carbamate Yield: 63%. **^{1}H NMR** (400 MHz, $CDCl_3$) δ 7.76 (dd, *J* = 8.0Hz, 4.0Hz, 2H), 7.70 (q, *J*

= 4.0Hz, 0.5H),7.53 (q, J = 4.0Hz, 0.5H), 7.47-7.38 (m, 3H), 5.12 (d, J = 12.0Hz, 2H), 5.02 (brs, 1H), 4.22 (t, J = 8.0Hz, 1H), 3.56-3.52 (m, 2H), 3.44-3.38 (m, 2H), 1.42 (s, 9H). **^{13}C NMR** (101 MHz, $CDCl_3$) δ 167.78, 155.68, 148.53, 134.64, 130.89, 129.29, 128.81, 128.67, 127.55, 127.37, 80.05, 55.20, 39.97, 38.74, 28.35.

Step III: The compound **C** (6 mmol, 1.0 equiv) was dissolved in 20 mL of 50% TFA in DCM at room temperature. After completion of the reaction monitored by TLC, DIPEA was added dropwise to quench the reaction. Then the reaction mixture was concentrated under reduced pressure.

Step IV: In a 50 mL round-bottom flask, biotin derivative **E** (1.2 equiv) was dissolved in DCM (0.33 M), HOBt (1.6 equiv) and EDCI (1.6 equiv) were added in turn. The mixture was stirred at room temperature for 30 min, the DCM solution of the compound **D** (1.0 equiv) and DIPEA (4.0 equiv) was added into the above mixture at 0 °C. The reaction mixture was warmed to room temperature and stirred overnight. The resulting mixture was washed by citric acid solution (× 3), saturated $NaHCO_3$ solution (× 3) and brine (× 2). The organic layer was dried over Na_2SO_4 and concentrated in vacuo. The residue was purified by column chromatography and **2n** was obtained.

4-((3,5-dioxo-4,5-dihydro-1,2,4-triazin-2(3H)-yl)methyl)-*N*-(2-(5-((3*aS*,4*S*,6*aR*)-2-oxohexahydro-1H-thieno[3,4-d]imidazol-4-yl)pentanamido)ethyl)benzamide (2n) Yield: 49%. **^{1}H NMR** (400 MHz, DMSO) δ 7.85-7.39 (m, 4H), 5.05 (s, 1H), 4.44 (brs, 1H), 3.65-3.64 (m, 1H), 3.45 (brs, 1H), 3.37 (brs, 1H), 3.13-3.12 (m, 1H), 2.85-2.83 (m, 1H), 2.68-2.64 (m, 1H), 2.17 (brs, 1H), 1.55 (brs, 1H), 1.30 (s, 10H). **^{13}C NMR** (101 MHz, DMSO) δ 172.88, 166.48, 163.18, 156.73, 149.64, 139.51, 135.34, 127.85, 127.78, 61.45, 59.65, 55.84, 53.97, 42.51, 42.24, 38.64, 35.71, 28.63, 28.48, 25.70. **HRMS (ESI)** $C_{23}H_{29}N_7NaO_5S$ $[M+Na]^+$ calcd: 538.1860, found: 538.1843.

Synthesis of 2o

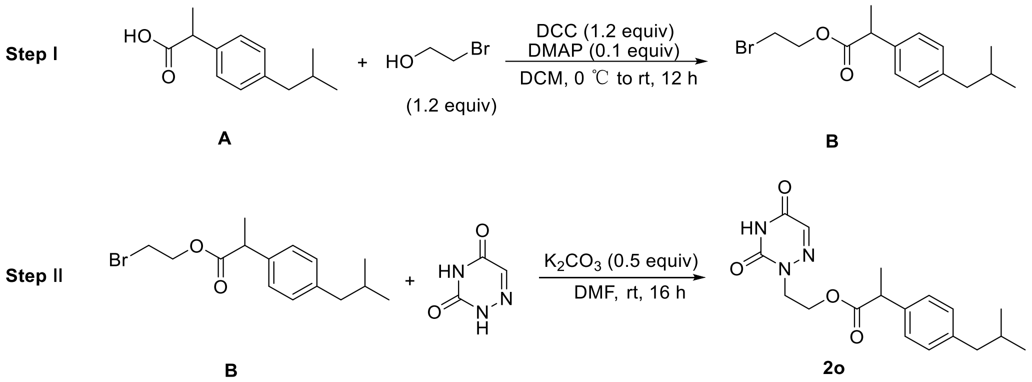

Step I: In a 50 mL round-bottom flask, ibuprofen **A** (5.0 mmol, 1.0 equiv), DCC (6.0 mmol, 1.2 equiv) and DMAP (0.5 mmol, 0.1 equiv) were dissolved in 30 mL DCM. At 0 °C, 2-bromoethanol (6.0 mmol, 1.2 equiv) was added and stirred for 5 min. The reaction mixture was warmed to room temperature and stirred overnight. After completion of the reaction monitored by TLC, the resulting mixture was filtered and the solid was washed with DCM (× 3). The filtrate was concentrated under reduced pressure. The compound **B** were purified through silica gel column chromatography using petroleum ether/ethyl acetate as eluent.

2-bromoethyl 2-(4-isobutylphenyl)propanoate Yield: 84%. **^{1}H NMR** (400 MHz, $CDCl_3$) δ 7.20 (d, J = 8.0 Hz, 2H), 7.09 (d, J = 8.0 Hz, 2H), 4.34 (dd, J = 8.0 Hz, 4.0 Hz, 2H), 3.72 (q, J = 4.0 Hz, 1H), 3.44-3.40 (m, 2H), 2.44 (d, J = 8.0 Hz, 2H), 1.85-1.82 (m, 1H), 1.50 (d, J = 8.0 Hz, 3H), 0.90-0.88 (d, J = 8.0 Hz, 6H). **^{13}C NMR** (101 MHz, $CDCl_3$) δ 174.24, 140.68, 137.38, 129.41, 127.24,

63.95, 45.08, 45.04, 30.22, 28.60, 22.44, 18.51.

Step II: The product **2o** was prepared according to GP3 using compound **B** (1.0 equiv), 6-azauracil (1.0 equiv) and K_2CO_3 (0.5 equiv) for the monoalkylation. Purification by column chromatography gave **2o**.

2-(3,5-dioxo-4,5-dihydro-1,2,4-triazin-2(3H)-yl)ethyl-2-(4-isobutylphenyl)propanoate (2o) Yield: 60%. **^{1}H NMR** (600 MHz, $CDCl_3$) δ 9.30 (s, 1H), 7.31 (s, 1H), 7.15 (d, J = 7.8 Hz, 2H), 7.07 (d, J = 7.8 Hz, 2H), 4.42-4.38 (m, 1H), 4.28-4.24 (m, 2H), 4.15-4.11 (m, 1H), 3.65 (q, J = 7.2 Hz, 1H), 2.44 (d, J = 7.2 Hz, 2H), 1.86-1.84 (m, 1H), 1.46 (d, J = 7.2 Hz, 3H), 0.90 (d, J = 6.6 Hz, 6H). **^{13}C NMR** (150 MHz, $CDCl_3$) δ 174.67, 155.88, 149.28, 140.63, 137.35, 135.24, 129.33, 127.21, 60.74, 45.04, 39.02, 30.18, 22.41, 22.38, 18.47, 18.31. **HRMS (ESI)** $C_{18}H_{23}N_3NaO_4$ $[M+Na]^+$ calcd: 368.1598, found: 368.1581.

2.5 Synthesis of FAM-PEG$_3$-N$_3$

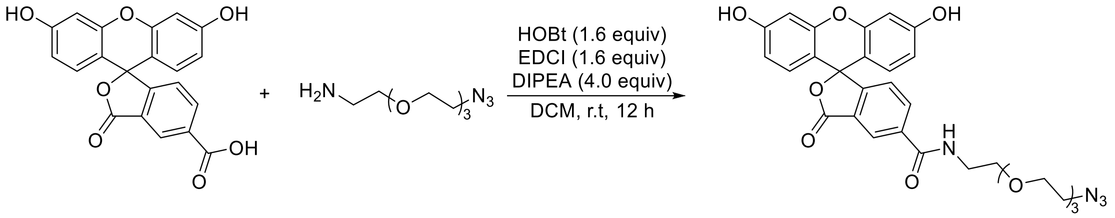

FAM-PEG$_3$-N$_3$

In a 50 mL round-bottom flask, FAM (0.6 mmol, 1.2 equiv) was dissolved in DCM (5.0 mL), HOBt (0.8 mmol, 1.6 equiv) and EDCI (0.8 mmol, 1.6 equiv) were added in turn. The mixture was stirred at room temperature for 30 min, the DCM solution of DIPEA (2.4 mmol, 4.0 equiv) and N_3-PEG$_3$-NH_2 (0.5 mmol, 1.0 equiv) was added into the above mixture at 0 °C. The reaction mixture was warmed to room temperature and stirred overnight. The resulting mixture was extracted with ethyl acetate and washed by citric acid solution (× 3), saturated $NaHCO_3$ solution (× 3) and brine (× 3). The organic layer was dried over Na_2SO_4 and concentrated in vacuo. The residue was purified by recycling preprative HPLC system (Japan Analytical Industry Co.,Ltd LaboACE LC-5060 Plus II, ethyl acetate) to afford the desired product **FAM-PEG$_3$-N$_3$**.

***N*-(2-(2-(2-(2-azidoethoxy)ethoxy)ethoxy)ethyl)-3',6'-dihydroxy-3-oxo-3H-spiro[isobenzofuran-1,9'-xanthene]-5-carboxamide (FAM-PEG$_3$-N$_3$)** Yield: 90%. **^{1}H NMR** (600 MHz, CD_3CN) δ 8.34 (s, 1H), 8.15 (d, J = 6.0 Hz, 1H), 7.27 (d, J = 6.0 Hz, 1H), 6.71 (d, J = 3.0 Hz, 2H), 6.65 (d, J = 8.4 Hz, 2H), 6.55 (dd, J = 3.0 Hz, 6.0 Hz, 2H), 3.64 (t, J = 6.0 Hz, 2H), 3.62-3.56 (m, 10H), 3.32 (t, J = 4.8 Hz, 2H), 1.94 (t, J = 2.4 Hz, 2H). **^{13}C NMR** (150 MHz, CD_3CN) δ 169.02, 166.08, 158.85, 155.65, 152.94, 137.35, 134.82, 130.38, 129.94, 124.80, 123.97, 113.03, 110.89, 103.10, 80.16, 70.77, 70.75, 70.57, 70.07, 69.57, 51.06, 40.28.

3. Optimization of reaction conditions

3.1 Screen the reaction conditions for 6-azauridine modification of peptide

Yields were determined by integrated areas of HPLC peaks (at 220 nm) with coumarin as an internal standard. **3aa** (0.05 mmol) was mixed with coumarin (0.05 mmol) and dissolved in 1 mL 50% acetonitrile in water, the injection volumes were 10, 20, 30, 40 and 50 µL, standard curve for yield was prepared on a UNIMICRO EasySep-3030® using Waters Symmetry® C18 Column (100 Å, 5 µm, 4.6 mm × 250 mm). Linear gradients were used using A: MeCN (0.1% CF_3COOH) and B: H_2O (0.1% CF_3COOH). Taking the HPLC peak area of **3aa** as the abscissa and the HPLC peak area of coumarin as the ordinate, a straight line was obtained with a slope of 0.5403 (R square = 0.9942), as shown in **Figure S1**.

Table S1. The HPLC elution gradient for content internal standard curves

Time (min)	A (%)	B (%)	Flow (mL/min)
0.00	10.0	90.0	1.0
20.00	75.0	25.0	1.0
21.00	95.0	5.0	1.0
25.00	95.0	5.0	1.0
25.10	10.0	90.0	1.0
32.00	10.0	90.0	1.0

The yield was determined using HPLC peak area (at 220 nm) in comparison with the absorption of the internal standard by the equation below:

$$Yield(\%) = \frac{x}{(y-b)/k} \times 100$$

x, y refers to the following HPLC peak areas (λ = 220 nm), respectively:

x: HPLC peak area of **3aa** in the mixture

y: HPLC peak area of coumarin in the mixture

k: slope = 0.5403

b: intercept = 233.09

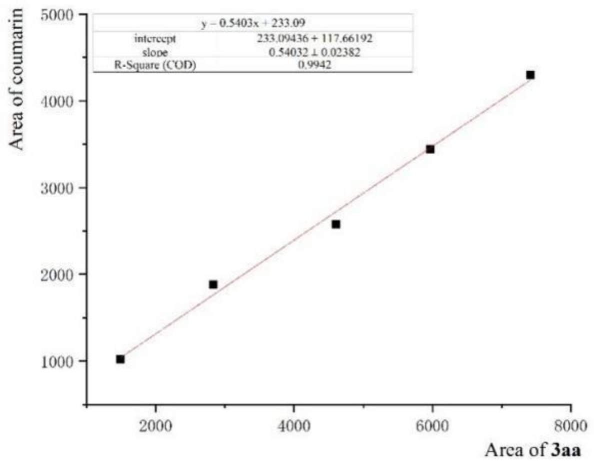

Figure S1. Internal standard curves for the determination of **3aa** content.

Table S2. Screen the reaction conditions for 6-azauridine modification of peptide

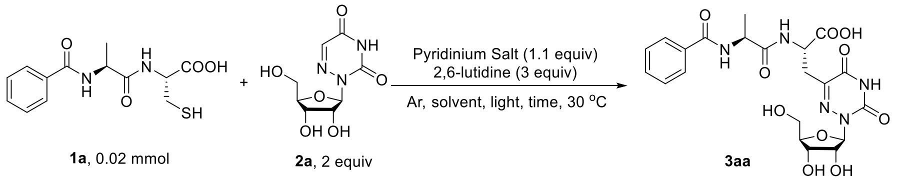

Entry	Pyridinium Salt	Solvent	Light	Time	Yield[a]
1	**I**	0.2 M PBS buffer, pH = 8.0 (9% v/v MeCN) (1 mL)	400 nm	10 h	61%
2	**II**	0.2 M PBS buffer, pH = 8.0 (9% v/v MeCN) (1 mL)	400 nm	10 h	55%
3	**III**	0.2 M PBS buffer, pH = 8.0 (9% v/v MeCN) (1 mL)	400 nm	10 h	15%
4	**IV**	0.2 M PBS buffer, pH = 8.0 (9% v/v MeCN) (1 mL)	400 nm	10 h	47%
5	**V**	0.2 M PBS buffer, pH = 8.0 (9% v/v MeCN) (1 mL)	400 nm	10 h	N.R[b]
6	**VI**	0.2 M PBS buffer, pH = 8.0 (9% v/v MeCN) (1 mL)	400 nm	10 h	25%
7	**VII**	0.2 M PBS buffer, pH = 8.0 (9% v/v MeCN) (1 mL)	400 nm	10 h	90%
8	**VII**	1 M Tris-HCl buffer, pH = 8.0 (5% v/v MeCN) (1 mL)	400 nm	10 h	78%
9	**VII**	1 M Tris-HCl buffer, pH = 8.0 (5% v/v MeCN) (1 mL)	390 nm	10 h	83%
10	**VII**	1 M Tris-HCl buffer, pH = 8.0 (10% v/v MeCN) (1 mL)	390 nm	10 h	75%
11	**VII**	1 M Tris-HCl buffer, pH = 8.0 (5% v/v MeCN) (0.4 mL)	390 nm	10 h	46%
12	**VII**	1 M Tris-HCl buffer, pH = 8.0 (5% v/v MeCN) (1.6 mL)	390 nm	10 h	46%
13	**VII**	0.2 M PBS buffer, pH = 8.0 (9% v/v MeCN) (1 mL)	400 nm	5 h	77%

Pyridinium Salt

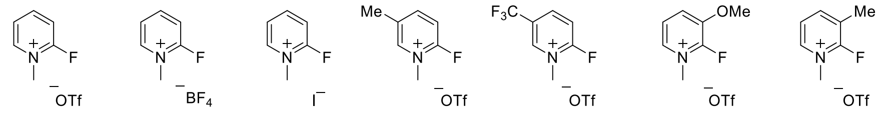

I **II** **III** **IV** **V** **VI** **VII**

[a]Yields were determined by integrated areas of HPLC peaks (at 220 nm) with coumarin as an internal standard. [b]N.R. = No Reaction.

Reaction under the optimized conditions as a representative example (Table S2, entry 7):

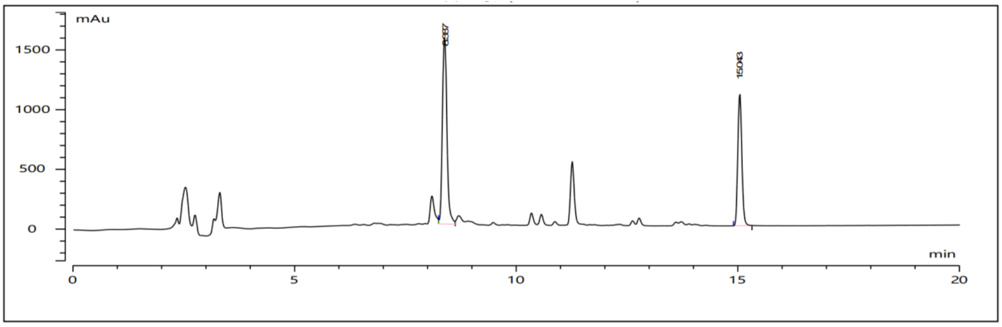

	Name	Ret.Time (min)	Area (mAu*s)	Rel.Area (%)	Height (mAu)
1	**3aa**	8.387	11248.036	61.555	1565.328
2	coumarin	15.043	7025.197	38.445	1112.320

Figure S2. HPLC of entry 7 (Analyzing the purities was carried out on a UNIMICRO EasySep®-3030 using a Waters Symmetry® C18 Column (100 Å, 5 µm, 4.6 mm × 250 mm)).

$$Yield(\%) = \frac{x}{(y-b)/k} \times 100 = \frac{11248.036}{(7025.197\text{-}233.09)/0.5403} = 90\%$$

Table S3. Competitive experiments

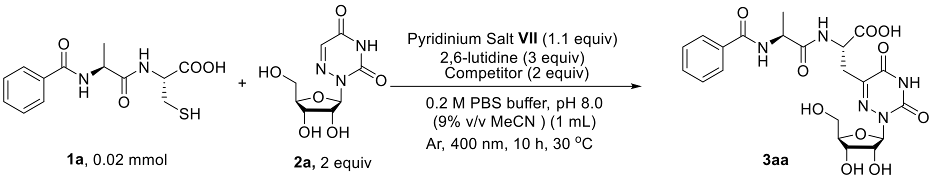

Entry	Competitors	Yield (%)[a]
1	BocNH-Lys-OH	84%
2	BocNH-Tyr-OH	84%
3	BocNH-His-OH	87%
4	BocNH-Trp-OH	83%
5	BocNH-Ser-OH	84%
6	BocNH-Arg-OH	83%
7	BocNH-Glu-OH	85%
8	Thymidine (T)	85%
9	Uridine (U)	83%
10	Cytidine (C)	83%
11	Guanosine (G)	77%
12	Adenosine (A)	78%

[a]Yields were determined by integrated areas of HPLC peaks (at 220 nm) with coumarin as an internal standard.

Table S4. Control experiments

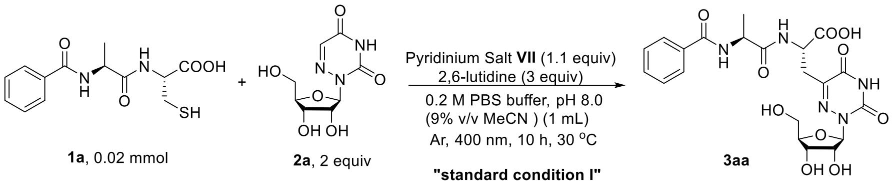

Entry	Changes from the "standard condition I"	Yield (%)[a]
1	in the dark, room temperature	N.R.[b]
2	in the dark, 60 °C	N.R.[b]
3	without Pyridinium Salt	N.R.[b]
4	air	32%
5	without 2,6-lutidine	70%
6	with TEMPO (4 equiv)	trace

[a]Yields were determined by integrated areas of HPLC peaks (at 220 nm) with coumarin as an internal standard. [b]N.R. = No Reaction

3.2 Screen the reaction conditions of *L*-alanyl radical with radical acceptor

After the reaction completed, the aqueous phase was extracted with DCM for yield determination. Yields were determined by integrated areas of HPLC peaks (at 220 nm) with coumarin as an internal standard. **5ba** (0.01 mmol) was mixed with coumarin (0.01 mmol) and dissolved 600 μL in 50% aqueous solution of acetonitrile. Taking 30, 60, 90, 120, and 150 μL from the solution successively, and added 500 μL of 50% acetonitrile in water, the injection volume of 50 μL, standard curve for yield was prepared on a UNIMICRO EasySep-3030® using Globalsil® C18-AP column (120 Å, 5 μm, 4.6 mm × 250 mm). Linear gradients were used using A: MeCN (0.1% CF_3COOH) and B: H_2O (0.1% CF_3COOH). Taking the HPLC peak area of **5ba** as the abscissa and the HPLC peak area of coumarin as the ordinate, a straight line was obtained with a slope of 5.8961 (R square = 0.9915), as shown in **Figure S3**.

Table S5. The HPLC elution gradient for content internal standard curves

Time (min)	A (%)	B (%)	Flow (mL/min)
0.00	5.0	95.0	1.0
2.00	5.0	95.0	1.0
25.00	50.0	50.0	1.0
25.10	95.0	5.0	1.0
32.00	95.0	5.0	1.0
32.10	5.0	95.0	1.0
38.0	5.0	95.0	1.0

The yield was determined using HPLC peak area in comparison with the absorption of the internal standard by the equation below:

$$Yield(\%) = \frac{x}{(y-b)/k} \times 100$$

x, y refers to the following HPLC peak areas (λ= 220 nm), respectively:

x: HPLC peak area of **5ba** in the mixture

y: HPLC peak area of coumarin in the mixture

k: slope = 5.8961

b: intercept = 1229.1876

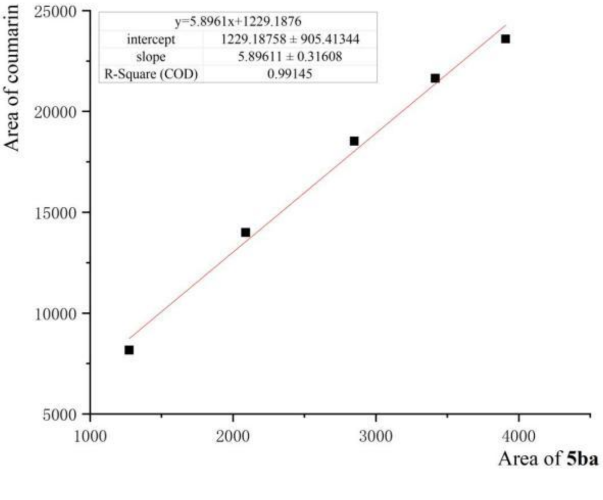

Figure S3. Internal standard curves for the determination of **5ba** content.

Table S6. Screen the reaction conditions for trapping *L*-alanyl radical with radical acceptor

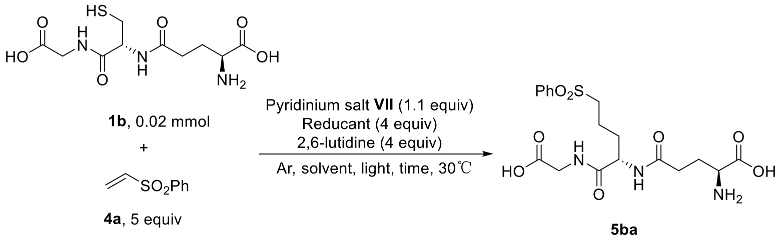

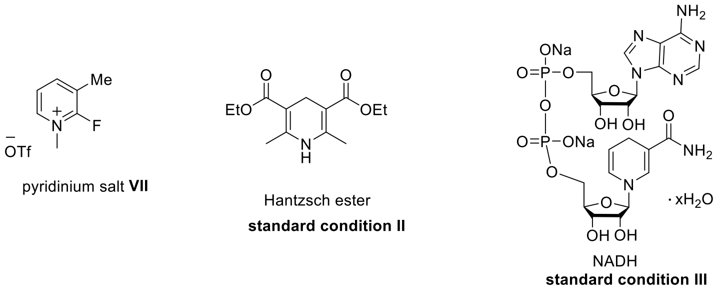

Entry	Reducant	Solvent	Light	Time	Yield(%)[a]
1	Hantzsch ester	1 M Tris-HCl buffer, pH 7.2 (20% v/v TFE) (0.5 mL)	390 nm	10 h	93%
2	Hantzsch ester	1 M Tris-HCl buffer, pH 7.2 (20% v/v TFE) (0.5 mL)	400nm	10 h	90%
3	Hantzsch ester	1 M Tris-HCl buffer, pH 7.2 (20% v/v TFE) (0.5 mL)	410-420 nm	10 h	81%
4	Hantzsch ester	1 M Tris-HCl buffer, pH 7.2 (20% v/v TFE) (0.5 mL)	420-430 nm	10 h	76%
5	Hantzsch ester	1 M Tris-HCl buffer, pH 7.2 (20% v/v TFE) (1.0 mL)	400 nm	10 h	70%
6	Hantzsch ester	1 M Tris-HCl buffer, pH 7.2 (20% v/v TFE) (0.25 mL)	400 nm	10 h	84%
7	Hantzsch ester	1 M Tris-HCl buffer, pH 7.2 (20% v/v TFE) (0.5 mL)	400 nm	0.5 h	68%
8	Hantzsch ester	1 M Tris-HCl buffer, pH 7.2 (20% v/v TFE) (0.5 mL)	400 nm	1.5 h	82%
9	Hantzsch ester	1 M Tris-HCl buffer, pH 7.2 (20% v/v TFE) (0.5 mL)	400 nm	3 h	90%
10	NADH	1 M Tris-HCl buffer, pH 7.2 (20% v/v TFE) (0.5 mL)	400 nm	10 h	49%
11	NADH	0.1 M PBS buffer, pH 7.4 (20% v/v TFE) (0.5 mL)	400 nm	10 h	41%
12	NADH	0.1 M PBS buffer, pH 6.5 (20% v/v TFE) (0.5 mL)	400 nm	10 h	58%
13	NADH	0.1 M PBS buffer, pH 6.5 (0.5 mL)	400 nm	10 h	34%
14	NADH	0.1 M PBS buffer, pH 6.5 (20% v/v TFE) (0.5 mL)	410-420 nm	10 h	70%
15	NADH	0.1 M PBS buffer, pH 6.5 (20% v/v TFE) (0.5 mL)	420-430 nm	10 h	71%
16[b]	NADH	0.1 M PBS buffer, pH 6.5 (20% v/v TFE) (0.5 mL)	420-430 nm	10 h	85%
17[b]	NADH	0.1 M PBS buffer, pH 6.5 (20% v/v TFE) (0.5 mL)	420-430 nm	0.5 h	42%
18[b]	NADH	0.1 M PBS buffer, pH 6.5 (20% v/v TFE) (0.5 mL)	420-430 nm	1.5 h	74%
19[b]	NADH	0.1 M PBS buffer, pH 6.5 (20% v/v TFE) (0.5 mL)	420-430 nm	5 h	86%

[a]Yields were determined by integrated areas of HPLC peaks (at 220 nm) with coumarin as an internal standard. [b]NADH (5 equiv).

Reaction under the optimized conditions as a representative example (Table S6, entry 9 and entry 19):

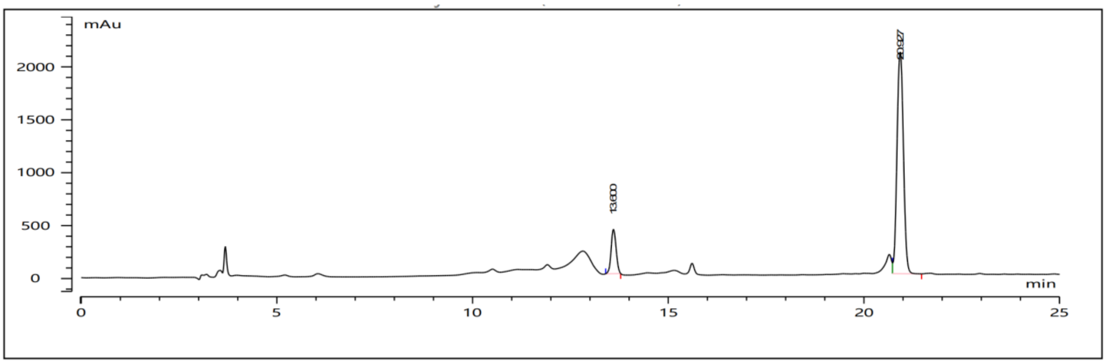

	Name	Ret.Time (min)	Area (mAu*s)	Rel.Area (%)	Height (mAu)
1	**5ba**	13.600	3710.422	13.135	420.182
2	coumarin	20.927	24536.982	86.865	2100.267

Figure S4. HPLC of entry 9 (Analyzing was carried out on a UNIMICRO EasySep®-3030 using a Globalsil® C18-AP column (120 Å, 5 μm, 4.6 mm × 250 mm)).

$$Yield(\%) = \frac{x}{(y-b)/k} \times 100 = \frac{3710.422}{(24536.982\text{-}1229.1876)/5.8961} = 90\%$$

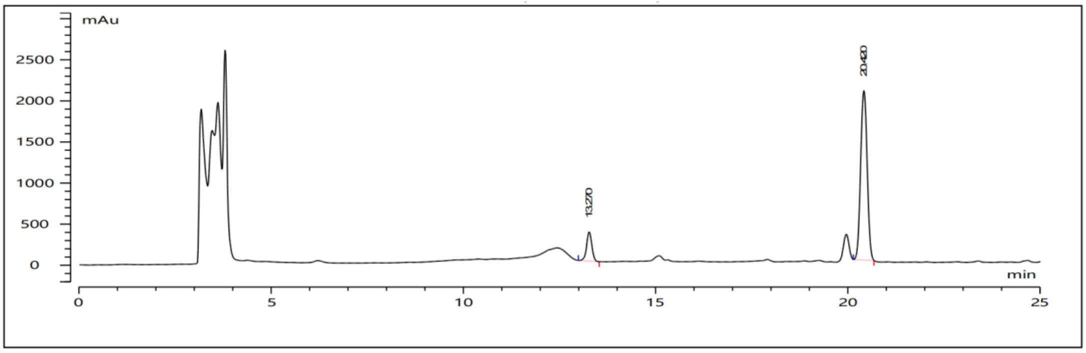

	Name	Ret.Time (min)	Area (mAu*s)	Rel.Area (%)	Height (mAu)
1	**5ba**	13.270	3358.585	12.130	355.997
2	coumarin	20.420	24329.609	87.870	2063.138

Figure S5. HPLC of entry 19 (Analyzing was carried out on a UNIMICRO EasySep®-3030 using a Globalsil® C18-AP column (120 Å, 5 μm, 4.6 mm × 250 mm)).

$$Yield(\%) = \frac{x}{(y-b)/k} \times 100 = \frac{3358.585}{(24329.609 - 1229.1876)/5.8961} = 86\%$$

Table S7. Control experiments of standard condition **II**

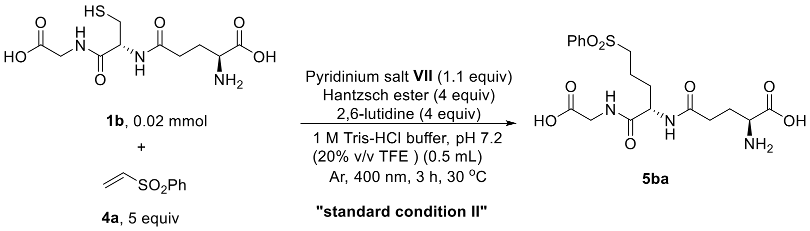

Entry	Changes from the "standard condition II"	Yield (%)[a]
1	in the dark, room temperature	N.R.[b]
2	in the dark, 60 °C	N.R.[b]
3	without pyridinium salt	N.R.[b]
4	without hantzsch eater	N.R.[b]
5	air	74%
6	without 2,6-lutidine	61%
7	with TEMPO (4 equiv)	trace

[a]Yields were determined by integrated areas of HPLC peaks (at 220 nm) with coumarin as an internal standard. [b]N.R. = No Reaction

Table S8. Control experiments of standard condition **III**

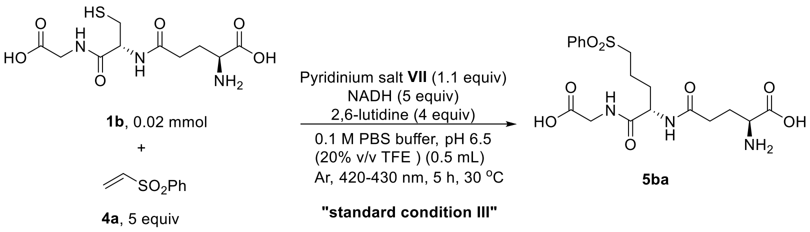

Entry	Changes from the "standard condition III"	Yield (%)[a]
1	in the dark, room temperature	N.R.[b]
2	in the dark, 60 °C	N.R.[b]
3	without pyridinium salt	N.R.[b]
4	without NADH	N.R.[b]
5	air	65%
6	without 2,6-lutidine	53%
7	with TEMPO (4 equiv)	trace

[a]Yields were determined by integrated areas of HPLC peaks (at 220 nm) with coumarin as an internal standard. [b]N.R. = No Reaction

3.3 General Procedure 4 (GP4) of condition I

To an oven-dried 10 mL quartz test tube with a stirring bar was added peptide (5 μmol), **2a-2f** (2 equiv), or **2h-2p** (2 equiv), or **2g** (1 equiv), pyridinium salt **VII** (1.1 equiv) and 2,6-lutidine (3 equiv). Then, air was withdrawn and backfilled with Ar (three times). 250 μL PBS buffer (0.2 M, pH 8.0, 9% v/v MeCN) was added. The mixture was transferred to a violet LED photoreactor (100-W, 400 nm), where it was irradiated for 10 h. Meanwhile, keep the ambient temperature at 30 °C. After the reaction was completed, the product was purified by the semi-HPLC (Hanbon Sci.& Tech.) equipped with the Nucifera C18U column. Linear gradients using A: MeCN (0.1% CF_3COOH) and B: H_2O (0.1% CF_3COOH).

3.4 General Procedure 5 (GP5) of condition II

To an oven-dried 10 mL quartz test tube with a stirring bar was added peptide (0.02 mmol), **4a-4g** (5 equiv), pyridinium salt **VII** (1.1 equiv), Hantzsch ester (4 equiv) and 2,6-lutidine (4 equiv). Then, air was withdrawn and backfilled with Ar (three times). 0.5 mL Tris-HCl buffer (1 M, pH 7.2, 20% v/v TFE) was added. The mixture was transferred to a violet LED photoreactor (100-W, 400 nm), where it was irradiated for 3 h. Meanwhile, keep the ambient temperature at 30°C. After the reaction was completed, the product was purified by the semi-HPLC (Hanbon Sci.& Tech.) equipped with the Nucifera C18U column. Linear gradients using A: MeCN (0.1% CF_3COOH) and B: H_2O (0.1% CF_3COOH).

3.5 General Procedure 6 (GP6) of condition III

To an oven-dried 10 mL quartz test tube with a stirring bar was added peptide (5 μmol), **4a** or **4c** (5 equiv) or no radical acceptor, pyridinium salt **VII** (1.1 equiv), NADH (5 equiv) and 2,6-lutidine (4 equiv). Then, air was withdrawn and backfilled with Ar (three times). 125 μL PBS buffer (0.1 M, pH 6.5, 20% v/v TFE) was added. The mixture was transferred to a violet LED photoreactor (24-W, 420-430 nm), where it was irradiated for 5 h. Meanwhile, keep the ambient temperature at 30°C. After the reaction was completed, the product was purified by the semi-HPLC (Hanbon Sci.& Tech.) equipped with the Nucifera C18U column. Linear gradients using A: MeCN (0.1% CF_3COOH) and B: H_2O (0.1% CF_3COOH).

3.6 Picture of the reaction photo set-up (400 nm)

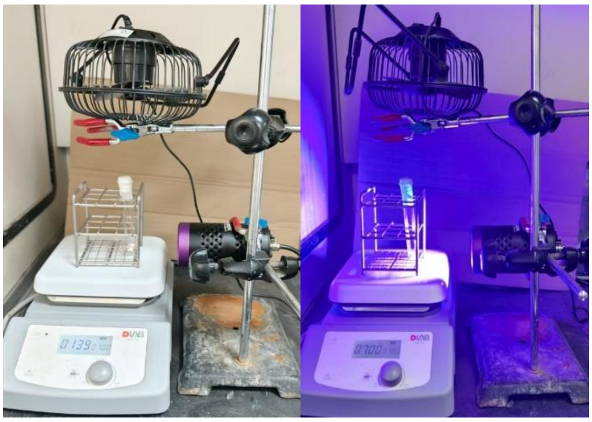

The photochemical reactions were conducted using a custom-built photoreactor equipped with 400 nm LEDs (Heyseek, HSL40-400 nm). The light source was positioned at a distance of approximately 8-10 cm from the reaction vials to ensure uniform irradiation.

The nature of the photoreactor is show as below:

Heyseek LED Spectra Test Report

Product ID

Product Model: UVC LED bulb(HSL40-400 nm)

Ambient Temperature: 20℃

Tester: admin

Manufacturer:

Ambient Humidity: 65%

Test Date: 2025-02-28,11:43:43

Parameter list

Parameter name	Parameter values	Parameter name	parameter values	Parameter name	parameter values	Parameter name	parameter values
Luminous Flux (lm)	1.79	Color Gamut Index Rg	-3.92	Flicker Percentage (%)	0.0	PPF_UV(umol/s)	4.928
Luminous efficacy (lm/W)	0.68	Dominant Wavelength (nm)	432.40	Flicker Index	0.00	PPF_B(umol/s)	2.601
CCT (K)	100000	Chromaticity Purity (%)	99.3	Φeb(mW)	0.089	PPF_G(umol/s)	0.000
Black Body Deviation Duv	-0.12127	FWHM (nm)	12.7	Φey(mW)	0.000	PPF_R(umol/s)	0.000
Coordinates x,y	0.1692,0.0100	Peak Wavelength (nm)	399.3	Φer(mW)	0.000	PPF_FR(umol/s)	0.000
Coordinates u,v	0.2433,0.0216	Center Wavelength (nm)	399.1	Φeuv(mW/cm²)	0.150	PPF_IR(umol/s)	0.000
Coordinates u',v'	0.2433,0.0323	Centroid Wavelength (nm)	398.8	Φefr(mW/cm²)	0.000	Kppfv(umol/s/klm)	1449.836
Color Tolerance	100.00	Luminous Flux Color Ratio (RGB)	0.0,20.0,80.0	Φeir(mW/cm²)	0.000	YPF(umol/s)	4.854
Color Rendering Index Ra	-71.1	CIE1931 X	44.456	Φec(mW/cm²)	0.000	Φep(mW)	0.146
Radiant Flux (mW)	2258.217	CIE1931 Y	2.627	Φerb Ratio	0.000	Integration Time (ms)	1
Blue Light Hazard Irradiationm W/㎡	0.04	CIE1931 Z	215.677	PAR(mW)	0.226	Peak Signal	54999
Bek (uW/lm)	198.56	TLCI-2012	0	Chlorophyll A(mW/cm²)	0.000	dark signal	2052
Photopic to Scotopic Ratio S/P	34.476	Flicker Frequency (Hz)	0.00	Chlorophyll B(mW/cm²)	0.000	Compensation Level	2892
Realism Rf	0.00	Fluctuation Depth (%)	0.0	PPF(umol/s)	2.601	Voltage (V)	3.780

Spectral Graph

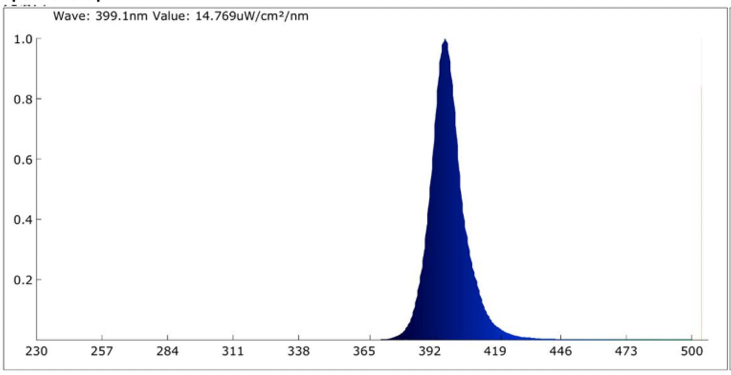

CIE 1931

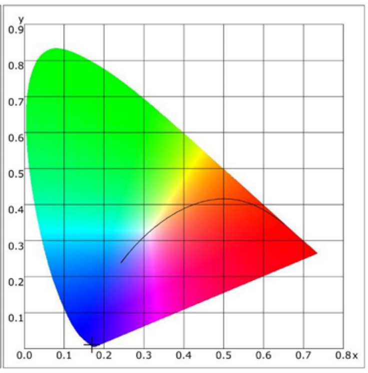

Color Rendering Index

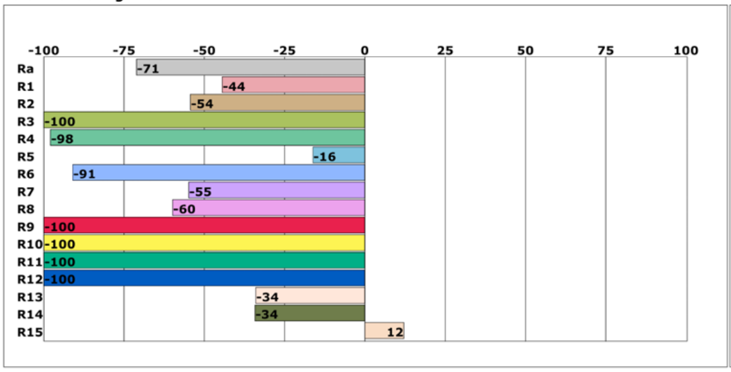

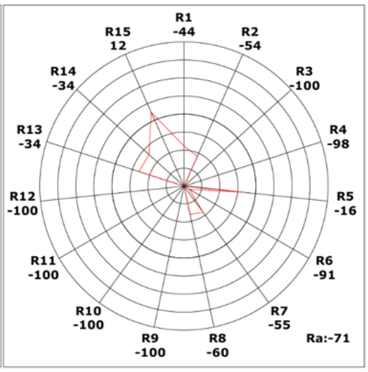

Instrument status

Instrument Model: OHSP-350M

Dwell Time: 0.555ms

Instrument Serial Number: 201908253

Peak Signal: 54999

Wavelength Range: 230-500nm

Dark Voltage: 2052

4. Characterization of products

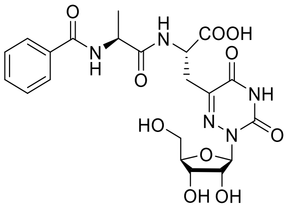

3aa

(*S*)-2-((*S*)-2-benzamidopropanamido)-3-(2-((2*R*,3*R*,4*S*,5*R*)-3,4-dihydroxy-5-(hydroxymethyl)-tetrahydrofuran-2-yl)-3,5-dioxo-2,3,4,5-tetrahydro-1,2,4-triazin-6-yl)propanoic acid (3aa) yield: 75 %, 23.7 mg. **^{1}H NMR** (400 MHz, D_2O) δ 7.59 (t, *J* = 8.0 Hz, 2H), 7.44 (t, *J* = 8.0 Hz, 1H), 7.35-7.32 (m, 2H), 5.82 (d, *J* = 3.2 Hz, 1H), 4.75-4.73 (m, 1H), 4.35-4.31 (m, 2H), 4.15-4.12 (m, 1H), 3.87-3.83 (m, 1H), 3.62-3.58 (m, 1H), 3.47-3.43 (m, 1H), 3.18-3.13 (m, 1H), 2.91-2.85 (m, 1H), 1.28 (d, *J* = 8.0 Hz, 3H). **^{13}C NMR** (101 MHz, D_2O) δ 174.90, 173.56, 170.43, 157.22, 149.40, 143.79, 132.63, 132.30, 128.68, 127.12, 89.59, 83.72, 72.63, 70.02, 61.37, 50.03, 49.71, 30.97, 16.10. **HRMS (ESI)** $C_{21}H_{25}N_5NaO_{10}$ $[M+Na]^+$ calcd: 530.1503, found: 530.1494.

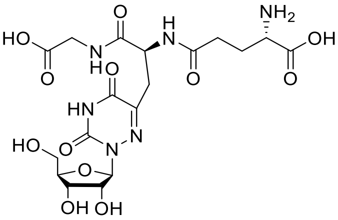

3ba

***N*^{5}-((*S*)-1-((carboxymethyl)amino)-3-(2-((2*R*,3*R*,4*S*,5*R*)-3,4-dihydroxy-5-(hydroxymethyl)tetrahydrofuran-2-yl)-3,5-dioxo-2,3,4,5-tetrahydro-1,2,4-triazin-6-yl)-1-oxopropan-2-yl)-*L*-glutamine (3ba)** yield: 50 %, 15.5 mg. **^{1}H NMR** (400 MHz, D_2O) δ 5.96 (d, *J* = 4.0 Hz, 1H), 4.72-4.70 (m, 1H), 4.40-4.37 (m, 1H), 4.20-4.17 (m, 1H), 3.96-3.93 (m, 1H), 3.88 (s, 2H), 3.87-3.81 (m, 1H), 3.70-3.66 (m, 1H), 3.53-3.48 (m, 1H), 3.12-3.08 (m, 1H), 2.87-2.81 (m, 1H), 2.38 (t, *J* = 8.0 Hz, 2H), 2.03 (t, *J* = 8.0 Hz, 2H). **^{13}C NMR** (101 MHz, D_2O) δ 174.15, 172.85, 172.55, 172.25, 157.28, 149.56, 143.78, 89.59, 83.31, 72.66, 70.10, 61.46, 52.63, 50.66, 40.99, 31.29, 30.86, 25.46. **HRMS (ESI)** $C_{18}H_{26}N_6NaO_{12}$ $[M+Na]^+$ calcd: 541.1506, found: 541.1501.

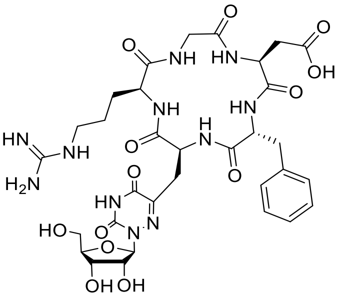

3ma

2-((2*S*,5*R*,8*S*,11*S*)-5-benzyl-8-((2-((2*R*,3*R*,4*S*,5*R*)-3,4-dihydroxy-5-(hydroxymethyl)tetrahydrofuran-2-yl)-3,5-dioxo-2,3,4,5-tetrahydro-1,2,4-triazin-6-yl)methyl)-11-(3-guanidinopropyl)-3,6,9,12,15-pentaoxo-1,4,7,10,13-pentaazacyclopentadecan-2-yl)acetic acid (3ma) yield: 67%, 31.7 mg. **^{1}H NMR** (400 MHz, D_2O) δ 7.13-7.10 (m, 2H), 7.07-7.01 (m, 3H), 5.91 (d, *J* = 2.8 Hz, 1H), 4.60 (t, *J* = 8.0 Hz, 1H), 4.41-4.37 (m, 2H), 4.25-4.23 (m, 1H), 4.19-4.14 (m, 2H), 4.05 (d, *J* =

15.2 Hz, 1H), 3.93-3.90 (m, 1H), 3.66 (dd, J = 3.2 Hz, 9.2 Hz, 1H), 3.48 (q, J = 6.0 Hz, 1H), 3.35 (d, J = 15.6 Hz, 1H), 3.05-3.01 (m, 2H), 2.87-2.81 (m, 4H), 2.78-2.72 (m, 2H), 1.76-1.67 (m, 1H), 1.59-1.49 (m, 1H), 1.38-1.33 (m, 2H). **^{13}C NMR** (101 MHz, D_2O) δ 174.17, 172.78, 172.53, 172.30, 171.32, 171.27, 156.69, 156.57, 149.22, 142.63, 135.77, 128.99, 128.42, 126.88, 89.70, 83.62, 72.99, 70.22, 61.55, 55.38, 52.77, 52.42, 49.50, 43.38, 40.40, 36.43, 34.40, 31.16, 26.98, 24.36. **HRMS (ESI)** $C_{32}H_{44}N_{11}O_{13}$ $[M+H]^+$ calcd: 790.3148, found: 790.3115.

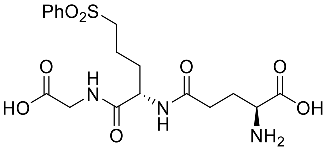

5ba

***N*^{5}-((*S*)-1-((carboxymethyl)amino)-1-oxo-5-(phenylsulfonyl)pentan-2-yl)-*L*-glutamine (5ba)** yield: 73%, 19.5 mg. **^{1}H NMR** (400 MHz, D_2O) δ 7.80-7.78 (m, 2H), 7.68-7.65 (m, 1H), 7.58-7.54 (m, 2H), 4.15-4.12 (m, 1H), 3.89-3.76 (m, 3H), 3.30-3.27 (m, 2H), 2.35 (t, J = 8.0 Hz, 2H), 2.05-1.99 (m, 2H), 1.75-1.72 (m, 1H), 1.65-1.58 (m, 3H). **^{13}C NMR** (101M Hz, D_2O) δ 174.18, 173.84, 172.77, 171.77, 136.42, 134.74, 129.62, 127.74, 54.14, 52.83, 52.35, 40.87, 30.74, 29.10, 25.44, 18.66. **HRMS (ESI)** $C_{18}H_{26}N_3O_8S$ $[M+H]^+$ calcd: 444.1456, found: 444.1435.

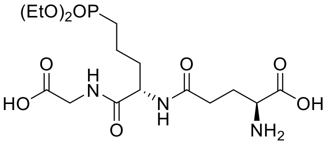

5bb

***N*^{5}-((*S*)-1-((carboxymethyl)amino)-5-(diethoxyphosphoryl)-1-oxopentan-2-yl)-*L*-glutamine (5bb)** yield: 52%, 13.7mg. **^{1}H NMR** (400 MHz, D_2O) δ 4.23-4.19 (m, 1H), 4.04-3.86 (m, 7H), 2.47-2.40 (m, 2H), 2.14-2.06 (m, 2H), 1.85-1.79 (m, 3H), 1.77-1.67 (m, 1H), 1.59-1.50 (m, 2H), 1.18 (t, J = 4.0 Hz, 6H). **^{31}P NMR** (162 MHz, D_2O) δ 35.40. **^{13}C NMR** (101 MHz, D_2O) δ 174.41, 174.33, 172.94, 172.03, 63.27 (t, J = 7.0 Hz), 53.27, 52.59, 40.98, 31.45, 31.28, 30.86, 25.56, 23.81, 22.43, 18.02 (d, J = 4.0 Hz), 15.52 (d, J = 6.0 Hz). **HRMS (ESI)** $C_{16}H_{31}N_3O_9P$ $[M+H]^+$ calcd: 440.1798, found: 440.1789.

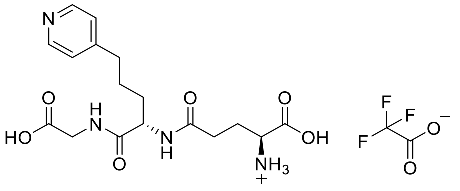

5bc

(*S*)-1-carboxy-4-(((*S*)-1-((carboxymethyl)amino)-1-oxo-5-(pyridin-4-yl)pentan-2-yl)amino)-4-oxobutan-1-aminium 2,2,2-trifluoroacetate (5bc) yield: 35%, 10.4 mg. **^{1}H NMR** (600 MHz, D_2O) δ 8.48 (d, J = 6.6 Hz, 2H), 7.66 (d, J = 6.6 Hz, 2H), 4.21-4.19 (m, 1H), 3.88-3.79 (m, 3H), 2.84 (t, J = 6.6 Hz, 2H), 2.43-2.35 (m, 2H), 2.03 (q, J = 7.2 Hz, 2H), 1.75-1.64 (m, 4H). **^{13}C NMR** (150 MHz, D_2O) δ 174.43, 174.37, 172.99, 172.27, 164.32, 163.12, 162.77, 140.29, 127.11, 53.54, 52.73, 40.96, 34.74, 30.86, 30.23, 25.58, 24.97. **HRMS (ESI)** $C_{17}H_{25}N_4O_6$ $[M]^+$ calcd: 381.1781, found: 381.1769.

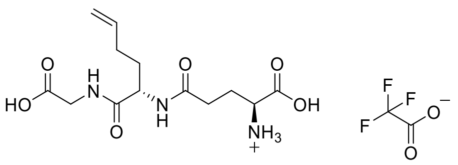

5bd

(*S*)-1-carboxy-4-(((*S*)-1-((carboxymethyl)amino)-1-oxohex-5-en-2-yl)amino)-4-oxobutan-1-aminium 2,2,2-trifluoroacetate (5bd) yield: 26%, 6.8 mg. **^{1}H NMR** (400 MHz, D_2O) δ 5.78-5.68 (m, 1H), 4.98-4.70 (m, 2H), 4.21-4.17 (m, 1H), 3.93-3.82 (m, 3H), 2.51-2.38 (m, 2H), 2.14-1.94 (m, 4H), 1.85-1.65 (m, 2H). **^{13}C NMR** (101 MHz, D_2O) δ 174.84, 174.33, 172.97, 171.83, 163.12, 162.77, 137.28, 115.72, 53.22, 52.40, 40.94, 30.82, 30.01, 29.19, 25.48. **HRMS (ESI)** $C_{13}H_{22}N_3O_6$ $[M]^+$ calcd: 316.1504, found: 316.1503.

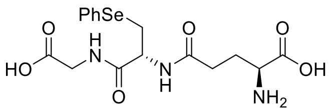

5be

***N*^{5}-((*R*)-1-((carboxymethyl)amino)-1-oxo-3-(phenylselanyl)propan-2-yl)-*L*-glutamine (5be)** yield: 56%, 14.5 mg. **^{1}H NMR** (400 MHz, D_2O) δ 7.46-7.44 (m, 2H), 7.22-7.21 (m, 3H), 7.44 (q, *J* = 4.0 Hz, 1H), 3.77 (t, *J* = 8.0 Hz, 1H), 3.66 (s, 2H), 3.27-3.22 (m, 1H), 3.13-3.08 (m, 1H), 2.29-2.17 (m, 2H), 1.98-1.91 (m, 2H). **^{13}C NMR** (101 MHz, D_2O) δ 174.12, 172.89, 172.45, 172.26, 133.33, 129.43, 127.97, 127.92, 53.69, 52.77, 40.98, 30.87, 27.71, 25.53. **HRMS (ESI)** $C_{16}H_{22}N_3O_6Se$ $[M+H]^+$ calcd: 432.0671, found: 432.0669.

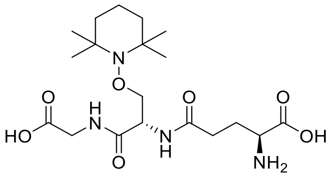

5bf

***N*^{5}-((*S*)-1-((carboxymethyl)amino)-1-oxo-3-((2,2,6,6-tetramethylpiperidin-1-yl)oxy)propan-2-yl)-*L*-glutamine (5bf)** yield: 39%, 10 mg. **^{1}H NMR** (400 MHz, D_2O) δ 4.86 (t, *J* = 6.0 Hz, 1H), 4.54-4.51 (m, 1H), 4.47-4.44 (m, 1H), 3.92 (s, 2H), 3.88-3.86 (m, 1H), 2.49 (t, *J* = 6.0 Hz, 2H), 2.12-2.06 (m, 2H), 1.78-1.67 (m, 5H), 1.53-1.47 (m, 1H), 1.36 (s, 3H), 1.34 (s, 3H), 1.31 (s, 3H), 1.27 (s, 3H). **^{13}C NMR** (101 MHz, D_2O) δ 174.25, 174.23, 172.86, 169.72, 76.64, 71.13, 71.09, 52.68, 51.52, 41.22, 36.54, 36.49, 30.98, 27.44, 27.40, 25.51, 19.41, 14.72. **HRMS (ESI)** $C_{19}H_{35}N_4O_7$ $[M+H]^+$ calcd: 431.2513, found: 431.2500.

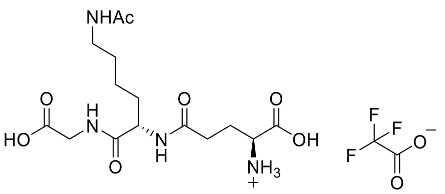

5bg

(*S*)-4-(((*S*)-6-acetamido-1-((carboxymethyl)amino)-1-oxohexan-2-yl)amino)-1-carboxy-4-oxobutan-1-aminium 2,2,2-trifluoroacetate (5bg) yield: 49%, 14.4 mg. **^{1}H NMR** (600 MHz, D_2O) δ 4.17-4.15 (m, 1H), 3.92-3.83 (m, 3H), 3.03 (t, *J* = 7.2 Hz, 2H), 2.47-2.38 (m, 2H), 2.12-2.04 (m, 2H), 1.84 (s, 3H), 1.73-1.66 (m, 1H), 1.63-1.57 (m, 1H), 1.41-1.37 (m, 2H), 1.31-1.22 (m, 2H). **^{13}C NMR** (150 MHz, D_2O) δ 174.79, 174.33, 173.94, 172.95, 171.78, 163.12, 162.77, 53.79, 52.39,

40.92, 39.02, 30.80, 30.51, 27.72, 25.48, 22.22, 21.76. **HRMS (ESI)** $C_{15}H_{27}N_4O_7$ $[M]^+$ calcd: 375.1875, found: 375.1874.

5. Analytical HPLC data of peptides

Analytical data for **1a-1o**, **3aa-3oa**

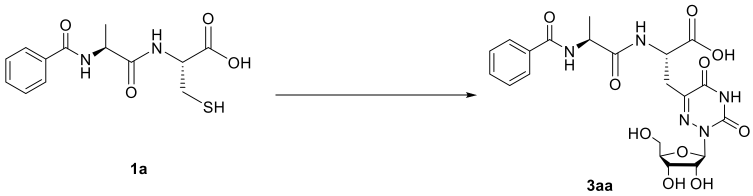

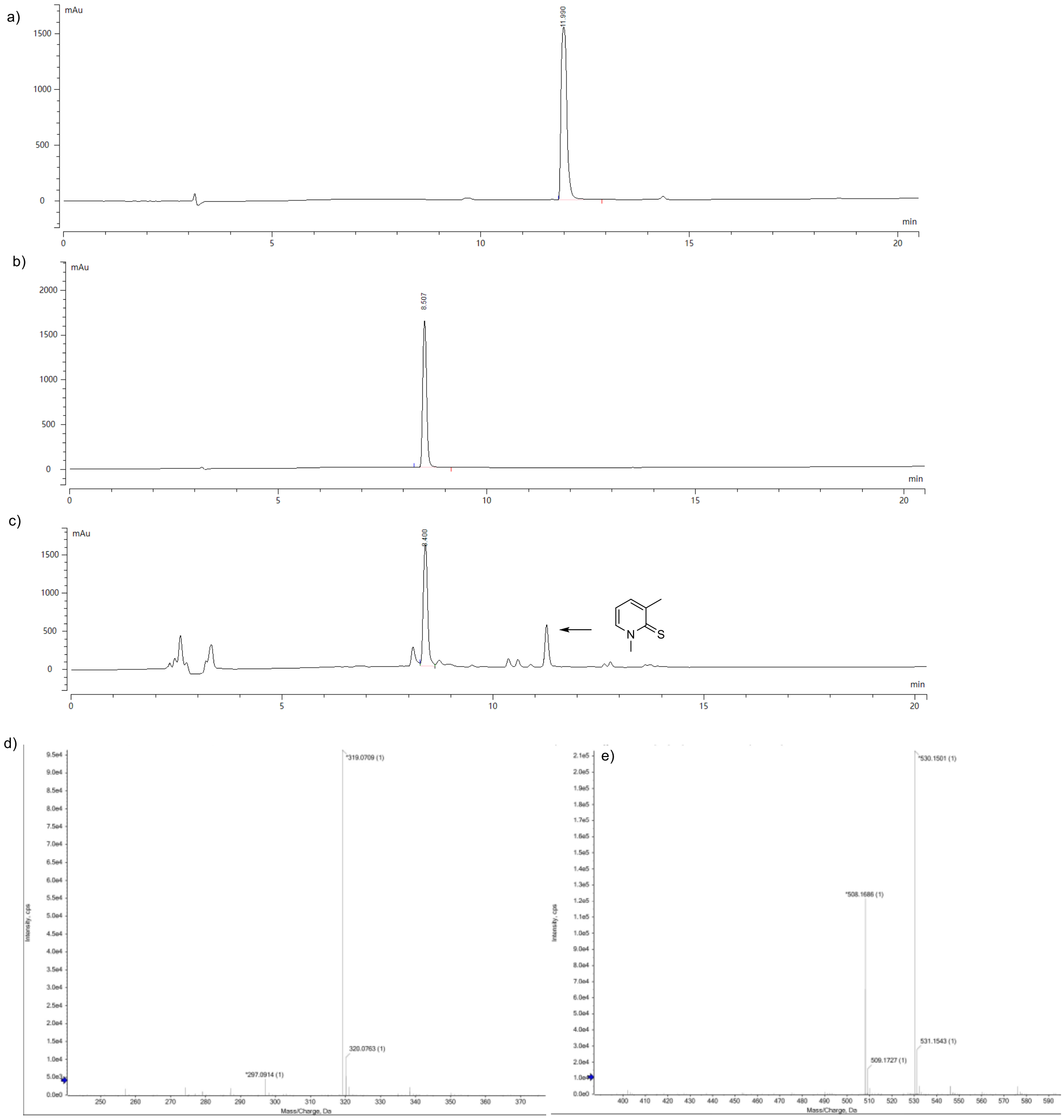

Figure S6. a) Analytical HPLC of **1a**. b) Analytical HPLC of **3aa**. c) Analytical HPLC trace of reaction system for 6-azauridine modification of **1a.** Analyzing the purities was carried out on a UNIMICRO EasySep®-3030 using a Waters Symmetry® C18 Column (100 Å, 5 µm, 4.6 mm × 250 mm). Linear gradients using 10% A/ 90% B to 75% A/ 25% B over 20 min. d) Ms spectrum of **1a** (Calculated Mass $[M+H]^+$: 297.0904; $[M+Na]^+$: 319.0723; Observed Mass $[M+H]^+$: 297.0914; $[M+Na]^+$: 319.0709). e) Ms spectrum of **3aa** (Calculated Mass $[M+H]^+$: 508.1675; $[M+Na]^+$: 530.1494; Observed Mass $[M+H]^+$: 508.1686; $[M+Na]^+$: 530.1501).

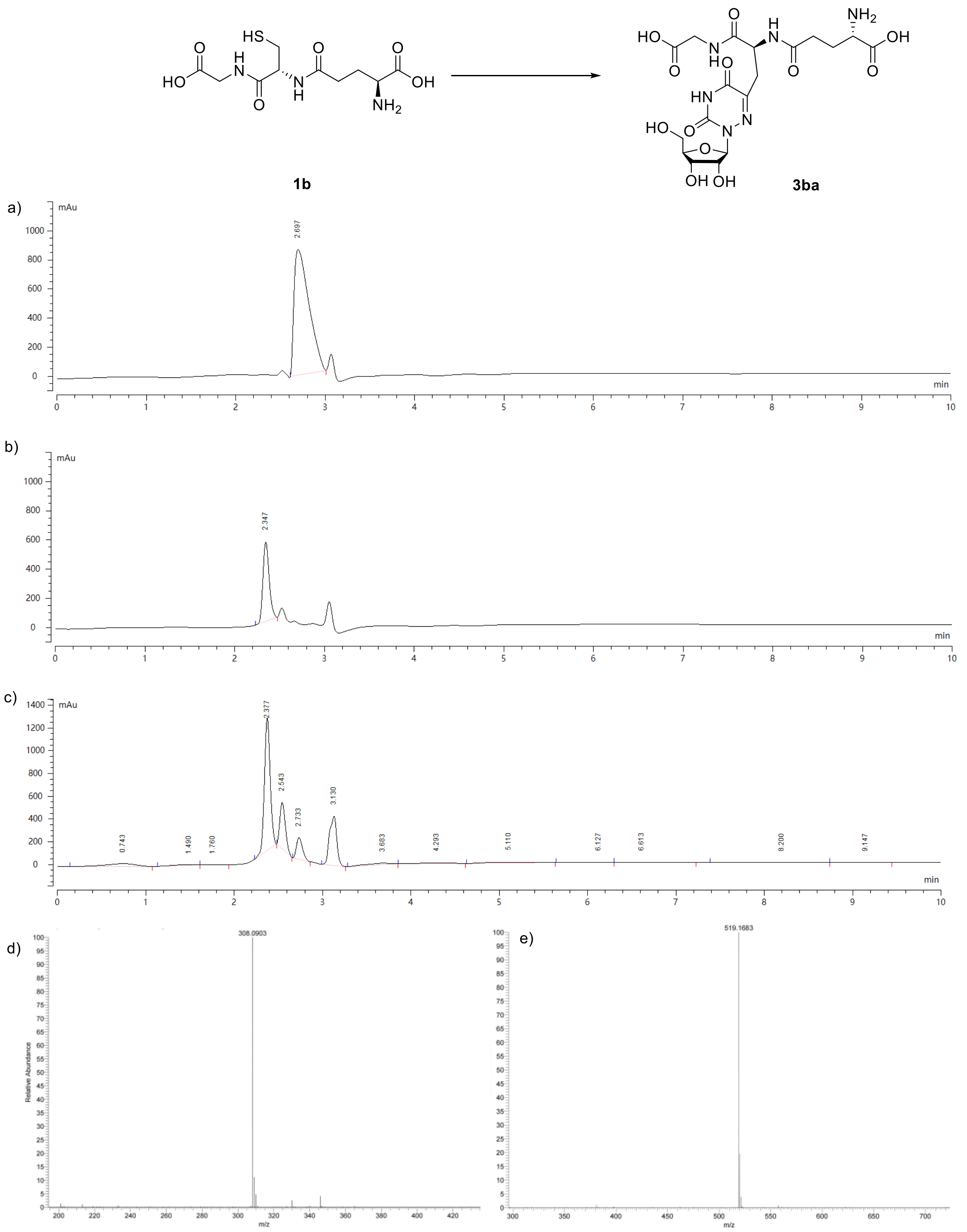

Figure S7. a) Analytical HPLC of GSH **1b**. b) Analytical HPLC of **3ba**. c) Analytical HPLC trace of reaction system for 6-azauridine modification of GSH**.** Analyzing the purities was carried out on a UNIMICRO EasySep®-3030 using a Waters Symmetry® C18 Column (100 Å, 5 μm, 4.6 mm × 250 mm). Isocratic elution using 7% A/ 93% B over 10 min. d) Ms spectrum of GSH **1b** (Calculated Mass $[M+H]^+$: 308.0911; Observed Mass $[M+H]^+$: 308.0903). e) Ms spectrum of **3ba** (Calculated Mass $[M+H]^+$: 519.1682; Observed Mass $[M+H]^+$: 519.1683).

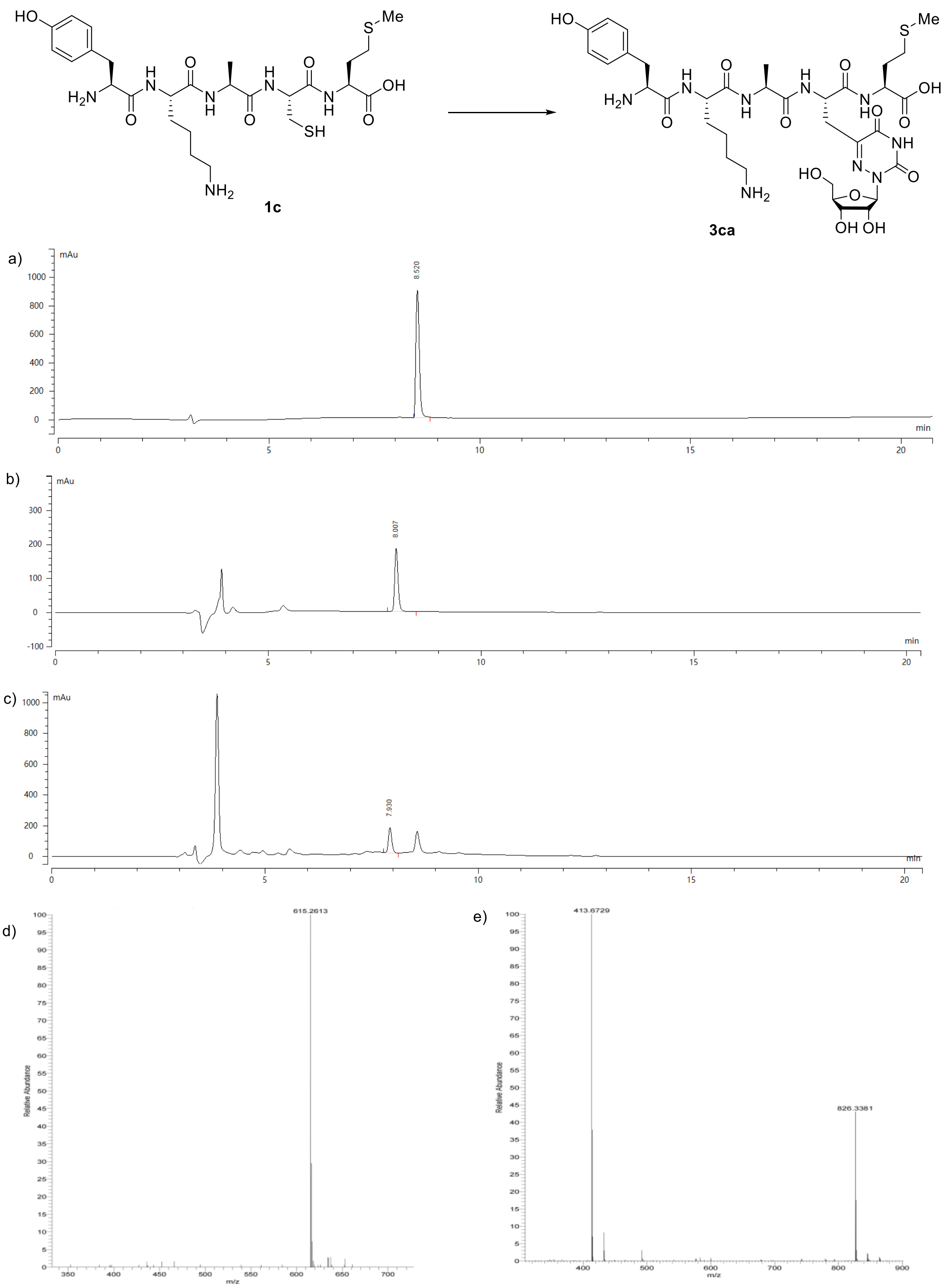

Figure S8. a) Analytical HPLC of peptide **1c**. b) Analytical HPLC of **3ca**. c) Analytical HPLC trace of reaction system for 6-azauridine modification of peptide **1c.** Analyzing the purities was carried out on a UNIMICRO EasySep®-3030 using a Globalsil® C18-AP column (120 Å, 5 μm, 4.6 mm × 250 mm). Linear gradients using 10% A/ 90% B to 75% A/ 25% B over 20 min. d) Ms spectrum of peptide **1c** (Calculated Mass $[M+H]^+$: 615.2630; Observed Mass $[M+H]^+$: 615.2613). e) Ms spectrum of **3ca** (Calculated Mass $[M+H]^+$: 826.3400; $[M+2H]^{2+}$: 413.6737; Observed Mass $[M+H]^+$: 826.3381; $[M+2H]^{2+}$: 413.6729).

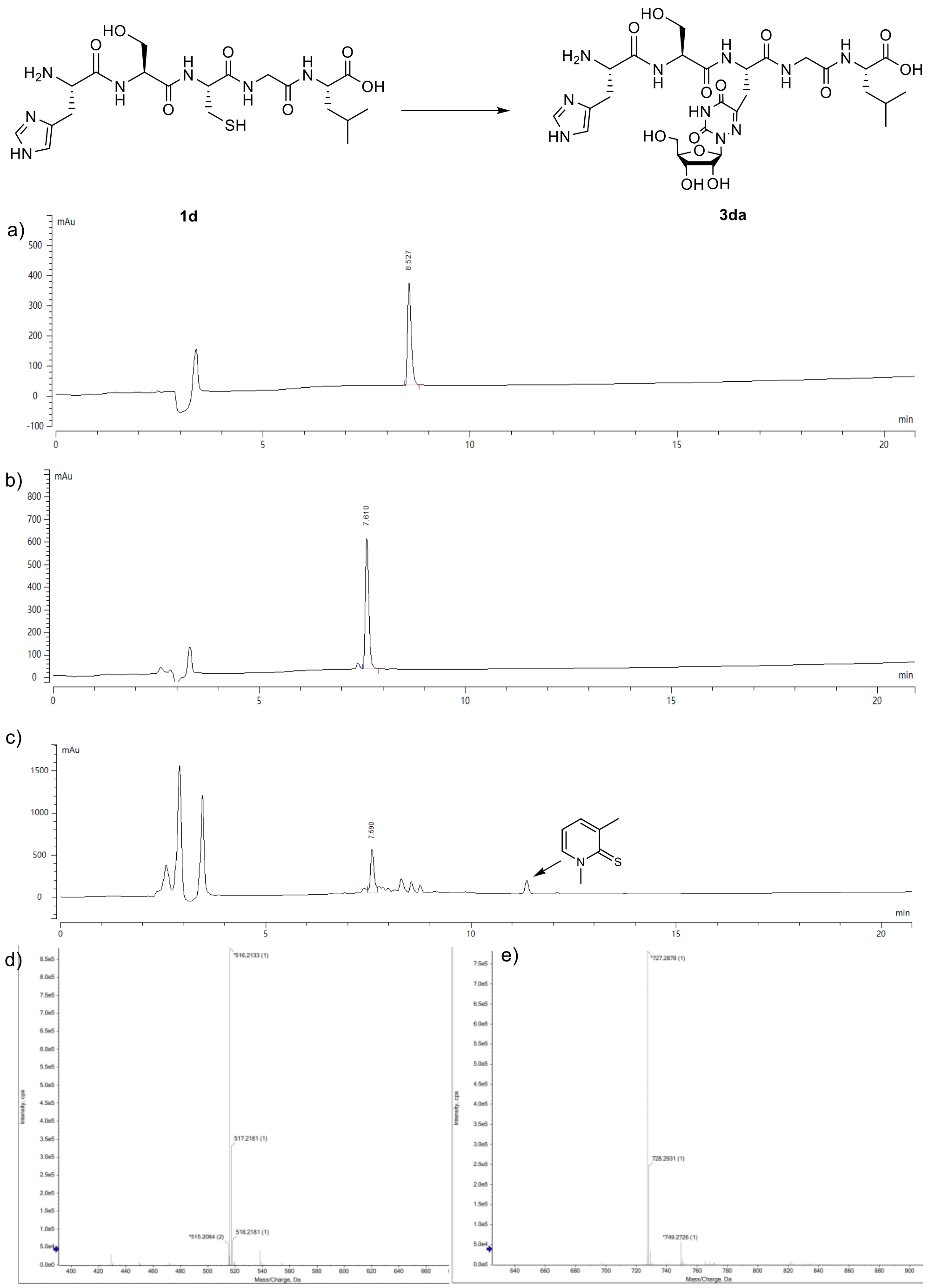

Figure S9. a) Analytical HPLC of peptide **1d**. b) Analytical HPLC of **3da**. c) Analytical HPLC trace of reaction system for 6-azauridine modification of peptide **1d.** Analyzing the purities was carried out on a UNIMICRO EasySep®-3030 using a Waters Symmetry® C18 Column (100 Å, 5 µm, 4.6 mm × 250 mm). Linear gradients using 10% A/ 90% B to 75% A/ 25% B over 20 min. d) Ms spectrum of peptide **1d** (Calculated Mass $[M+H]^+$: 516.2235; Observed Mass $[M+H]^+$: 516.2133). e) Ms spectrum of **3da** (Calculated Mass $[M+H]^+$: 727.3006; Observed Mass $[M+H]^+$: 727.2878).

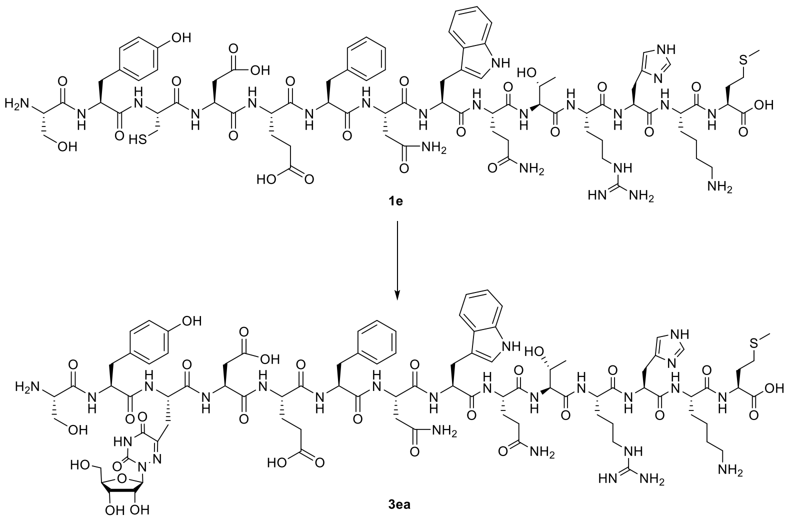

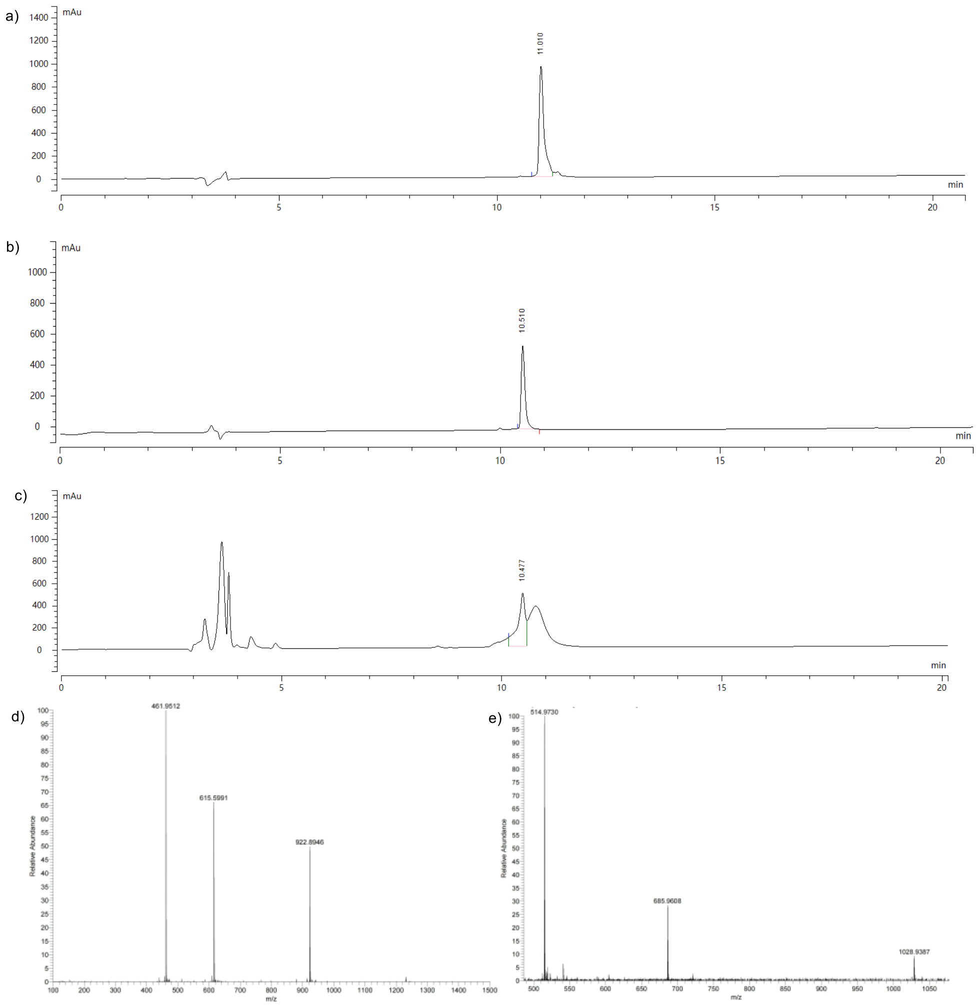

Figure S10. a) Analytical HPLC of peptide **1e**. b) Analytical HPLC of **3ea**. c) Analytical HPLC trace of reaction system for 6-azauridine modification of peptide **1e.** Analyzing the purities was carried out on a UNIMICRO EasySep®-3030 using a Globalsil® C18-AP column (120 Å, 5 μm, 4.6 mm × 250 mm). Linear gradients using 10% A/ 90% B to 75% A/ 25% B over 20 min. d) Ms spectrum of peptide **1e** (Calculated Mass $[M+2H]^{2+}$: 922.8958; $[M+3H]^{3+}$: 615.5996; $[M+4H]^{4+}$: 461.9515; Observed Mass $[M+2H]^{2+}$: 922.8946; $[M+3H]^{3+}$: 615.5991; $[M+4H]^{4+}$: 461.9512). e) Ms spectrum of **3ea** (Calculated Mass $[M+2H]^{2+}$: 1028.9360; $[M+3H]^{3+}$: 685.9587; $[M+4H]^{4+}$: 514.9716; Observed Mass $[M+2H]^{2+}$: 1028.9387; $[M+3H]^{3+}$: 685.9608; $[M+4H]^{4+}$: 514.9730).

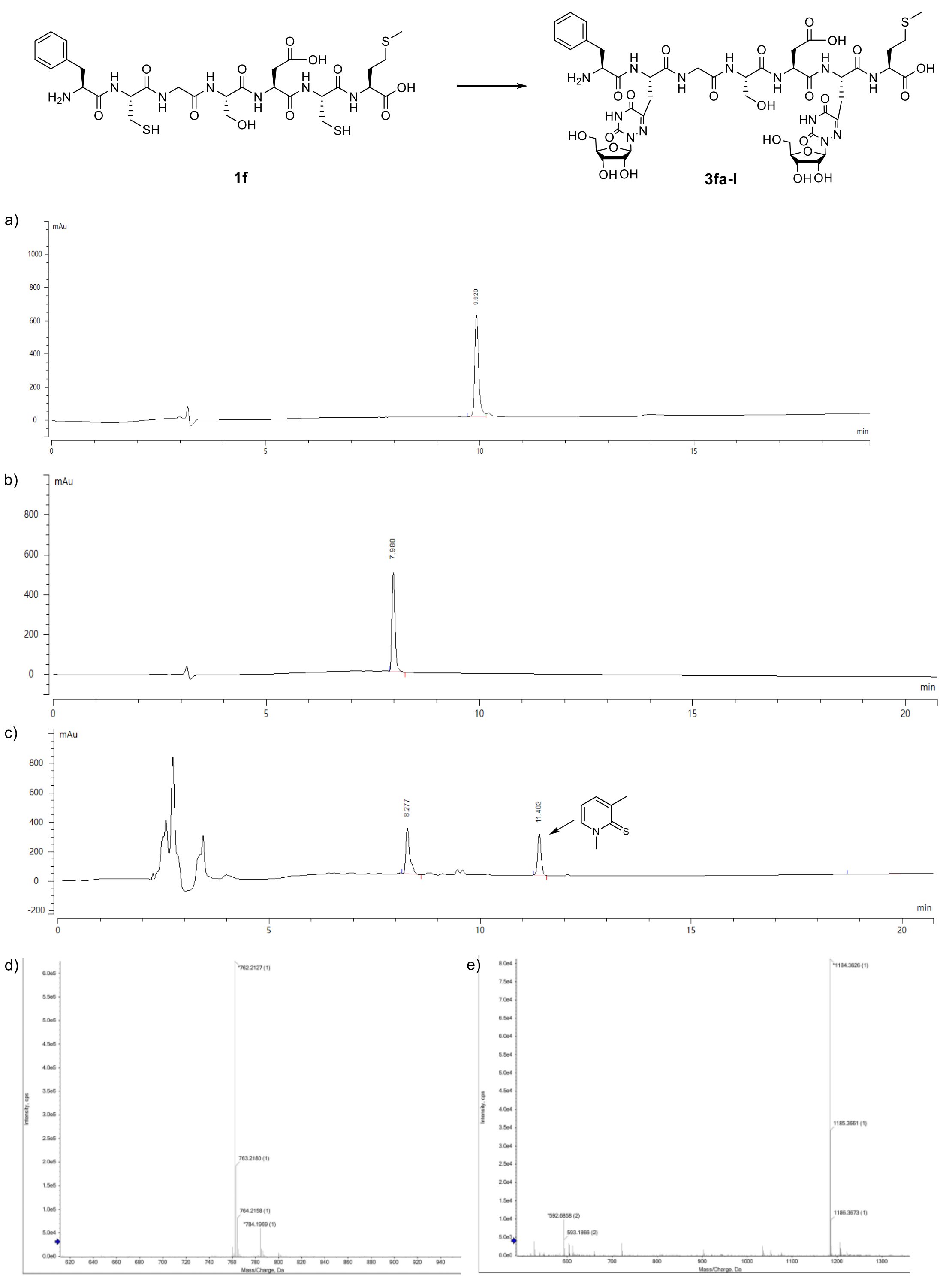

Figure S11a. a) Analytical HPLC of peptide **1f**. b) Analytical HPLC of **3fa-I**. c) Analytical HPLC trace of reaction system for 6-azauridine modification of peptide **1f**. Analyzing the purities was carried out on a UNIMICRO EasySep®-3030 using a Waters Symmetry® C18 Column (100 Å, 5 µm, 4.6 mm × 250 mm). Linear gradients using 10% A/ 90% B to 75% A/ 25% B over 20 min. d) Ms spectrum of peptide **1f** (Calculated Mass $[M+H]^+$: 762.2256; Observed Mass $[M+H]^+$: 762.2127). e) Ms spectrum of **3fa-I** (Calculated Mass $[M+H]^+$: 1184.3797; $[M+2H]^{2+}$: 592.6935; Observed Mass $[M+H]^+$: 1184.3626; $[M+2H]^{2+}$: 592.6858).

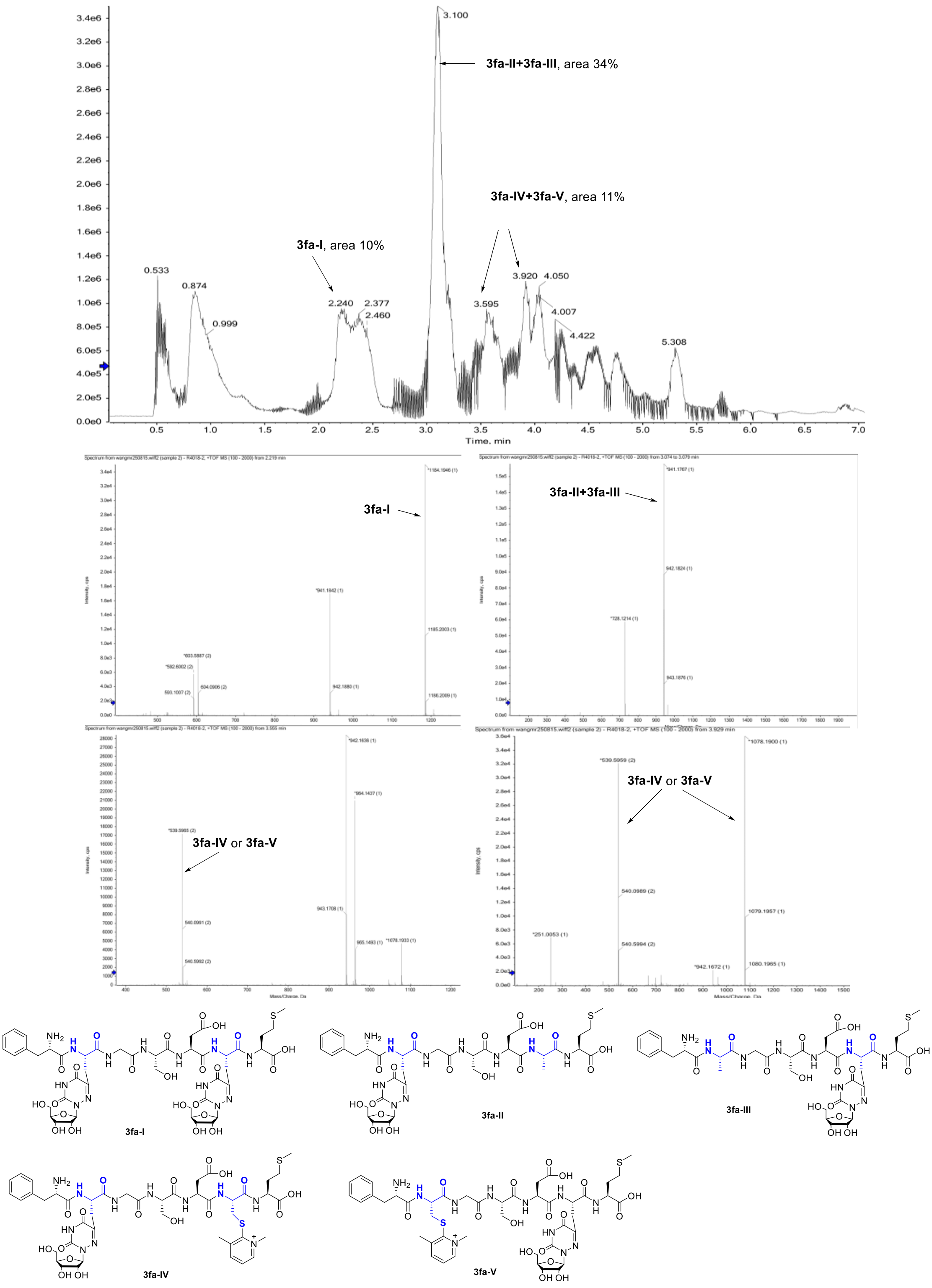

Figure S11b. When the reaction of peptide **1f** bearing two Cys was conducted under standard conditions [6-azauridine **2a** (2 equiv), pyridinium salt **VII** (1.1 equiv), 2,6-lutidine (3 equiv)], the reaction yielded both mono- and dimodified products: the dimodified product **3fa-I** was obtained in 10% yield, monomodified products included **3fa-II** and **3fa-III** gave 34% combined yield, and the S_NAr products (**3fa-IV** and **3fa-V**) formed in 11% combined yield. These results indicate that the desulfurative nucleotide modification itself is not chemoselective.

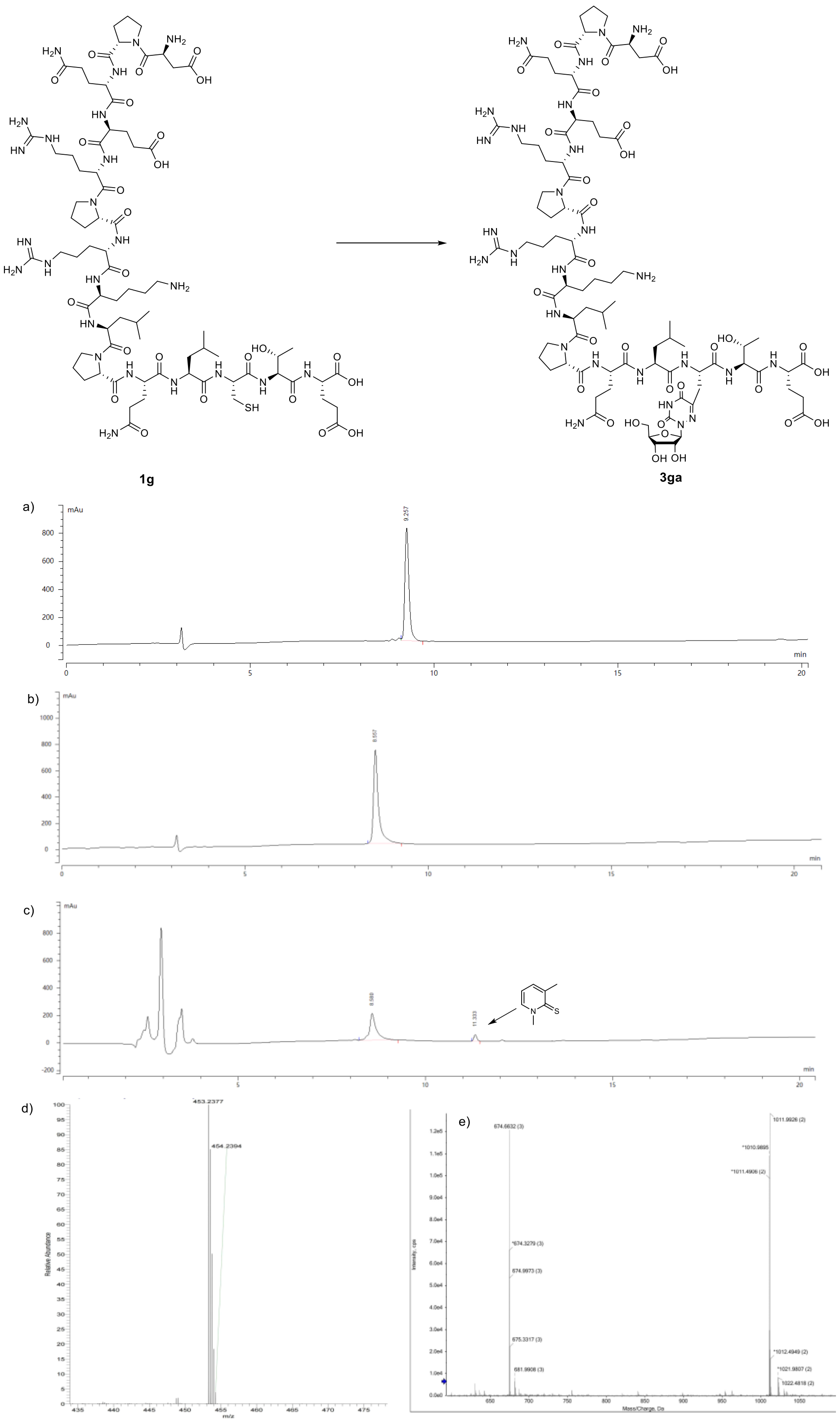

Figure S12. a) Analytical HPLC of peptide **1g**. b) Analytical HPLC of **3ga**. c) Analytical HPLC trace of reaction system for 6-azauridine modification of peptide **1g**. Analyzing the purities was carried out on a UNIMICRO EasySep®-3030 using a Waters Symmetry® C18 Column (100 Å, 5 µm, 4.6 mm × 250 mm). Linear gradients using 10% A/ 90% B to 75% A/ 25% B over 20 min. d) Ms spectrum of peptide **1g** (Calculated Mass $[M+4H]^{4+}$: 453.2374; Observed Mass $[M+4H]^{4+}$: 453.2377). e) Ms spectrum of **3ga** (Calculated Mass $[M+2H]^{2+}$: 1011.0060; $[M+3H]^{3+}$: 674.6742; Observed Mass $[M+2H]^{2+}$: 1010.9895; $[M+3H]^{3+}$: 674.6632).

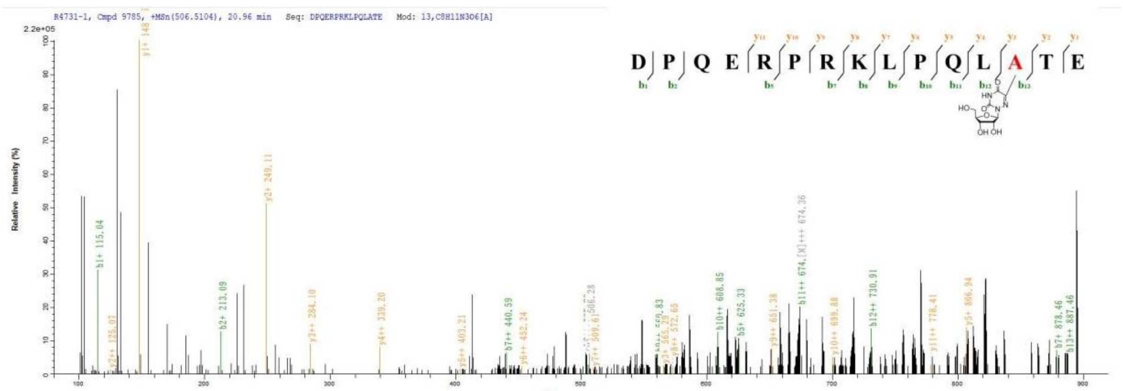

Figure S13. MS-MS of **3ga (**Thermo Fisher, Q Exactive HF-X)**.**

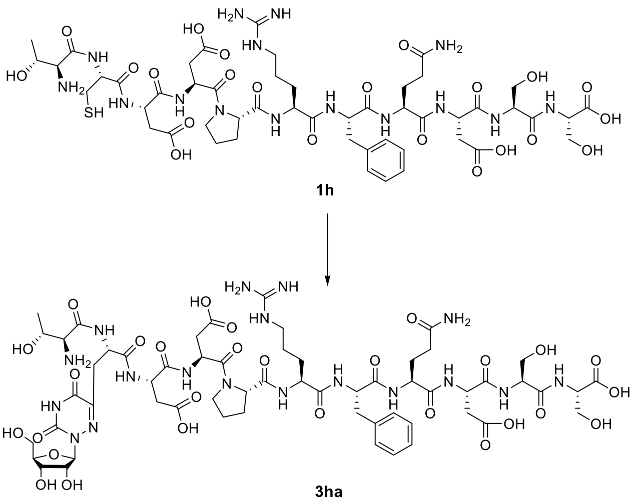

3ha

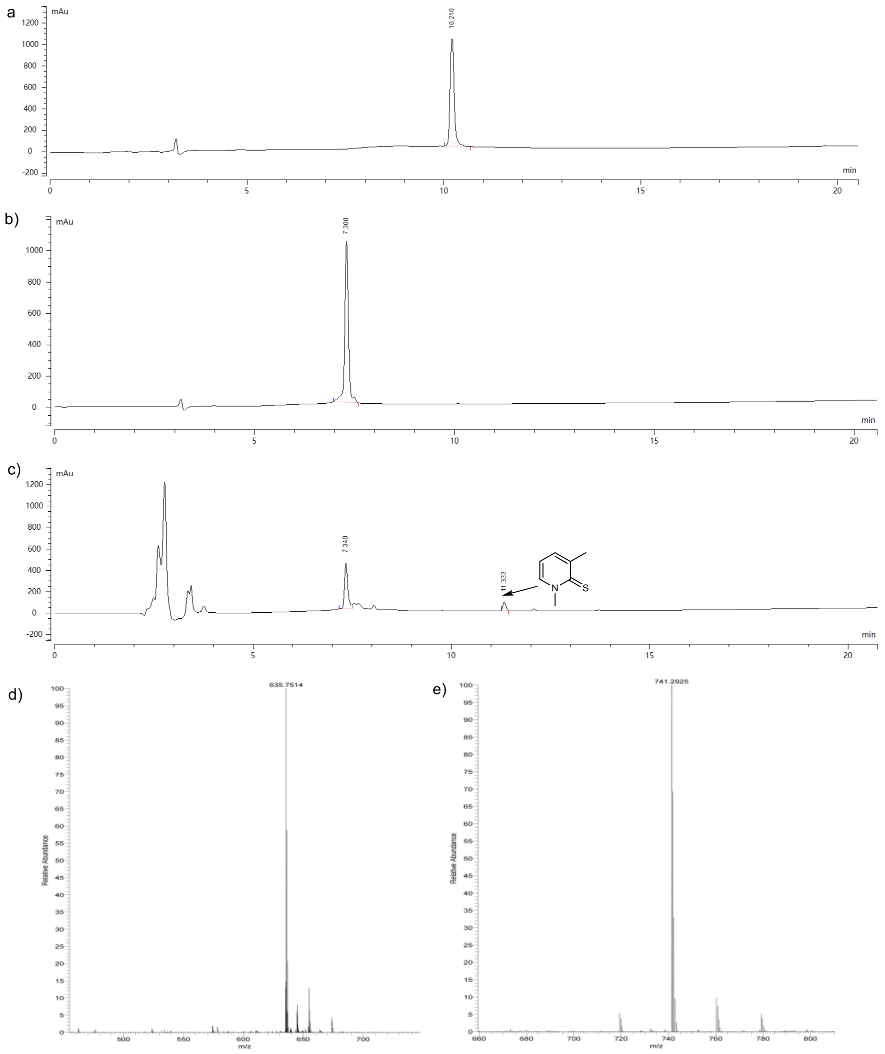

Figure S14. a) Analytical HPLC of peptide **1h**. b) Analytical HPLC of **3ha**. c) Analytical HPLC trace of reaction system for 6-azauridine modification of peptide **1h.** Analyzing the purities was carried out on a UNIMICRO EasySep®-3030 using a Waters Symmetry® C18 Column (100 Å, 5 µm, 4.6 mm × 250 mm). Linear gradients using 10% A/ 90% B to 75% A/ 25% B over 20 min. d) Ms spectrum of peptide **1h** (Calculated Mass $[M+2H]^{2+}$: 635.7539; Observed Mass $[M+2H]^{2+}$: 635.7514). e) Ms spectrum of **3ha** (Calculated Mass $[M+2H]^{2+}$: 741.2924; Observed Mass $[M+2H]^{2+}$: 741.2925).

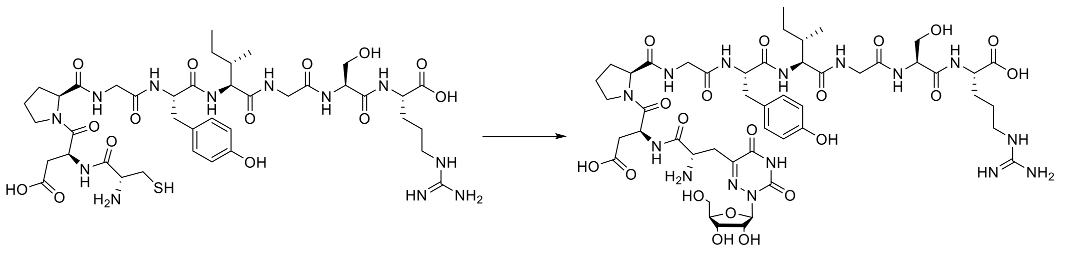

1i **3ia**

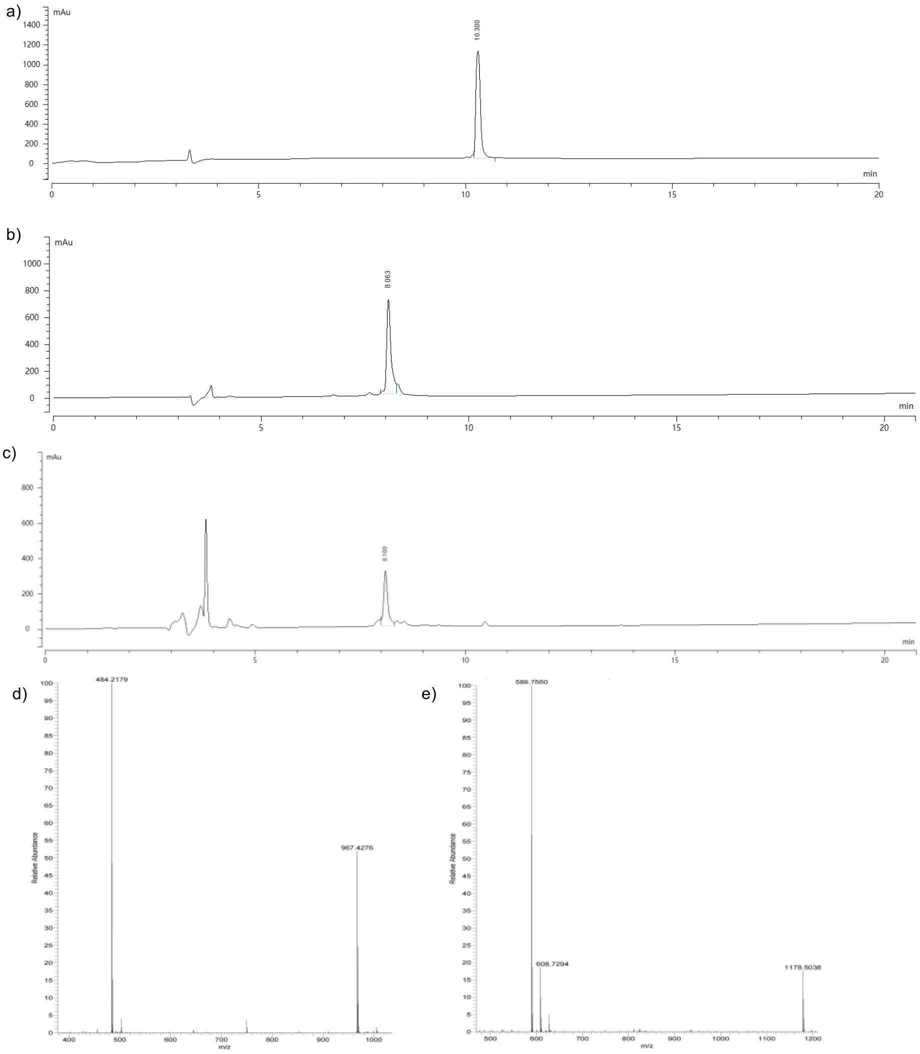

Figure S15. a) Analytical HPLC of peptide **1i**. b) Analytical HPLC of **3ia**. c) Analytical HPLC trace of reaction system for 6-azauridine modification of peptide **1i.** Analyzing the purities was carried out on a UNIMICRO EasySep®-3030 using a Globalsil® C18-AP column (120 Å, 5 µm, 4.6 mm × 250 mm). Linear gradients using 10% A/ 90% B to 75% A/ 25% B over 20 min. d) Ms spectrum of peptide **1i** (Calculated Mass $[M+H]^+$: 967.4302; $[M+2H]^{2+}$: 484.2188; Observed Mass $[M+H]^+$: 967.4276; $[M+2H]^{2+}$: 484.2179). e) Ms spectrum of **3ia** (Calculated Mass $[M+H]^+$: 1078.5073; $[M+2H]^{2+}$: 589.7573; Observed Mass $[M+H]^+$: 1078.5038; $[M+2H]^{2+}$: 589.7560).

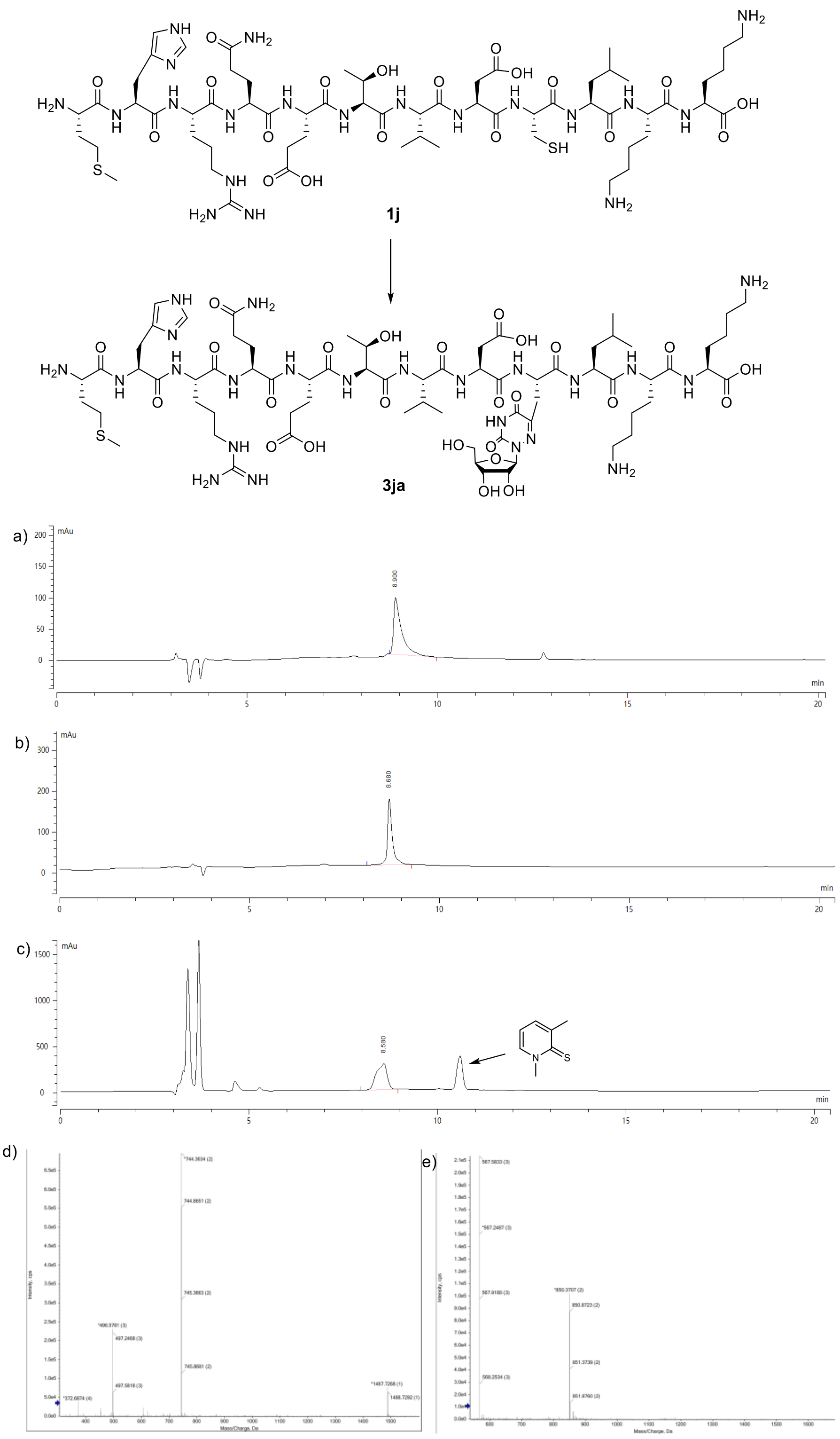

Figure S16. a) Analytical HPLC of peptide **1j**. b) Analytical HPLC of **3ja**. c) Analytical HPLC trace of reaction system for 6-azauridine modification of peptide **1j.** Analyzing the purities was carried out on a UNIMICRO EasySep®-3030 using a Globalsil® C18-AP column (120 Å, 5 µm, 4.6 mm × 250 mm). Linear gradients using 10% A/ 90% B to 75% A/ 25% B over 20 min. d) Ms spectrum of peptide **1j** (Calculated Mass $[M+H]^{+}$: 1487.7458; $[M+2H]^{2+}$: 744.3765; $[M+3H]^{3+}$: 496.5868; $[M+4H]^{4+}$: 372.6919; Observed Mass $[M+H]^{+}$: 1487.7268; $[M+2H]^{2+}$: 744.3634; $[M+3H]^{3+}$: 496.5781; $[M+4H]^{4+}$: 372.6974). e) Ms spectrum of **3ja** (Calculated Mass $[M+2H]^{2+}$: 849.9151; $[M+3H]^{3+}$: 567.2802; Observed Mass $[M+2H]^{2+}$: 850.3707; $[M+3H]^{3+}$: 567.5833).

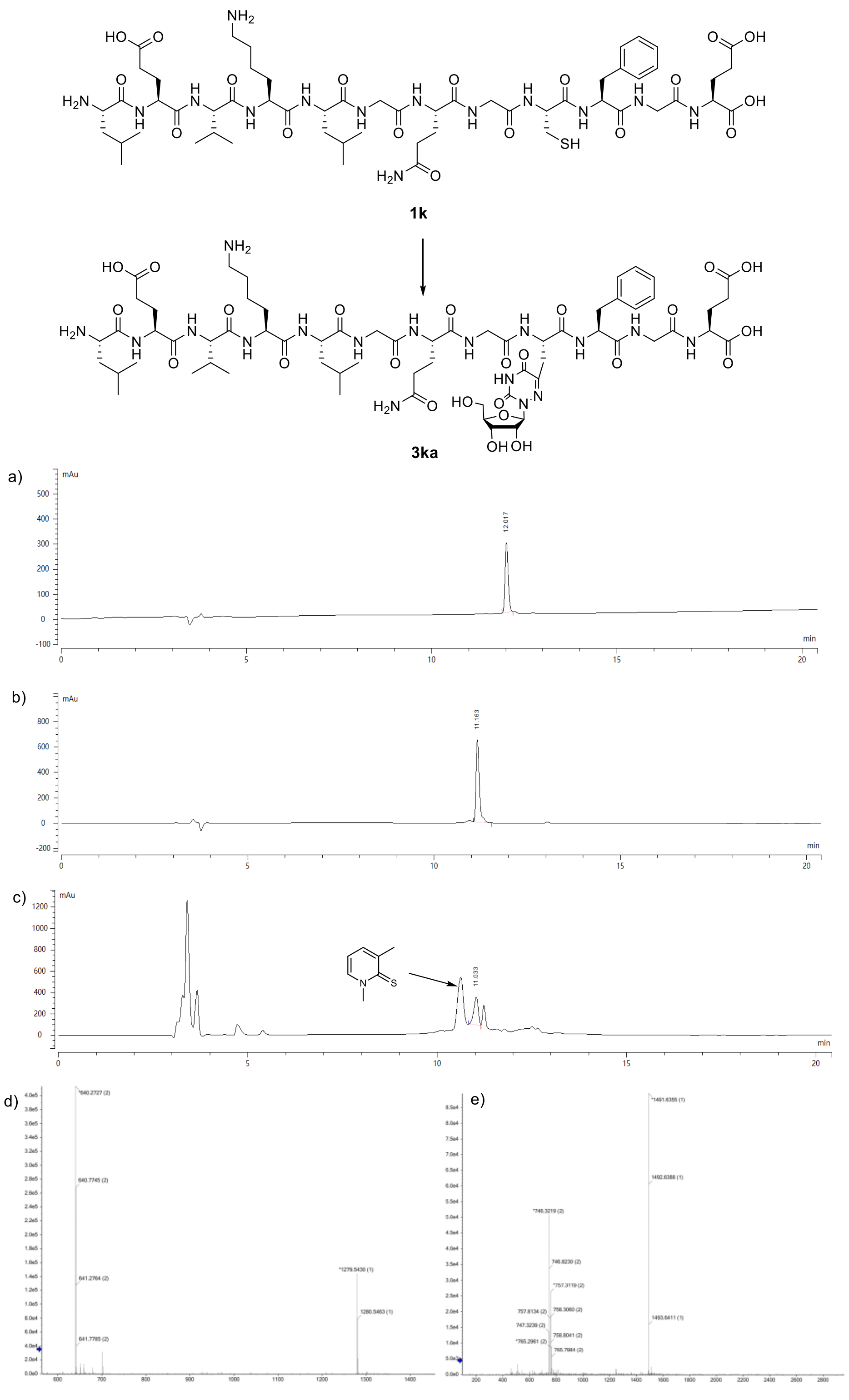

Figure S17. a) Analytical HPLC of peptide **1k**. b) Analytical HPLC of **3ka**. c) Analytical HPLC trace of reaction system for 6-azauridine modification of peptide **1k.** Analyzing the purities was carried out on a UNIMICRO EasySep®-3030 using a Globalsil® C18-AP column (120 Å, 5 µm, 4.6 mm × 250 mm). Linear gradients using 10% A/ 90% B to 75% A/ 25% B over 20 min. d) Ms spectrum of peptide **1k** (Calculated Mass $[M+H]^+$: 1279.6351; $[M+2H]^{2+}$: 640.3212; Observed Mass $[M+H]^+$: 1279.5430; $[M+2H]^{2+}$: 640.2727). e) Ms spectrum of **3ka** (Calculated Mass $[M+H]^+$: 1490.7122; $[M+2H]^{2+}$: 745.8597; Observed Mass $[M+H]^+$: 1491.6355; $[M+2H]^{2+}$: 746.3219).

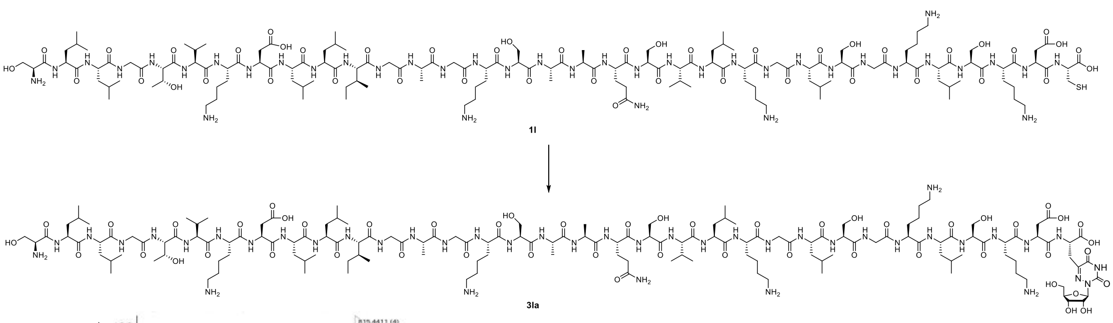

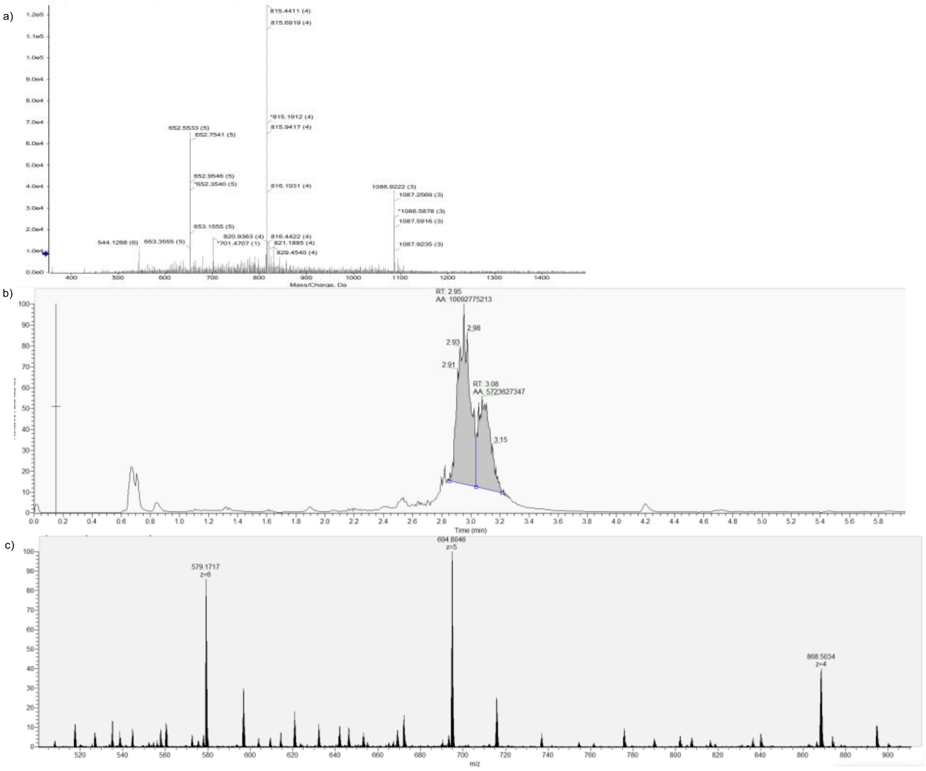

Figure S18. a) Ms spectrum of peptide **1l** (Calculated Mass $[M+3H]^{3+}$: 1086.9560; $[M+4H]^{4+}$: 815.4688; $[M+5H]^{5+}$: 652.5765; $[M+6H]^{6+}$: 544.1489; Observed Mass $[M+3H]^{3+}$: 1086.9222; $[M+4H]^{4+}$: 815.4411; $[M+5H]^{5+}$: 652.5533; $[M+6H]^{6+}$: 544.1288). b) TIC spectrum of the reaction system for 6-azauridine modification of peptide **1l**. c) Ms spectrum of retain time in 2.95 min (Calculated $[M+4H]^{4+}$:868.2381; $[M+5H]^{5+}$:694.7919; $[M+6H]^{6+}$:579.1612; Observed $[M+4H]^{4+}$:868.5034; $[M+5H]^{5+}$:694.8046; $[M+6H]^{6+}$:579.1717).

Peptide modification reaction was monitored and evaluated using a Thermo Scientific Orbitrap IQ-X Tribrid coupled to an Thermo Scientific™ Accucore™ RP-MS column (2.6 µm, 2.1 mm × 100mm). Solvent A (water with 0.1% formic acid) and solvent B (acetonitrile) were used as the mobile phase at a flow rate of 0.3 mL/min. The gradient was programmed as follows: 20% B in 2 min, then 20% B to 70% B from 2 min to 5 min, 70% B to 95% B from 5.1 min to 8 min. The target product **3la** was detected in 2.91-2.98 min.

The conversion rate of **1l** can be calculated using the following formula:

$$\text{Conversion rate }(\%) = \frac{B1}{B1 + B2} \times 100$$

B1: The mass intensity of 2.95 min in the reaction mixture.

B2: The mass intensity of 3.08 min in the reaction mixture.

$$\text{Conversion rate}(\%) = \frac{10092775213}{10092775213 + 5723627347} \times 100 = 63.8\%$$

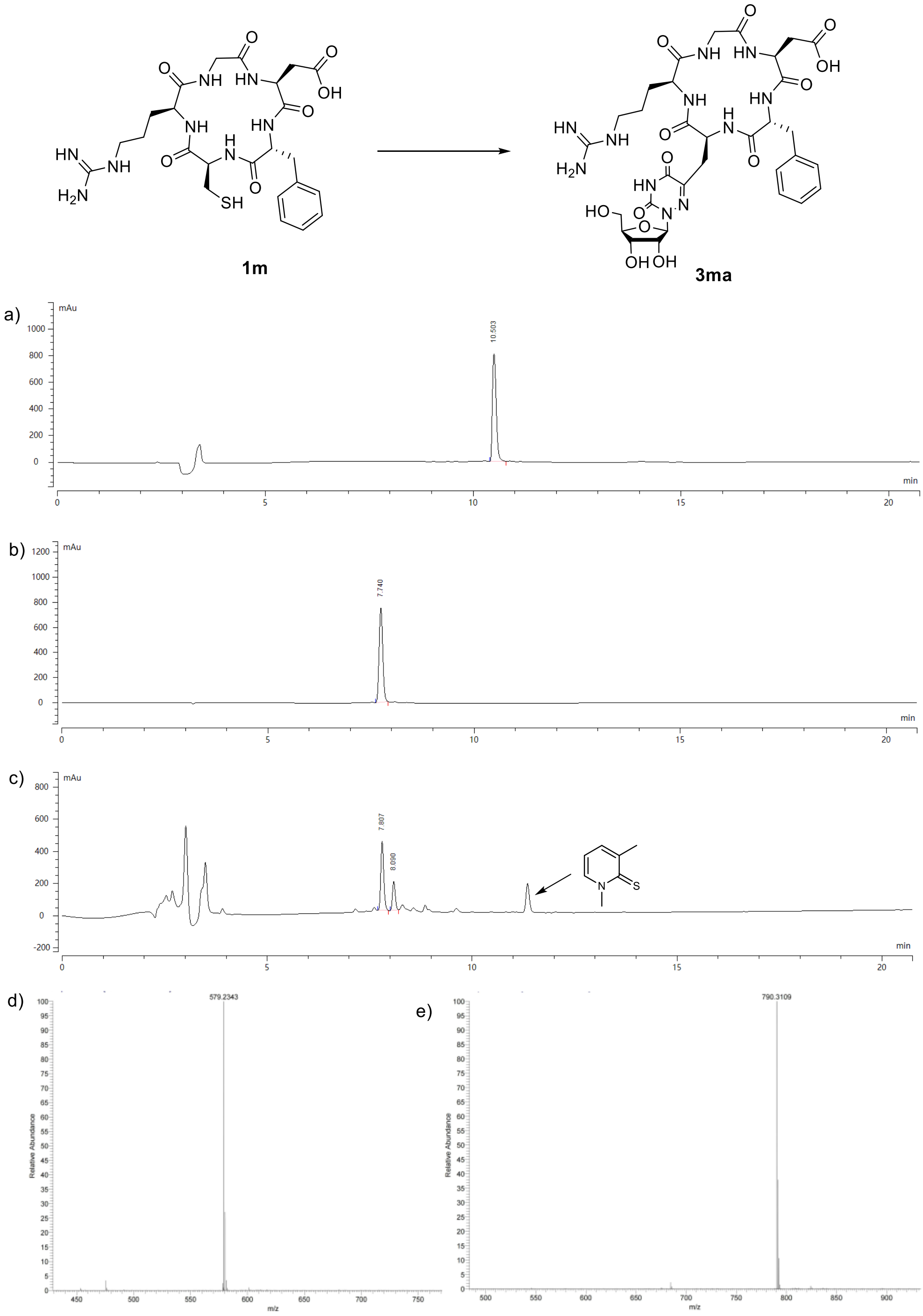

Figure S19. a) Analytical HPLC of peptide **1m**. b) Analytical HPLC of **3ma**. c) Analytical HPLC trace of reaction system for 6-azauridine modification of peptide **1m.** Analyzing the purities was carried out on a UNIMICRO EasySep®-3030 using a Waters Symmetry® C18 Column (100 Å, 5 µm, 4.6 mm × 250 mm). Linear gradients using 10% A/ 90% B to 75% A/ 25% B over 20 min. d) Ms spectrum of peptide **1m** (Calculated Mass $[M+H]^+$: 579.2344; Observed Mass $[M+H]^+$: 579.2343). e) Ms spectrum of **3ma** (Calculated Mass $[M+H]^+$: 790.3115; Observed Mass $[M+H]^+$: 790.3109).

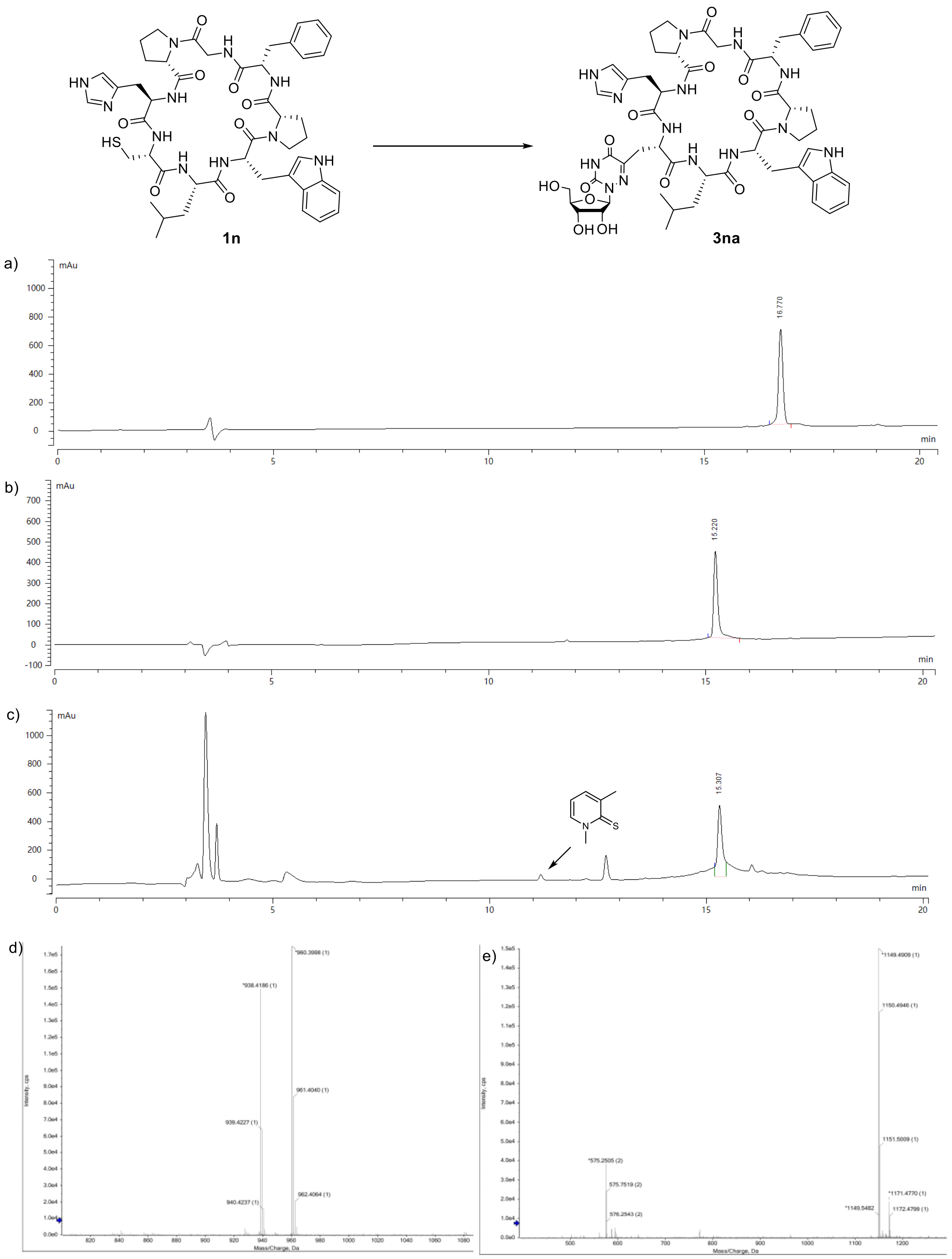

Figure S20. a) Analytical HPLC of peptide **1n**. b) Analytical HPLC of **3na**. c) Analytical HPLC trace of reaction system for 6-azauridine modification of peptide **1n.** Analyzing the purities was carried out on a UNIMICRO EasySep®-3030 using a Waters Symmetry® C18 Column (100 Å, 5 μm, 4.6 mm × 250 mm). Linear gradients using 10% A/ 90% B to 75% A/ 25% B over 20 min. d) Ms spectrum of peptide **1n** (Calculated Mass $[M+H]^+$: 938.4342; $[M+Na]^+$: 960.4161; Observed Mass $[M+H]^+$: 938.4186; $[M+Na]^+$: 960.3998). e) Ms spectrum of **3na** (Calculated Mass $[M+H]^+$: 1149.5113; $[M+2H]^{2+}$: 575.2593; Observed Mass $[M+H]^+$: 1149.4909; $[M+2H]^{2+}$: 575.2505).

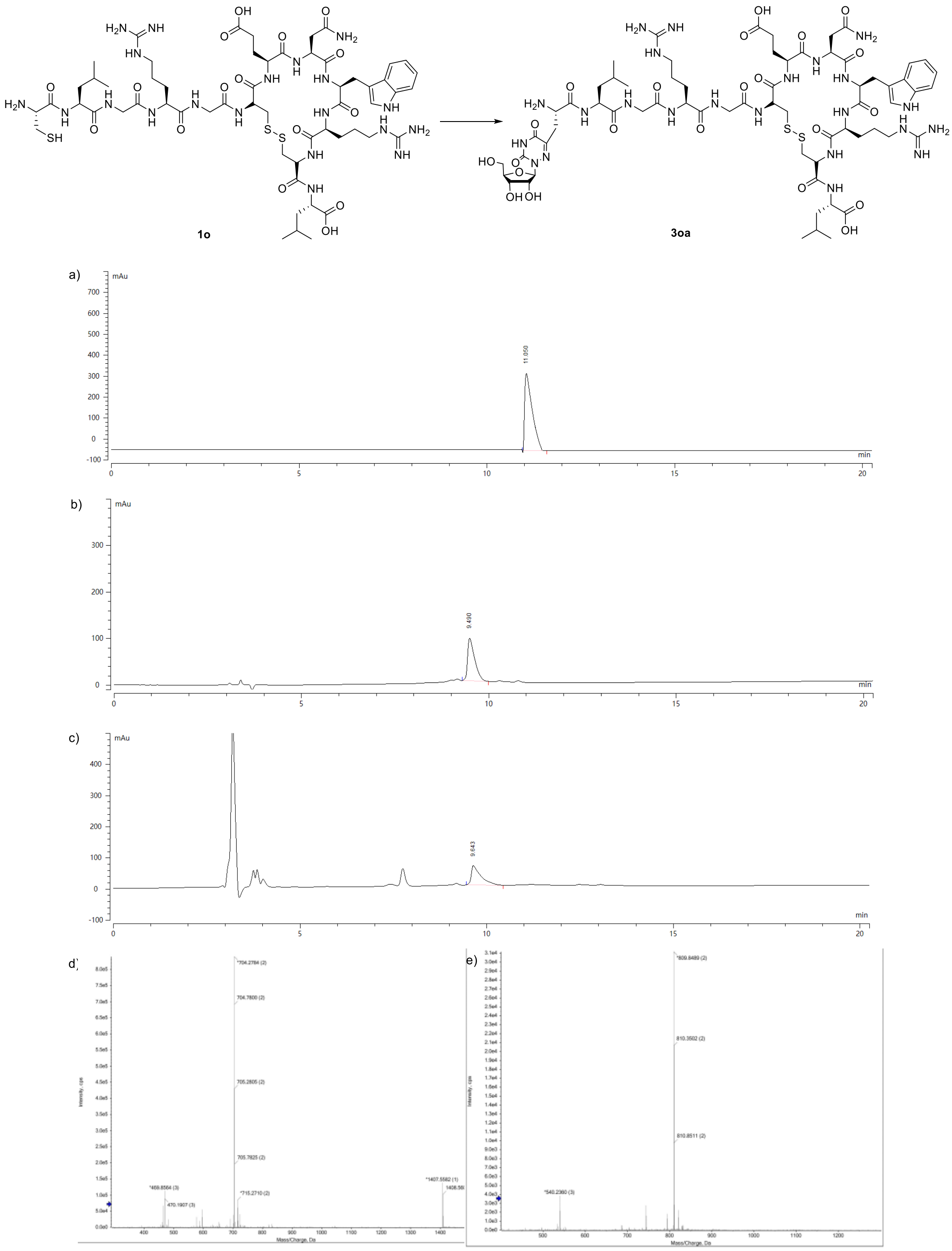

Figure S21. a) Analytical HPLC of peptide **1o**. b) Analytical HPLC of **3oa**. c) Analytical HPLC trace of reaction system for 6-azauridine modification of peptide **1o.** Analyzing the purities was carried out on a UNIMICRO EasySep®-3030 using a Globalsil® C18-AP column (120 Å, 5 μm, 4.6 mm × 250 mm). Linear gradients using 20% A/ 80% B to 50% A/ 50% B over 25 min. d) Ms spectrum of peptide **1o** (Calculated Mass $[M+H]^+$:1407.6079; $[M+2H]^{2+}$:704.3076; $[M+3H]^{3+}$:469.8742; Observed Mass $[M+H]^+$: 1407.5582; $[M+2H]^{2+}$: 704.2784; $[M+3H]^{3+}$: 469.8564). e) Ms spectrum of **3oa** (Calculated Mass $[M+2H]^{2+}$: 809.8461; $[M+3H]^{3+}$: 540.2332; Observed Mass $[M+2H]^{2+}$: 809.8489; $[M+3H]^{3+}$: 540.2360).

Analytical data for **3mb-3mp**

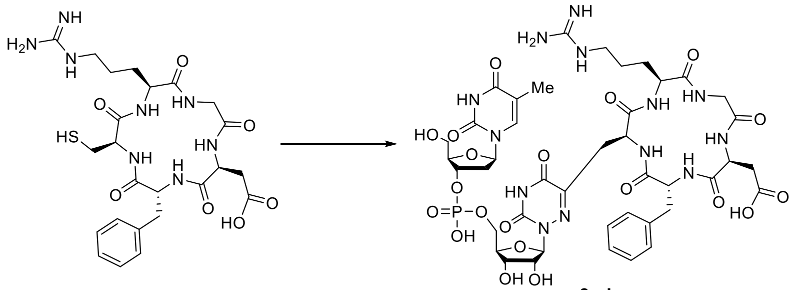

3mb

a)

b)

c)

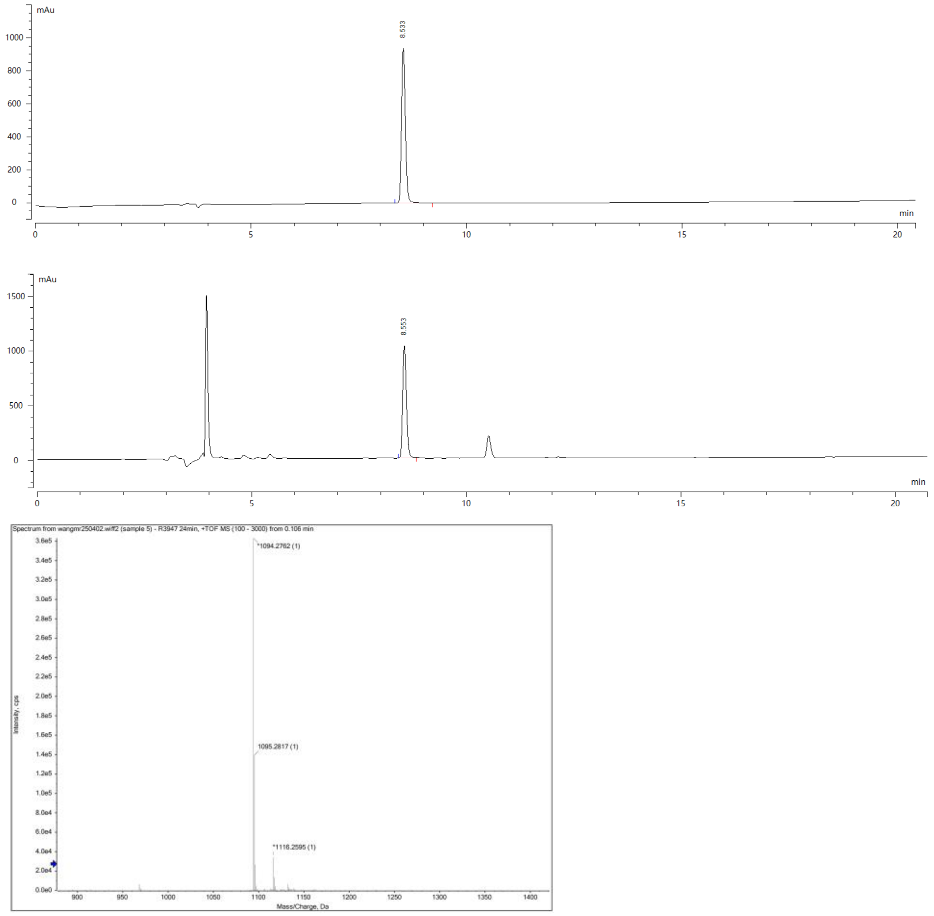

Figure S22. a) Analytical HPLC of **3mb.** b) Analytical HPLC trace of reaction system for synthesizing **3mb.** c) Ms spectrum of **3mb** (Calculated Mass $[M+H]^+$: 1094.3575; Observed Mass $[M+H]^+$: 1094.2764). Analyzing the purities was carried out on a UNIMICRO EasySep®-3030 using a Globalsil® C18 Column (100 Å, 5 μm, 4.6 mm × 250 mm). Linear gradients using 10% A/ 90% B to 75% A/ 25% B over 20 min.

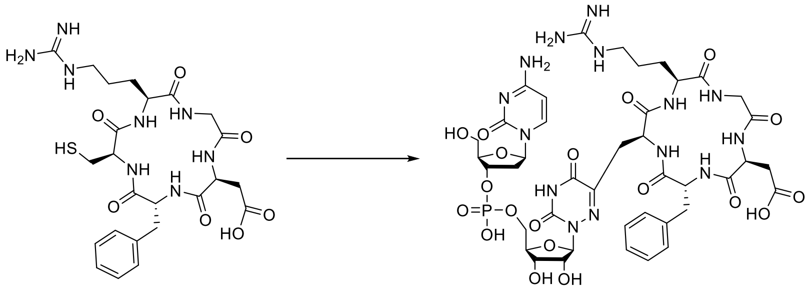

3mc

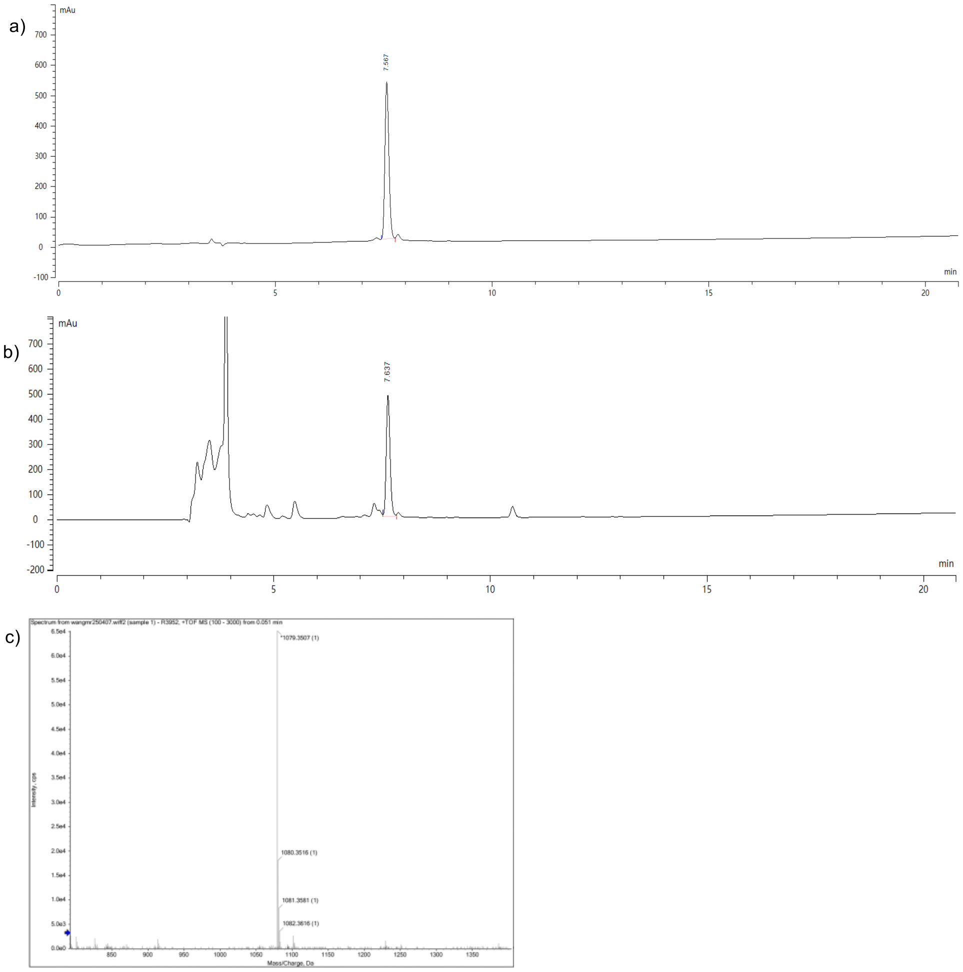

Figure S23. a) Analytical HPLC of **3mc.** b) Analytical HPLC trace of reaction system for synthesizing **3mc.** c) Ms spectrum of **3mc** (Calculated Mass $[M+H]^+$: 1079.3579; Observed Mass $[M+H]^+$: 1079.3507). Analyzing the purities was carried out on a UNIMICRO EasySep®-3030 using a Globalsil® C18 Column (100 Å, 5 µm, 4.6 mm × 250 mm). Linear gradients using 10% A/ 90% B to 75% A/ 25% B over 20 min.

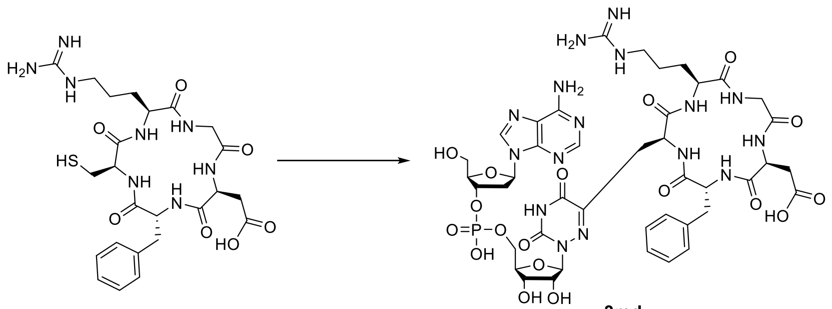

3md

a)

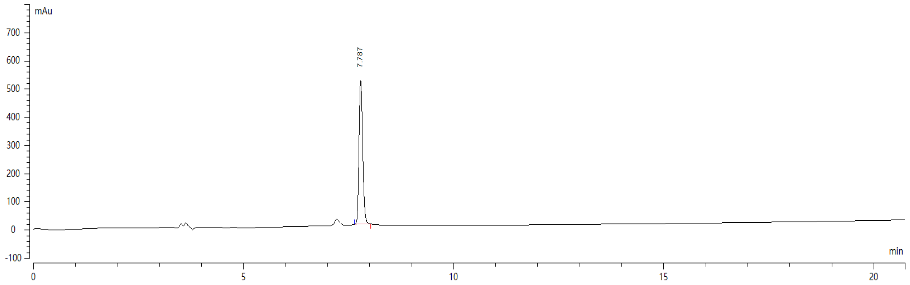

b)

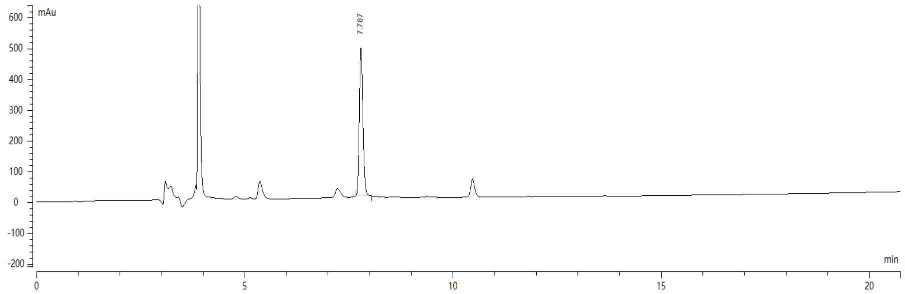

c)

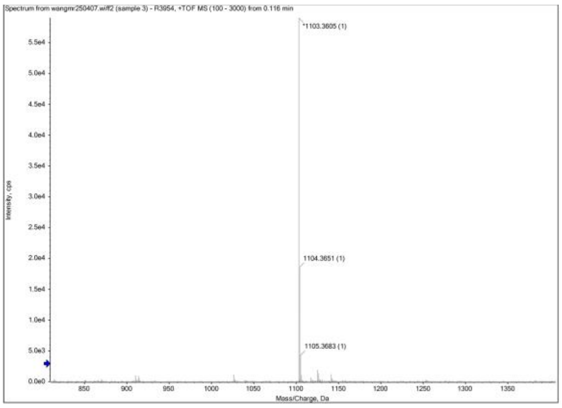

Figure S24. a) Analytical HPLC of **3md.** b) Analytical HPLC trace of reaction system for synthesizing **3md.** c) Ms spectrum of **3md** (Calculated Mass $[M+H]^+$: 1103.3691; Observed Mass $[M+H]^+$: 1103.3605). Analyzing the purities was carried out on a UNIMICRO EasySep®-3030 using a Globalsil® C18 Column (100 Å, 5 μm, 4.6 mm × 250 mm). Linear gradients using 10% A/ 90% B to 75% A/ 25% B over 20 min.

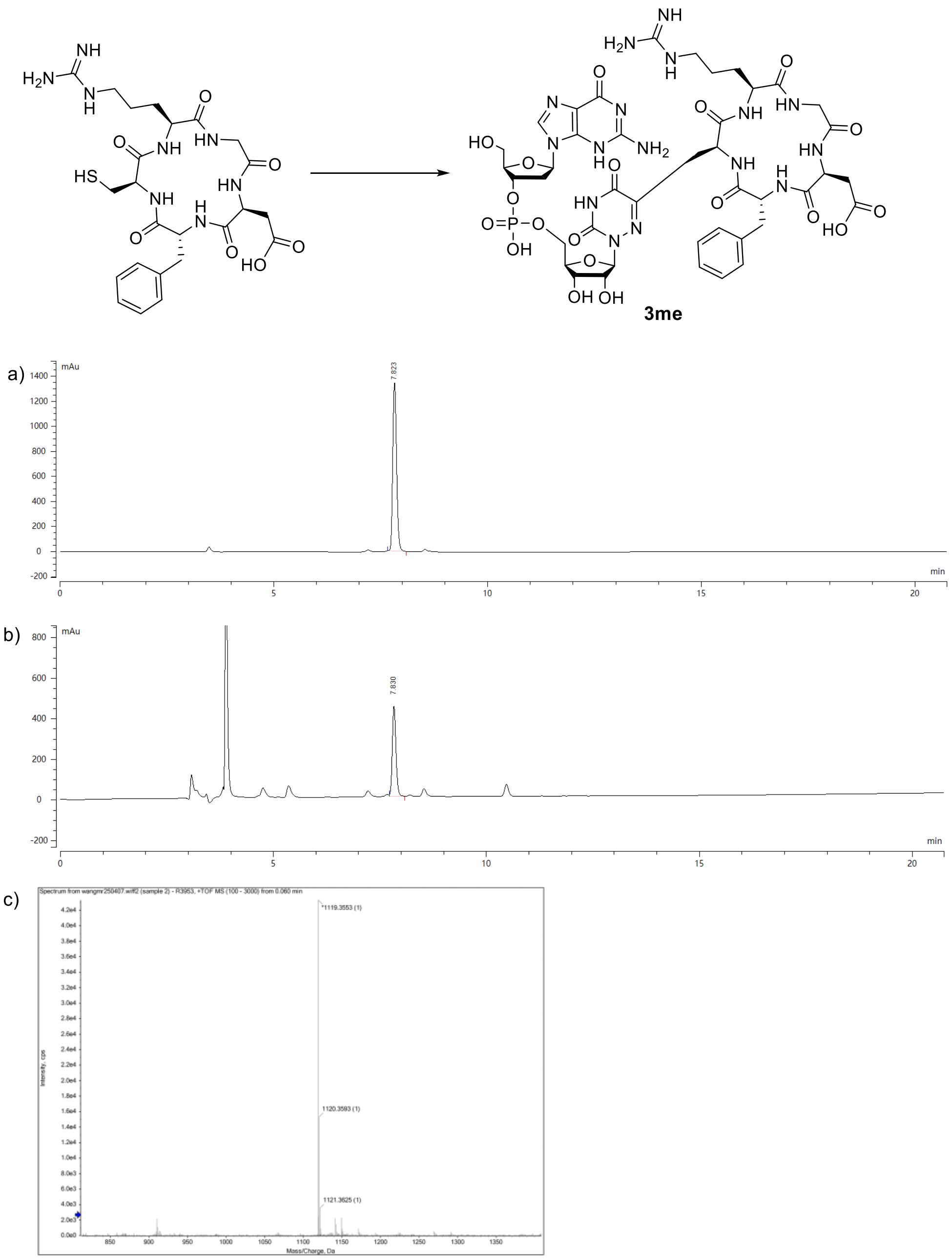

Figure S25. a) Analytical HPLC of **3me.** b) Analytical HPLC trace of reaction system for synthesizing **3me.** c) Ms spectrum of **3me** (Calculated Mass $[M+H]^+$: 1119.3640; Observed Mass $[M+H]^+$: 1119.3553). Analyzing the purities was carried out on a UNIMICRO EasySep®-3030 using a Globalsil® C18 Column (100 Å, 5 µm, 4.6 mm × 250 mm). Linear gradients using 10% A/ 90% B to 75% A/ 25% B over 20 min.

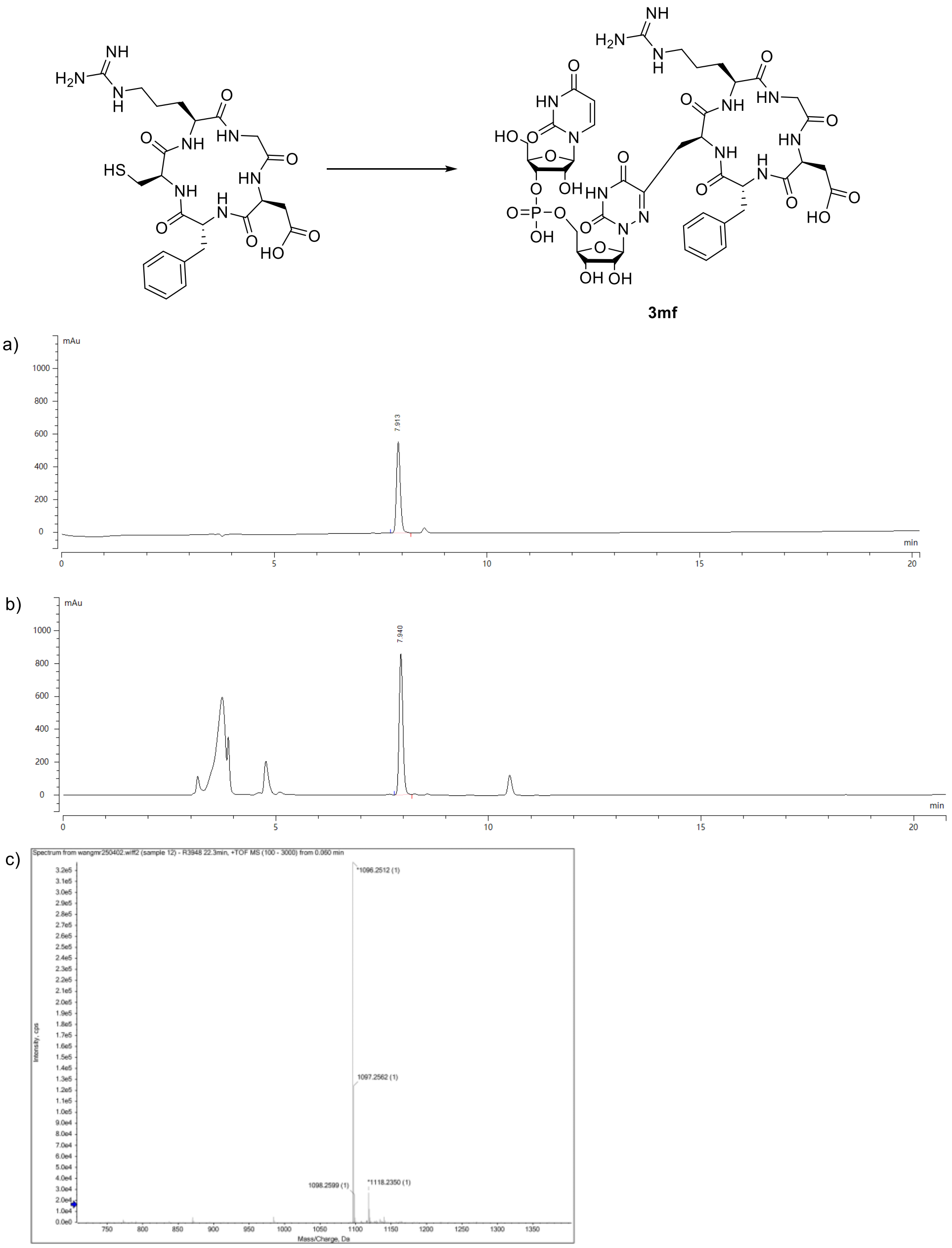

Figure S26. a) Analytical HPLC of **3mf.** b) Analytical HPLC trace of reaction system for synthesizing **3mf.** c) Ms spectrum of **3mf** (Calculated Mass $[M+H]^+$: 1096.3368; Observed Mass $[M+H]^+$: 1096.2512). Analyzing the purities was carried out on a UNIMICRO EasySep®-3030 using a Globalsil® C18 Column (100 Å, 5 μm, 4.6 mm × 250 mm). Linear gradients using 10% A/ 90% B to 75% A/ 25% B over 20 min.

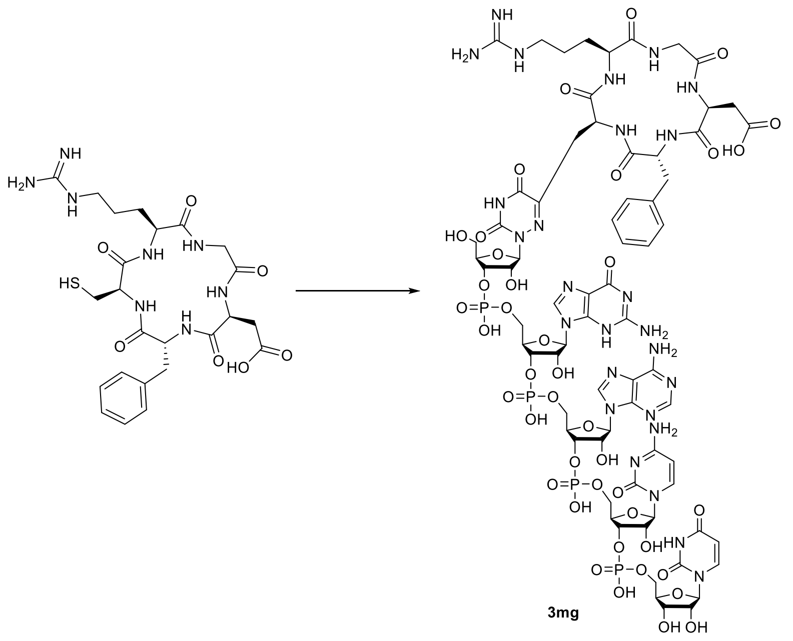

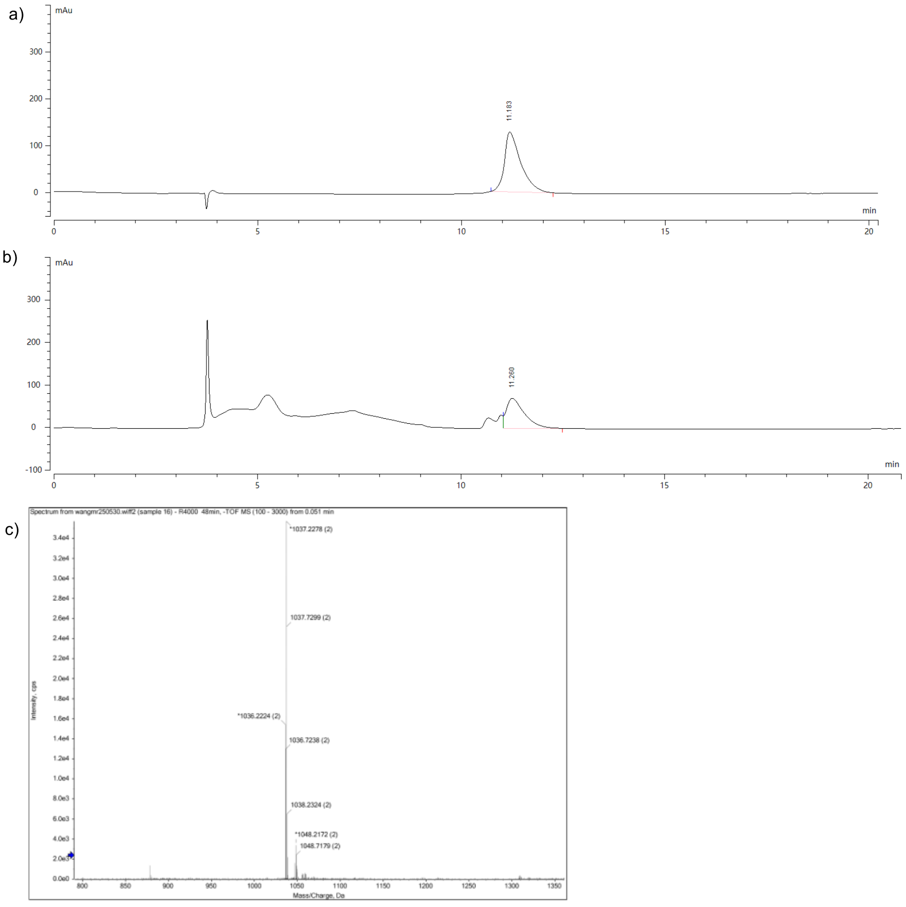

Figure S27. a) Analytical HPLC of **3mg.** b) Analytical HPLC trace of reaction system for synthesizing **3mg.** c) Ms spectrum of **3mg** (Calculated Mass $[M\text{-}2H]^{2-}$: 1036.7297; Observed Mass $[M\text{-}2H]^{2-}$: 1037.2278). Analyzing the purities was carried out on a UNIMICRO EasySep®-3030 using a Globalsil® C18 Column (100 Å, 5 µm, 4.6 mm × 250 mm). Linear gradients using 5% A/ 95% B to 30% A/ 70% B over 20 min.

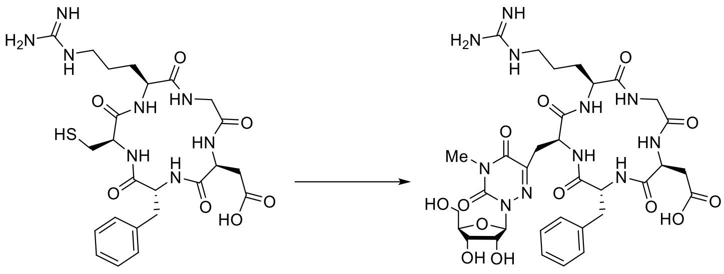

3mh

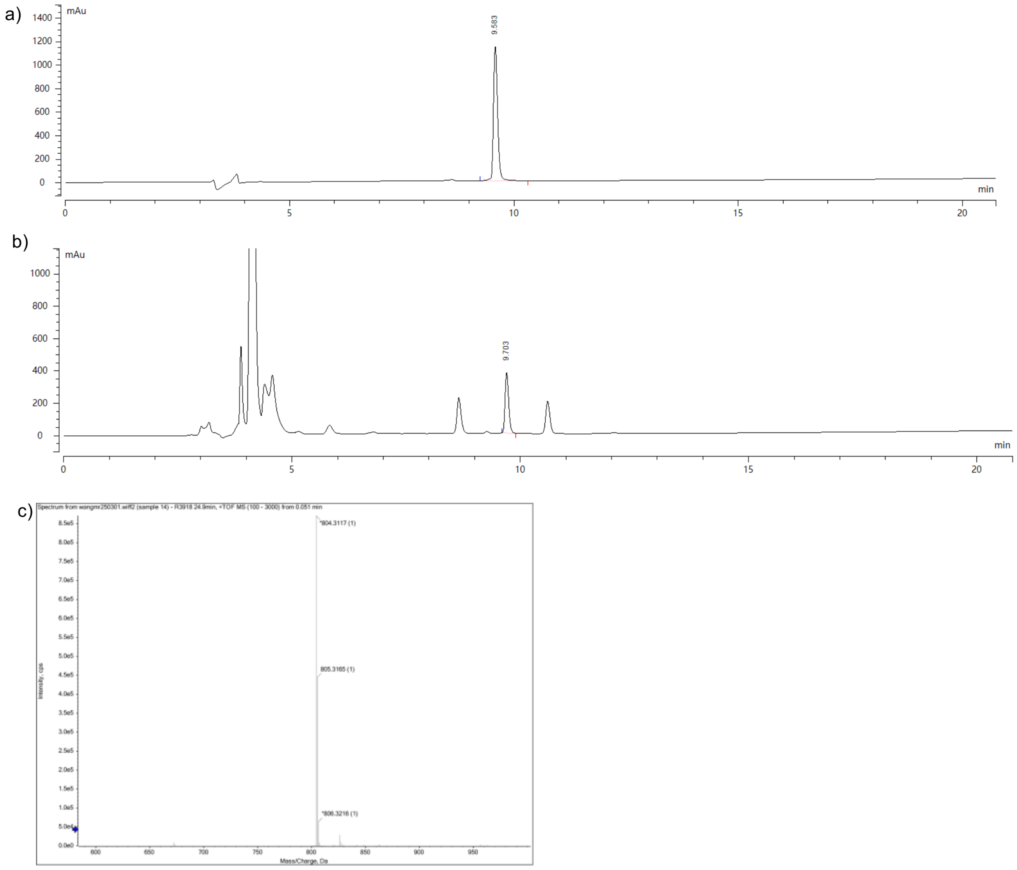

Figure S28. a) Analytical HPLC of **3mh.** b) Analytical HPLC trace of reaction system for synthesizing **3mh.** c) Ms spectrum of **3mh** (Calculated Mass $[M+H]^+$: 804.3272; Observed Mass $[M+H]^+$: 804.3117). Analyzing the purities was carried out on a UNIMICRO EasySep®-3030 using a Globalsil® C18 Column (100 Å, 5 µm, 4.6 mm × 250 mm). Linear gradients using 10% A/ 90% B to 75% A/ 25% B over 20 min.

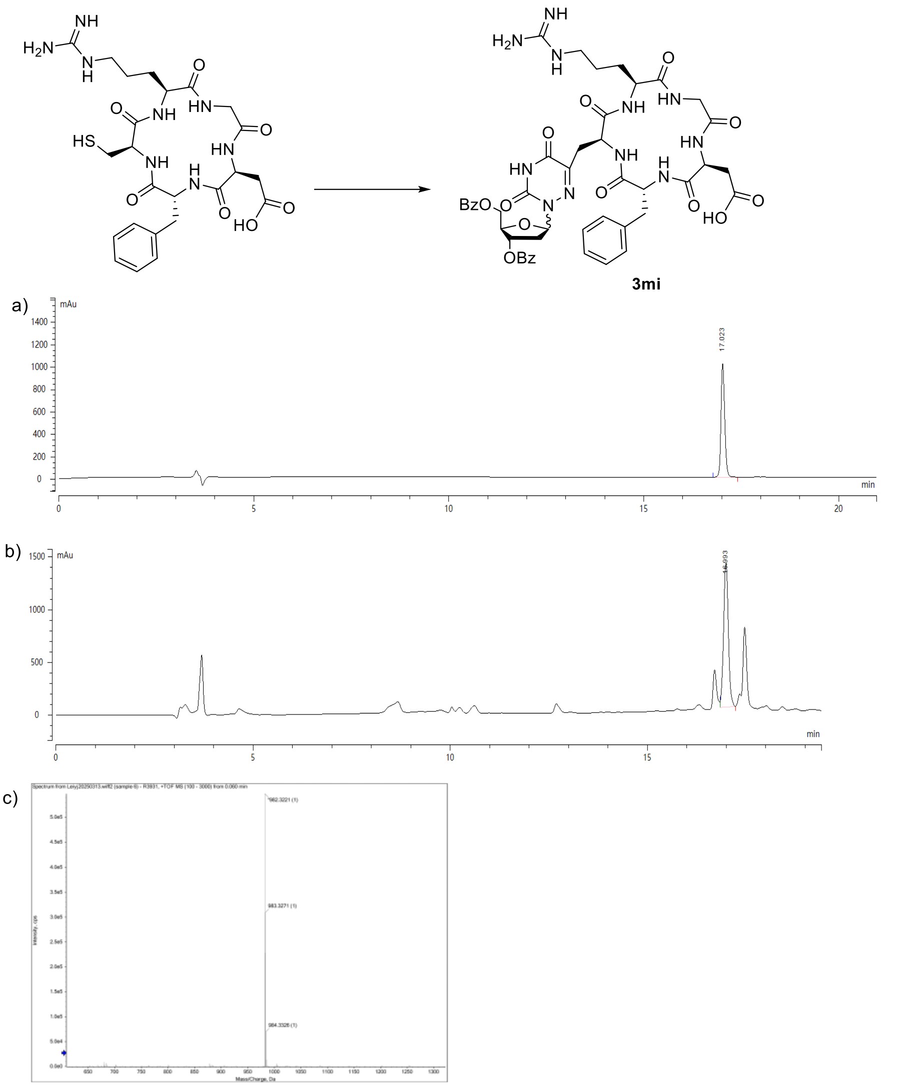

Figure S29. a) Analytical HPLC of **3mi.** b) Analytical HPLC trace of reaction system for synthesizing **3mi**. c) Ms spectrum of **3mi** (Calculated Mass $[M+H]^+$: 982.3690; Observed Mass $[M+H]^+$: 982.3221). Analyzing the purities was carried out on a UNIMICRO EasySep®-3030 using a Globalsil® C18 Column (100 Å, 5 µm, 4.6 mm × 250 mm). Linear gradients using 10% A/ 90% B to 75% A/ 25% B over 20 min.

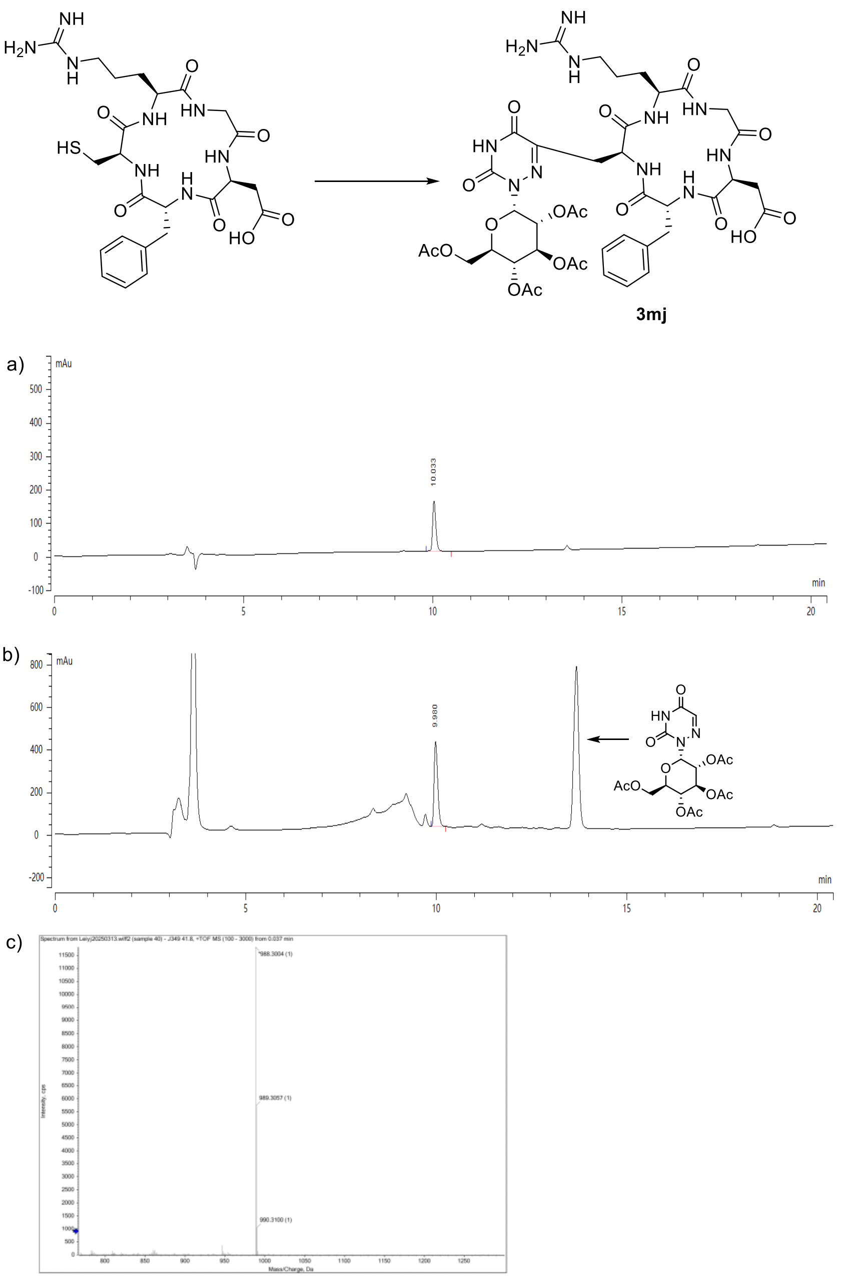

Figure S30. a) Analytical HPLC of **3mj.** b) Analytical HPLC trace of reaction system for synthesizing **3mj.** c) Ms spectrum of **3mj** (Calculated Mass $[M+H]^+$: 988.3643; Observed Mass $[M+H]^+$: 988.3004). Analyzing the purities was carried out on a UNIMICRO EasySep®-3030 using a Globalsil® C18 Column (100 Å, 5 µm, 4.6 mm × 250 mm). Linear gradients using 10% A/ 90% B to 75% A/ 25% B over 20 min.

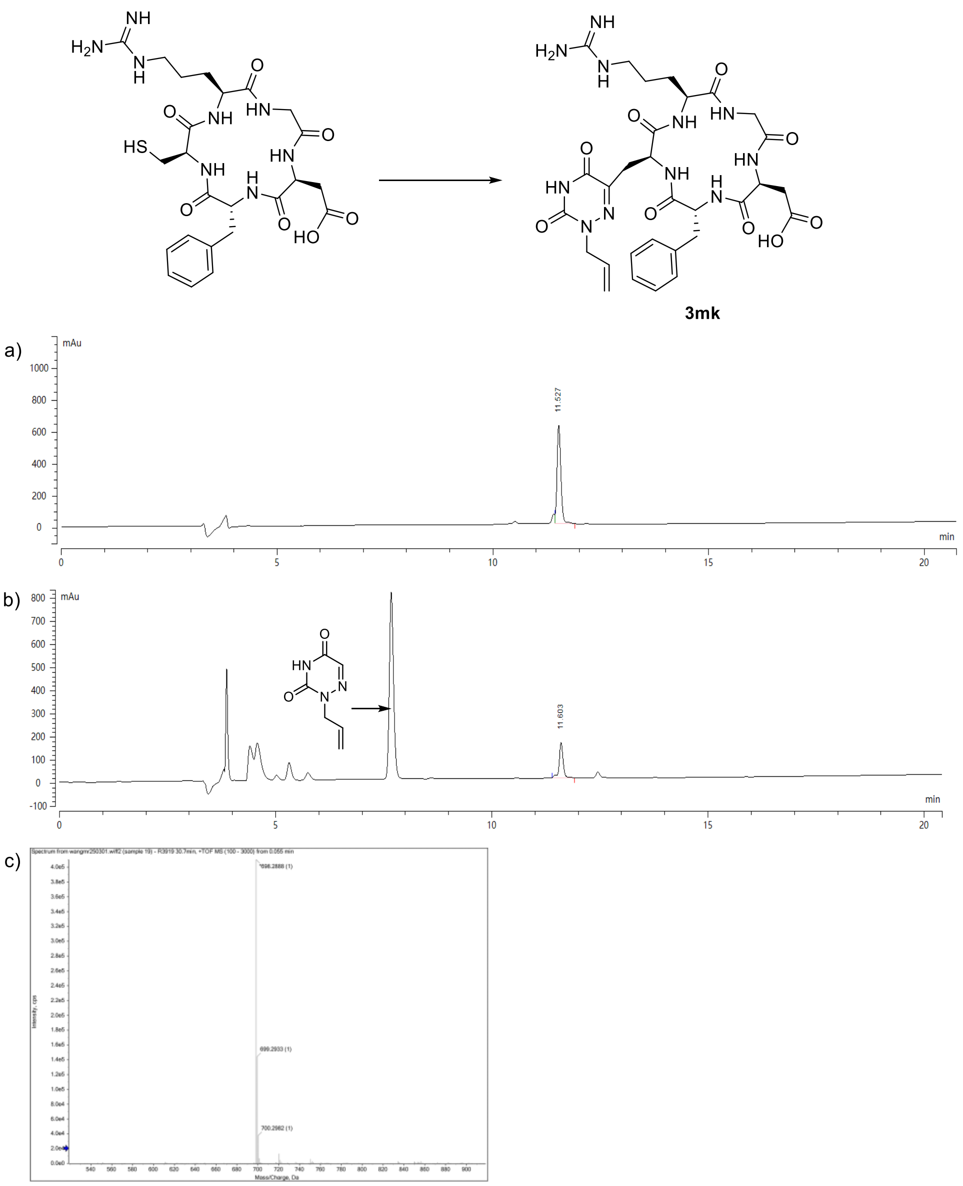

Figure S31. a) Analytical HPLC of **3mk.** b) Analytical HPLC trace of reaction system for synthesizing **3mk.** c) Ms spectrum of **3mk** (Calculated Mass $[M+H]^+$: 698.3005; Observed Mass $[M+H]^+$: 698.2888). Analyzing the purities was carried out on a UNIMICRO EasySep®-3030 using a Globalsil® C18 Column (100 Å, 5 µm, 4.6 mm × 250 mm). Linear gradients using 10% A/ 90% B to 75% A/ 25% B over 20 min.

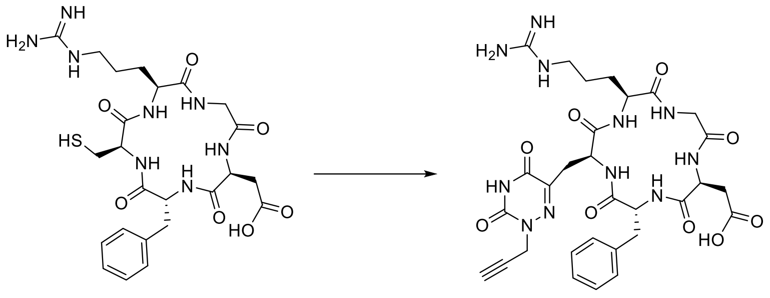

3ml

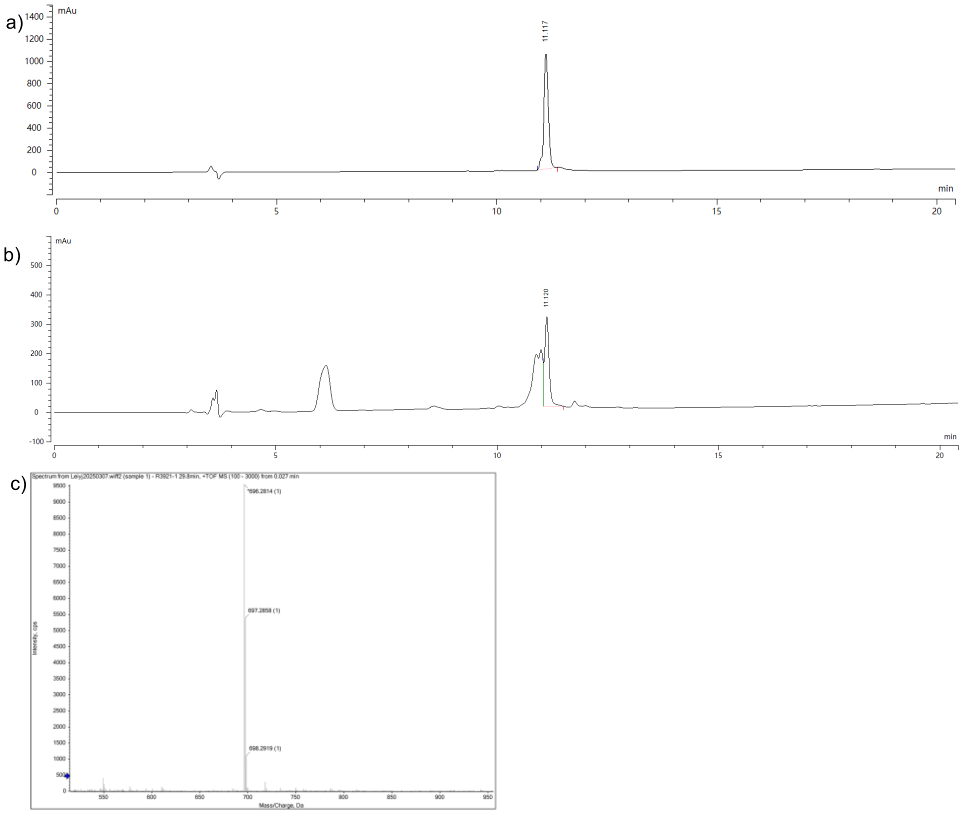

Figure S32. a) Analytical HPLC of **3ml.** b) Analytical HPLC trace of reaction system for synthesizing **3ml.** c) Ms spectrum of **3ml** (Calculated Mass $[M+H]^+$: 696.2849; Observed Mass $[M+H]^+$: 696.2814). Analyzing the purities was carried out on a UNIMICRO EasySep®-3030 using a Globalsil® C18 Column (100 Å, 5 μm, 4.6 mm × 250 mm). Linear gradients using 10% A/ 90% B to 75% A/ 25% B over 20 min.

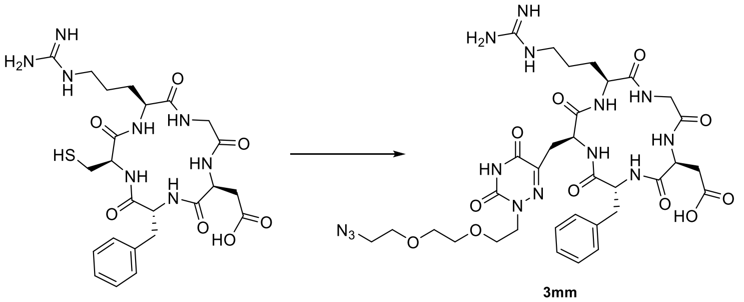

3mm

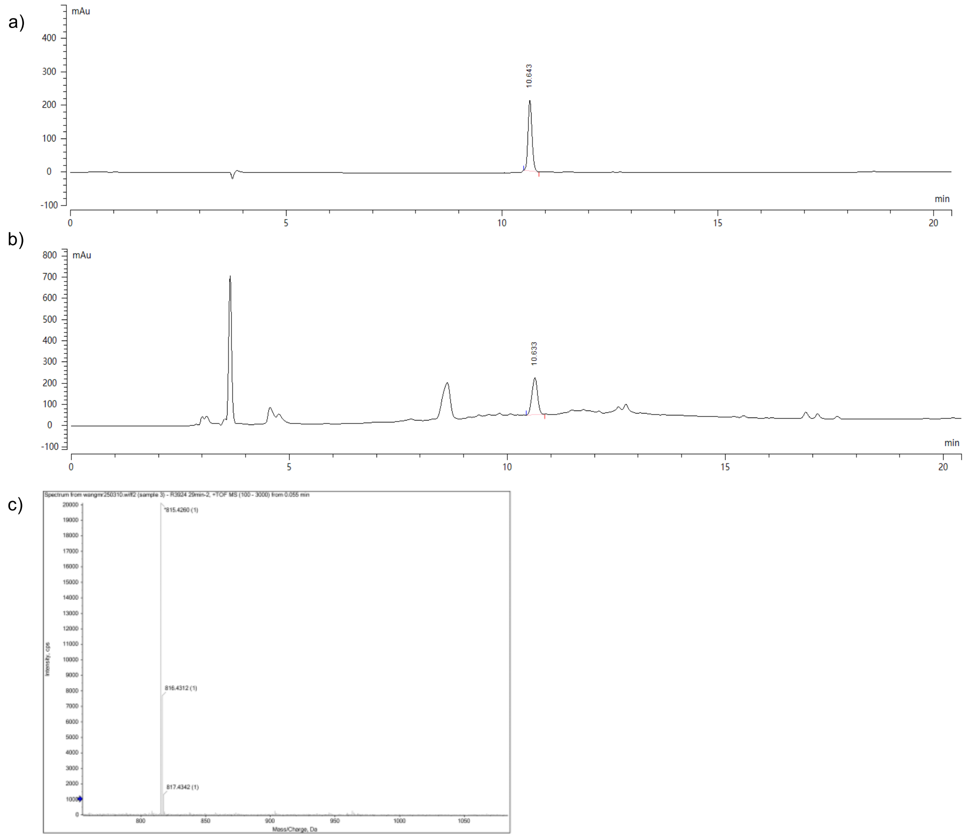

Figure S33. a) Analytical HPLC of **3mm.** b) Analytical HPLC trace of reaction system for synthesizing **3mm.** c) Ms spectrum of **3mm** (Calculated Mass $[M+H]^+$: 815.3541; Observed Mass $[M+H]^+$: 815.4260). Analyzing the purities was carried out on a UNIMICRO EasySep®-3030 using a Globalsil® C18 Column (100 Å, 5 μm, 4.6 mm × 250 mm). Linear gradients using 10% A/ 90% B to 75% A/ 25% B over 20 min.

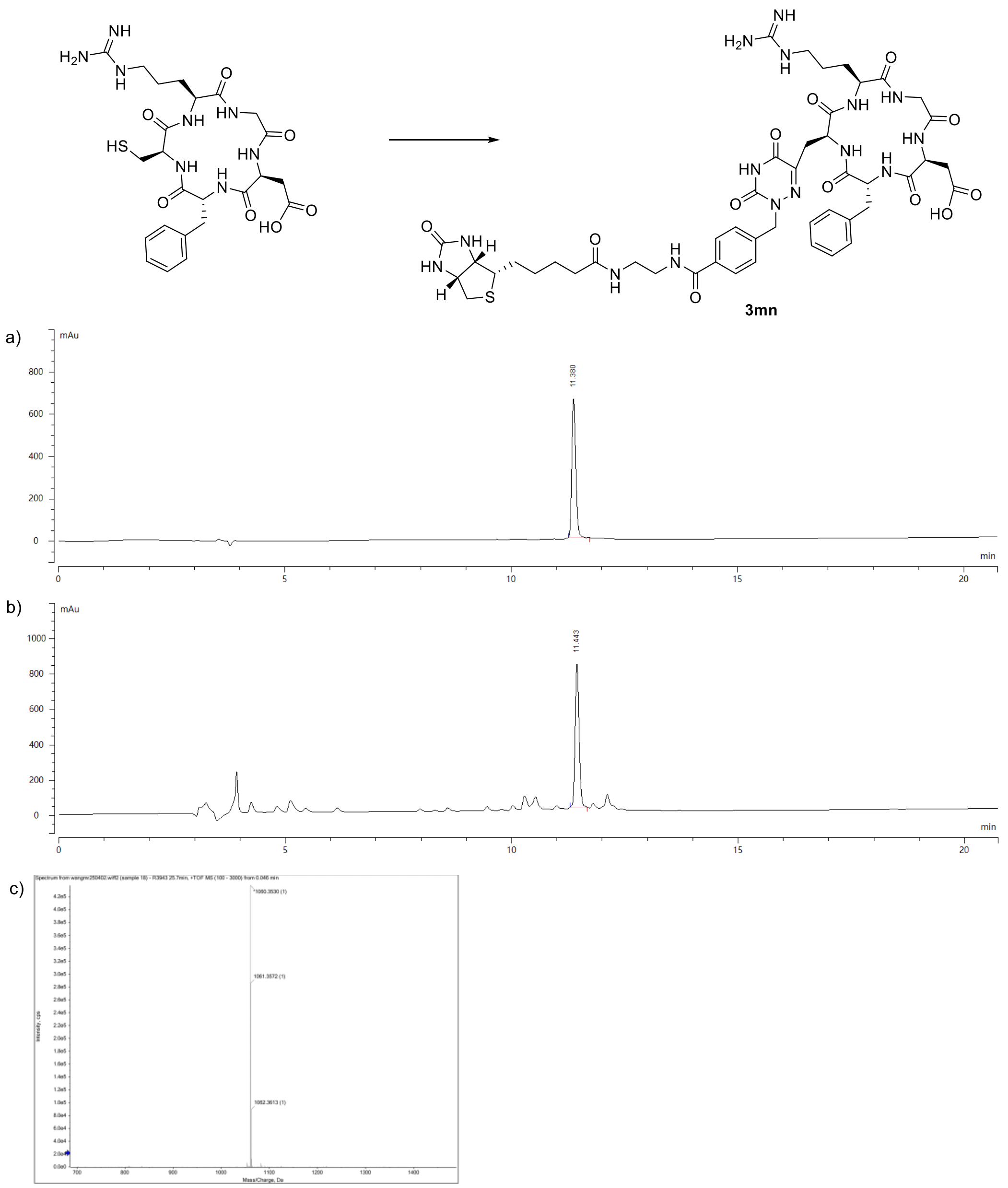

Figure S34. a) Analytical HPLC of **3mn.** b) Analytical HPLC trace of reaction system for synthesizing **3mn.** c) Ms spectrum of **3mn** (Calculated Mass $[M+H]^+$: 1060.4418; Observed Mass $[M+H]^+$:1060.3530). Analyzing the purities was carried out on a UNIMICRO EasySep®-3030 using a Globalsil® C18 Column (100 Å, 5 µm, 4.6 mm × 250 mm). Linear gradients using 10% A/ 90% B to 75% A/ 25% B over 20 min.

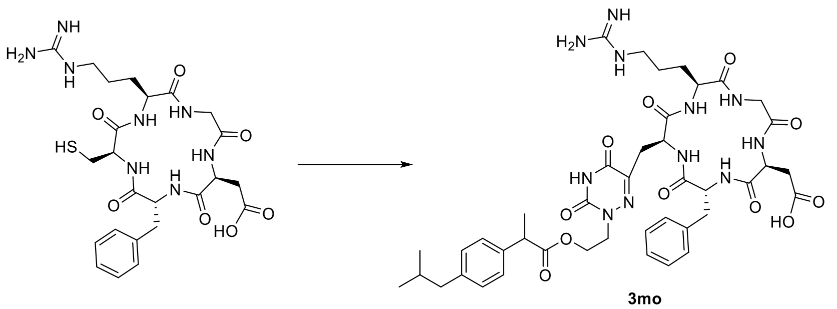

3mo

a)

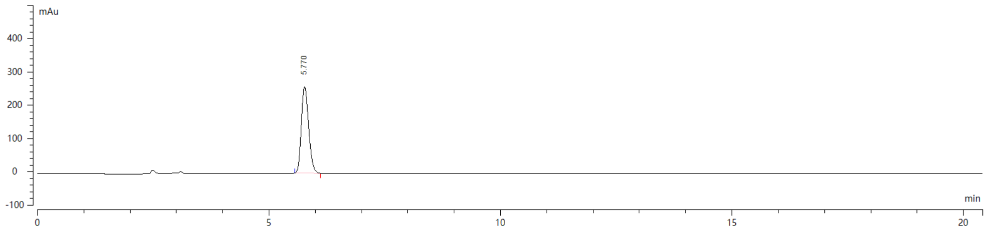

b)

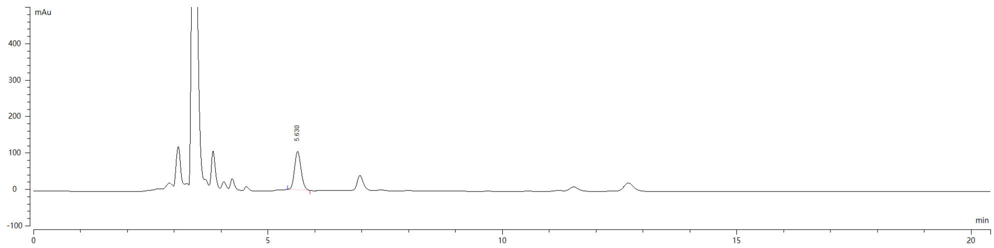

c)

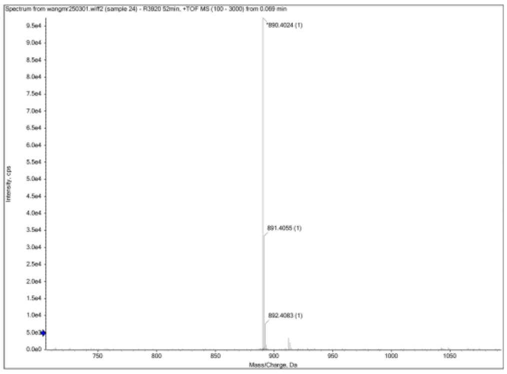

Figure S35. a) Analytical HPLC of **3mo.** b) Analytical HPLC trace of reaction system for synthesizing **3mo.** c) Ms spectrum of **3mo** (Calculated Mass $[M+H]^+$: 890.4156; Observed Mass $[M+H]^+$: 890.4024). Analyzing the purities was carried out on a UNIMICRO EasySep®-3030 using a Globalsil® C18 Column (100 Å, 5 µm, 4.6 mm × 250 mm). Isocratic elution was performed using 50% A/ 50% B over 20 min.

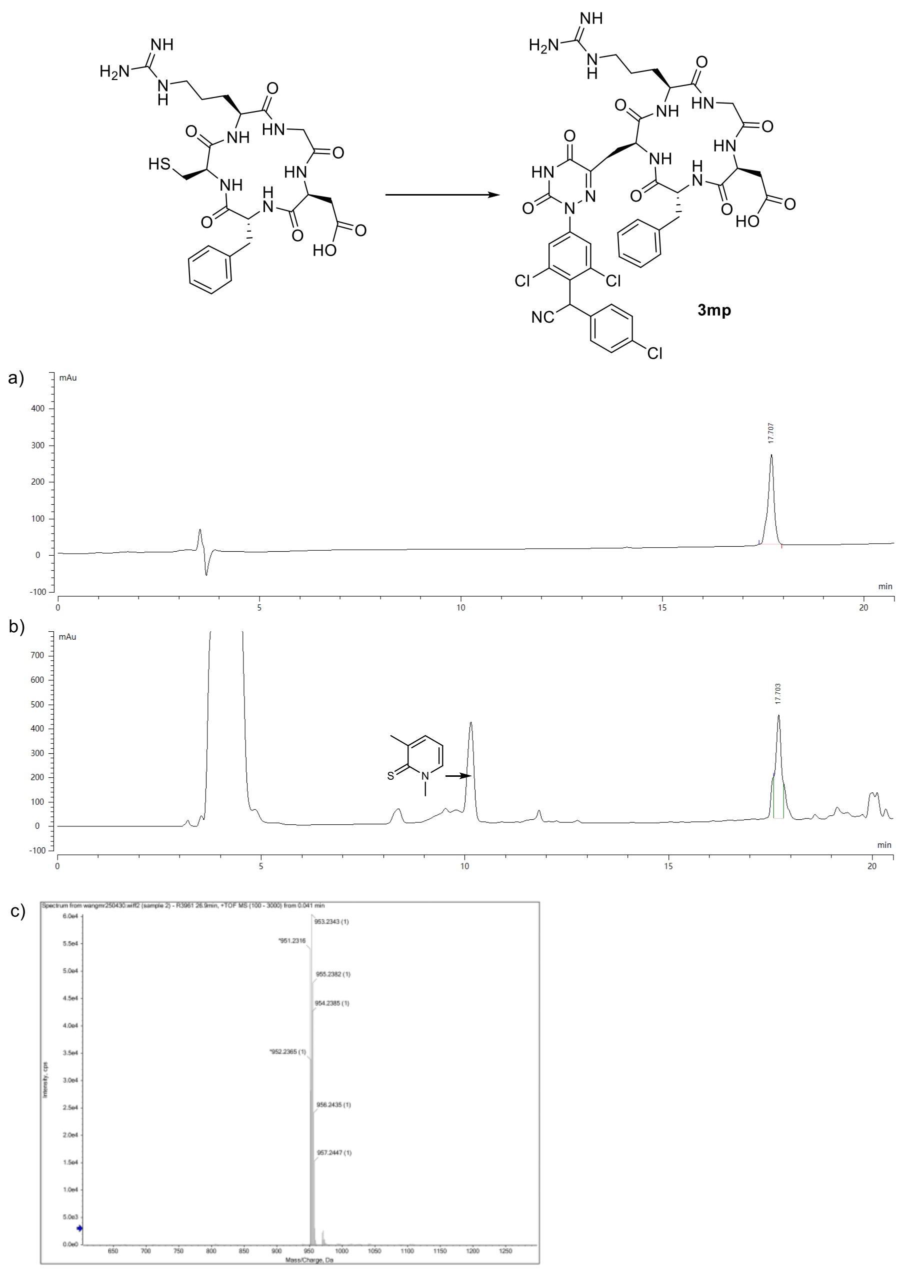

Figure S36. a) Analytical HPLC of **3mp.** b) Analytical HPLC trace of reaction system for synthesizing **3mp.** c) Ms spectrum of **3mp** (Calculated Mass $[M+H]^+$: 951.2258; Observed Mass $[M+H]^+$: 951.2316). Analyzing the purities was carried out on a UNIMICRO EasySep®-3030 using a Globalsil® C18 Column (100 Å, 5 μm, 4.6 mm × 250 mm). Linear gradients using 10% A/ 90% B to 75% A/ 25% B over 20 min.

Analytical data for **5ba-5bg, 5ha, 5hc, 5he, 5hh**

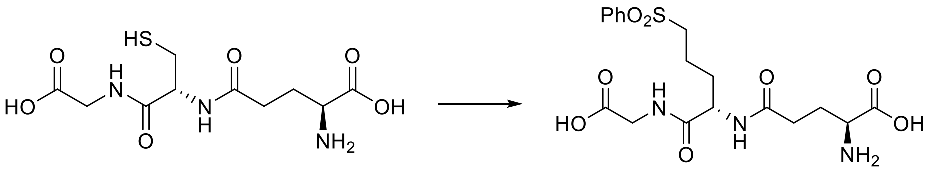

1b **5ba**

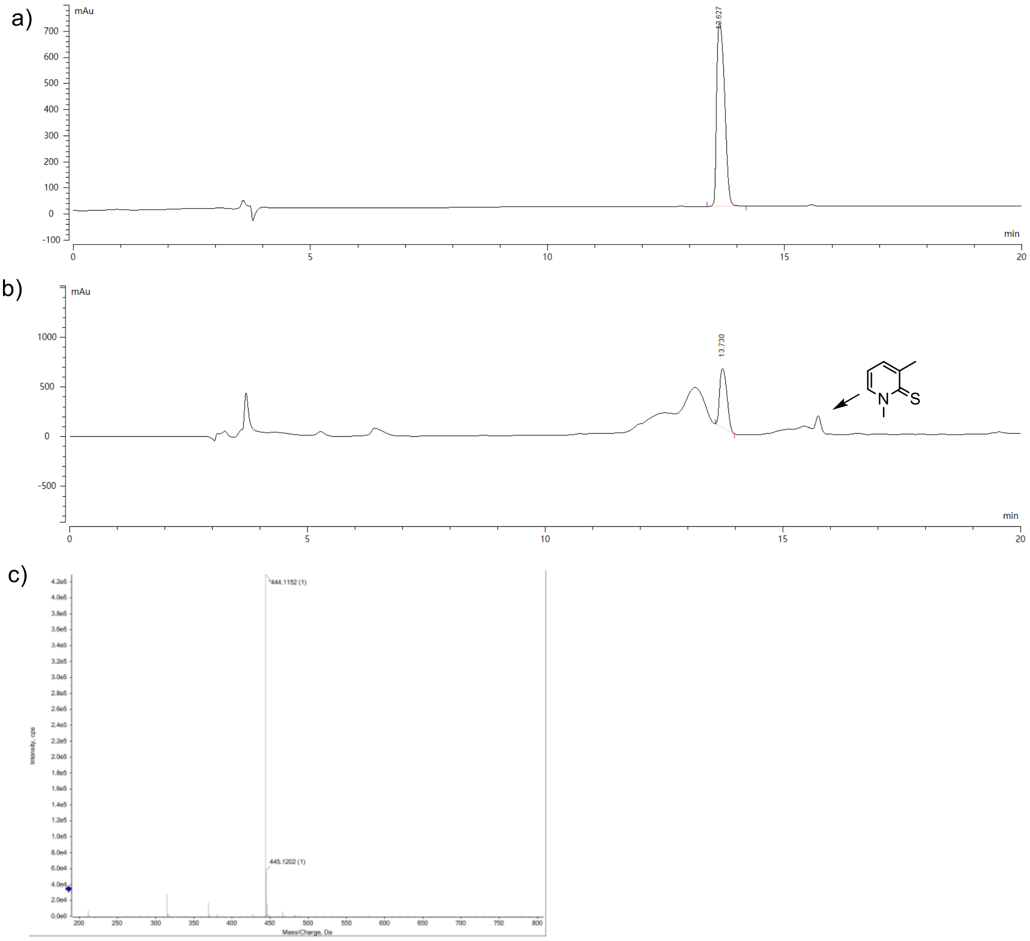

Figure S37. a) Analytical HPLC of **5ba.** b) Analytical HPLC trace of reaction system for synthesizing **5ba.** c) Ms spectrum of **5ba** (Calculated Mass $[M+H]^+$: 444.1436; Observed Mass $[M+H]^+$: 444.1152). Analyzing the purities was carried out on a UNIMICRO EasySep®-3030 using a Globalsil® C18-AP column (120 Å, 5 µm, 4.6 mm × 250 mm). Linear gradients using 5% A/ 95% B to 50% A/ 50% B over 25 min.

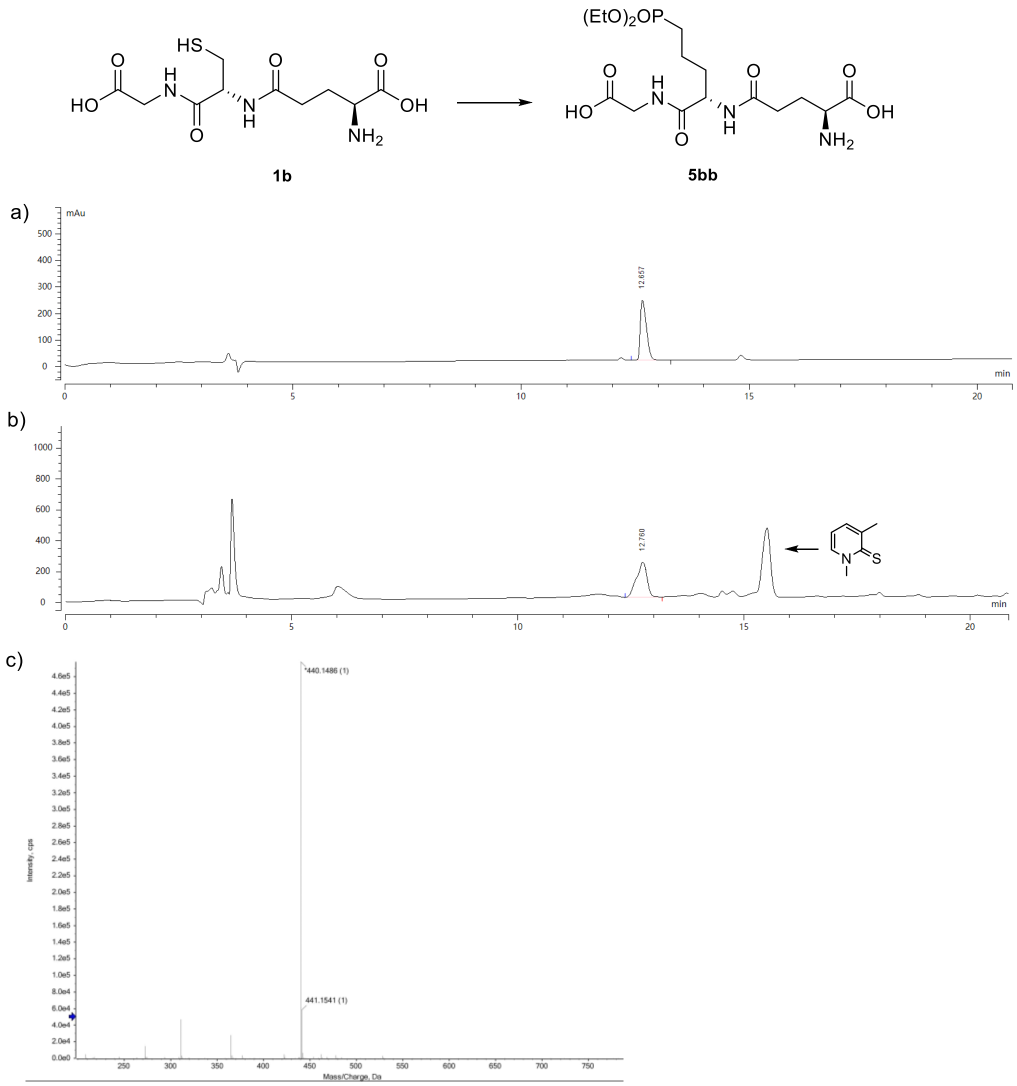

Figure S38. a) Analytical HPLC of **5bb.** b) Analytical HPLC trace of reaction system for synthesizing **5bb.** c) MS spectrum of **5bb** (Calculated Mass $[M+H]^+$: 440.1793; Observed Mass $[M+H]^+$: 440.1486). Analyzing the purities was carried out on a UNIMICRO EasySep®-3030 using a Globalsil® C18-AP column (120 Å, 5 µm, 4.6 mm × 250 mm). Linear gradients using 5% A/ 95% B to 50% A/ 50% B over 25 min.

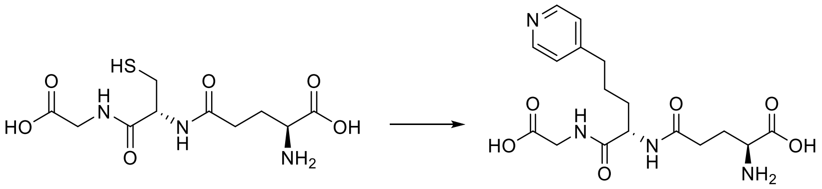

1b **5bc**

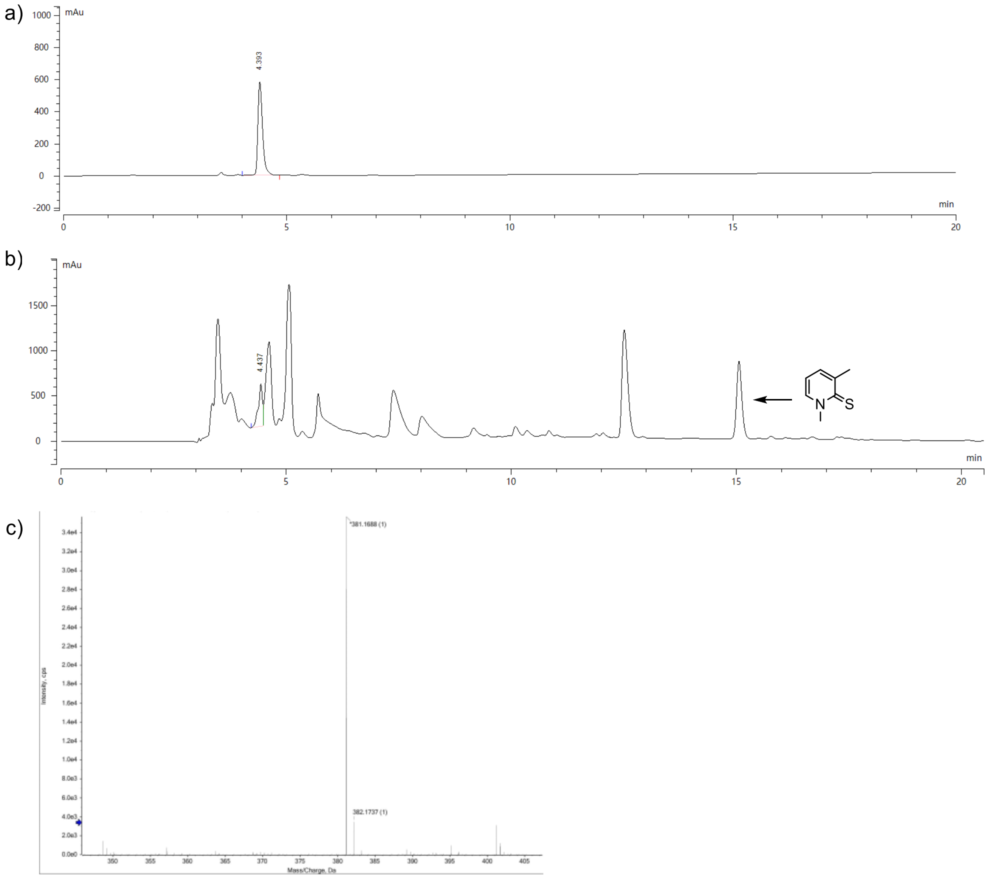

Figure S39. a) Analytical HPLC of **5bc.** b) Analytical HPLC trace of reaction system for synthesizing **5bc.** c) MS spectrum of **5bc** (Calculated Mass $[M+H]^+$: 381.1768; Observed Mass $[M+H]^+$: 381.1688). Analyzing the purities was carried out on a UNIMICRO EasySep®-3030 using a Globalsil® C18-AP column (120 Å, 5 μm, 4.6 mm × 250 mm). Linear gradients using 5% A/ 95% B to 50% A/ 50% B over 25 min.

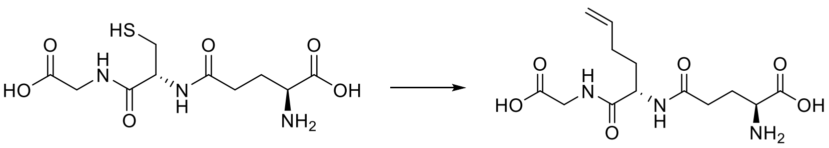

1b **5bd**

a)

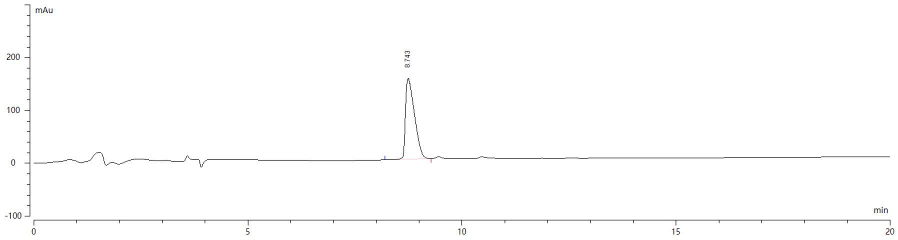

b)

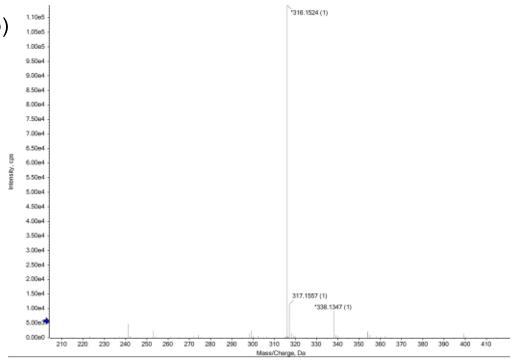

Figure S40. a) Analytical HPLC of **5bd.** b) MS spectrum of **5bd** (Calculated Mass $[M+H]^+$: 316.1504; $[M+Na]^+$: 338.1323; Observed Mass $[M+H]^+$: 316.1524; $[M+Na]^+$: 338.1347). Analyzing the purities was carried out on a UNIMICRO EasySep®-3030 using a Globalsil® C18-AP column (120 Å, 5 μm, 4.6 mm × 250 mm). Linear gradients using 5% A/ 95% B to 50% A/ 50% B over 25 min.

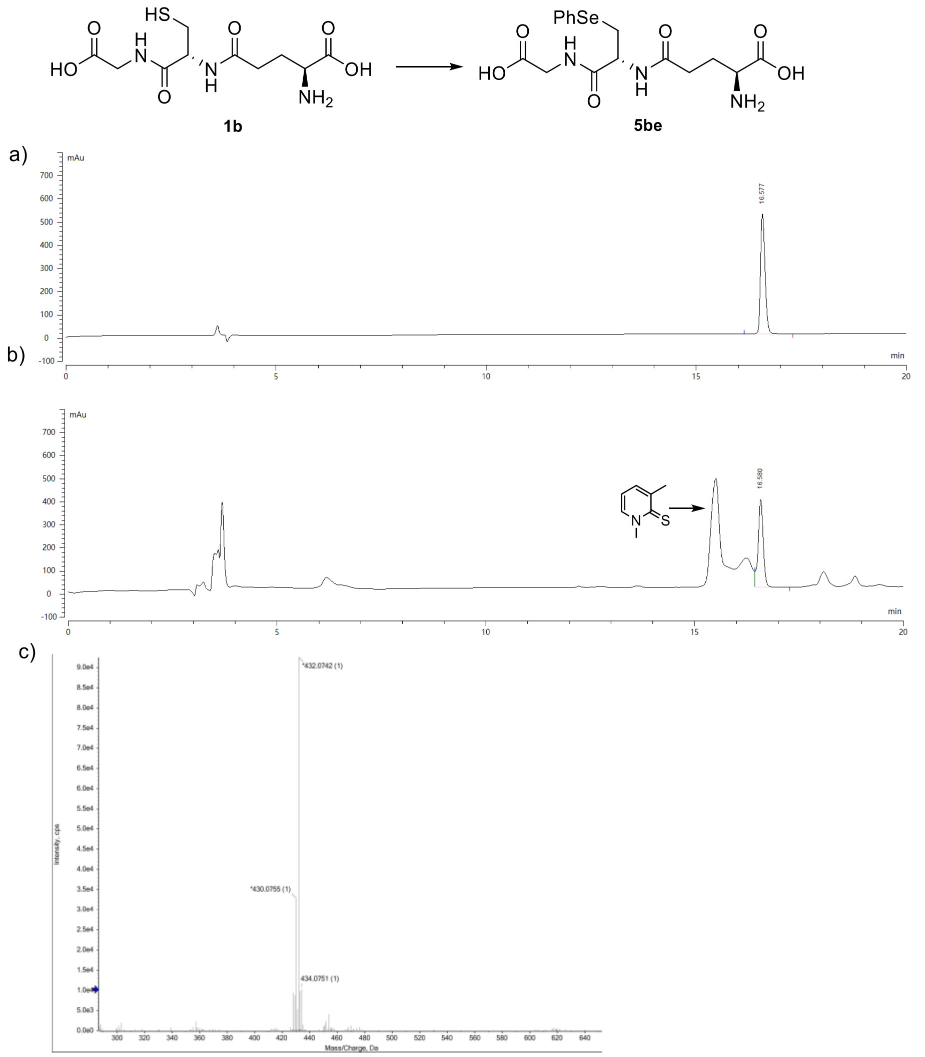

Figure S41. a) Analytical HPLC of **5be.** b) Analytical HPLC trace of reaction system for synthesizing **5be.** c) MS spectrum of **5be** (Calculated Mass $[M+H]^+$: 432.0669; Observed Mass $[M+H]^+$: 432.0742). Analyzing the purities was carried out on a UNIMICRO EasySep®-3030 using a Globalsil® C18-AP column (120 Å, 5 µm, 4.6 mm × 250 mm). Linear gradients using 5% A/ 95% B to 50% A/ 50% B over 25 min.

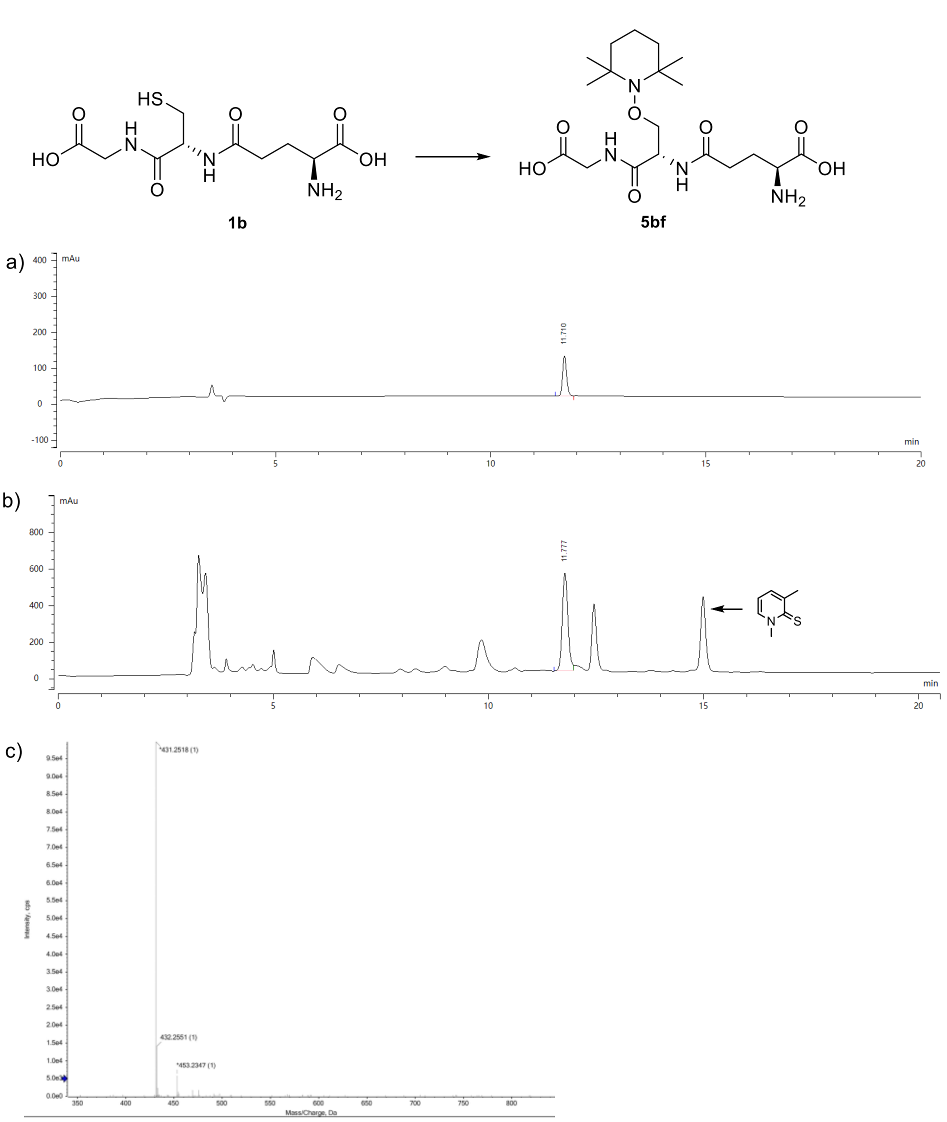

Figure S42. a) Analytical HPLC of **5bf.** b) Analytical HPLC trace of reaction system for synthesizing **5bf.** c) MS spectrum of **5bf** (Calculated Mass $[M+H]^+$: 431.2501; $[M+Na]^+$: 453.2320; Observed Mass $[M+H]^+$: 431.2518; $[M+Na]^+$: 453.2347). Analyzing the purities was carried out on a UNIMICRO EasySep®-3030 using a Globalsil® C18-AP column (120 Å, 5 μm, 4.6 mm × 250 mm). Linear gradients using 5% A/ 95% B to 50% A/ 50% B over 25 min.

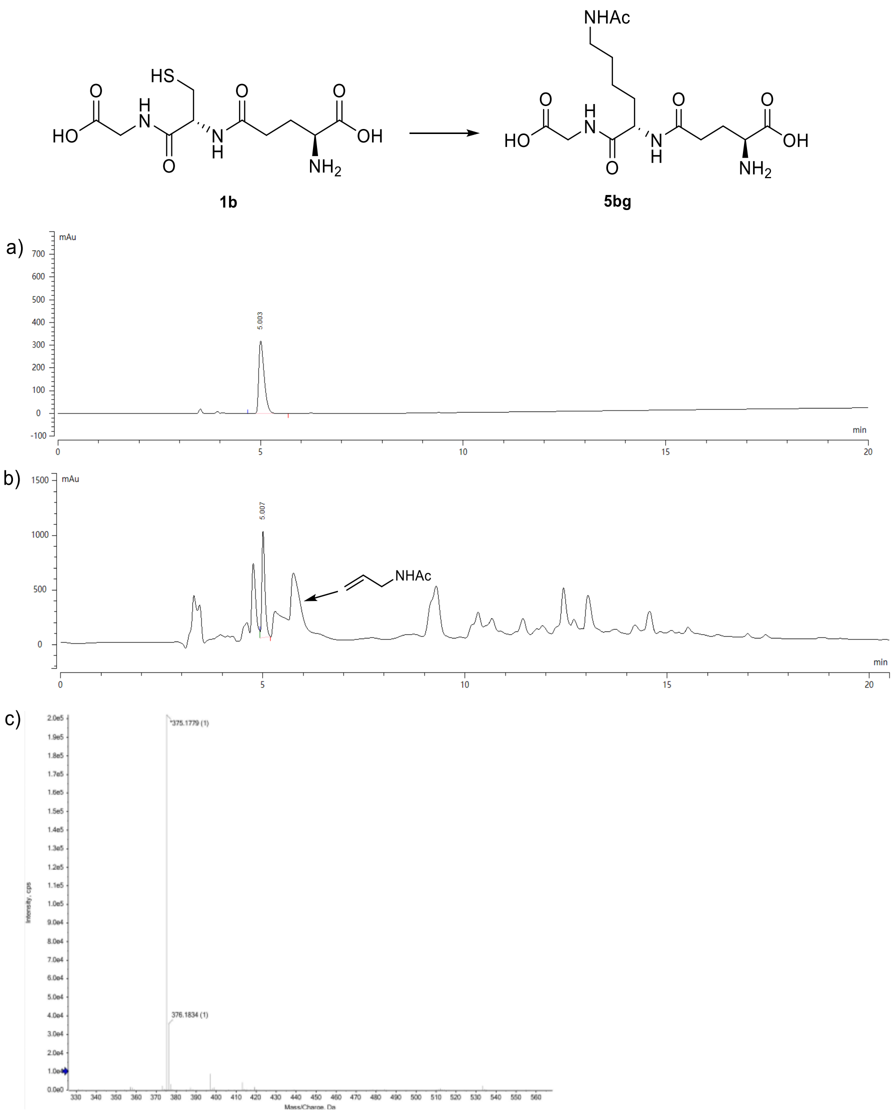

Figure S43. a) Analytical HPLC of **5bg.** b) Analytical HPLC trace of reaction system for synthesizing **5bg.** c) MS spectrum of **5bg** (Calculated Mass $[M+H]^+$: 375.1875; Observed Mass $[M+H]^+$: 375.1779). Analyzing the purities was carried out on a UNIMICRO EasySep®-3030 using a Globalsil® C18-AP column (120 Å, 5 µm, 4.6 mm × 250 mm). Linear gradients using 5% A/ 95% B to 50% A/ 50% B over 25 min.

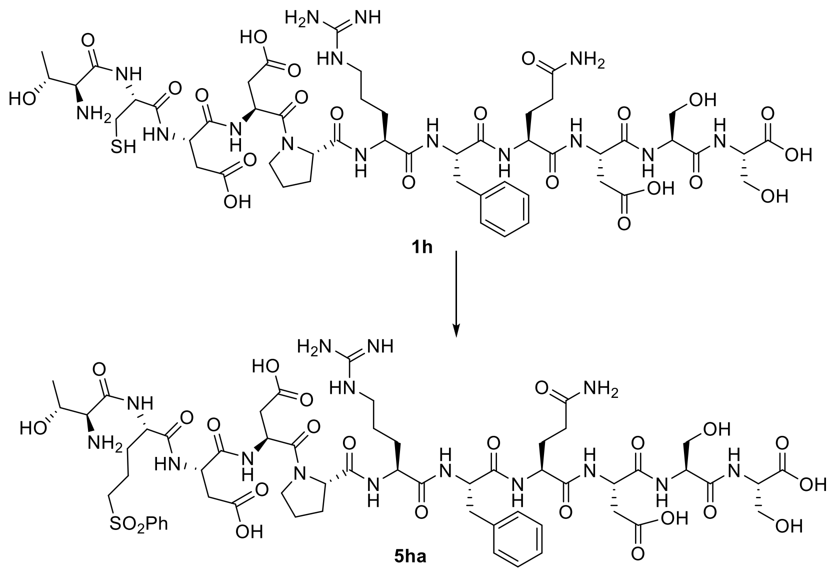

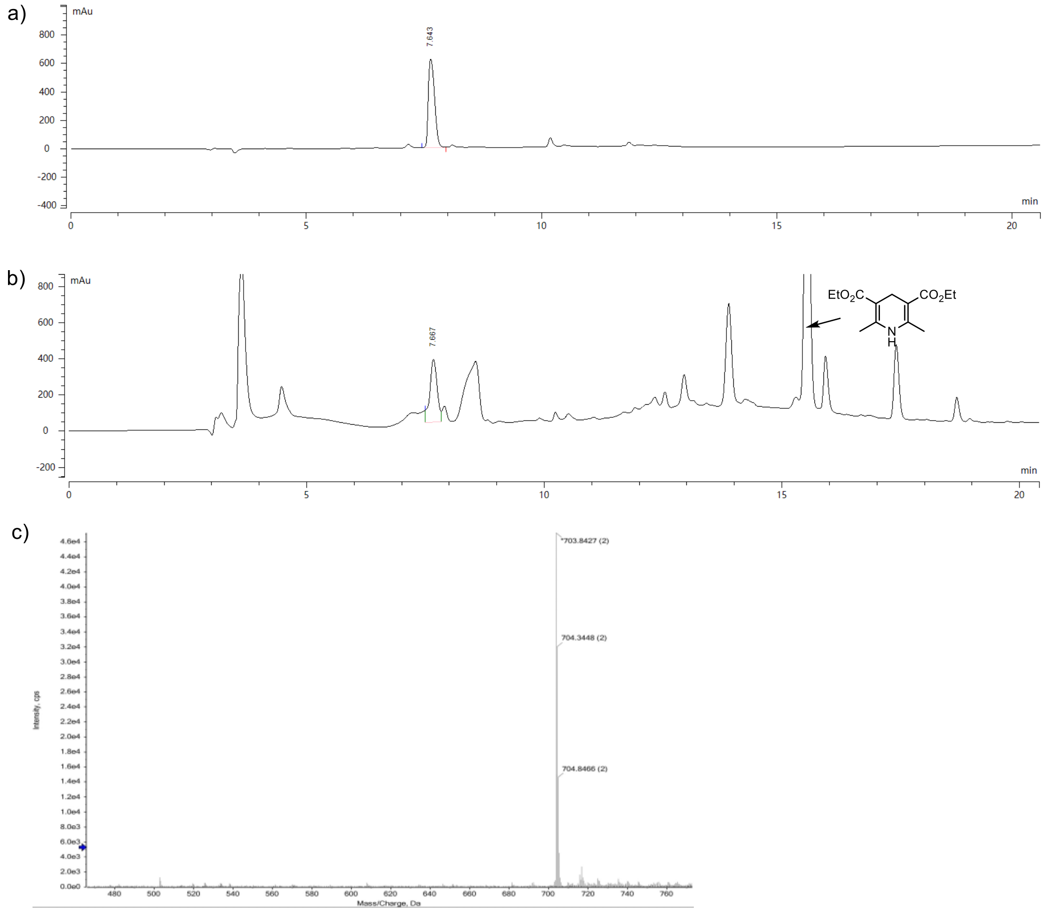

Figure S44. a) Analytical HPLC of **5ha**. b) Analytical HPLC trace of reaction system for synthesizing **5ha** using Hantzch ester as reductant**.** c) MS spectrum of **5ha** (Calculated Mass $[M+2H]^{2+}$:703.7801; Observed Mass $[M+2H]^{2+}$:703.8427). Analyzing the purities was carried out on a UNIMICRO EasySep®-3030 using a Globalsil® C18-AP column (120 Å, 5 µm, 4.6 mm × 250 mm). Linear gradients using 10% A/ 90% B to 75% A/ 25% B over 20 min.

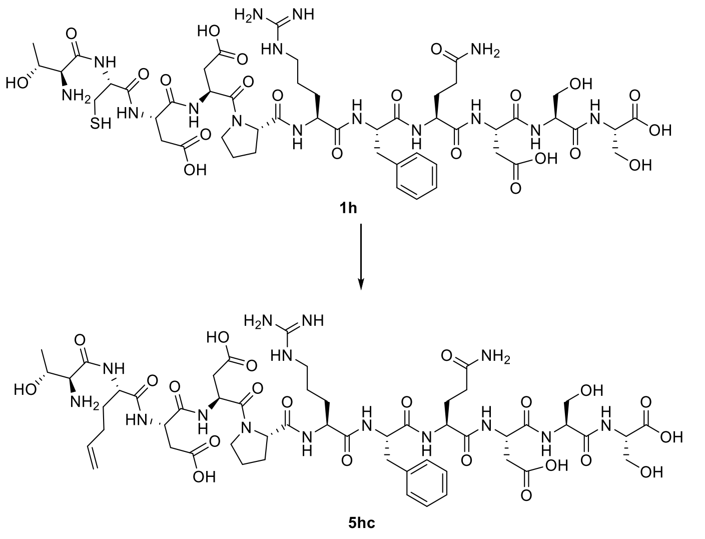

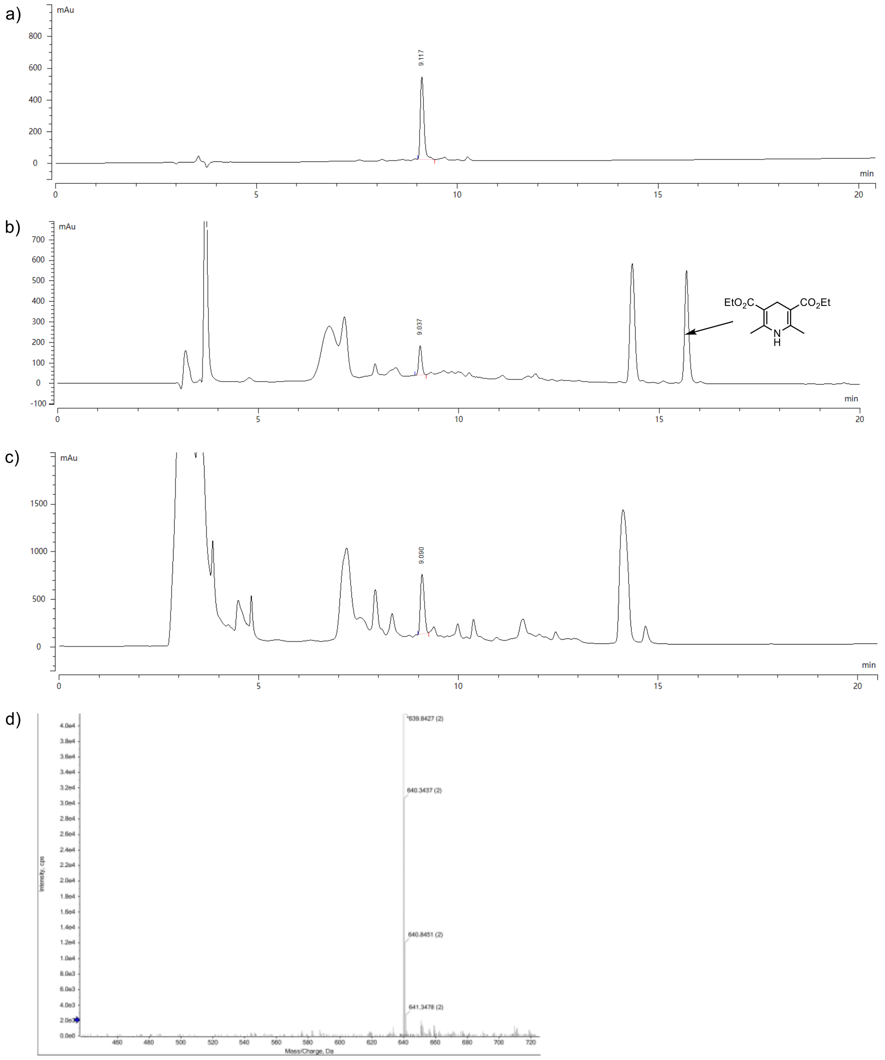

Figure S45. a) Analytical HPLC of **5hc.** b) Analytical HPLC trace of reaction system for synthesizing **5hc** using Hantzch ester as reductant. c) Analytical HPLC trace of reaction system for synthesizing **5hc** using NADH as reductant. d) MS spectrum of **5hc** (Calculated Mass $[M+2H]^{2+}$:639.7835; Observed Mass $[M+2H]^{2+}$:639.8427). Analyzing the purities was carried out on a UNIMICRO EasySep®-3030 using a Globalsil® C18-AP column (120 Å, 5 µm, 4.6 mm × 250 mm). Linear gradients using 10% A/ 90% B to 75% A/ 25% B over 20 min.

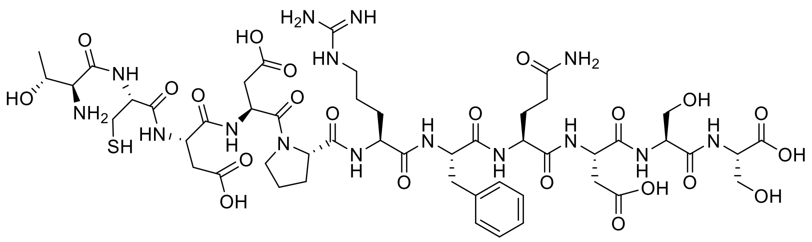

1h

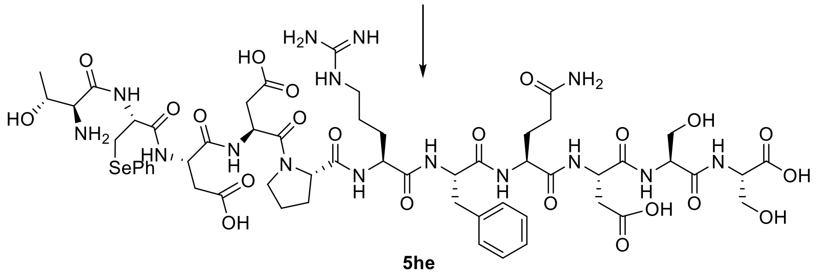

5he

a)

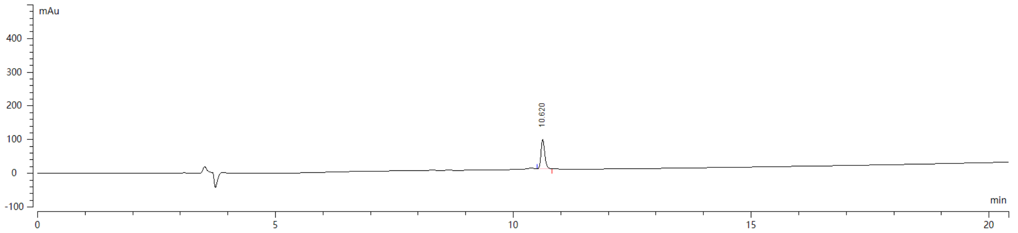

b)

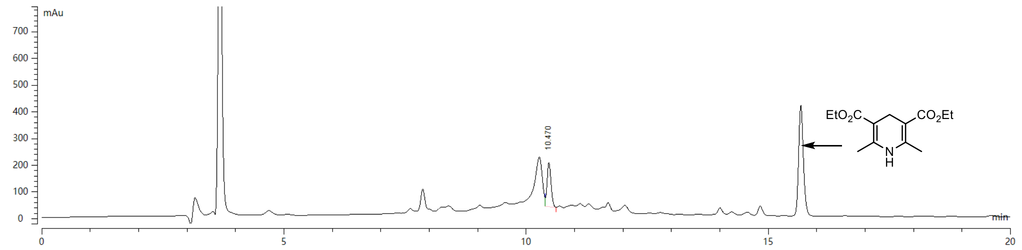

c)

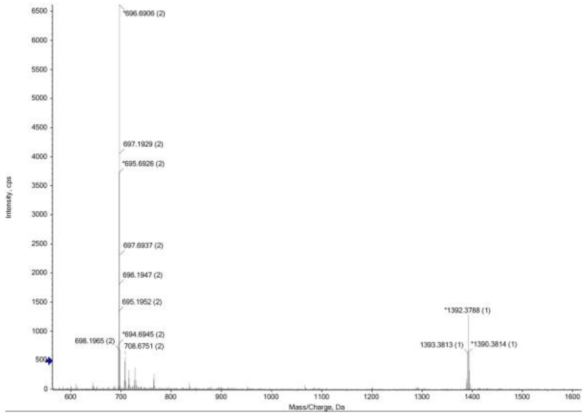

Figure S46. a) Analytical HPLC of **5he.** b) Analytical HPLC trace of reaction system for synthesizing **5he** using Hantzch ester as reductant. c) MS spectrum of **5he** (Calculated Mass $[M+H]^+$:1392.4731; $[M+2H]^{2+}$:696.7422; Observed Mass $[M+H]^+$:1392.3788; $[M+2H]^{2+}$:696.6906). Analyzing the purities was carried out on a UNIMICRO EasySep®-3030 using a Globalsil® C18-AP column (120 Å, 5 μm, 4.6 mm × 250 mm). Linear gradients using 10% A/ 90% B to 75% A/ 25% B over 20 min.

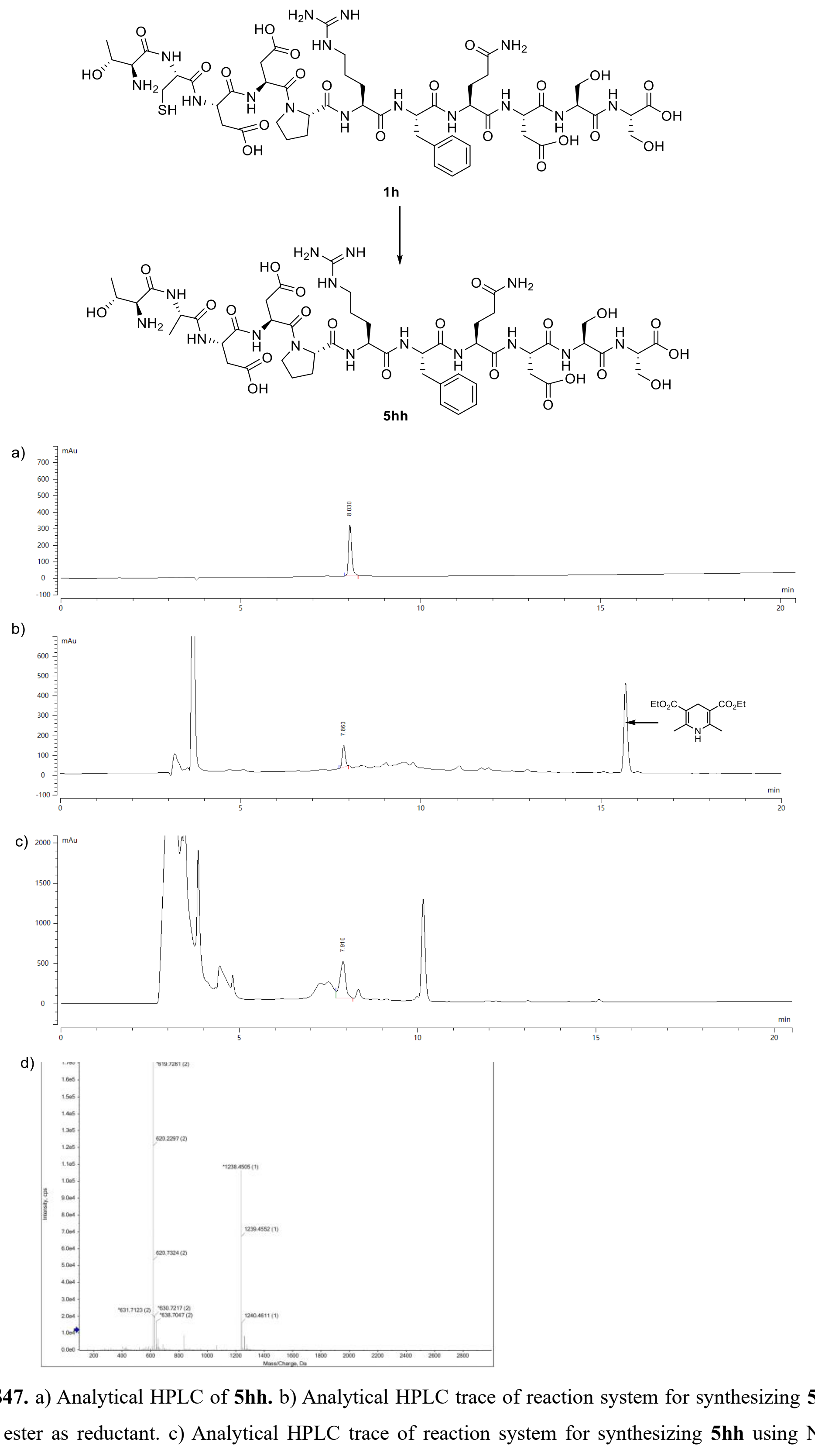

Figure S47. a) Analytical HPLC of **5hh.** b) Analytical HPLC trace of reaction system for synthesizing **5hh** using Hantzch ester as reductant. c) Analytical HPLC trace of reaction system for synthesizing **5hh** using NADH as reductant. d) MS spectrum of **5hh** (Calculated Mass $[M+H]^+$:1238.5284; $[M+2H]^{2+}$:619.7679; Observed Mass $[M+H]^+$:1238.4505; $[M+2H]^{2+}$:619.7281). Analyzing the purities was carried out on a UNIMICRO EasySep®-3030 using a Globalsil® C18-AP column (120 Å, 5 μm, 4.6 mm × 250 mm). Linear gradients using 10% A/ 90% B to 75% A/ 25% B over 20 min.

6. Mechanistic studies

6.1 Radical trapping experiment

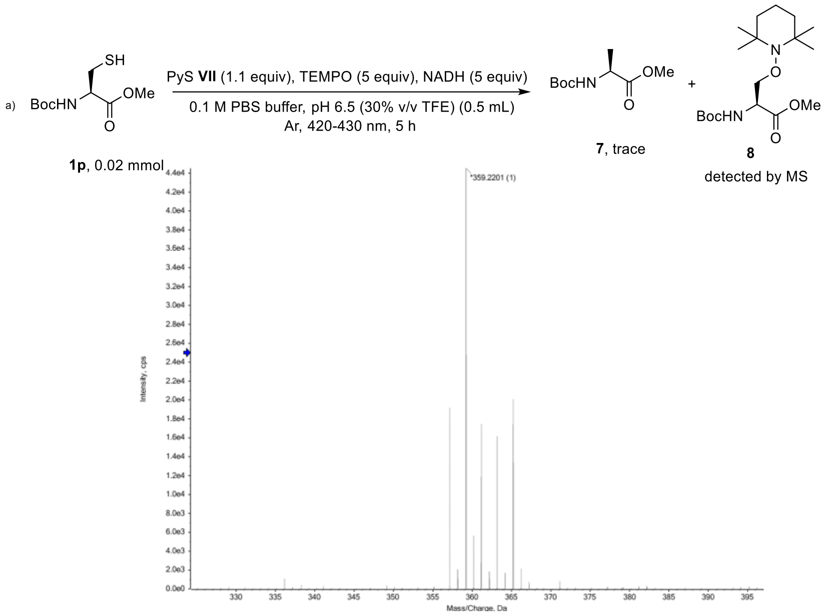

Figure S48. Radical trapping of the reaction condition using NADH as reductant **(**Calculated Mass $[M+H]^+$:359.2541; Observed Mass $[M+H]^+$:359.2201)

6.2 Chirality verification of alanyl radical

Chiral analysis was carried out on an Agilent 1260 Infinity II HPLC using a CHIRALPAK® AD-H Column (5 μm, 4.6 mm × 250 mm), Linear gradients using hexane/i-PrOH = 9/1, flow rate = 1.0 mL/min, detection at 254 nm.

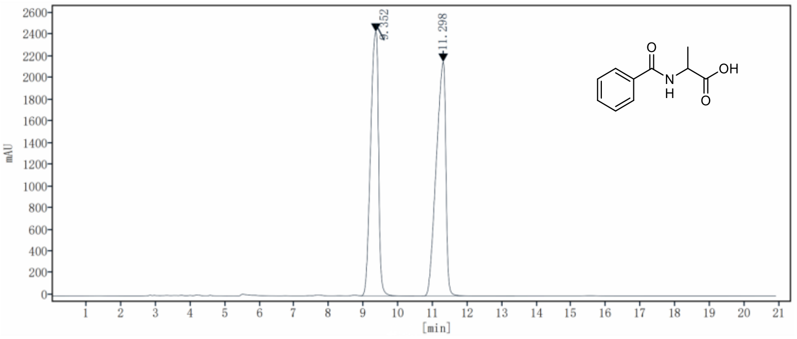

Entry	Retention time (min)	Area	Area (%)	Height
1	9.352	38586.13	48.12	2434.7
2	11.298	41602.64	51.88	2157.0

Figure S49. Chiral HPLC analysis of ***DL*-Bz-Ala-OH**

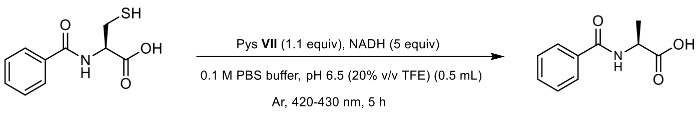

1q, 0.02 mmol

9, 95% yield, >99% ee

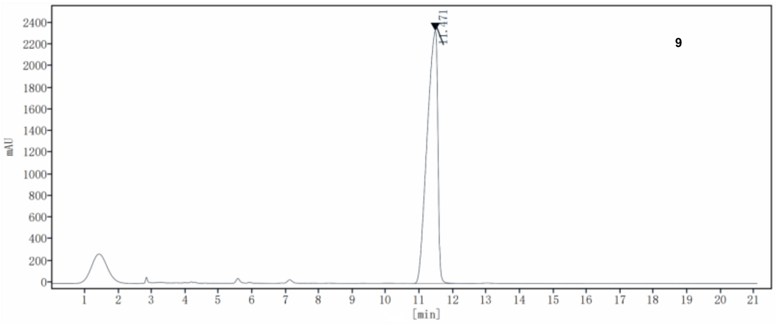

Entry	Retention time (min)	Area	Area (%)	Height
1	11.471	51579.56	100	2337.60

Figure S50. Chiral HPLC analysis of product **9**

6.3 Analysis of UV-vis absorption spectra

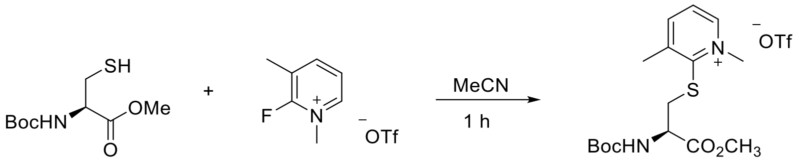

1p 1.2 equiv **10**, 88% yield

1p and Pys **VII** were reacted in acetonitrile solution for 1 hour, followed by recycling preprative HPLC system (Japan Analytical Industry Co.,Ltd LaboACE LC-5060 Plus II, ethyl acetate) separation and purification, yields the S_NAr product **10**.

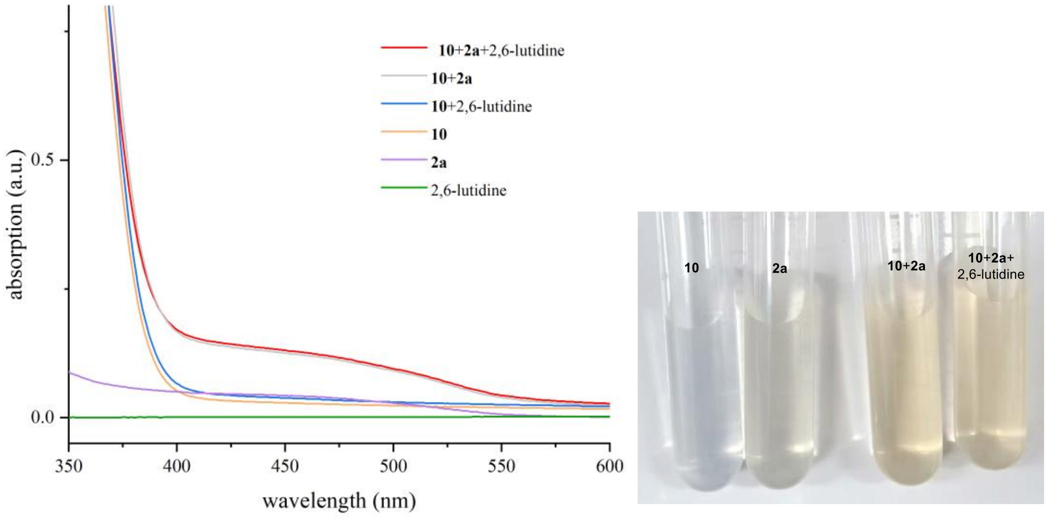

Figure S51. UV-vis absorption spectra

The UV-vis absorption spectra were recorded on a SHIMADZU UV-2600 spectrophotometer using 1 mm path length quartz cuvettes under the same buffer conditions. Solutions of compound **10** (3×10^{-3} mol/L), compound **2a** (3×10^{-3} mol/L), a mixture of **10** and **2a** (3×10^{-3} mol/L, **10**:**2a** = 1:2), and a mixture of **10**, **2a**, and 2,6-lutidine (3×10^{-3} mol/L, **10**:**2a**:2,6-lutidine = 1:2:3) were prepared in 0.2 M PBS buffer (pH 8.0, 20% MeCN v/v), respectively. Upon mixing compounds **10** and **2a**, the UV-Vis absorption spectrum exhibited a bathochromic shift into the visible region. The

resulting solution displayed a distinct yellow color, consistent with the formation of an electron donor-acceptor (EDA) complex.

6.4 Light/dark experiment

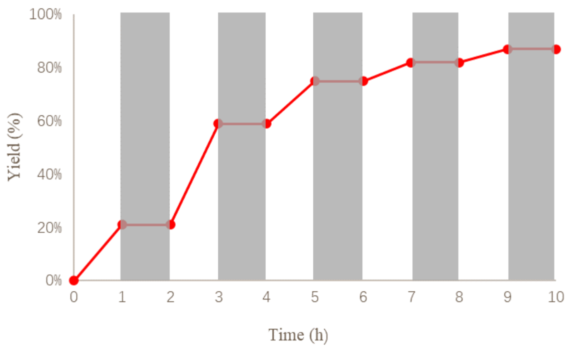

Figure S52. Light/dark experiment

To an oven-dried 10 mL quartz test tube with a stirring bar was added **1a** (0.02 mmol), **2a** (2 equiv), pyridinium salt **VII** (1.1 equiv) and 2,6-lutidine (3 equiv). Then, air was withdrawn and backfilled with Ar (three times). 1 mL PBS buffer (0.2 M, pH 8.0, 9% v/v MeCN) was added. The mixture was transferred to a violet LED photoreactor (100-W, 400 nm) and kept in the dark in one-hour intervals. The yield was determined by HPLC.

6.5 Proposed Mechanism

Based on our mechanistic studies and previous reports (*ACS Cent. Sci.* **2023**, *9*, 405; *Green Chem.* **2023**, *25*, 7971), we proposed a possible mechanism (Fig. S53a): Initially, Cys undergoes S_NAr with Mukaiyama reagent to form a Cys-Mukaiyama reagent adduct. This adduct then forms an EDA complex with compound **2**. Under 400 nm LED irradiation, SET occurs within the EDA complex, generating radical intermediate **A** and radical cation **11**. The subsequent β-scission cleaves the C-S bond and releases 1,3-dimethylpyridine-2(1H)-thione, alone with the formation of an Ala radical. The Ala radical adds to intermediate **11**, followed by a 1,2-H shift, yielding carbocation intermediate **13**. Deprotonation of **13** affords the final product **3**.

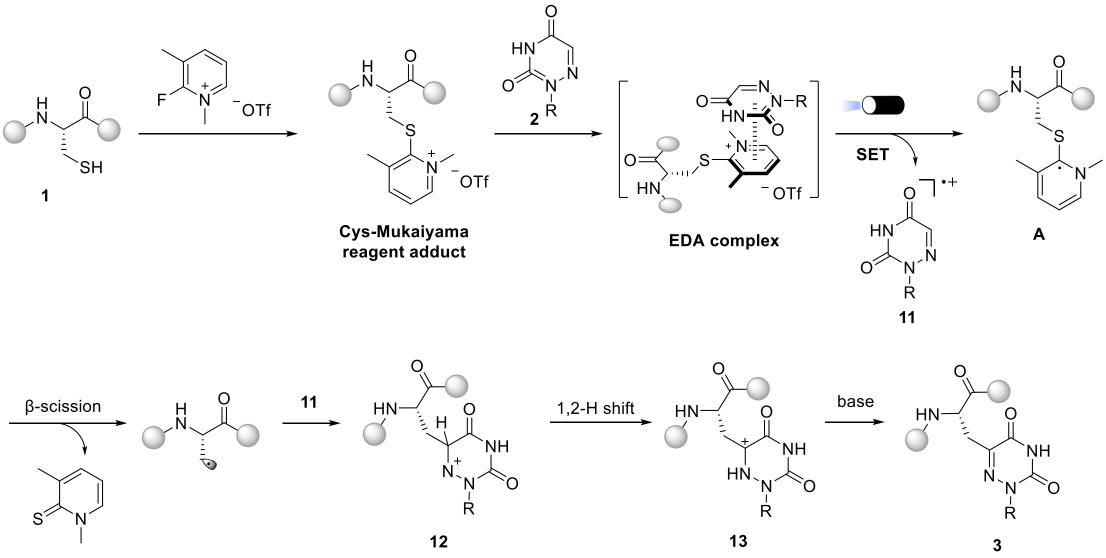

Figure S53a. Proposed mechanism

Drawing on previous studies (*Chem. Eur. J.* **2019**, *25*, 8240; *Angew. Chem. Int. Ed.* **2019**, *58*,

5697) and our own work (*Angew. Chem. Int. Ed.* **2020**, *59*, 7462), we propose a mechanism for the formation of Ala radicals in reactions employing Hantzsch ester or NADH as single-electron reductant (Fig. S53b).

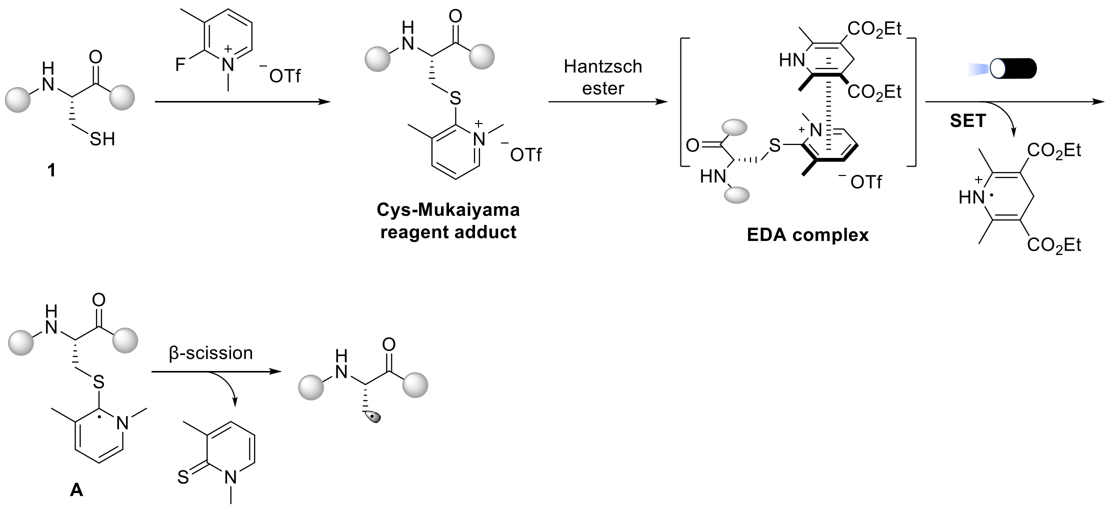

Figure S53b. Ala radical formation using Hantzsch ester or NADH as single-electron reductant.

7. Modifications of bovine serum albumin (BSA)

DTHKSEIAHRFKDLGEEHFKGLVLIAFSQYLQQCPFDEHVKLVNELTEFAKTCVADESHAG
CEKSLHTLFGDELCKVASLRETYGDMADCCEKQEPERNECFLSHKDDSPDLPKLKPDPNT
LCDEFKADEKKFWGKYLYEIARRHPYFYAPELLYYANKYNGVFQECCQAEDKGACLLPKI
ETMREKVLTSSARQRLRCASIQKFGERALKAWSVARLSQKFPKAEFVEVTKLVTDLTKVH
KECCHGDLLECADDRADLAKYICDNQDTISSKLKECCDKPLLEKSHCIAEVEKDAIPENLP
PLTADFAEDKDVCKNYQEAKDAFLGSFLYEYSRRHPEYAVSVLLRLAKEYEATLEECCAK
DDPHACYSTVFDKLKHLVDEPQNLIKQNCDQFEKLGEYGFQNALIVRYTRKVPQVSTPTL
VEVSRSLGKVGTRCCTKPESERMPCTEDYLSLILNRLCVLHEKTPVSEKVTKCCTESLVNR
RPCFSALTPDETYVPKAFDEKLFTFHADICTLPDTEKQIKKQTALVELLKHKPKATEEQLKT
VMENFVAFVDKCCAADDKEACFAVEGPKLVVSTQTALA

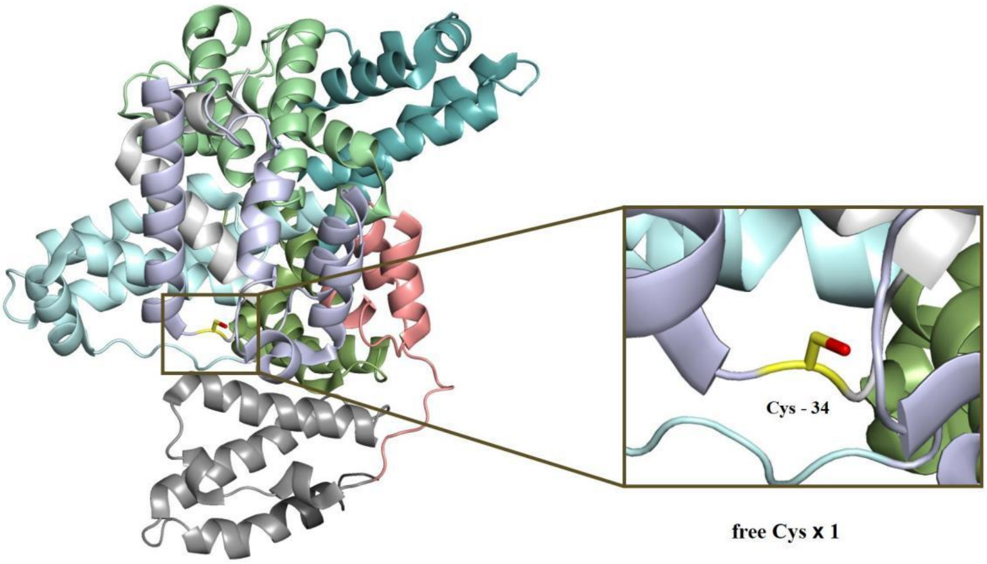

BSA (66.4 kDa)

The protein modification reaction was monitored and evaluated on a Thermo Scientific Orbitrap IQ-X Tribrid coupled to an Acquity UPLC® Protein BEH C4 column (300 Å, 1.7 µm, 2.1 mm × 100mm). Solvent A, water with 0.1% formic acid and solvent B, 100% acetonitrile were used as the mobile phase at a flow rate of 0.3 mL/min, the gradient was programmed as follows: 5% B in 2 min, then 5% B to 95% B from 2 min to 12 min. The electrospray source (ESI) was operated in positive and negative ion modes at 3800 V and 1800 V, respectively. Total mass spectra were reconstructed from the ion series using the intact mass analysis of Thermo BioPharma Finder software according to the manufacturer's instructions. A typical analysis of a protein is described below. The combined ion series and deconvoluted spectra are shown below.

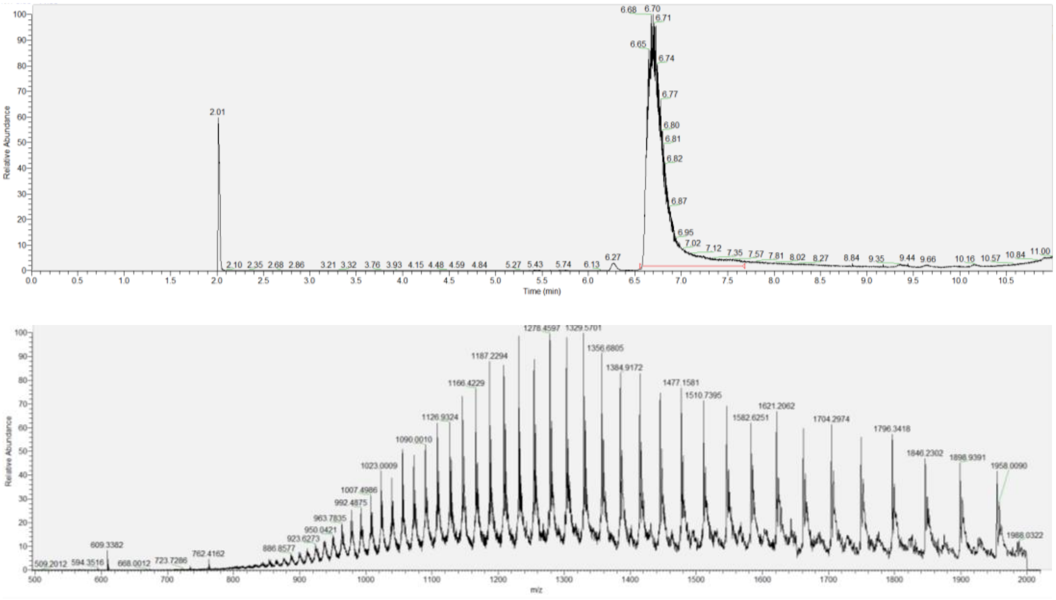

Figure S54. The total ion current spectrum and the combined ion series of **BSA**.

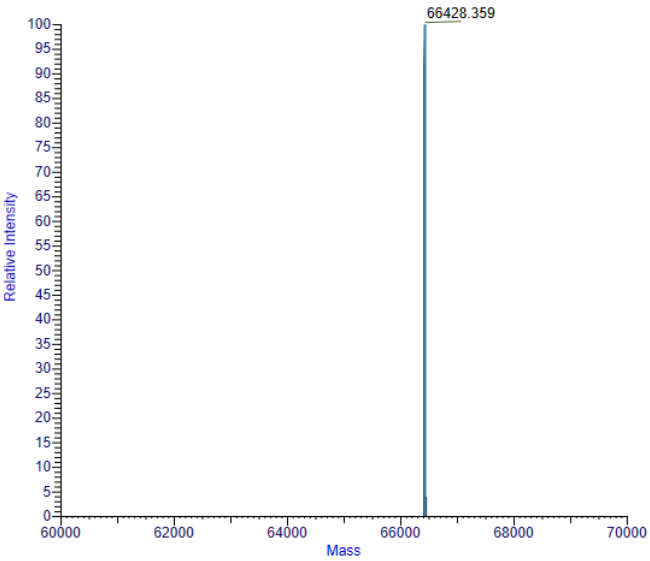

Figure S55. The deconvoluted spectrum of **BSA**. The expected molecular weight was 66428, and the observed molecular weight was 66428.

The conversion rate of proteins can be calculated using the following formula:

$$conversion\ rate\ \% = \frac{B2}{B1 + B2} \times 100$$

B1: The mass intensity of protein-related peak in the reaction mixture.

B2: The mass intensity of protein conjugation in the reaction mixture.

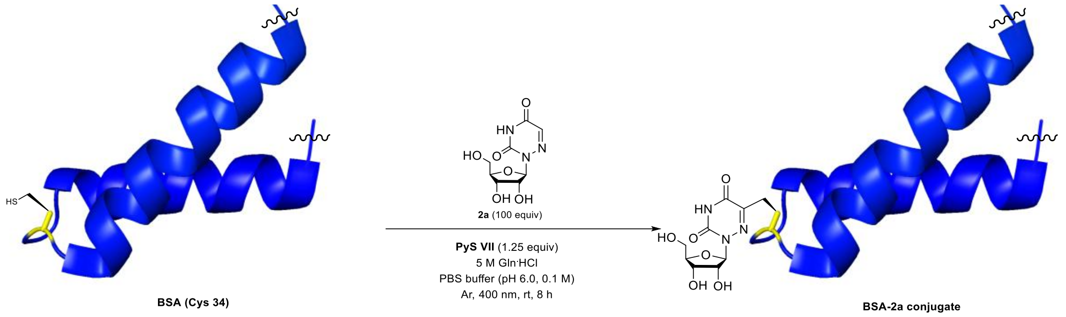

Pyridinium Salt **VII** (0.7 mg) was dissolved in ddH$_2$O (0.25 mL), Gln-HCl (0.5 g, 5 M) was dissolved in PBS buffer (0.1 M, pH 6.0, 1 mL). Then BSA (2.7 mg, 0.04 μmol) was dissolved in 175 μL of 5 M Gln-HCl PBS buffer (0.1 M, pH 6.0) and 5 μL pyridinium salt **VII** solution (10 mM) was added. The reaction mixture was shaken on a shaker for 10 min at room temperature. Then, **2a** (2.5 mg) was dissolved in 40 μL PBS buffer (0.1 M, pH 6.0) and 10 μL MeCN. 20 μl of **2a** solution (200 mM) was mixed with the reaction solution of pyridinium salt **VII** and BSA in the glovebox. The reaction mixtures were transformed into quartz test tube and reacted under 400 nm for 8 hours, while adding a fan to keep room temperature the reaction.

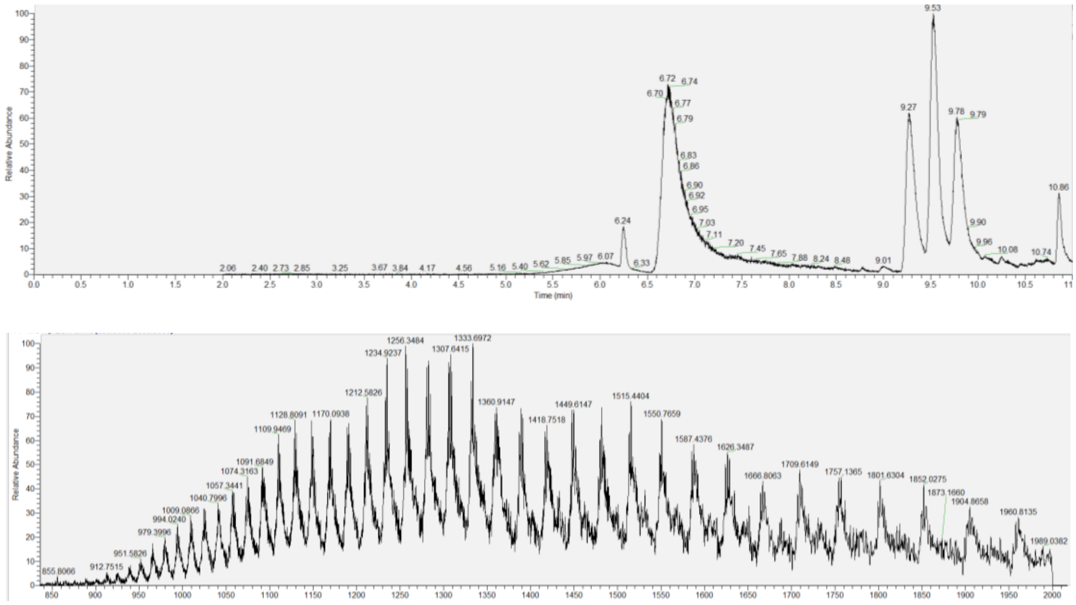

Figure S56. The total ion current spectrum and the combined ion series of the **BSA-2a** conjugate reaction.

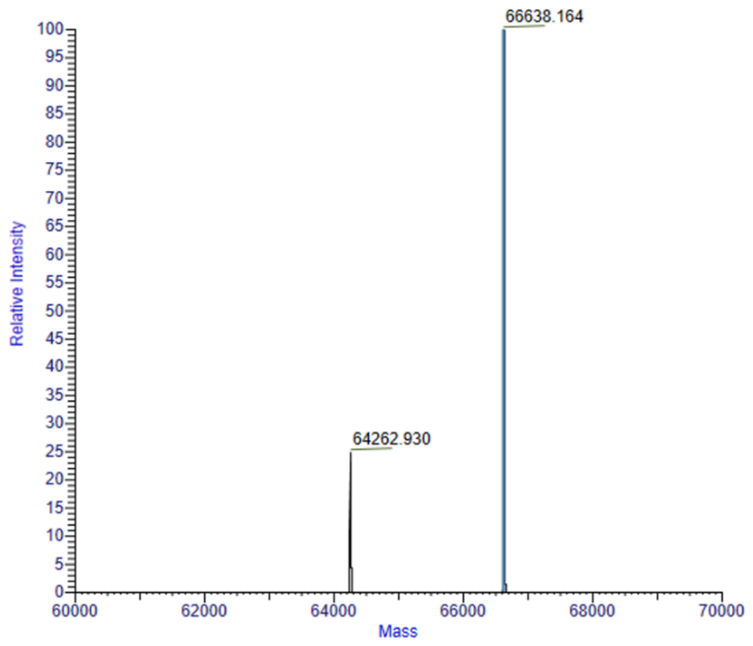

Average Mass	Intensity	Relative Abundance(%)	Fractional Abundance(%)
66638.164	5.30 E + 06	100.00	80.06
64262.930	1.32 E + 06	24.91	19.94

Figure S57. The deconvoluted spectrum of **BSA-2a conjugate**. The expected molecular weight was 66639, and the observed molecular weight was 66638.

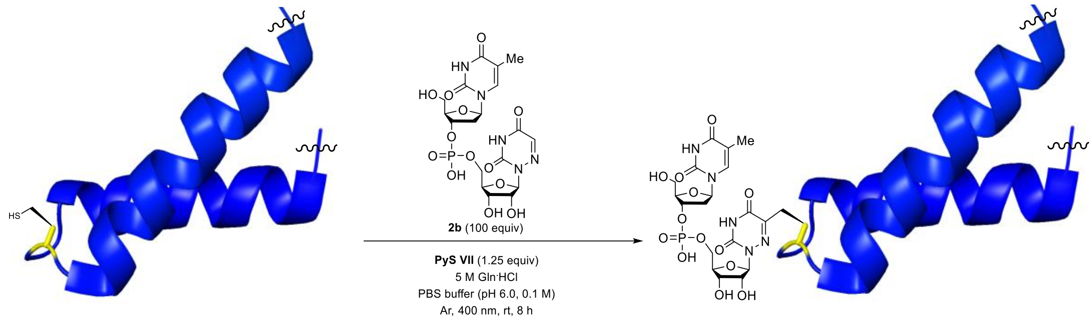

BSA (Cys 34)

BSA-2b conjugate

Pyridinium Salt **VII** (0.7 mg) was dissolved in ddH_2O (0.25 mL), Gln-HCl (0.5 g, 5 M) was dissolved in PBS buffer (0.1 M, pH 6.0, 1 mL). Then BSA (2.7 mg, 0.04 μmol) was dissolved in 175 μL of 5 M Gln-HCl PBS buffer (0.1 M, pH 6.0) and 5 μL pyridinium salt **VII** solution (10 mM) was added. The reaction mixture was shaken on a shaker for 10 min at room temperature. Then, **2b** (5.5 mg) was dissolved in 40 μL PBS buffer (0.1 M, pH 6.0) and 10 μL MeCN. 20 μl of **2b** solution (200 mM) was mixed with the reaction solution of pyridinium salt **VII** and BSA in the glovebox. The reaction mixtures were transformed into quartz test tube and reacted under 400 nm for 8 hours, while adding a fan to keep room temperature the reaction.

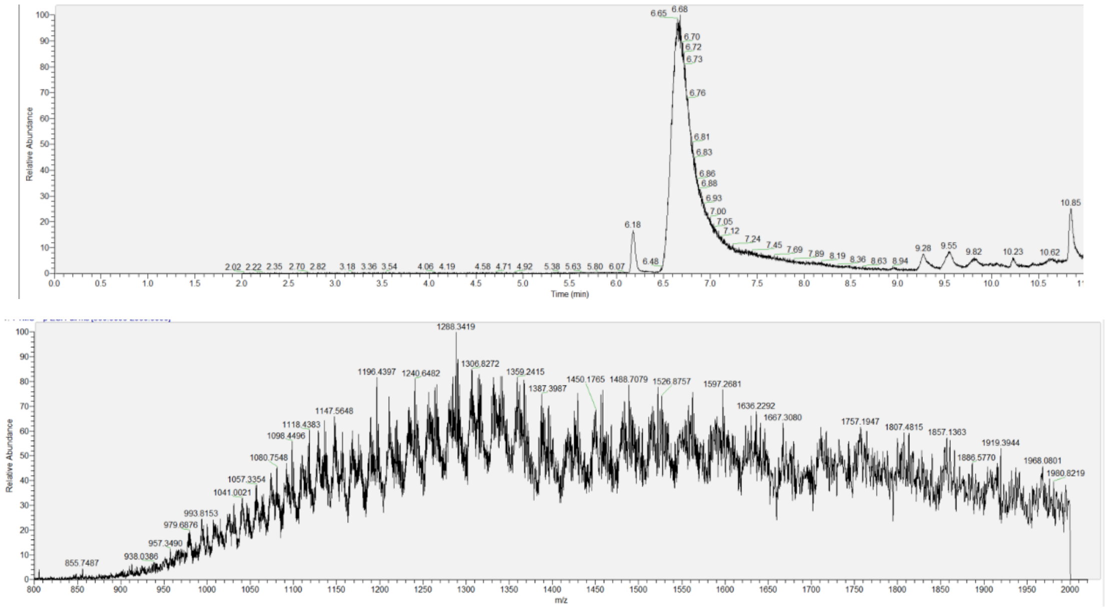

Figure S58. The total ion current spectrum and the combined ion series of **BSA-2b** conjugate reaction.

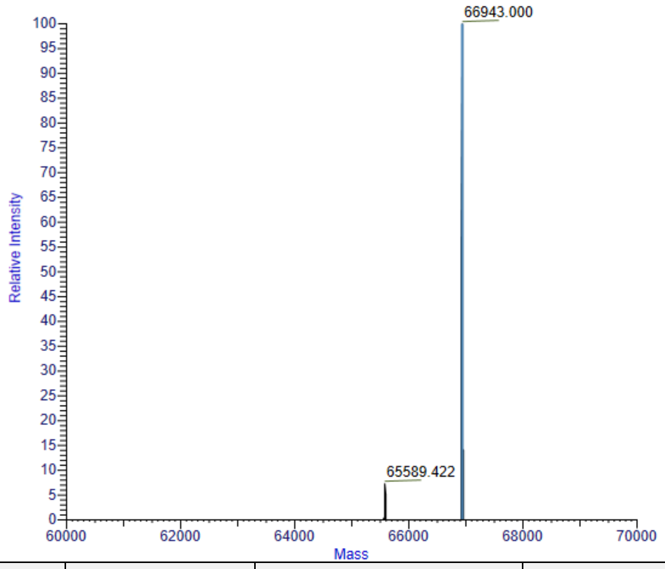

Average Mass	Intensity	Relative Abundance(%)	Fractional Abundance(%)
66943.000	3.74 E + 06	100.00	93.19
65589.422	2.73 E + 05	7.31	6.81

Figure S59. The deconvoluted spectrum of **BSA-2b conjugate**. The expected molecular weight was 66943, and the observed molecular weight was 66943.

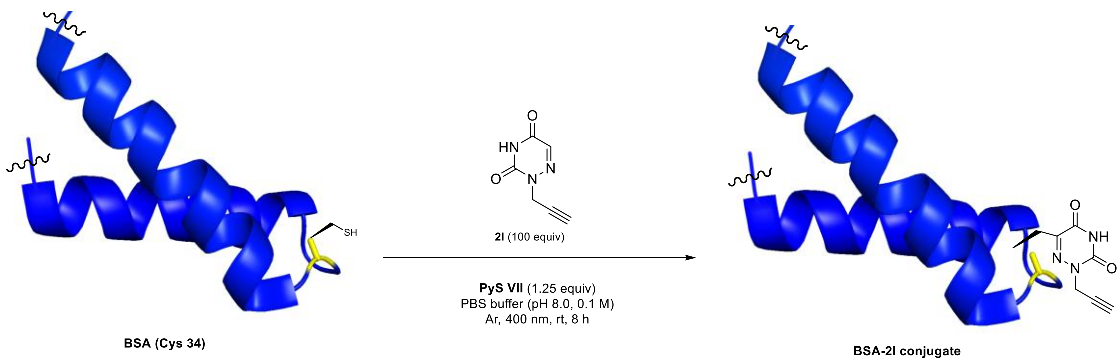

Pyridinium Salt **VII** (0.7 mg) was dissolved in ddH$_2$O (0.25 mL). Then BSA (2.7 mg, 0.04 μmol) was dissolved in 175 μL of PBS buffer (0.1 M, pH 8.0) and 5 μL pyridinium salt **VII** solution (10 mM) was added. The reaction mixture was shaken on a shaker for 10 min at room temperature. Then, **2l** (1.5 mg) was dissolved in 40 μL PBS buffer (0.1 M, pH 8.0) and 10 μL MeCN. 20 μl of **2l** solution (200 mM) was mixed with the reaction solution of pyridinium salt **VII** and BSA in the glovebox. The reaction mixtures were transformed into quartz test tube and reacted under 400 nm for 8 hours, while adding a fan to keep room temperature the reaction.

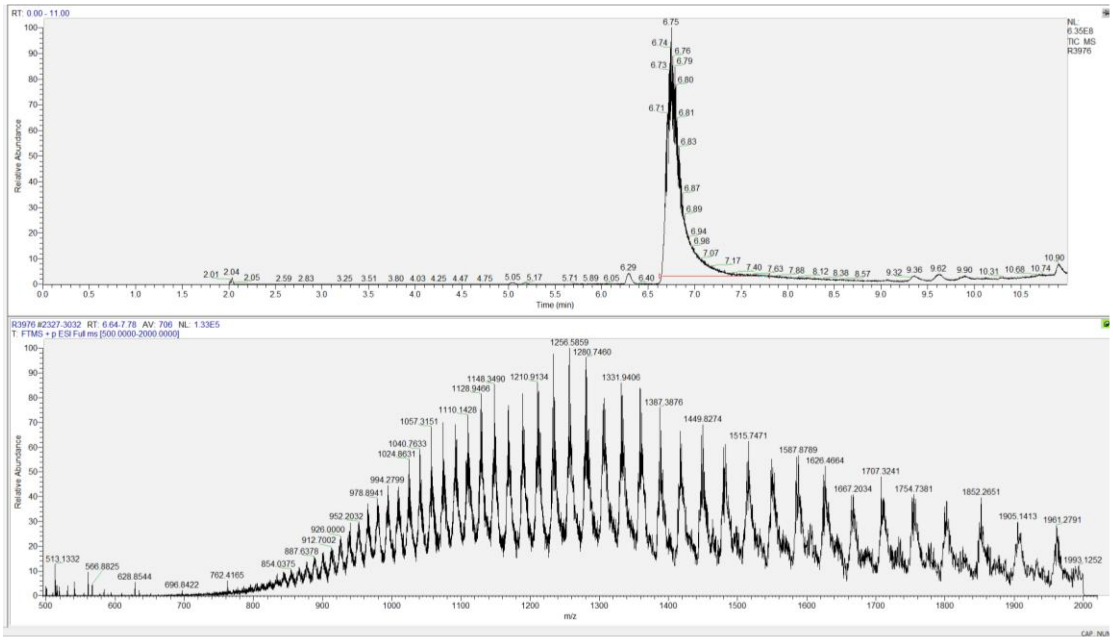

Figure S60. The total ion current spectrum and the combined ion series of **BSA-2l** conjugate reaction.

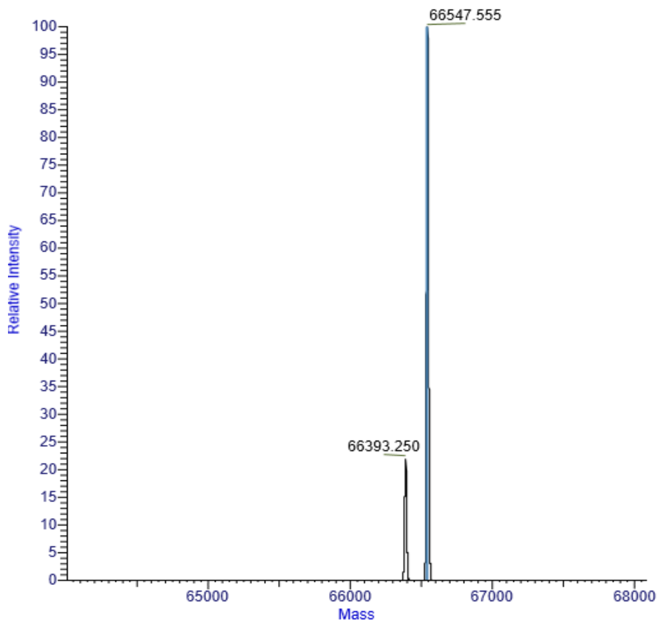

Average Mass	Intensity	Relative Abundance(%)	Fractional Abundance(%)
66547.555	1.43 E + 07	100.00	82.03
66393.250	3.14 E + 07	21.91	17.97

Figure S61. The deconvoluted spectrum of **BSA-2l** conjugate. The expected molecular weight was 66547, and the observed molecular weight was 66547.

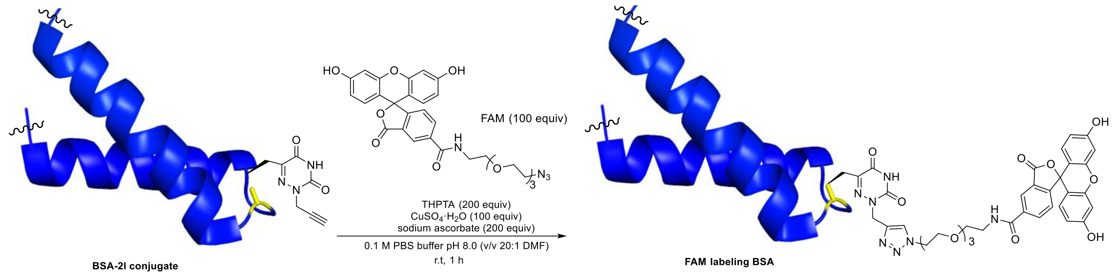

After the reaction of **BSA-2l** conjugate is completed, the mixture of **FAM-PEG$_3$-N$_3$** (100 equiv), THPTA (200 equiv), $CuSO_4 \cdot 5H_2O$ (100 equiv), sodium ascorbate (200 equiv) in DMF (10 μL) was added to the **BSA-2l** conjugate reaction mixture. After the reaction mixture was shaken on a shaker for 1 h, an aliquot of each sample (10 μL) was diluted with ddH$_2$O (10 μL). Each sample (10 μL) was loaded onto a 12-well 12% SDS-PAGE gel. [11] The gel was run at room temperature and at 160 V for 150 min. In-gelfluorescence was imaged with a Typhoon FLA 9500 (GE) at 460 nm.

	1	2	3
**BSA**	+	+	+
**2l**	-	+	+
**FAM**	-	-	+

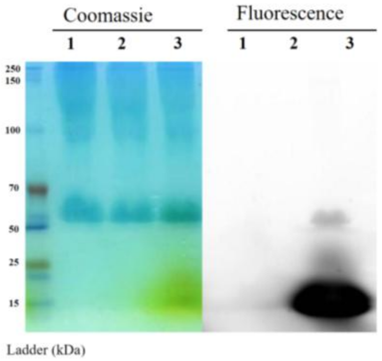

Figure S62. SDS-PEDG gel electrophoresis of **BSA, BSA-2l conjugate, FAM labeling BSA**. In-gelfluorescence was imaged with a Typhoon FLA 9500 (GE) at 460 nm. The gel was stained with Coomassie Brilliant Blue Fast Stain (Solarbio Coomassie Brilliant Blue Fast Staining solution).

8. References

[1] Wang, C.; Zhao, Z.; Ghadir, R.; Yang, D.; Zhang, Z.; Ding, Z.; Cao, Y.; Li, Y.; Fassler, R.; Reichmann, D.; et al. Peptide and protein cysteine modification enabled by hydrosulfuration of ynamide. *ACS Cent. Sci.* **2024**, *10*, 1742-1754.

[2] Xie, R.; Li, W.; Ge, Y.; Zhou, Y.; Xiao, G.; Zhao, Q.; Han, Y.; Li, Y.; Chen, G. Late-stage guanine C8–H alkylation of nucleosides, nucleotides, and oligonucleotides via photo-mediated Minisci reaction. *Nat Commun.* **2024**, *15*, 2549.

[3] Ching, S. M.; Tan, W. J.; Chua, K. L.; Lam, Y. Synthesis of cyclic di-nucleotidic acids as potential inhibitors targeting diguanylate cyclase. *Bioorganic & Medicinal Chemistry,* **2010**, *18*, 6657-6665.

[4] Seela, F.; Chittepu, P. Oligonucleotides containing 6-Aza-2'-deoxyuridine: synthesis, nucleobase protection, pH-dependent duplex stability, and metal-DNA formation. *J. Org. Chem.* **2007**, *72*, 4358-4366.

[5] Wilfried, B.; Sean Colm, T.; Andreas, H.; Udo, L.; Herve, G.; Karla, D.; Liliane, U.; Ana Lucia, J.; Anton, B.; Abbott, G.; et al. 1,2,4-Triazin-3,5-dione compounds for treating disorders that respond to modulation of the dopamine D3 receptor. International patent WO2009056625 A1, filed October 31, 2008, and granted May 7, 2009.

[6] Ghosh, P.; Kwon, N. Y.; Kim, S.; Han, S.; Lee, S. H.; An, W.; Mishra, N. K.; Han, S. B.; Kim, I. S. C−H methylation of iminoamido heterocycles with sulfur ylides. *Angew. Chem. Int. Ed.* **2021**, *60*, 191-196.

[7] Shiozaki, Y.; Sakurai, S.; Sakamoto, R.; Matsumoto, A.; Maruoka, K. Iron-catalyzed radical cleavage/C−C bond formation of acetal-derived alkylsilyl peroxides. *Chem. Asian J.* **2020**, *15*, 573-576.

[8] Panda, S. P.; Hota, S. K.; Dash, R.; Roy, L.; Murarka, S. Photodecarboxylative C–H alkylation of azauracils with N-(acyloxy)phthalimides. *Org. Lett.* **2023**, *25*, 3739-3744.

[9] Wang, C., Qi, R., Xue, H., Shen, Y., Chang, M., Chen, Y., Wang, R., Xu, Z. Visible-light-promoted C(sp^3)-H alkylation by intermolecular charge Transfer: preparation of unnatural α-amino acids and late-stage modification of peptides. *Angew. Chem. Int. Ed.* **2020**, *59,* 7461.

[10] Cheng, F., Fan, L., Lv, Q., Chen, X., Yu, B. Alkyl radicals from diacyl peroxides: metal-/base-/additive-free photocatalytic alkylation of N-heteroaromatics. *Green Chem.* **2023**, *25*, 7971.

[11] Bao, G.; Song, X.; Li, Y.; He, Z.; Zuo, Q.; E, R.; Yu, T.; Li, K.; Xie, J.; Sun, W.; et al. Orthogonal bioconjugation targeting cysteine-containing peptides and proteins using alkyl thianthrenium salts. *Nat.Commun.* **2024**, *15*, 6909.

9. NMR spectra of products

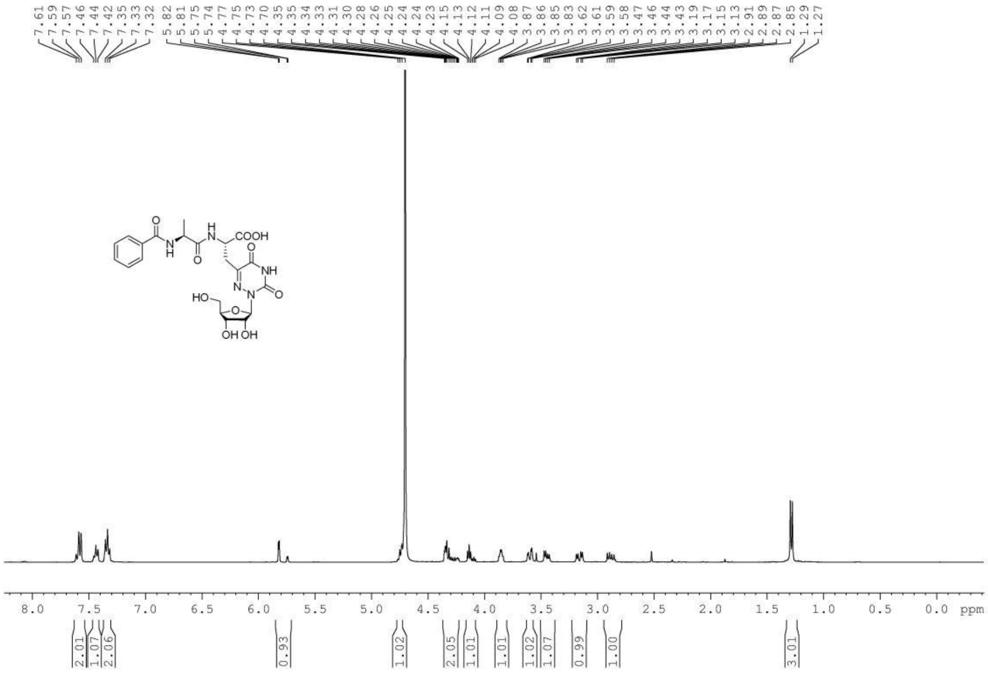

The ^{1}H NMR of **3aa** (D_2O, 400 MHz)

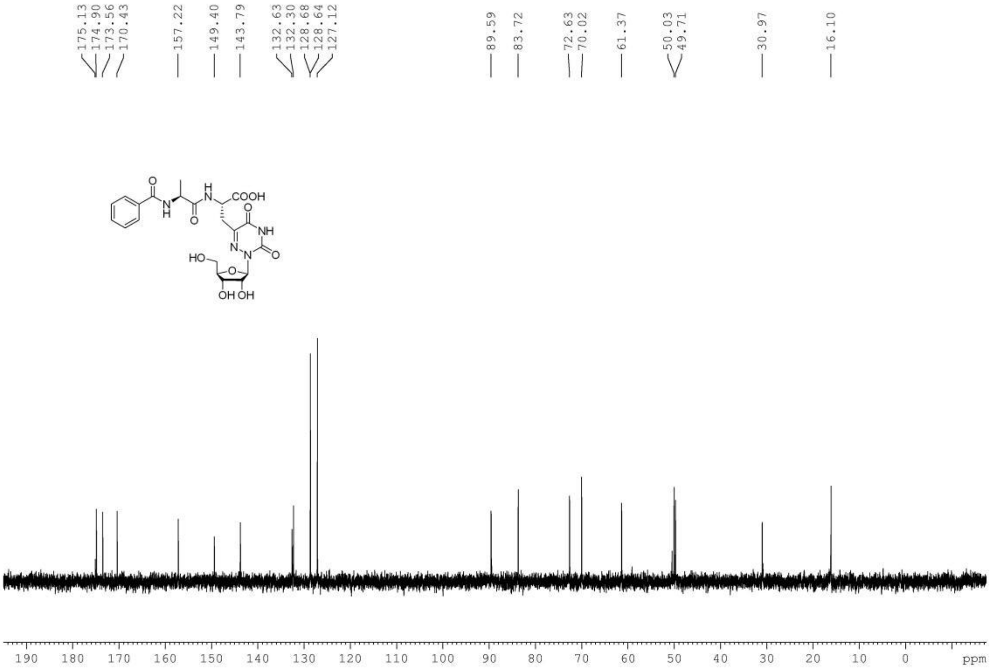

The ^{13}C NMR of **3aa** (D_2O, 101 MHz)

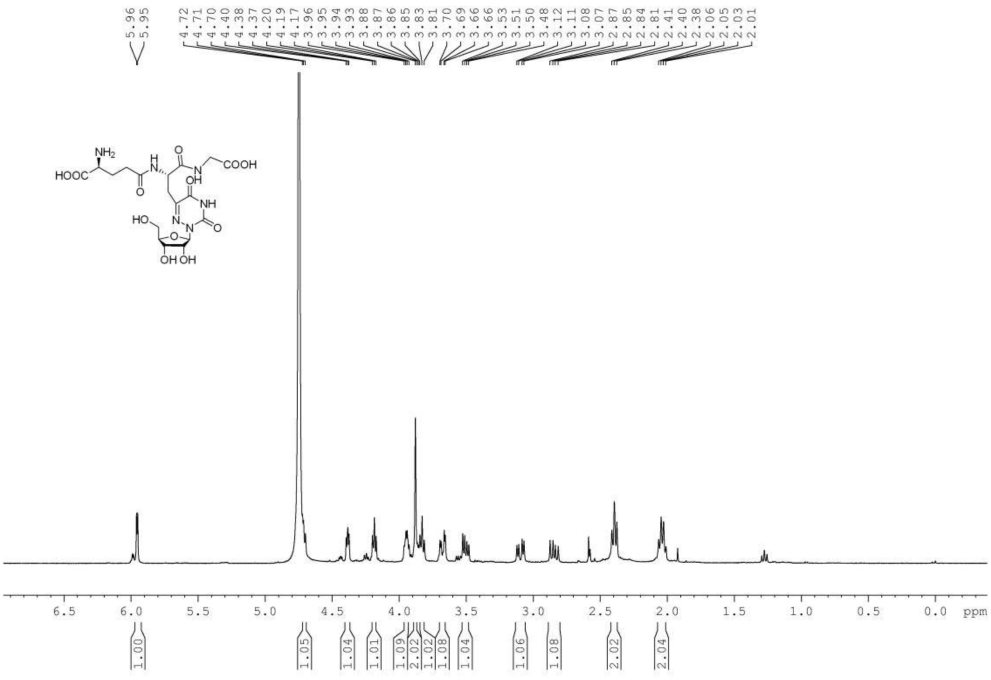

The ^{1}H NMR of **3ba** (D_2O, 400 MHz)

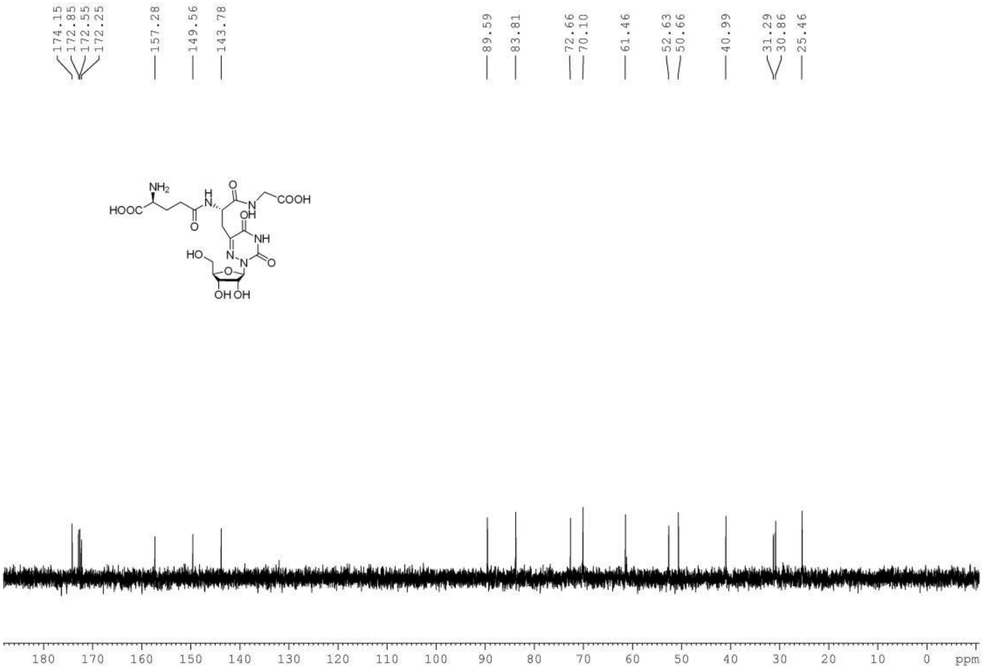

The ^{13}C NMR of **3ba** (D_2O, 101 MHz)

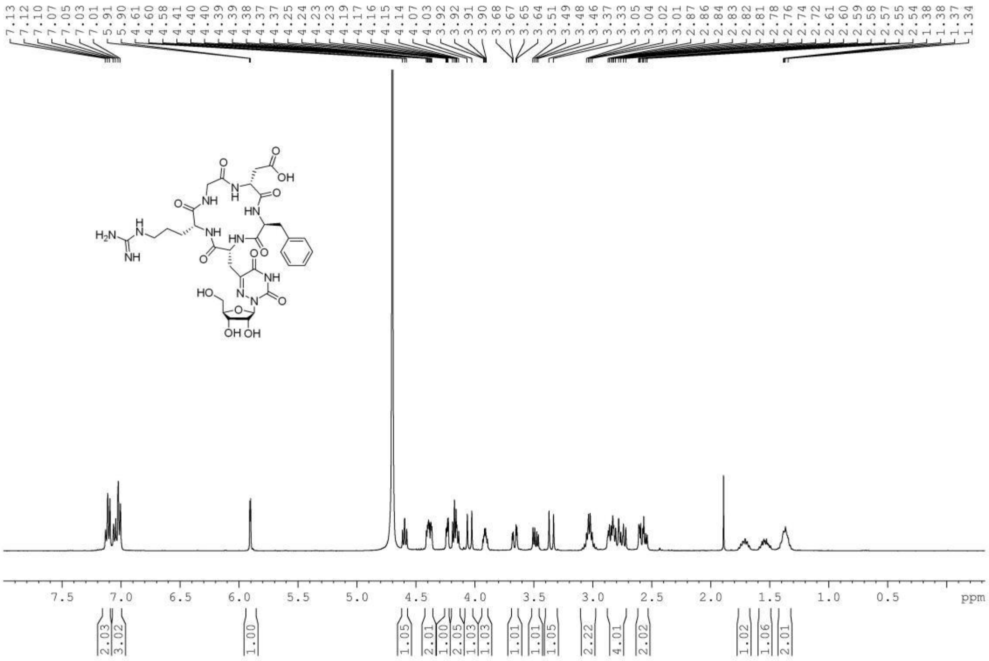

The ^{1}H NMR of **3ma** (D_2O, 400 MHz)

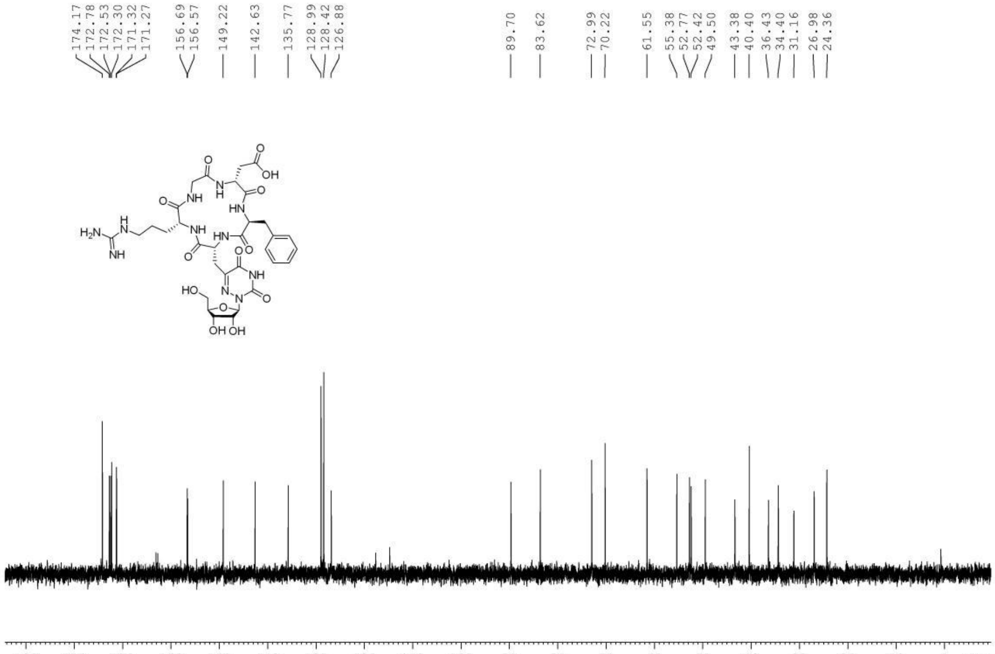

The ^{13}C NMR of **3ma** (D_2O, 101 MHz)

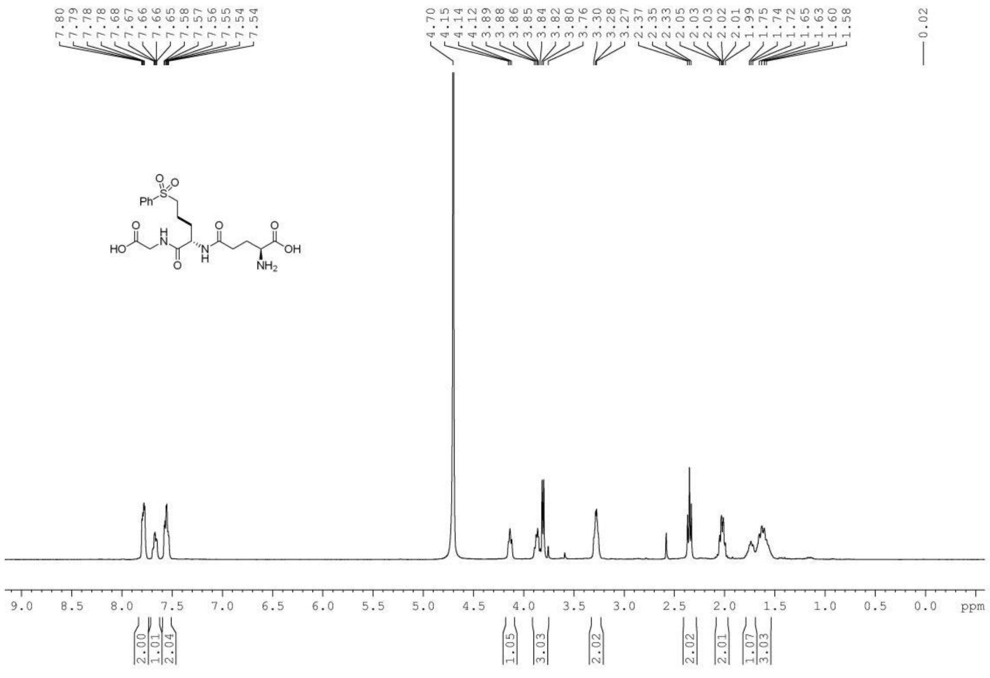

The ^{1}H NMR of **5ba** (D_2O, 400 MHz)

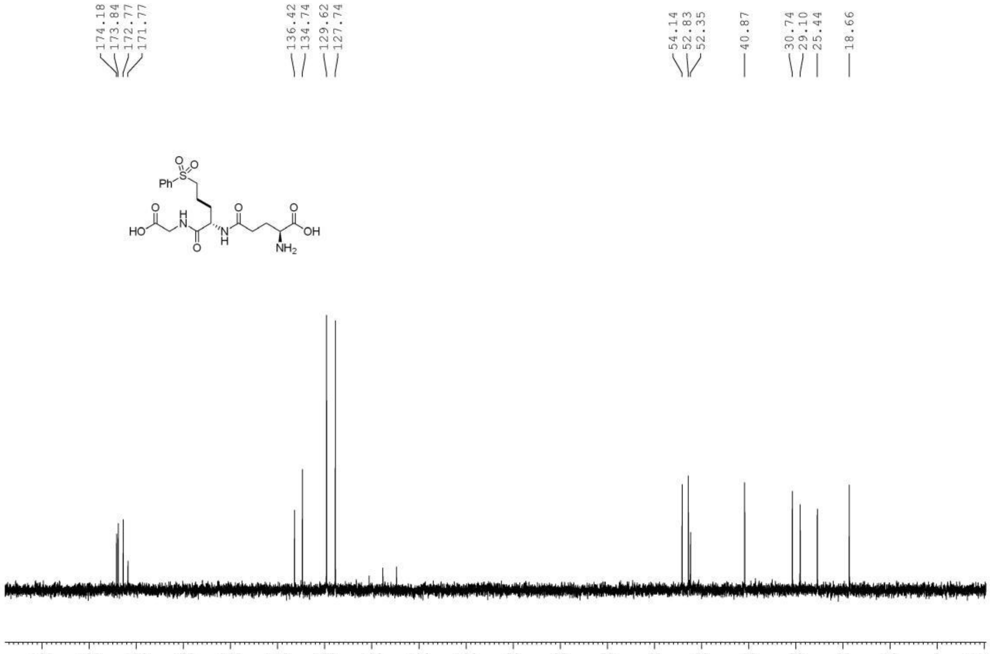

The ^{13}C NMR of **5ba** (D_2O, 101 MHz)

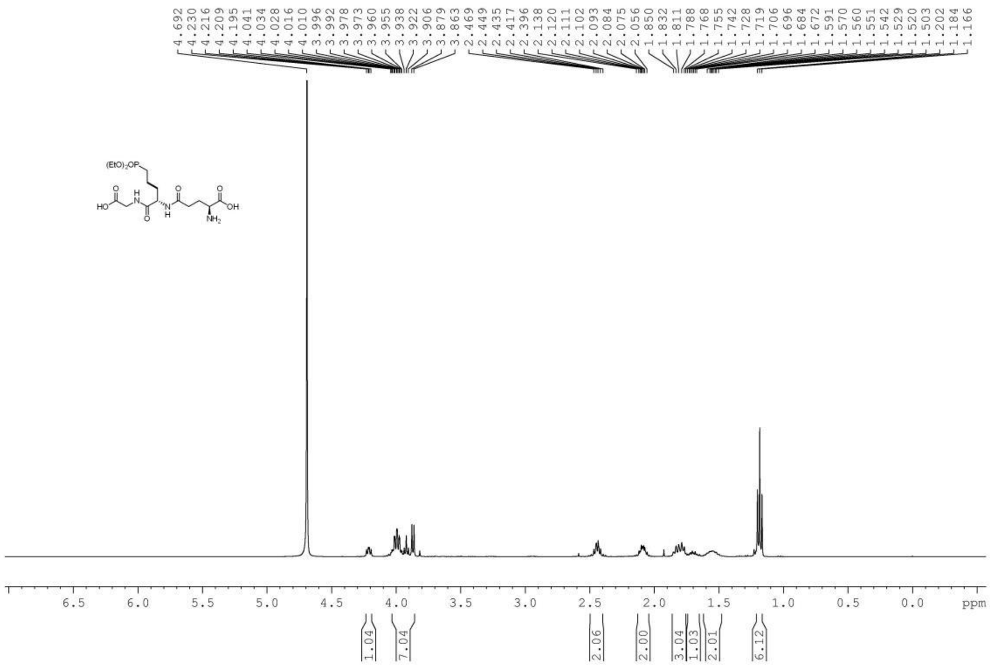

The ^{1}H NMR of **5bb** (D_2O, 400 MHz)

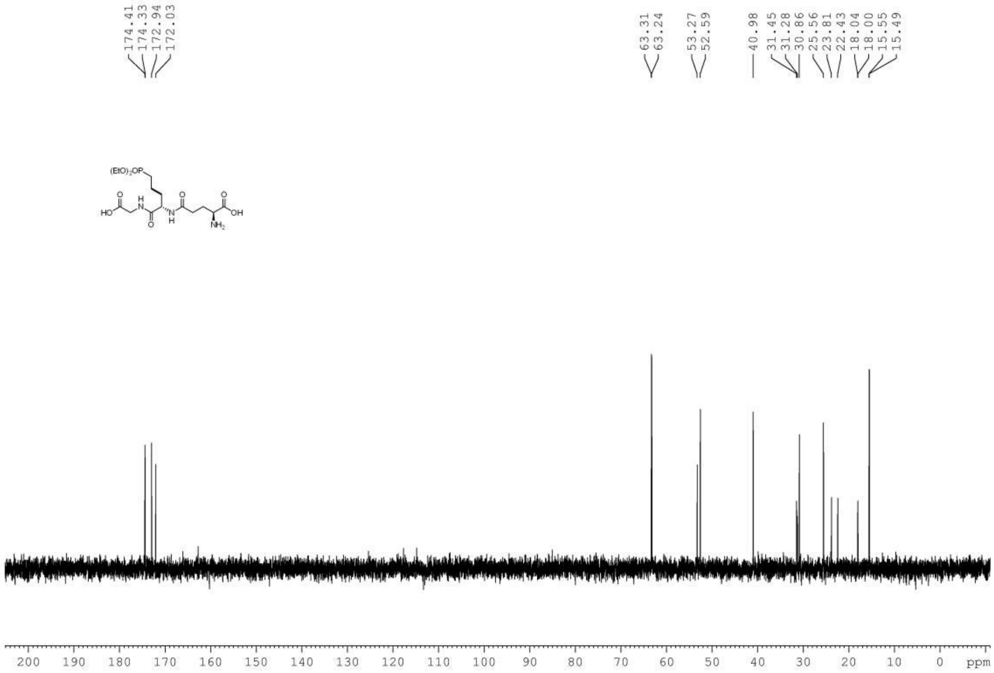

The ^{13}C NMR of **5bb** (D_2O, 101 MHz)

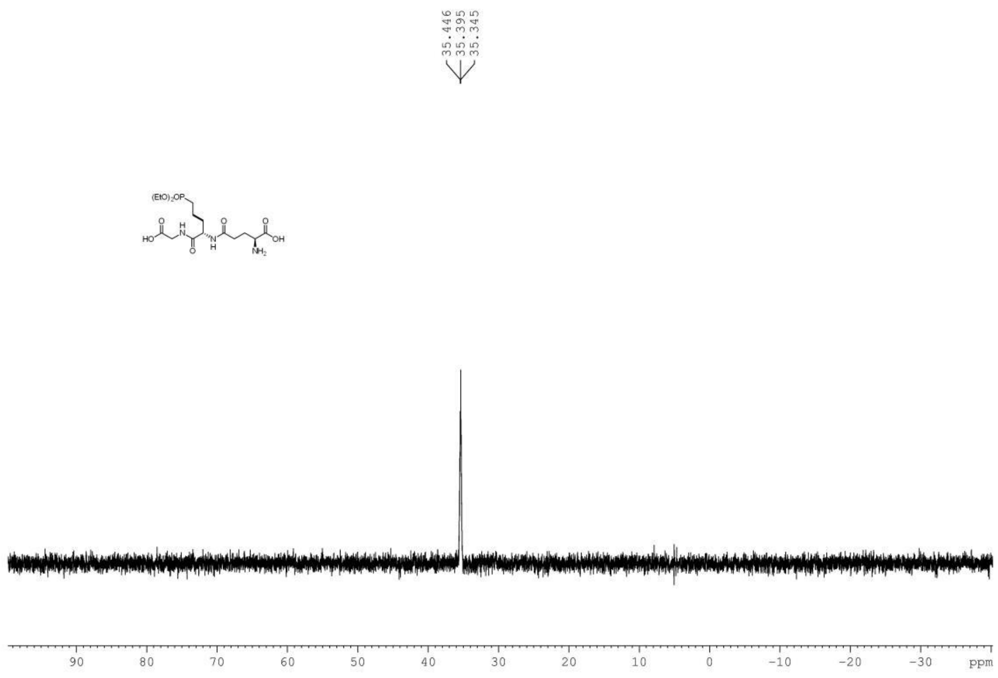

The ^{31}P NMR of **5bb** (D_2O, 162 MHz)

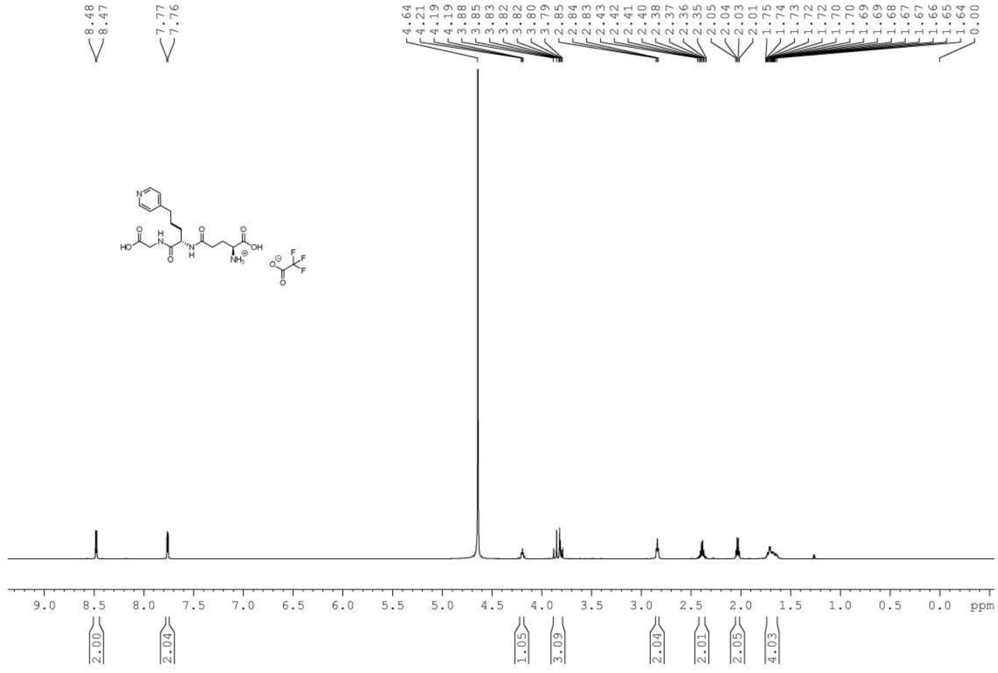

The ^{1}H NMR of **5bc** (D_2O, 600 MHz)

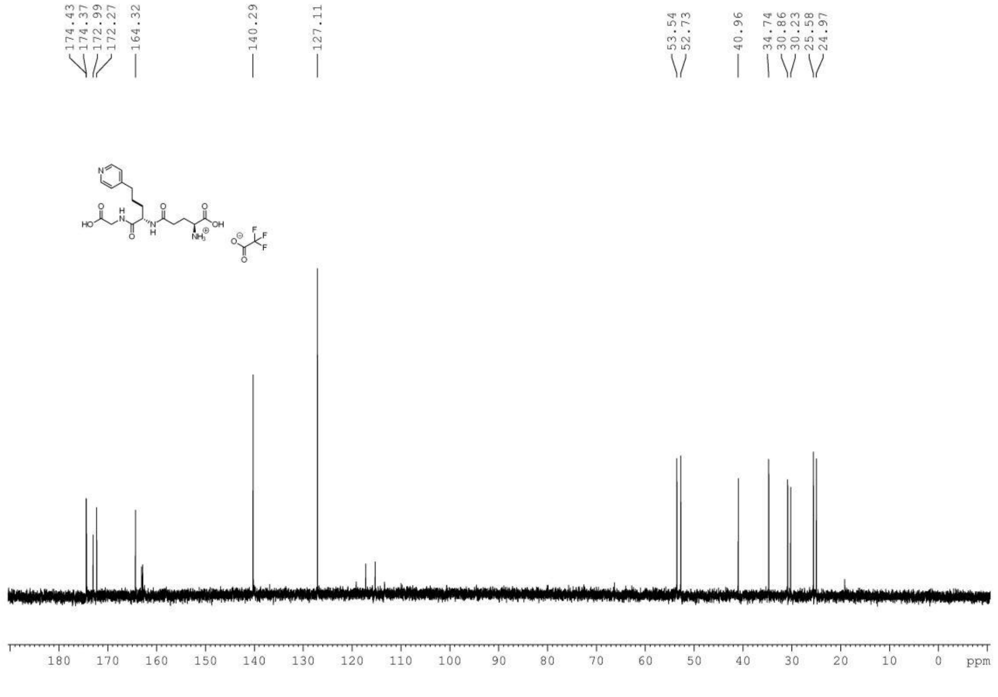

The ^{13}C NMR of **5bc** (D_2O, 151 MHz)

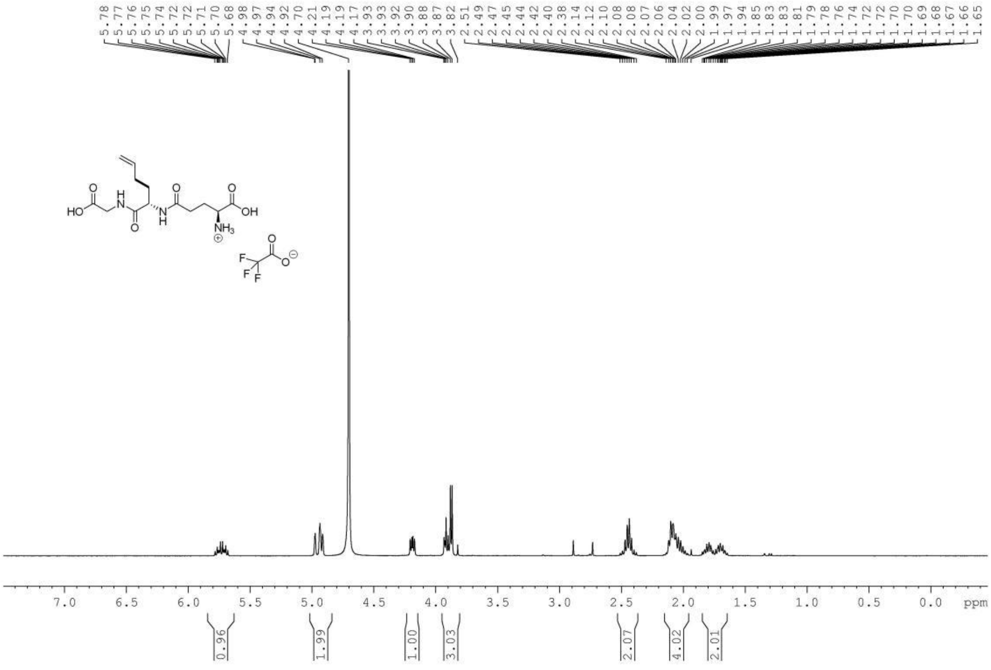

The ^{1}H NMR of **5bd** (D_2O, 400 MHz)

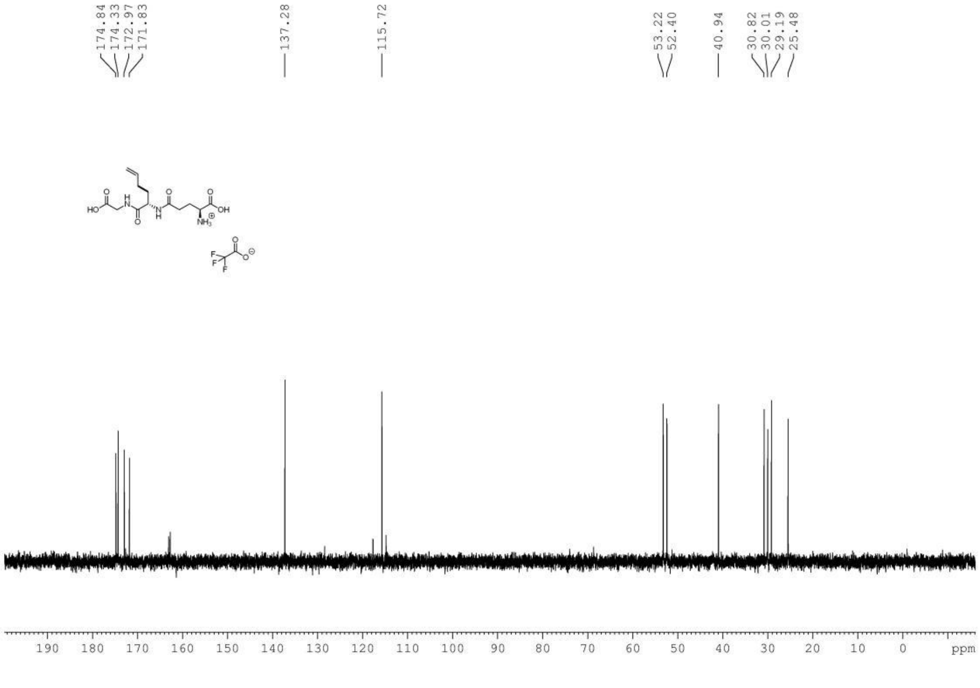

The ^{13}C NMR of **5bd** (D_2O, 101 MHz)

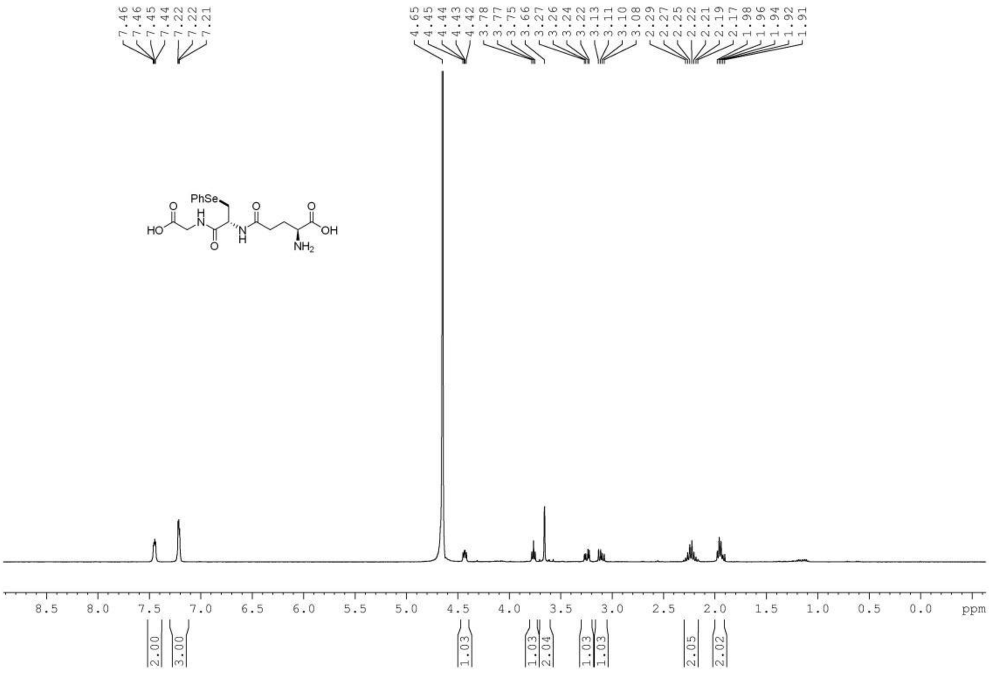

The ^{1}H NMR of **5be** (D_2O, 400 MHz)

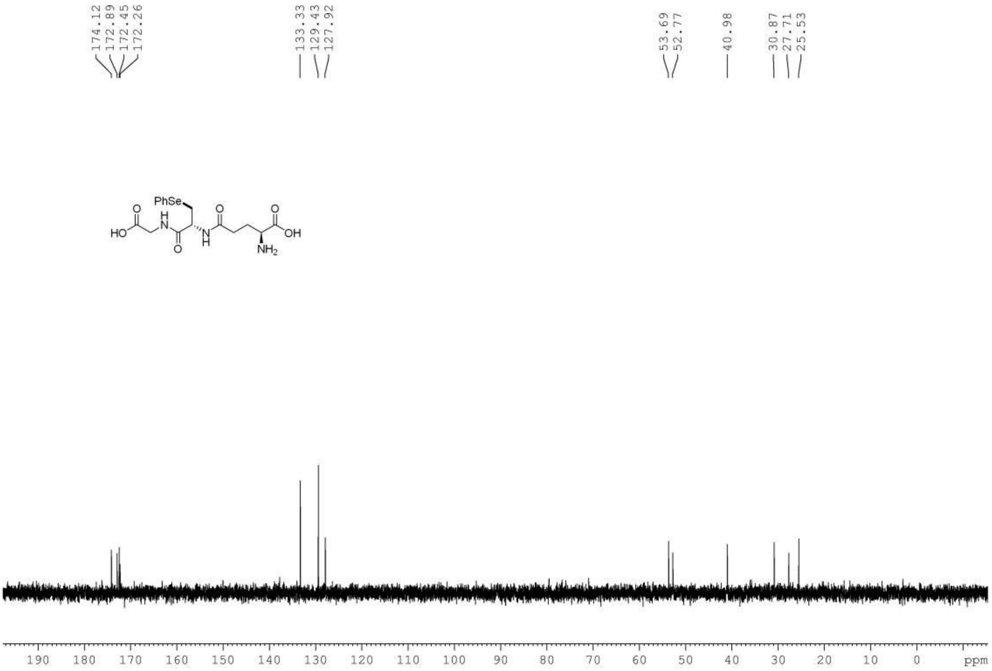

The ^{13}C NMR of **5be** (D_2O, 101 MHz)

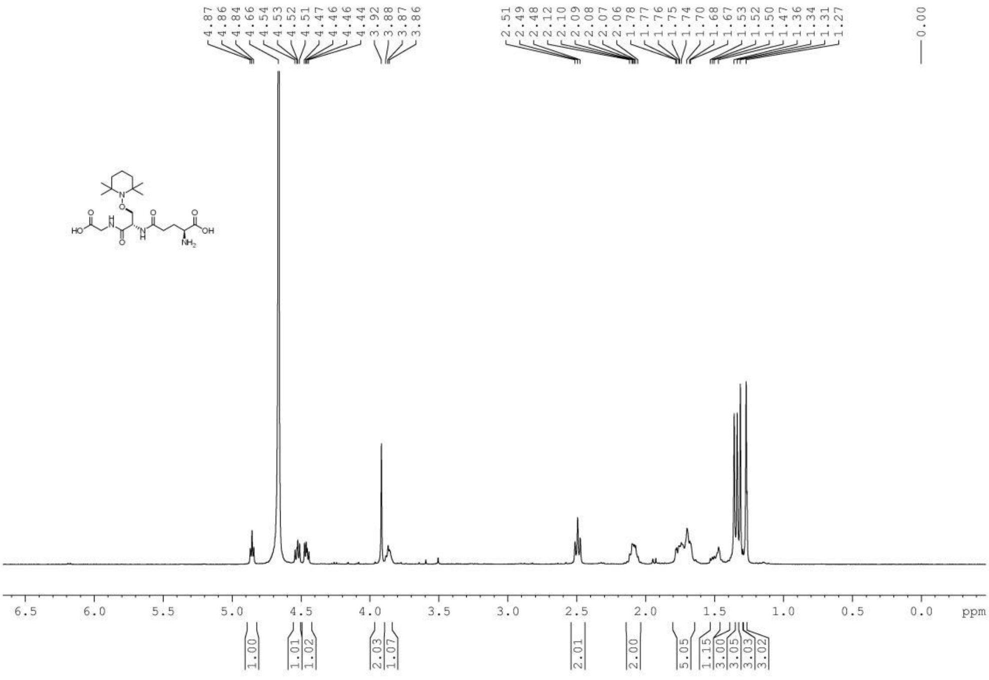

The ^{1}H NMR of **5bf** (D_2O, 400 MHz)

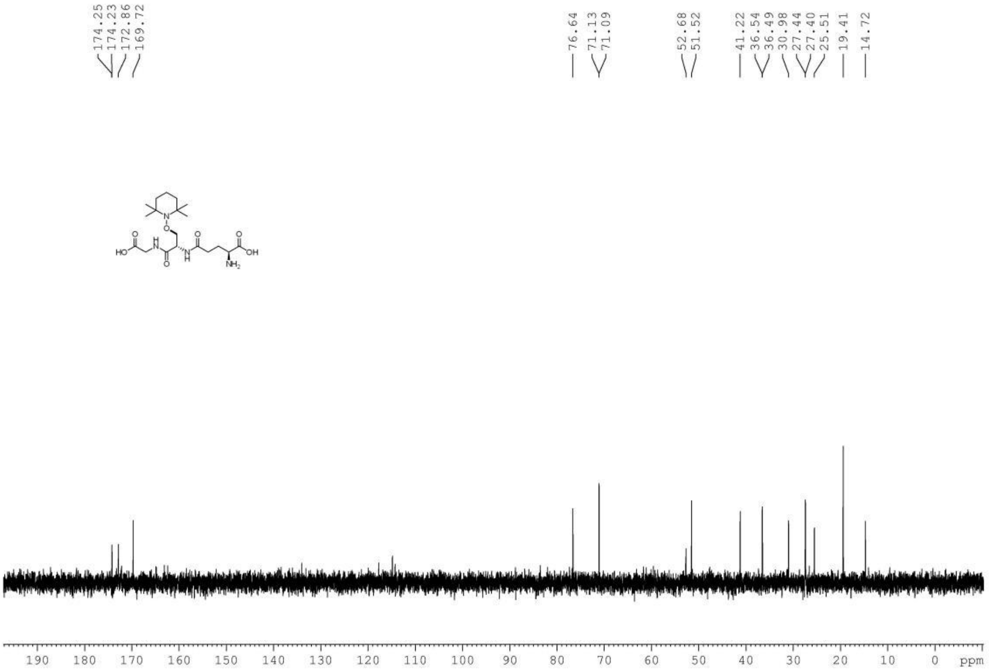

The ^{13}C NMR of **5bf** (D_2O, 101 MHz)

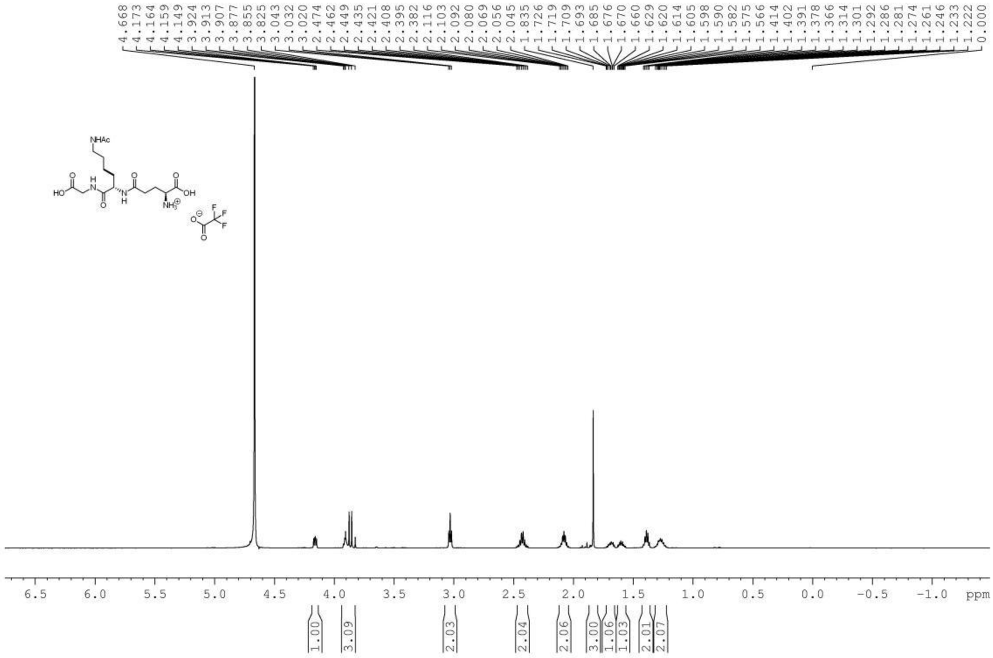

The ^{1}H NMR of **5bg** (D_2O, 600 MHz)

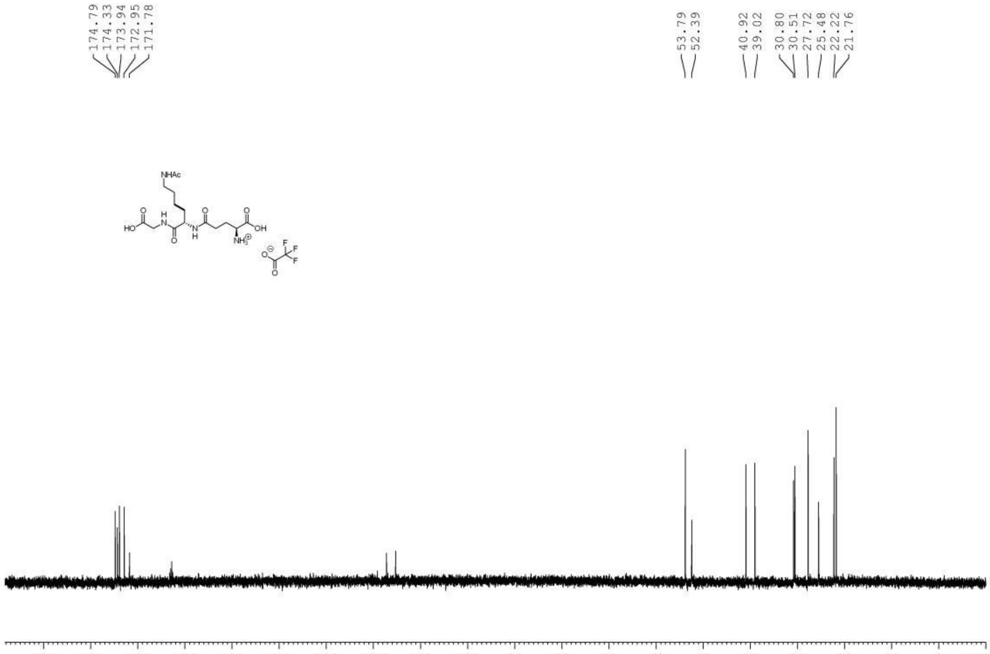

The ^{13}C NMR of **5bg** (D_2O, 151 MHz)
